# Partial depletion of yolk during zebrafish embryogenesis changes the dynamics of methionine cycle and metabolic genes

**DOI:** 10.1186/s12864-015-1654-6

**Published:** 2015-06-04

**Authors:** Yunxian Huang, Sam E.V. Linsen

**Affiliations:** Key Laboratory of Computational Biology, CAS-MPG Partner Institute for Computational Biology, Shanghai Institutes for Biological Sciences, Chinese Academy of Sciences, Shanghai, China

**Keywords:** Nutrition, Development, Methionine, Metabolism, Embryonic development, Metabolic syndrome

## Abstract

**Background:**

Limited nutrient availability during development is associated with metabolic diseases in adulthood. The molecular cause for these defects is unclear. Here, we investigate if transcriptional changes caused by developmental malnutrition reveal an early response that can be linked to metabolism and metabolic diseases.

**Results:**

We limited nutrient availability by removing yolk from zebrafish (Danio rerio) embryos. We then measured genome expression after 8, 24, 32 h post-fertilization (hpf) by RNA sequencing and 48 hpf by microarray profiling. We assessed the functional impact of deregulated genes by enrichment analysis of gene ontologies, pathways and CpG sites around the transcription start sites. Nutrient depletion during embryogenesis does not affect viability, but induces a bias towards female development. It induces subtle expression changes of metabolic genes: lipid transport, oxidative signaling, and glycolysis are affected during earlier stages, and hormonal signaling at 48 hpf. Co-citation analysis indicates association of deregulated genes to the metabolic syndrome, a known outcome of early-life nutrient depletion. Notably, deregulated methionine cycle genes indicate altered methyl donor availability. We find that the regulation of deregulated genes may be less dependent on methyl donor availability.

**Conclusions:**

The systemic response to reduced nutrient availability in zebrafish embryos affects metabolic pathways and can be linked to metabolic diseases. Further exploration of the reported zebrafish model system may elucidate the consequences of reduced nutrient availability during embryogenesis.

**Electronic supplementary material:**

The online version of this article (doi:10.1186/s12864-015-1654-6) contains supplementary material, which is available to authorized users.

## Background

Numerous mechanisms have evolved to cope with early-developmental nutrient stress [1–3]. The fine-tuning of physiological systems is driven by an (epi)gentic response towards environmental cues, a process known as developmental plasticity (reviewed in [4]).

The outcomes of developmental plasticity affect post-developmental health [5, 6]. In vertebrate systems, nutrient restriction during development has been linked to traits in adult life [7, 8]. Complications range from fetal growth restriction (FGR) and small-for-gestational age (SGA) phenotypes (which may lead to oxidative stress, neurological and growth problems) to obesity, type 2 diabetes, cardiovascular disease and osteoporosis. A combination of these symptoms is known as the metabolic syndrome [4, 9–11]. In humans, the risk to develop metabolic diseases later in life is most prominent if nutrient restriction occurs during early gestation, even when it resolves during later stages [12].

Developmental plasticity triggered by nutrient depletion during development would cause changes in embryonic gene expression, which may contribute to the phenotypes observed in adulthood. Here, we test this hypothesis experimentally by partially depleting zebrafish embryos from yolk at an early developmental stage. We analyze changes in gene expression at multiple time points after yolk depletion using RNA sequencing and microarrays. We find that partial yolk-depletion (YD) leads to subtle changes in gene expression that are statistically significant, thereby affecting expression changes of metabolic genes and hormonal signaling. By scoring the number of abstracts in which genes and diseases are both mentioned (so-called co-citation analysis) we find that deregulated genes associate to the metabolic syndrome. Therefore, the response shows similarities to the impact of developmental malnutrition in humans. Notably, deregulated methionine cycle genes indicate altered methyl donor availability. According to a comparably low amount of CpG sites around the transcription start site (TSS) of deregulated genes, regulation of these genes may be relatively insensitive to methyl donor availability. The overall response indicates that the developing zebrafish embryo responds to nutrient depletion by subtle fine-tuning of its metabolic pathways.

## Results and discussion

### Nutrient depletion during embryogenesis does not affect viability, but induces a bias towards female development

**Fig. 1:**
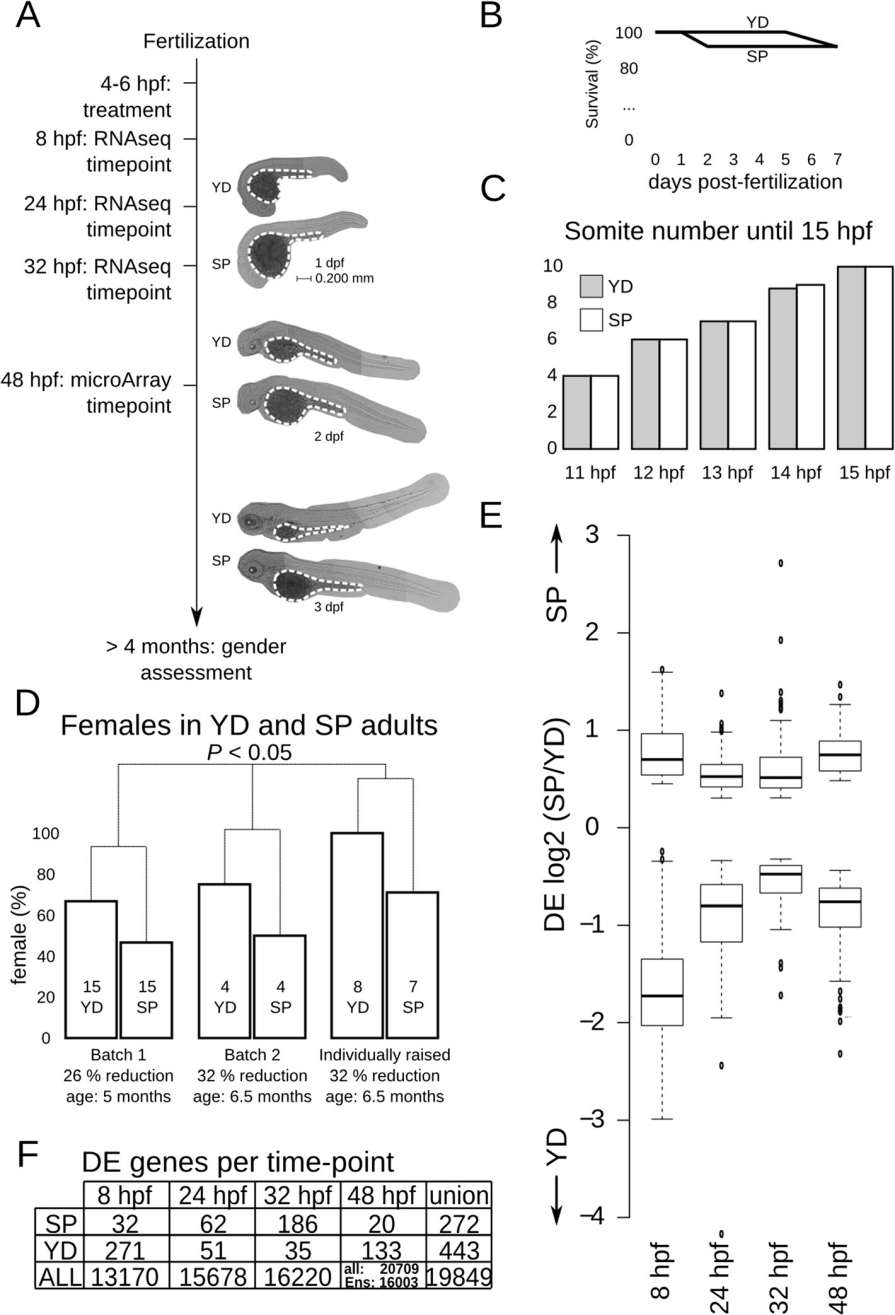
Study design, developmental outcome and differential expression datasets of YD vs. SP embryos. **a** Study design. Transcriptome profiles were generated at 4 time-points until 48 hpf. The images represent YD and SP embryos at the given time points. YD embryos show a reduced yolk volume (stained by ORO and marked within dashed line) and size. **b** Survival of YD (25 % yolk reduction) and SP embryos during one week post-fertilization in YD (treated) and SP (control-punctured) embryos during 7 days after treatment is >90 % in each group. **c** Average somite number from 11 hpf to 15 hpf in YD (24 % yolk reduction) and SP embryos. **d** Gender of adult fish, number of fish per treatment noted inside bars. A paired *T*-test for the three batches shows a significant increase in females. **e** Box-plot of differential expression (DE) levels at each time-point for genes that were significantly differentially expressed (DE genes). **f** Number of DE genes obtained at each time-point. SP: down-regulated in YD; YD: up-regulated in YD; ALL: all genes which passes expression level threshold and were included in this study (see methods for details); Ens: genes from microarray data with Ensembl identifier. Union indicates union of genes with Ensembl identifier

The study design is depicted in Fig. 1a. We removed yolk shortly after yolk-syncytial layer (YSL) formation, between 4–6 hpf. At 4 hpf, maternally deposited transcripts in yolk are expected to be reduced, as the yolk passage of maternal transcripts towards the embryo along ooplasmic streaming is completed around the 32 cell stage [13]. Also, maternal transcripts enriched in extra-embryonic tissue around 4 hpf localize to the extra-embryonic YSL [14], not to yolk. The YSL accommodates nutrient transport to the zebrafish embryo starting from 3 h post fertilization (hpf) [15]. Representative pictures of embryos show yolk depleted (YD) embryos and sham-punctured (SP) controls (Fig. 1a and Additional file 1). YD treatment did not affect survival: after removing 25 % of yolk content, the one-week survival was 92 % (22/24), compared to 92 % (23/25) in SP controls (Fig. 1b). The survival outcome is in agreement with previous studies [16, 17]. Also, embryo handling did not cause an early developmental delay in YD and SP embryos. At least until 9 h after treatment (15 hpf), somatogenesis was unaffected (Fig. 1c). We therefore exclude that differences in gene transcription between YD and SP embryos are due to lethality or developmental delay.

Both SP and YD embryos developed into healthy adult fish. In zebrafish, female development is induced when food availability, or growth rate, during early adulthood is increased [18]. We observed a higher number of females in adult YD fish (Fig. 1d). This was observed in fish raised in batches and fish raised individually, in tanks that were placed in alternating order to control for location and feeding-induced biases. Combined, the gender differences in two batches and individually raised fish leads to a statistically significant outcome in a paired 2-sided *T*-test (*P* < 0.05). We hypothesize that a higher number of females among YD adult fish could be indicative for increased food consumption or caloric storage up to early adulthood. Potentially, this could resemble the early onset of metabolic syndrome in humans: in humans, a high caloric intake and storage before adulthood increases the risk to develop the metabolic syndrome [19].

### YD induces subtle expression changes in embryogenesis

To study the effect of nutrient depletion on gene expression, we obtained transcriptome profiles at 8, 24 and 32 hpf by RNAseq and 48 hpf by microarray hybridization, each time point in triplicate (see methods for details). Despite platform-dependent biases, relative, not absolute, expression values obtained by RNAseq and microarray hybridization correlate well [20]. Differentially expressed (DE) genes could therefore be compared across all 4 time-points. Generally, DE was less than 2-fold, except for DE genes up-regulated in YD embryos at 8 hpf (Fig. 1e and Additional files 2, 3). We detected over a hundred DE genes at each time-point. The number of genes that passed threshold expression levels ranged from 13170 to 20709 (Fig. 1f, Additional files 4, 5). We verified differential expression by quantitative real-time PCR (qRT PCR). This analysis confirmed deregulation for 18 out of 19 tested genes (Additional file 6A,B). qRT PCR vs. RNAseq or microarray values correlated well, with a spearman correlation coefficient of 0.9 (Additional file 6C). RNAseq and microarray samples showed an overall high correlation between identical time-points (Additional file 6D).

### Transcriptional response to nutrient depletion involves metabolic genes

To uncover the pathways common to deregulated genes we performed functional enrichment analysis for the 4 time-points separately. We identified 58 unique molecular function gene-ontology (mfGO) terms [21] significantly enriched at least at one time-point (Additional file 7). As a score for the enrichment of mfGO terms over all 4 time-points combined, the product of *P*-values was calculated. Among the 10 lowest *P*-value products we found lipid transport, oxidative processes and hormone activity. These classes of genes are closely associated with core metabolism and are likely to be deregulated in a cellular response that aims to compensate for the lack of nutrients. We also looked into the functions of genes that were deregulated at least at 3 time-points. We discuss the significance of these classes and details on deregulated genes below.

### Apolipoprotein genes and other lipid-transporters are deregulated

**Fig. 2:**
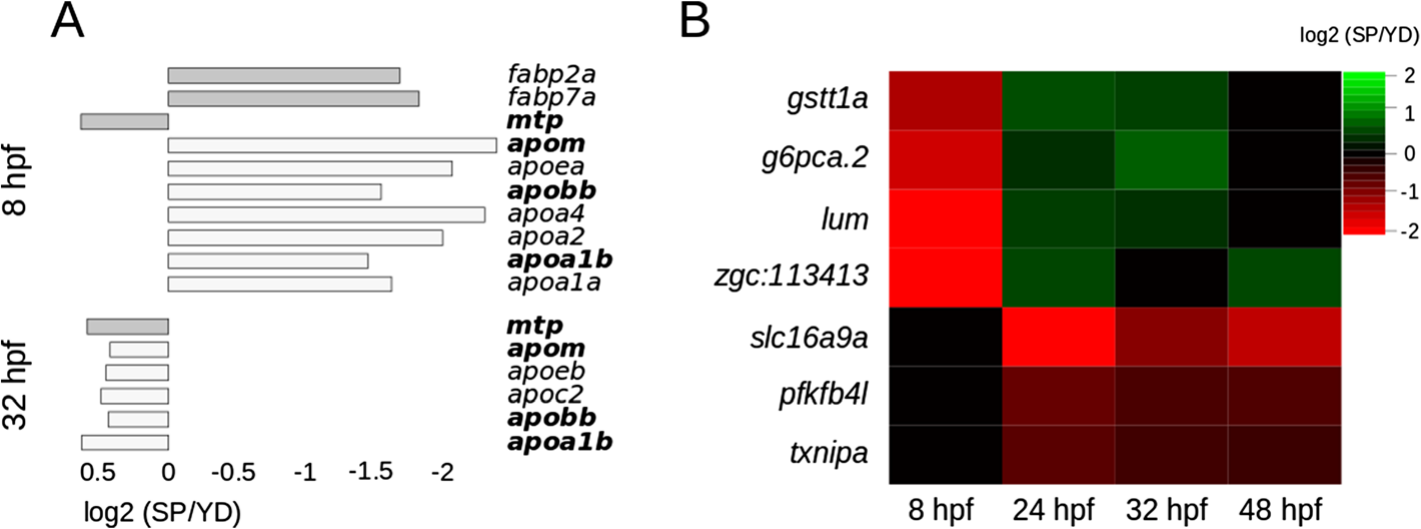
Stage-specific changes in gene expression and functional annotation. **a** Genes that are involved in lipid transport are dynamically DE between 8 and 32 hpf. Four genes (in bold) overlap with these two time-points. The enrichment of *apo* genes at each of these two time-points is significant (*P* < 0.001, see main text). **b** DE gene symbols that overlap three time-points, which is the largest overlap observed in this study

At 8 hpf, we identified 10 deregulated lipid-transport genes, 7 of which are *apolipoprotein* (*apo*) genes. At 32 hpf, we identified 6 lipid-transport genes, 5 of which are *apo* genes (Fig. 2a). Based on hypergeometric testing using the 28 known *apo* genes in zebrafish (obtained from www.ensembl.org), the elevated number of *apo* genes at 8 hpf as well as at 32 hpf is significant (*P* < 0.001). Deregulated lipid-transport genes are up-regulated in YD embryos at 8 hpf and down-regulated in YD at 32 hpf, except for *microsomal triglyceride transfer protein (mtp)*, which is down-regulated in YD embryos both at 8 hpf as well as 32 hpf. Yolk has a high lipid content [22]. We propose that lipid transporters respond dynamically to reduction in lipid availability due to a reduced yolk content.

### Hypoxia signaling is activated

Oxidative processes were notably enriched among deregulated genes. The observed down-regulation in YD embryos of *egln2* and *egln3* (regulators of hypoxia-inducible factors (Hifs) [23] and also known as *phd1 and phd3* [24]) at 32 hpf and 24/48 hpf respectively, complies with hypoxia signaling in YD embryos (*egln3* has been validated by qRT PCR, Additional file 6, see Additional file 2 for DE values). *Egln3* down-regulation in YD embryos is reminiscent with down-regulation of its human orthologue in placenta from fetal growth restricted (FGR) children [25]. Hypoxia signaling has also been observed in fetal murine hearts and brains from undernourished mothers [26, 27] and is consistent with pronounced oxidative stress in small-for-gestational age (SGA) newborns from undernourished mothers [9]. Potentially, oxidative processes in YD embryos relate to a modified metabolic demand during development. For instance, beside its roles in hypoxia signaling, *Egln3* (*Phd3* in mice) has been implicated in lipid and glucose metabolism [28].

### Enhanced glucose dependence in YD embryos

Seven DE genes in our dataset are DE at three time-points (Fig. 2b), of which 3 relate directly to glucose. Firstly, *glucose-6-phosphatase a, catalytic, tandem duplicate 2* is up-regulated in YD at 8 hpf and down-regulated in YD at 24 and 32 hpf. Glucose-6-phosphatases hydrolyze phosphate from glucose-6-phosphates, thereby releasing glucose, which completes gluconeogenesis. Second, *pfkfb4l* belongs to a class of genes that encode bi-functional enzymes that either stimulate gluconeogenesis or glycolysis; under hypoxic conditions in cell systems, it is up-regulated and expected to stimulate glycolysis [29]. This gene is up-regulated in YD at 24, 32 and 48 hpf. The disturbed glucose metabolism is furthermore exemplified by the up-regulation of *txnipa* in YD embryos from 24 hpf onwards, and *txnipb* at 24 hpf (DE of *txnipb* has been validated by qRT PCR, see Additional file 6). In mammalian systems, up-regulation of *txnip* in beta-cells reduces insulin levels and induces apoptosis, which leads to diabetes [30]. Based on the deregulation of these 3 genes as well as the oxidative signaling in YD embryos, it is expected that gluconeogenesis is down-regulated and glycolysis is up-regulated in YD embryos from 24 hpf onwards.

### Hormonal responses at 48 hpf

**Fig. 3:**
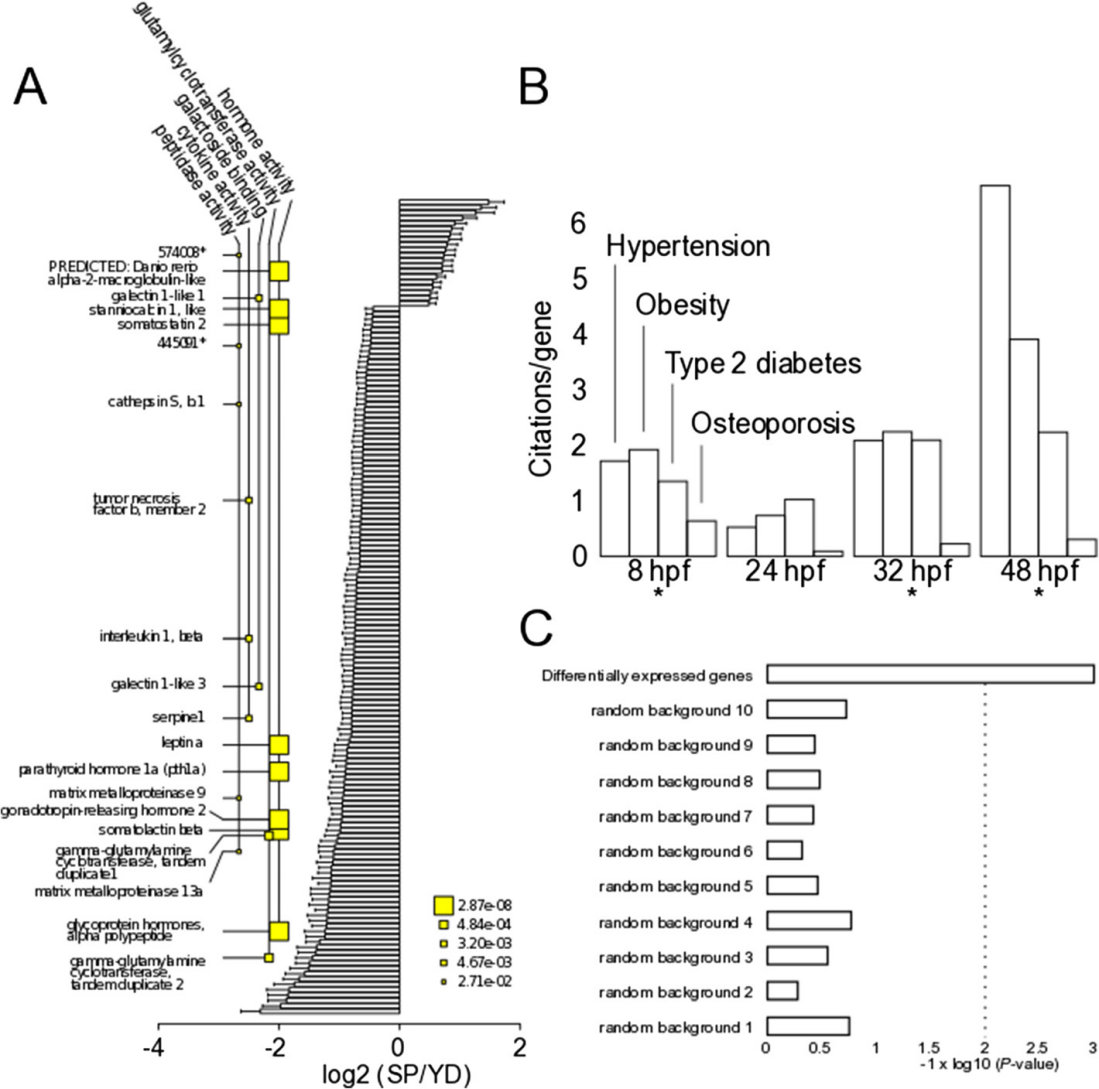
Molecular function gene ontology (mfGO) and co-citation enrichments. **a** Molecular function GO enrichment. From left to right: semantic transcript name, enriched mfGO terms (the size of the square relates to the *P*-values, see legend), barplot of log2 transformed DE of all significant transcripts. The position of the transcript names are aligned with the corresponding bars on the barplot, to indicate their rank among DE genes. Only transcript names linked to enriched mfGO terms are denoted. Error bars denote standard deviations. Asterisks show Entrez gene IDs for which no semantic name is available. **b** CoCiter counts for given terms and Entrez IDs from DE genes at all time points, divided by the number of included genes (citations per gene). Asterisks denote significant enrichment of combined terms (*P* < 0.005). **c** Enrichment analysis of combined terms from (**b**, 48 hpf) for significantly expressed genes in this study (top) and 10 collections of 153 randomly picked identifiers from the input (random background). The dashed line marks *P* = 0.01

At 48 hpf, hormone activity was the most highly ranked mfGO term (Fig. 3a and Additional file 7). Together with the enriched term “cytokine activity”, this strongly points out a systemic response at 48 hpf. One of these hormones, *i.e. Gonadotropin-releasing hormone 2* (g*nrh2*), was induced in the YD embryos (which was confirmed by qRT PCR, see Additional file 6). This hormone, which is conserved from fish to humans, but has been lost in rodents, negatively regulates feeding behavior in the zebrafish and other systems after intracerebroventricular injection [31]. Gnrh2 is an anorexic hormone, and potentially its up-regulation may reflect a reduced yolk consumption at 48 hpf YD embryos. Among genes with an mfGO term related to hormonal regulation, we furthermore found hormones involved in growth and metabolism, such as *somatostatin 2*, *stanniocalcin*, *parathyroid hormone 1a* (see Additional file 3), *glycoprotein hormones alpha polypeptide* and *somatolactin beta*.

### DE genes associate to the metabolic syndrome

Co-citation enrichments are useful to reveal associations between large gene sets and diseases. We applied the CoCiter tool [32] to look for associations between DE genes and “hypertension”, “obesity”, “type 2 diabetes” and “osteoporosis”, *i.e.* metabolic diseases of which the susceptibility is increased after early-life malnutrition in mammalian systems [4]. Required Entrez gene identifiers were obtained for 300/303 DE genes at 8 hpf, 113/113 DE genes at 24 hpf, 221/221 DE genes at 32 hpf and 132/153 DE genes at 48 hpf. Gene vs. term enrichments where found at 8 hpf (*P* < 0.005), 32 hpf (*P* < 0.005) and 48 hpf (*P* < 0.001), but not at 24 hpf (Fig. 3b and Additional files 8, 9, 10, 11). This outcome seems specific to DE genes: we repeated CoCiter analysis for 10 sets of 153 randomly sampled names in our 48 hpf dataset (at 48 hpf, the number of citations per gene is highest (Fig. 3b and Additional file 11)) and none of these samplings resulted in a significant outcome (Fig. 3c). Co-citation with metabolic diseases points out that the transcriptional response in YD embryos likely associates to the disease outcome of developmental nutrient restrictions.

### The methionine cycle is affected throughout development

The methionine cycle produces methyl donors for methylation of nucleic acids, as well as proteins. Upstream of this cycle, methyl groups enter the methionine cycle via folic acid, betaine and other metabolites [33]. Differential DNA methylation has been implicated in developmental outcomes of early-life nutrient restriction [34].

**Fig. 4:**
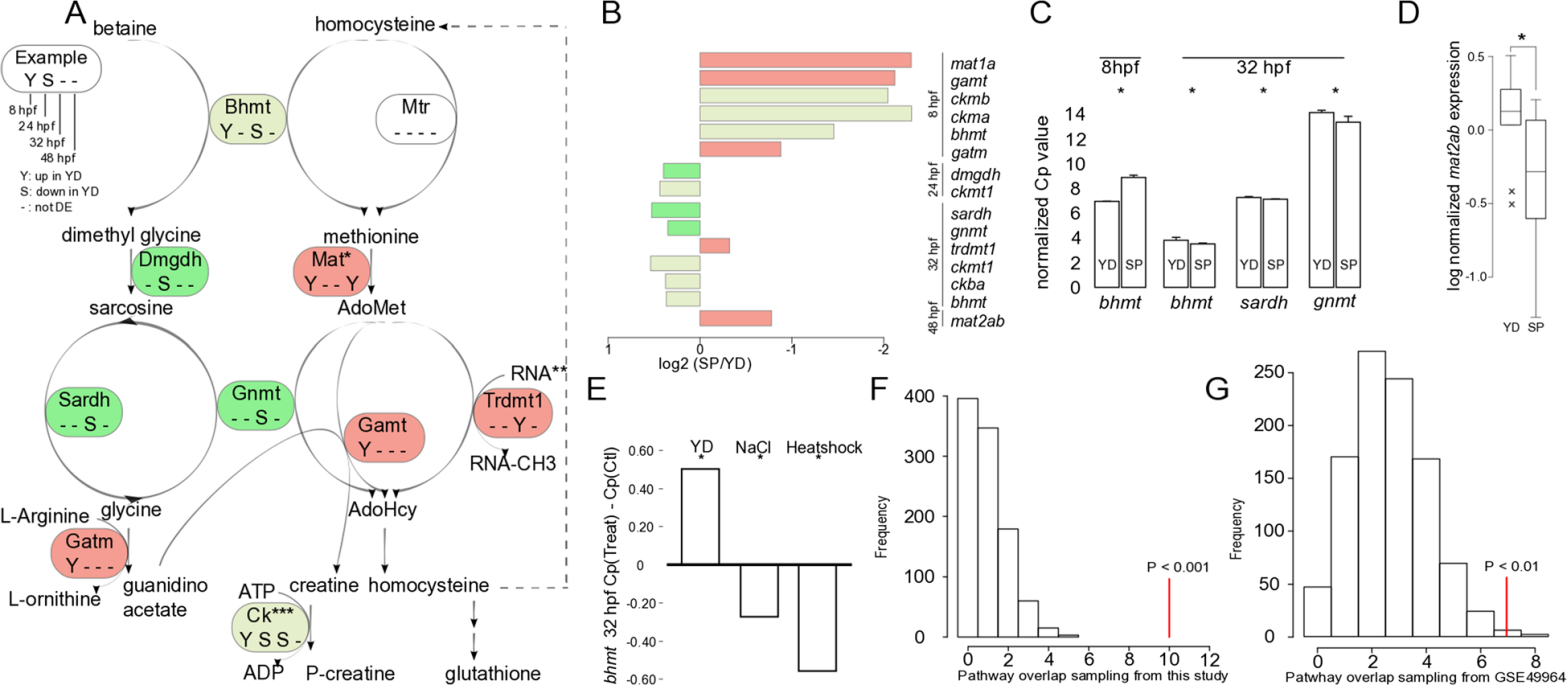
The methionine cycle is involved in the response to YD and its involvement is conserved. **a** Schematic representation of the methionine cycle. Gene symbols are followed by information about temporal DE. “Y” indicates up-regulation in YD; “S” indicates down-regulation in YD; a dash (−) indicates no DE. From left to right: 8, 24, 32, 48 hpf. See example in top left corner. Colors highlight whether genes are up-regulated in YD only (red), down-regulated in YD only (green) or up- and down regulated at different time-points (grey). *Two different DE *mat* genes are identified: *mat1a* at 8 hpf and *mat2ab* at 48 hpf. **trdmt1 is the only DE *dnmt* homologue in this study. Since it is a RNA methyl transferase, for clarity, only RNA is depicted as a substrate for methylation. ***Four DE creatine kinase genes are combined in this schematic figure. **b** DE levels of DE genes from (**a**), with identical color scheme. **c** DE of three genes confirmed by qRT PCR, RNA obtained from pooled embryos. **d** DE of *mat2ab* confirmed by qRT PCR, RNA obtained from individual embryos. **e** DE of *bhmt* in response to YD treatment (25 % yolk reduction), NaCl exposure and heatshock, data obtained from qRT PCR. Values shown are Cp (Treatment) – Cp(Control), normalized against Cp(*actb2).* **f** Methionine cycle genes that overlap with DE genes (red line) and identical-sized random samples from all genes in this study (open bars). **g** Seven out of 12 DE genes homologues that correspond to methionine cycle genes from this study overlap to DE genes in the liver of maternally starved fetal mice (red line). Open bars show the overlap with methionine cycle genes and 12 randomly sampled genes from the complete mouse liver gene set. Asterisks denote significant *P*-values according to the Student’s *T*-test in (**c**) and (**d**). In (**f**) and (**g**), the *P*-value represents the fraction of randomly sampled overlaps that is equal to or larger than the overlap with DE genes after 1000 permutations

In the methionine cycle, in total 10 enzymes (9 unique ones and at any time-point at least one) are DE (Fig. 4a and b):

- At 8 hpf, *bhmt*, *mat1a*, g*atm* and g*amt* are up-regulated in YD;
- at 24 hpf, *dmgdh* is down-regulated in YD;
- at 32 hpf, *trdmt1* is up-regulated in YD and *bhmt*, *sardh*, *gnmt* are down-regulated in YD; and
- at 48 hpf, *mat2ab* is up-regulated in YD.

Downstream enzymes, such as *creatine kinases*, are deregulated as well. We sampled 4 genes from this pathway (*i.e. bhmt, sardh, gnmt* and *mat2ab)* and validated their DE by qRT PCR (Fig. 4c and d).

To investigate whether the methionine cycle deregulation could be part of a general stress response, we measured expression levels of *bhmt* under two additional types of perturbation at 8.5 hpf by qRT PCR: 1) exposure to 10 mM NaCl and 2) exposure to 39.5C heatshock for 30 min. We harvested the embryos to measure gene expression at 32 hpf. At 32 hpf, after heatshock, survival dropped to 42 %, whereas in the other groups survival exceeded 95 %. *Bhmt* is significantly differentially expressed in all treatments. However, while this gene is down-regulated after YD treatment compared to SP, it is up-regulated after NaCl and heatshock treatments compared to non-exposed controls (Fig. 4e). *bhmt* is differentially expressed in two directions, both temporally after YD treatment and in response to different perturbations. We suggest that the dynamic response of the methionine cycle alters the availability of methyl donors, glutathione and creatine. This may affect methylation, oxidation and cellular energy availability, respectively, even though expression level differences are significant but small.

The overlap of DE genes with methionine cycle genes is significantly large (*P* < 0.001) (Fig. 4f). Our original set of DE genes overlaps with 10 genes in the methionine cycle, creatine kinases not considered. We replaced the original sets of DE genes with randomly sampled gene identifiers from all DE and non-DE genes and intersected these with methionine cycle enzymes. The maximum overlap was 5 genes after 1000 permutations. We included all known methyltransferases, and excluded creatine kinases (see methods).

The involvement of the methionine cycle may furthermore be evolutionary conserved. We observed an enrichment of DE methionine cycle genes in fetal livers of male C57BL/6 J mice at gestational day 18, of which the mothers had been exposed to 50 % food restriction (expression data obtained from GSE49964). Seven out of 12 homologues of DE genes from Fig. 4a and b are differentially expressed in these mouse livers, which is significantly more than randomly selected groups of 12 genes (*P* < 0.01, Fig. 4g).

Taken together, the genes in the methionine cycle dynamically respond to YD treatment and other types of perturbations. The involvement of the methionine cycle is likely conserved from fish to mammals. At present, we do not have direct evidence whether the state of the methionine cycle in YD leads to differential DNA methylation. We have not identified DNA methyltransferases among DE genes. The only DE methyl transferase in our data is *trdmt1*, which is an RNA methyltransferase. It methylates asp-tRNA [35] and has been linked to phenotypic, non-Mendelian inheritance, whereby it possibly affects mRNA methylation [36]. Possibly, the response to YD treatment involves differential methylation of selected genes (see next section), RNA, or other substrates.

### Potential avoidance of perturbations in methylation capacity

**Fig. 5:**
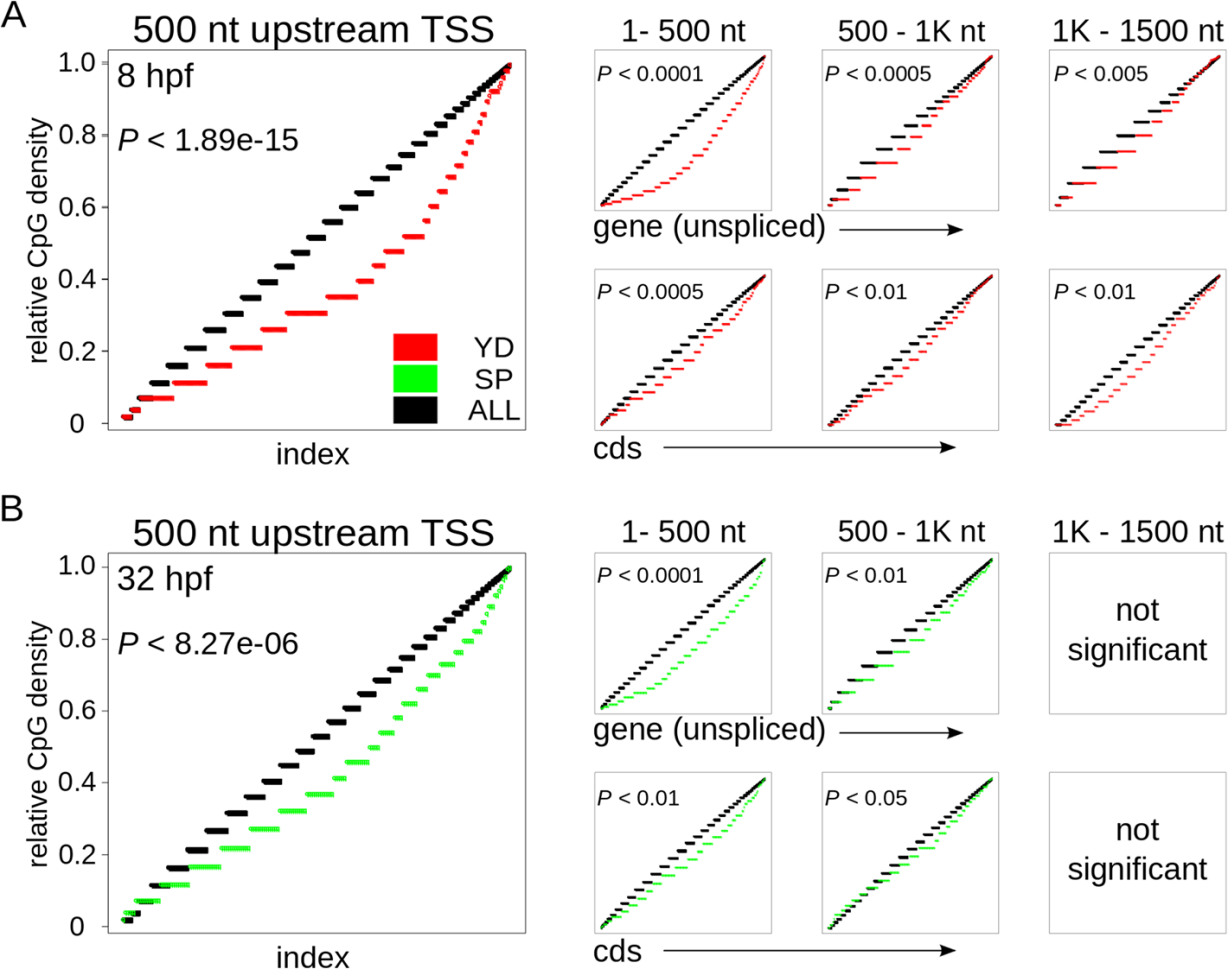
CpG density is reduced in genes that are deregulated. The empirical cumulative distribution (see methods) of the number of CpG sites in all genes (black) projected onto the number of CpG sites in DE genes. **a** Left panel: at 8 hpf, the Kolmogorov-Smirnov test reveals a significant reduction of CpG sites in promotor regions of DE genes that are up-regulated in YD (*P*-value in top left corner). Right panel: this depletion is most prominent around 5′ of the gene, including the proximal part of the cds. Five-hundred nt windows of the unspliced gene (top) and cds (bottom) are shown. **b** As in (**a**). At 32 hpf, a significant reduction of CpG sites in promotor regions and around the TSS is observed in DE genes that are down-regulated in YD. YD: up-regulated in YD; SP: down-regulated in YD; ALL: all available Ensembl-annotated genes

CpG sites around the transcription start site (TSS) are targets of DNA methyl transferases: the methylation of cytosine residues represses transcription [37]. In our dataset, we find sets of time-point specific deregulated genes with significantly reduced numbers of CpG sites around their TSS, compared to a genome-wide background: at 8 hpf, in YD embryos, genes that are up-regulated show a reduced CpG content (Fig. 5a). At 32 hpf, down-regulated genes in YD embryos show a reduced CpG content (Fig. 5b). A reduced CpG content in deregulated genes could indicate that CpG sites around the TSS of responsive genes are under negative selection and that these genes avoid transcriptional regulation by DNA methylation. Responsive genes may therefore be less sensitive for methylation perturbations and/or methylation may not be the dominant transcriptional regulator of these genes.

## Conclusions

YD treatment in zebrafish embryos dynamically affects the expression of metabolic genes, hormones and genes of the methionine cycle. Although expression differences between YD and SP embryos are subtle, the responding genes are part of a diverse set of functional pathways. Together, these pathways likely contribute to developmental plasticity after YD treatment.

Through the deregulated methionine cycle we find a possible role for methylation pathways in the response towards reduced nutrient availability. Functional approaches are required to obtain mechanistic insights in the temporally dynamic response of the methionine cycle and its downstream targets. We found the co-citation of metabolic traits and deregulated genes is significant. Also, the higher number of females after YD treatment may reflect a differential food consumption or growth rate between embryogenesis and early adulthood [18] which in humans could potentially increase the risk to develop metabolic disease [19]. Taken together there are strong similarities between the developmental outcome of YD treatment and the outcome of mammalian developmental nutrient restriction and FGR. In conclusion, our model revealed a dynamic transcriptional landscape after early-developmental nutrient reduction. This was identified by transcriptome profiling at various time-points. The zebrafish system we established here can help to elucidate the response to reduced nutrient availability during developmental stages and may serve as a model to study its physiological consequences.

## Methods

### Fish maintenance

Embryos were obtained from natural spawnings of an AB strain and raised at 28 °C. Paramecia (yolk yellow) were fed from 5 dpf to 14 days post-fertilization (dpf), twice daily. After 14 dpf, larvae were raised in 1.5 L tor 2.5 L tanks in a recirculating water system and fed with live brine shrimp, twice daily. Partially yolk-depleted (YD) and sham-punctured (SP) were placed in tanks at 14 dpf. Fish were fed by facility staff that was unaware of treatment information. Gender was determined by two independent observations of life fish in absence of treatment information. Prior to imaging adults, fish were anesthetized in MS222 according to [38]. Batches of embryos were divided to perform embryonic, larval and adult investigations. To perform experiments involving zebrafish in this study, approval was obtained from the Ethics Committee of Shanghai Institutes for Biological Sciences, Chinese Academy of Sciences (animal use protocol number: IBCB-ZF005).

### Embryo treatment

Embryos were kept in Hank’s embryo medium [38] with 0.01 mg/L methylene blue. A pulled glass syringe with an opening of 50 μm in diameter was mounted to a syringe system (Hamilton Bonaduz AG, Switzerland: #86257, 55750–01, 55752–01, 55753–01, PRMKIT), connected to a Pump 11 Elite (Harvard apparatus), programmed to withdraw 50 nl at 35 nl/min. Embryos were mounted on 1 % agarose-Hank’s grooves with the animal poles facing downwards. Yolk was removed from the vegetal pole. Sham treatment was performed by puncturing the embryo only. Pictures were analyzed using GIMP 2.6.8: for yolk removal estimates, the sphere volume of each embryo (at 7 to 8 hpf) was calculated in arbitrary units. Detailed images were created on a Zeiss Observer.Z1 microscope. Trizol (Ambion) was used for RNA collection.

### Expression sets

Expression profiles of pooled whole embryos were generated at 8, 24, 32 and 48 hpf. The first three profiles were generated by RNAseq from three batches of embryos in 3-fold, from which 32 %, 35 % and 35 % of yolk was removed. At 8, 24 and 32 hpf, respectively 15, 10 and 10 embryos were collected. Data was analyzed with EdgeR [39] (see below). At 48 hpf, we generated microarray expression profiles in 3-fold: for the first, second and third microarray experiment, respectively 36 %, 26 % and 26 % of yolk was removed and the number of pooled embryos was respectively 15 YD vs. 15 SP, 39 YD vs. 48 SP and 45 YD vs. 42 SP. We used the median expression difference between the three YD vs. SP comparisons and used these to estimate the *P*-value, adapted from an earlier described miRNA-seq method [40] (see below).

### Oil Red O (ORO) staining

This procedure was adapted from [41]. Embryos and larvae were fixed in PBS and 4 % formaldehyde for 4 h at RT. After 2 washes in PBS-Tween (0.1 %), embryos were stained with 0.3 % ORO in PBS- isopropanol (60 %) for 30 min at RT. Embryos were washed in PBST twice and stored overnight in glycerol at 4 °C.

### RNAseq and data analysis

Samples were prepared with Truseq RNA Sample prep kit v2 (Illumina), 50 bp sequence reads were obtained from the Illumina HiSeq 2000 platform (Illumina). Reads were mapped using Tophat [42] unique hits were collected using SAMtools [43]. Non-redundant bed files of exon sequences from the zebrafish zv9.71 assembly were intersected with reads using bedtools [44]. Genes were included in the down-stream analysis if the maximal readcount of that gene on the respective time-point exceeded 5. We subsequently compared data analysis by EdgeR and DESeq2 [45], which rendered a highly similar outcome (Additional file 12). We used EdgeR used batches as a blocking variable, as suggested by this tool’s guidelines. *P*-values were corrected [46] and differentially expressed (DE) genes were selected if FDR < 0.05. We only kept differentially expressed (DE) genes with consistent up- or down regulation in YD over three biological replicates. Data has been deposited under ArrayExpress accession E-MTAB-2194.

### Microarray experimental procedures and analysis

The Shanghai Biotechnology Corporation hybridized samples to zebrafish (V3) 4x44K microarrays (Agilent). We analyzed the log-transformed normalized data from Feature extraction 10.7 (Agilent) with the R package [47–49]. We collected transcripts with Ensembl, Genbank, Entrez or Refseq identifiers and selected probes with a reliable signal-to-noise ratio in more than 2/3 of the arrays (noted by a “P-flag” in the Feature extraction output). We quantile-normalized the data and calculated the average intensity of probes covering identical transcripts. We calculated the median intensity of each transcript over the 3 YD arrays and the 3 SP arrays and took their average (the transcript intensity score). Next, we calculated the median difference of every YD vs. SP pair (transcript differential expression score). We sorted the transcript intensity scores along transcript differential expression scores. The Z-score was calculated by comparing each transcript differential expression score to the mean and the standard deviation of a window of 65 transcripts surrounding this value. *P*-values were obtained, which were subsequently corrected [46]. Transcripts were collected with a false-discovery rate (FDR) below 0.1. Data has been deposited under ArrayExpress accession E-MTAB-1605.

### GO enrichment analysis

We identified molecular function (mf) GO terms that were represented at least twice. We performed hypergeometric testing by using the R package [48]. Significant GO terms were collected with a FDR < 0.05. To validate our method, we re-analyzed the 48 hpf dataset with the publicly available tool Webgestalt [50], which confirmed enrichment of 4 out of the 5 observed enriched mfGO terms (Additional file 13).

### Random sampling, empirical distribution

Differential expression from the mouse liver expression dataset GSE49964 was obtained with the limma package [49]. We counted the overlap of DE gene symbols in this mouse dataset with gene symbols *Trdmt1*, *Bhmt*, *Dmgdh*, *Sardh*, *Gnmt*, *Ckb*, *Ckmt1*, *Mat1a, Mat2a*, *Ckm*, *Gatm*, *Gamt* and 12 randomly sampled gene symbols*.* For the enrichment analysis of methionine cycle genes within the zebrafish expression profiles, we counted the overlap of randomly obtained gene symbols and Ensembl IDs from all genes with *bhmt* (ENSDARG00000013430), *dmgd* (ENSDARG00000025703), *sardh* (ENSDARG00000058102), *gnmt* (ENSDARG00000006840), *gamt* (ENSDARG00000070844), *gatm* (ENSDARG00000036239), *mtr* (ENSDARG00000039134), *mat1a* (ENSDARG00000039605), *mat2aa* (ENSDARG00000040334), *mat2al* (ENSDARG00000063665), *mat2ab* (ENSDARG00000037121), *mat2b* (ENSDARG00000012932), *dnmt1* (ENSDARG00000030756), *dnmt3ab* (ENSDARG00000015566), *dnmt3* (ENSDARG00000057830), *dnmt3aa* (ENSDARG00000005394), *dnmt3b* (ENSDARG00000052402), *dnmt4* (ENSDARG00000036791), *dnmt5* (ENSDARG00000057863), *trdmt1* (ENSDARG00000034518). For CpG counts, sequences were downloaded from www.ensembl.org/biomart. CpG sequences were counted and the distributions of all included Ensembl gene identities for each time-point was calculated using the ecdf function in R. The CpG counts of up- and downregulated genes were aligned with the empirical distributions. The Kolmogorov-Smirnov test was performed on the CpG counts using the ks.test function in R.

### qRT PCR

For qRT PCR validation of RNAseq, we pooled the original YD or SP total RNA used for RNAseq from each time-point in equimolar amounts and used 150 ng in the reverse-transcription (RT) reaction. To validate the microarray data method of analysis, we collected and pooled 19 2 dpf YD and 19 2 dpf SP embryos, for which we used 1 μg per sample in the RT reaction. For the *mat2ab* verification, we collected total RNA from YD and SP embryos (10 individuals per group). We used 40 ng from each embryo in RT reactions. To address the different types of perturbations (*i.e.* YD treatment, NaCl exposure and heatshock), we used 210 ng RNA in the RT reaction. For RT, we used Superscript II (Life technologies) according to the manufacturer’s guidelines. We diluted the RT product (microarray validation: 25 to 250x, *mat2ab*: 70x) and used commercial master mixes for quantitative PCR in 5–10 μl reaction volumes (Lightcycler 480 DNA SYBR Green I Master (Roche) or Sybr select Master mix (Life technologies)). See Additional file 14 for primer sequences and genomic coordinates. Two *actb2* primer pairs were used: the beta-act_FR2 was only used for *mat2ab* validation assay. PCR conditions were optimized for each primer pair in order to obtain single amplicons of the correct size. Samples were amplified as follows: 95C (5 or 10 min); 45 x [95C 10s; 56 or 58C (10 or15s); 72C (15 or 20s)]; 65C to 95 or 97C melting curve, amplified and analyzed on a Lightcycler 480 system (Roche).

To analyze the data, we used the ΔCp value that provided by the Lightcycler 480 system analysis software. We calculated ΔCp(gene of interest) – mean ΔCp(*actb2*) and calculated the *P*-value according to the Student’s *T*-test. To analyze the *mat2ab* data, the concentration was measured according to a serial dilution of one RT sample. We performed a Student’s *T*-test to obtain the corresponding *P*-value.

## Availability of supporting data

The data sets supporting the results of this article are available in the ArrayExpress repository, E-MTAB-1605 and E-MTAB-2194.

## Additional files

Additional file 1:
**Impression of YD and SP embryos at 4 time-points.** A) Mean sphere volume of embryos after YD and SP treatment. In this particular batch, on average 31 % of yolk was removed from YD embryos. Error bars denote standard deviations. B) Images of the total batch (8 hpf), and samples within this batch at 23, 31 and 48 hpf. Additional file 2:
**Tabulized differentially expressed genes across 4 time-points.** Only genes with Ensembl identifiers are included. Not all genes at 48 hpf have an Ensembl identifier: for a complete list for DE genes at 48 hpf, see Additional file 3.xls. Differential expression is denoted as the log2-fold expression difference (SP/YD). Column 1: wiki description (if available); column 2: Ensembl identifier; column 3–6: DE for each time-point, 0 if not significantly differentially expressed. Additional file 3:
**Differentially expressed genes at 48 hpf.** Column 1: database system; column 2: identifiers (either Ensembl, Genbank, Entrez or Refseq); column 3: median log2-fold expression difference (SP/YD); column 4: gene description in the normalized datafile (if available in the feature-extraction output). Additional file 4:
**Tabulized all included genes across 4 time-points.** Only genes with Ensembl identifiers are included. Not all genes at 48 hpf have an Ensembl identifier: for a complete list for all included genes at 48 hpf, see Additional file 5.txt. Column 1: Ensembl identifier; column 2–5: log2-fold expression difference for each time-point, 0 if not included in this time-point. Additional file 5:
**All included genes at 48 hpf.** Column 1: database system; column 2: identifiers (either Ensembl, Genbank, Entrez, Refseq, or other if not known); column 3: median log2-fold expression difference (SP/YD). Additional file 6:
**qRT PCR confirmation of a selection of DE genes.** A,B) Asterisks denote significant *P*-value (*P* < 0.05). C) Correlation plot of DE values obtained by RNAseq (black) and microarray (red) vs. DE values obtained from qPCR data (ΔΔCp). This graph shows log2-transformed data only: RNAseq and microarray data are log2 transformed. The ΔCp value approaches a log2-fold representation of expression levels. Up and down-regulation according to transcriptome profiles is consistent with qRT PCR outcome for all indicated genes. D) Correlation plot of all samples. Y8_1 is a YD sample, 8hpf, batch 1. Microarray batches have been denoted with A-C instead of 1–3, in order to distinguish RNAseq samples from microarray samples. Axes denote log2 expression values; red-colored numbers show spearman correlation coefficients. Additional file 7:
**Ranked GO enrichments.** Molecular function GO identifiers that are enriched (FDR < 0.05) in at least one time-point. The rank shows the identifiers from the highest to lowest significance, based on the product of corrected *P*-values over 8, 24, 32 and 48 hpf. Additional file 8:
**CoCiter output of differentially expressed genes at 8 hpf.** RNAseq outcome associated to the terms “hypertension”, “obesity”, “type 2 diabetes” and “osteoporosis”. Additional file 9:
**CoCiter output of differentially expressed genes at 24 hpf.** RNAseq outcome associated to the terms “hypertension”, “obesity”, “type 2 diabetes” and “osteoporosis”. Additional file 10:
**CoCiter output of differentially expressed genes at 32 hpf.** RNAseq outcome associated to the terms “hypertension”, “obesity”, “type 2 diabetes” and “osteoporosis”. Additional file 11:
**CoCiter output of differentially expressed genes at 48 hpf.** Microarray outcome associated to the terms “hypertension”, “obesity”, “type 2 diabetes” and “osteoporosis”. Additional file 12:
**Overlap between outcomes of EdgeR and DESeq2 is statistically significant.** For any given RNAseq datatset, the overlap between EdgeR and DESeq2 leads to a significant outcome, as denoted by the *P*-value of the hypergeometric test (Phyper). In the DESeq2 analysis, we compared YD vs SP triplicates at each time-point separately, using standard settings, using a multifactorial design (design = ~ treatment + batch). Phyper was calculated with the overlap between datasets, and the number of included genes (*i.e.* the sample pool) was adopted from de EdgeR dataset (numbers in Fig. 1F). EdgeR analysis was performed as described in the methods. YD: up-regulated in YD; SP: down-regulated in YD. Additional file 13:
**Webgestalt output of Gene-ontology enrichment.** Biological process, molecular function and cellular component-enriched terms are shown. *P*-values are adjusted according to [46]. Additional file 14:
**Primers used for qRT PCR.** Genome positions and orientation according to Zv9, Apr 2010 (www.ensembl.org). Splice site (Y) indicates that the primer overlaps with an exon-exon boundary, (N) if primer overlaps one exon only. Gonadotropin releasing hormone 2 was primed with a transcript-specific primer (gnrh2rt_tsp), all other transcripts were primed with the anchored (3′ V, V = A, C or G) oligo(dT) primer.

## Abbreviations

AB strain: Commonly used wild-type zebrafish strains
*cebpb*: CCAAT/enhancer binding protein (C/EBP) beta
CpG: Site cytosine linked on its 3′ to a guanine, linked by a phosphodiester bond
DE: Significant differential expression
DE genes: Significantly differentially expressed genes in the transcriptome profiles
DE and non-DE genes: Per time-point, the complete set of genes of that passed expression level thresholds, serves as background set.
FDR: False-discovery rate
FGR: Fetal growth restriction
ORO: Oil Red O for lipid staining
RNAseq: Transcriptome profiling by sequencing poly-adenylated, fragmented, reverse-transcribed and amplified RNA samples
RT: Reverse-transcription
SGA: Small-for-gestational age
SP: Sham-punctured control
TSS: Transcription start site
YD: Partially yolk-depleted
YSL: Yolk-syncytial layer
*abca1b*: ATP-binding cassette transporter ABCA1, b
*actb2*: Actin, beta 2
*apo*: Apolipoprotein
*bhmt*: Betaine-homocysteine S-methyltransferase
*ckb*: Creatine kinase, brain
*ckm*: Creatine kinase, muscle
*ckmt*: Creatine kinase, mitochondrial
ΔCp: The Lightcycler 480 system (Roche) provides expression values as Cp values
Cp values of genes of interest where subtracted from Cp values of *actb2*. The lower the Cp: the higher the expression
*dmgdh*: Dimethylglycine dehydrogenase
*dnmt*: DNA methyltransferase
dpf: Days post-fertilization
*egln2*: Egl-9 family hypoxia-inducible factor 2
*egln3*: Egl-9 family hypoxia-inducible factor 3
*gamt*: Guanidinoacetate N-methyltransferase
*gatm*: Glycine amidinotransferase (L-arginine:glycine amidinotransferase)
*gnmt*: Glycine N-methyltransferase
*gnrh2*: Gonadotropin-releasing hormone 2
Hifs: Hypoxia-inducible factors
hpf: Hours post-fertilization
*mat1a*: Methionine adenosyltransferase I, alpha
*mat2ab*: Methionine adenosyltransferase a
mfGO: Molecular function Gene ontology term
*mtp*: Microsomal triglyceride transfer protein
*mtr*: 5-methyltetrahydrofolate-homocysteine methyltransferase
*pfkfb4l*: 6-phosphofructo-2-kinase/fructose-2,6-biphosphatase 4, like
*phd3*: Prolyl hydroxylase domain-containing protein 3. Alias for *egln3*
qRT PCR: Quantitative real-time PCR
*sardh*: Sarcosine dehydrogenase
*trdmt1*: tRNA aspartic acid methyltransferase 1
*txnipa*: Thioredoxin interacting protein, a
*txnipb*: Thioredoxin interacting protein, b
