# Supplementary figures and images for: Partial depletion of yolk during zebrafish embryogenesis changes the dynamics of methionine cycle and metabolic genes
Source: BMC Genomics. 2015 Jun 4;16(1):427. doi: 10.1186/s12864-015-1654-6 (PMC4455928; doi:10.1186/s12864-015-1654-6)

A

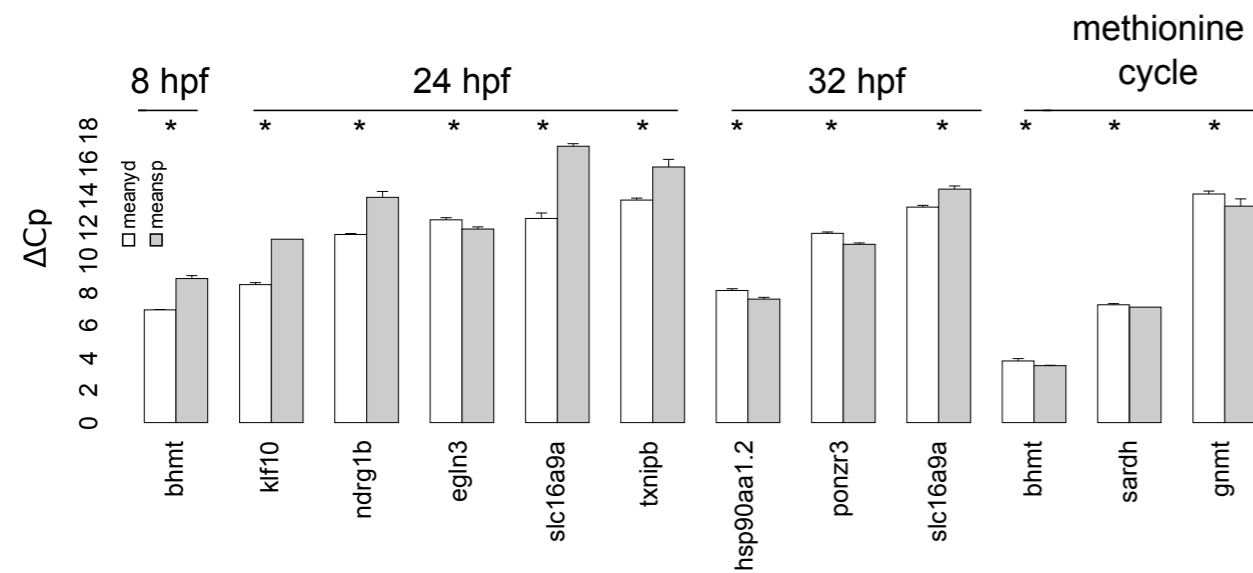

B

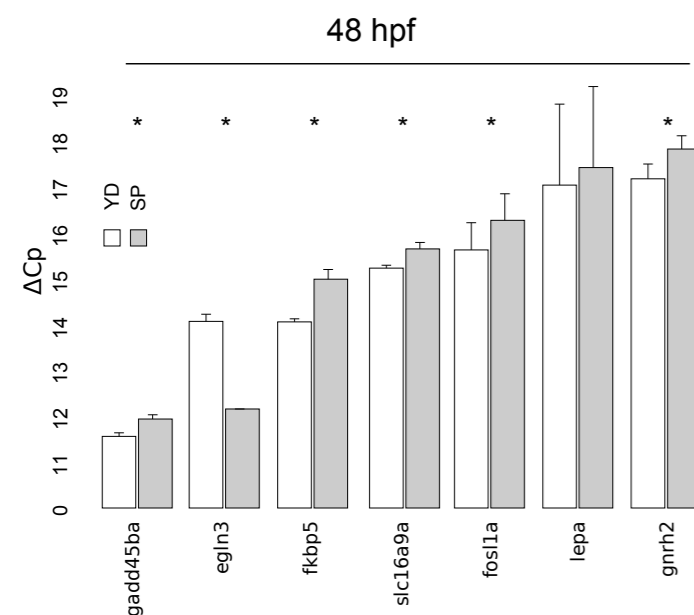

C

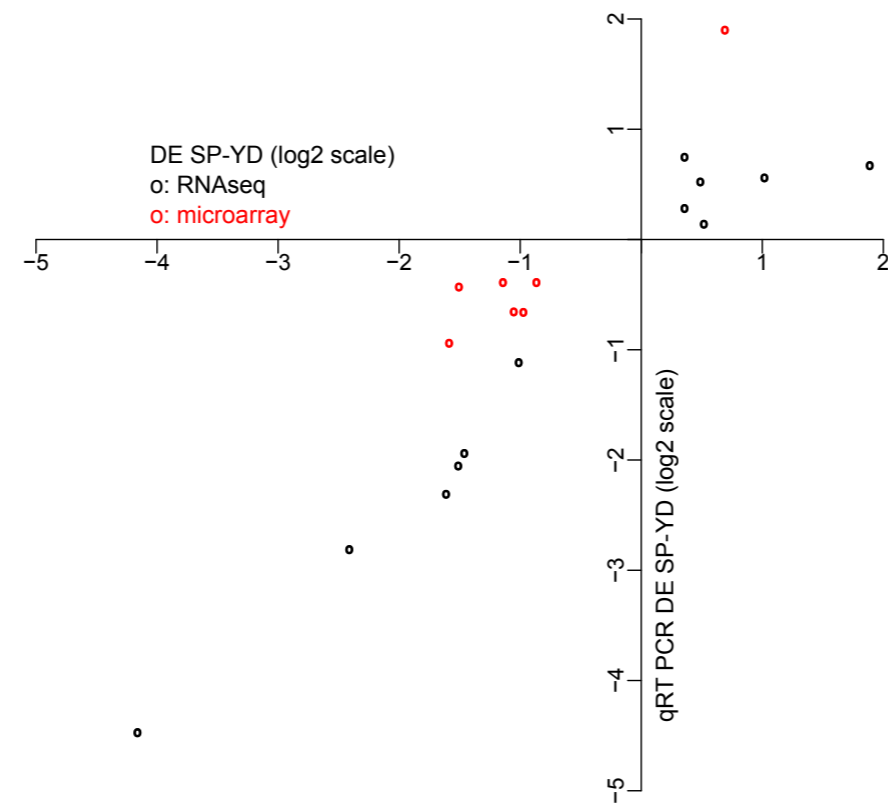

D

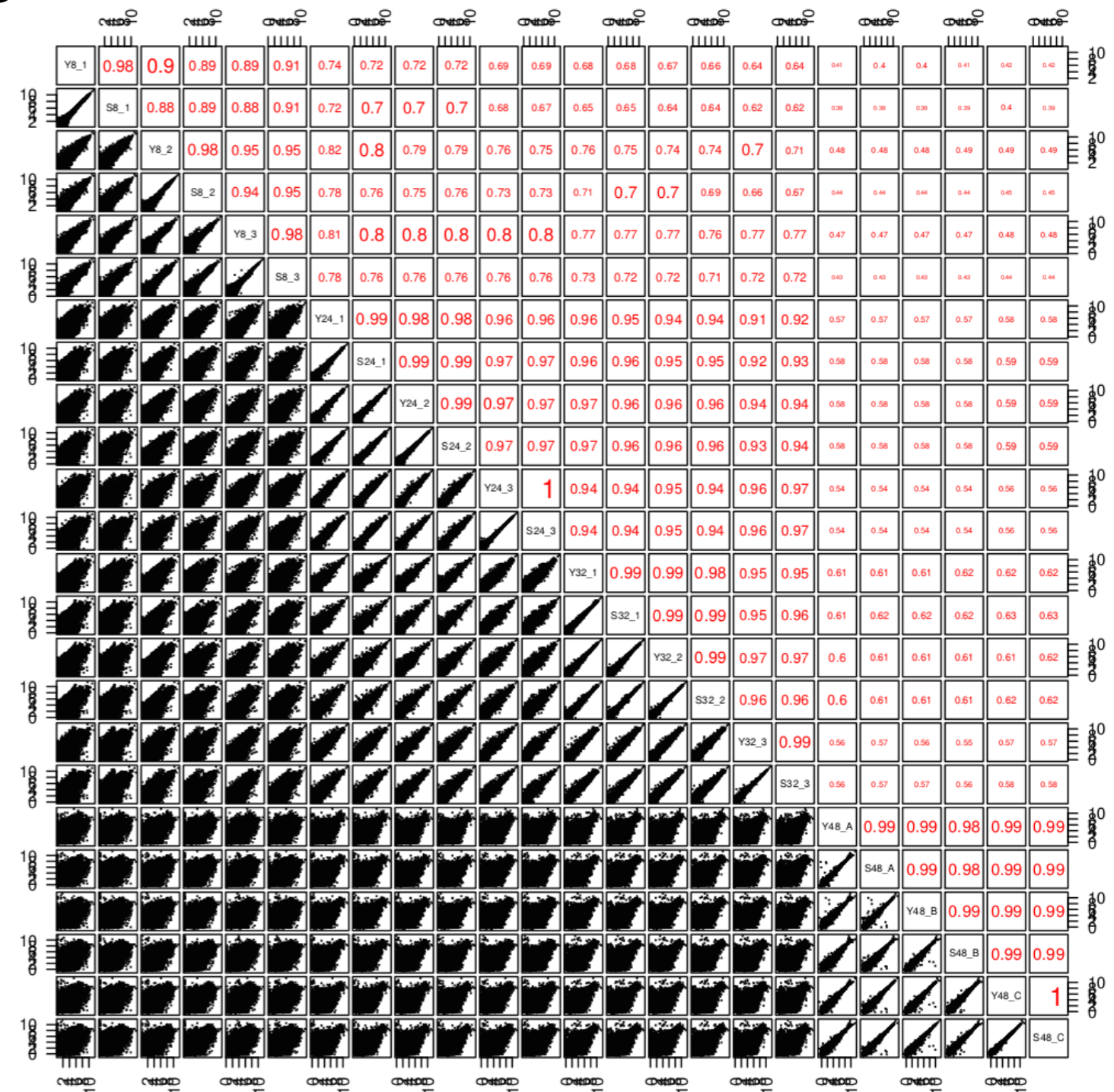

Supplement: Additional file 6: — qRT PCR confirmation of a selection of DE genes. A,B) Asterisks denote significant P-value (P < 0.05). C) Correlation plot of DE values obtained by RNAseq (black) and microarray (red) vs. DE values obtained from qPCR data (ΔΔCp). This graph shows log2-transformed data only: RNAseq and microarray data are log2 transformed. The ΔCp value approaches a log2-fold representation of expression levels. Up and down-regulation according to transcriptome profiles is consistent with qRT PCR outcome for all indicated genes. D) Correlation plot of all samples. Y8_1 is a YD sample, 8hpf, batch 1. Microarray batches have been denoted with A-C instead of 1–3, in order to distinguish RNAseq samples from microarray samples. Axes denote log2 expression values; red-colored numbers show spearman correlation coefficients. [file 12864_2015_1654_MOESM6_ESM.pdf]
