# Supplementary material for: Partial depletion of yolk during zebrafish embryogenesis changes the dynamics of methionine cycle and metabolic genes
Source: BMC Genomics. 2015 Jun 4;16(1):427. doi: 10.1186/s12864-015-1654-6 (PMC4455928; doi:10.1186/s12864-015-1654-6)
Supplement: Additional file 7: — Ranked GO enrichments. Molecular function GO identifiers that are enriched (FDR < 0.05) in at least one time-point. The rank shows the identifiers from the highest to lowest significance, based on the product of corrected P-values over 8, 24, 32 and 48 hpf. [file 12864_2015_1654_MOESM7_ESM.pdf]

rank	mfGo term	semantic name	FDR 8 hpf	FDR 24hpf	FDR 32hpf	FDR 48hpf	FDR product
1	GO:0019825	oxygen binding	2.5e-11	1.0e+00	3.9e-03	1.0e+00	1.0e-13
2	GO:0005344	oxygen transporter activity	2.5e-11	1.0e+00	3.9e-03	1.0e+00	1.0e-13
3	GO:0005509	calcium ion binding	8.1e-13	1.0e+00	1.0e+00	1.0e+00	8.1e-13
4	GO:0005319	lipid transporter activity	1.2e-07	1.0e+00	1.3e-02	1.0e+00	1.5e-09
5	GO:0016491	oxidoreductase activity	1.0e+00	1.3e-07	1.3e-02	1.0e+00	1.8e-09
6	GO:0005201	extracellular matrix structural constituent	1.2e-08	1.0e+00	1.0e+00	1.0e+00	1.2e-08
7	GO:0005506	iron ion binding	5.6e-04	1.9e-02	2.0e-03	1.0e+00	2.1e-08
8	GO:0005179	hormone activity	1.0e+00	1.0e+00	1.0e+00	2.9e-08	2.9e-08
9	GO:0003824	catalytic activity	1.0e+00	1.3e-02	1.3e-04	1.0e+00	1.8e-06
10	GO:0003779	actin binding	3.8e-06	1.0e+00	1.0e+00	1.0e+00	3.8e-06
11	GO:0030674	protein binding, bridging	4.3e-04	1.0e+00	1.3e-02	1.0e+00	5.6e-06
12	GO:0020037	heme binding	3.6e-04	1.0e+00	1.6e-02	1.0e+00	5.9e-06
13	GO:0008233	peptidase activity	1.0e+00	1.0e+00	4.9e-04	2.7e-02	1.3e-05
14	GO:0008289	lipid binding	2.9e-05	1.0e+00	1.0e+00	1.0e+00	2.9e-05
15	GO:0005198	structural molecule activity	2.6e-03	4.2e-02	1.0e+00	1.0e+00	1.1e-04
16	GO:0003839	gamma-glutamylcyclotransferase activity	1.0e+00	1.0e+00	1.0e+00	4.8e-04	4.8e-04
17	GO:0036094	small molecule binding	2.8e-02	1.0e+00	1.9e-02	1.0e+00	5.4e-04
18	GO:0005102	receptor binding	5.6e-04	1.0e+00	1.0e+00	1.0e+00	5.6e-04
19	GO:0005215	transporter activity	5.6e-04	1.0e+00	1.0e+00	1.0e+00	5.6e-04
20	GO:0004866	endopeptidase inhibitor activity	1.0e+00	1.0e+00	1.7e-03	1.0e+00	1.7e-03
21	GO:0017076	purine nucleotide binding	1.0e+00	1.0e+00	2.0e-03	1.0e+00	2.0e-03
22	GO:0004611	phosphoenolpyruvate carboxykinase activity	1.0e+00	1.0e+00	2.0e-03	1.0e+00	2.0e-03
23	GO:0004613	phosphoenolpyruvate carboxykinase (GTP) activity	1.0e+00	1.0e+00	2.0e-03	1.0e+00	2.0e-03
24	GO:0004563	beta-N-acetylhexosaminidase activity	1.0e+00	1.0e+00	2.0e-03	1.0e+00	2.0e-03
25	GO:0004252	serine-type endopeptidase activity	1.0e+00	1.0e+00	2.0e-03	1.0e+00	2.0e-03
26	GO:0004869	cysteine-type endopeptidase inhibitor activity	1.0e+00	1.0e+00	3.1e-03	1.0e+00	3.1e-03
27	GO:0016936	galactoside binding	1.0e+00	1.0e+00	1.0e+00	3.2e-03	3.2e-03
28	GO:0010181	FMN binding	1.0e+00	1.0e+00	3.8e-03	1.0e+00	3.8e-03
29	GO:0008158	hedgehog receptor activity	1.0e+00	1.0e+00	3.9e-03	1.0e+00	3.9e-03
30	GO:0004367	glycerol-3-phosphate dehydrogenase [NAD+] activity	1.0e+00	4.0e-03	1.0e+00	1.0e+00	4.0e-03
31	GO:0005125	cytokine activity	1.0e+00	1.0e+00	1.0e+00	4.7e-03	4.7e-03
32	GO:0043169	cation binding	1.0e+00	1.0e+00	4.7e-03	1.0e+00	4.7e-03
33	GO:0008236	serine-type peptidase activity	1.0e+00	1.0e+00	4.7e-03	1.0e+00	4.7e-03
34	GO:0003774	motor activity	8.5e-03	1.0e+00	1.0e+00	1.0e+00	8.5e-03
35	GO:0004553	hydrolase activity, hydrolyzing O-glycosyl compounds	1.0e+00	1.0e+00	1.3e-02	1.0e+00	1.3e-02
36	GO:0030247	polysaccharide binding	1.0e+00	1.0e+00	1.3e-02	1.0e+00	1.3e-02
37	GO:0016616	oxidoreductase activity, acting on the CH-OH group of donors, NAD or NADP as acceptor	1.0e+00	1.3e-02	1.0e+00	1.0e+00	1.3e-02
38	GO:0016614	oxidoreductase activity, acting on CH-OH group of donors	1.0e+00	1.3e-02	1.0e+00	1.0e+00	1.3e-02
39	GO:0016787	hydrolase activity	1.0e+00	1.0e+00	1.3e-02	1.0e+00	1.3e-02
40	GO:0008509	anion transmembrane transporter activity	1.4e-02	1.0e+00	1.0e+00	1.0e+00	1.4e-02
41	GO:0005452	inorganic anion exchanger activity	1.4e-02	1.0e+00	1.0e+00	1.0e+00	1.4e-02
42	GO:0004859	phospholipase inhibitor activity	1.4e-02	1.0e+00	1.0e+00	1.0e+00	1.4e-02
43	GO:0003735	structural constituent of ribosome	1.4e-02	1.0e+00	1.0e+00	1.0e+00	1.4e-02
44	GO:0005544	calcium-dependent phospholipid binding	1.9e-02	1.0e+00	1.0e+00	1.0e+00	1.9e-02
45	GO:0031418	L-ascorbic acid binding	1.0e+00	2.2e-02	1.0e+00	1.0e+00	2.2e-02
46	GO:0016705	oxidoreductase activity, acting on paired donors, with incorporation or reduction of molecular oxygen	1.0e+00	2.3e-02	1.0e+00	1.0e+00	2.3e-02
47	GO:0046872	metal ion binding	1.0e+00	2.6e-02	1.0e+00	1.0e+00	2.6e-02
48	GO:0050662	coenzyme binding	1.0e+00	2.6e-02	1.0e+00	1.0e+00	2.6e-02
49	GO:0042803	protein homodimerization activity	1.0e+00	2.8e-02	1.0e+00	1.0e+00	2.8e-02
50	GO:0016702	oxidoreductase activity, acting on single donors with incorporation of molecular oxygen, incorporation of two atoms of oxygen	1.0e+00	2.8e-02	1.0e+00	1.0e+00	2.8e-02
51	GO:0016706	oxidoreductase activity, acting on paired donors, with incorporation or reduction of molecular oxygen, 2-oxoglutarate as one donor, and incorporation of one atom each of oxygen into both donors	1.0e+00	3.3e-02	1.0e+00	1.0e+00	3.3e-02
52	GO:0008234	cysteine-type peptidase activity	1.0e+00	1.0e+00	3.3e-02	1.0e+00	3.3e-02
53	GO:0051287	NAD binding	1.0e+00	3.7e-02	1.0e+00	1.0e+00	3.7e-02
54	GO:0008484	sulfuric ester hydrolase activity	1.0e+00	1.0e+00	4.0e-02	1.0e+00	4.0e-02
55	GO:0046961	proton-transporting ATPase activity, rotational mechanism	1.0e+00	1.0e+00	4.6e-02	1.0e+00	4.6e-02
56	GO:0005540	hyaluronic acid binding	4.8e-02	1.0e+00	1.0e+00	1.0e+00	4.8e-02
57	GO:0004867	serine-type endopeptidase inhibitor activity	4.8e-02	1.0e+00	1.0e+00	1.0e+00	4.8e-02
58	GO:0030170	pyridoxal phosphate binding	1.0e+00	4.9e-02	1.0e+00	1.0e+00	4.9e-02
