# Supplementary material for: Partial depletion of yolk during zebrafish embryogenesis changes the dynamics of methionine cycle and metabolic genes
Source: BMC Genomics. 2015 Jun 4;16(1):427. doi: 10.1186/s12864-015-1654-6 (PMC4455928; doi:10.1186/s12864-015-1654-6)
Supplement: Additional file 8: — CoCiter output of differentially expressed genes at 8 hpf. RNAseq outcome associated to the terms “hypertension”, “obesity”, “type 2 diabetes” and “osteoporosis”. [file 12864_2015_1654_MOESM8_ESM.html]

CoCiter v1.1


**CoCiter v1.1**

## Co-citation analysis on genes/terms

Home
Gene-Gene
Gene-Term
Term-Term
ViewJob
HanLab

## Your search results:

**Genes/Terms involved in mistake below are not calculated in this result.**
Genes 100535293, 565644, 100537468 in the Gene Set are not included in the database of that species!

| Set | Genes/Terms | PubMed Count | CI | Significance |
| --- | --- | --- | --- | --- |
| GeneSet 288 genes | apoea (553587), rbp4 (30077), col1a1a (337158), fabp2 (30708), f2 (325881), apoa1a (30355), cpe (407979), c3a (321046), cav1 (323695), gstt1a (563972), fgb (337315), aldh2.2 (368239), akr1b1 (415138), LOC100333821 (100333821), mtp (406207), apoa4 (322543), zgc:194131 (570354), sparc (321357), serpina1 (322701), serpina1l (321195), col1a2 (336471), thbs1 (561901), tfa (30255), fga (378986), g6pca.2 (563180), nhlh2 (402968), gch2 (64263), plg (322691), cav3 (449679), ptk2bb (245950), ptk2.1 (142986), icn2 (100005083), bhmt (322228), icn (336655), tgm2b (323856), nr2f2 (30424), slc7a8a (100007704), smyhc1 (321552), dcn (64698), fgg (406327), mdka (30277), anxa1a (334724), actc1b (58114), mdkb (65231), postnb (337176), tgfbi (321421), fstl1a (325423), agpat2 (777624), gpib (246095), tmem38a (393874), pygma (553655), ctsc (368704), ca2 (387526), cxcl14 (58151), tyrp1b (437022), slc4a4a (568631), col5a1 (100334431), ttna (317731), trpm5 (557722), actn3a (58037), ak1 (445486), lum (415149), col4a5 (323561), slit3 (80354), anxa2a (325557), zgc:77517 (393540), gamt (796865), mat1a (334195), actn1 (560400), pls3 (436598), ckmb (794752), hinfp (406733), pabpc4 (321035), rps3a (337240), col9a3 (567110), tnnt3a (58084), mylpfa (30429), tnnc2 (58082), papd4 (553626), myl1 (336165), ambp (394093), slc39a1 (321324), ces2 (566132), h3f3a (406269), runx1t1 (767809), col5a2a (564821), matn4 (497348), acta1a (550445), gatm (266799), ckma (30095), tnnt3b (100003572), eef2k (437013), cfl2 (403001), LOC557250 (557250), elavl3 (30732), hbae1 (30597), meis1 (170446), rplp2 (335629), rps15 (337148), LOC100536875 (100536875), mgst1.2 (431762), myhz1.1 (58142), tnni2b.1 (550249), rac3b (795167), myoz1b (436720), LOC553326 (553326), atp2a1l (494489), atp2a1 (260440), zgc:92868 (445040), h2afy2 (558711), tuba1l (573122), cryba2b (436857), agr2 (335616), acta1b (407658), myhz2 (246275), lin9 (560867), dpysl3 (553166), lsm8 (566017), hoxb9a (30344), ivns1abpa (387591), mylpfb (447930), det1 (324089), si:dkey-222f2.1 (568321), zgc:92533 (445051), cx30.3 (404725), LOC100334599 (100334599), rps9 (114420), pgam2 (572733), h1f0 (321618), rps7 (393725), pvalb9 (360209), fkbp9 (445126), si:ch211-5k11.2 (572729), cryba2a (415139), klhl31 (407079), cyt1l (792062), si:dkeyp-113d7.4 (553371), aldoab (406496), mybpc2b (541384), nr2f1a (30418), urah (751742), tnni2a.4 (494164), crybb1 (114418), s100a10b (406276), ns:zf-e68 (100008070), pou3f2b (30397), rpl11 (415229), myhz1.2 (799300), fam219aa (445197), ldb3b (334100), hbbe3 (30596), tnnt2d (574000), uba52 (641289), si:busm1-118j2.5 (335781), wu:fd11e11 (386799), MGC174857 (563390), ptmaa (335707), wu:fc13c02 (561837), he1b (407971), mylz3 (58143), hoxb3a (30339), pcdh1g32 (554016), pcdh1g31 (554015), pcdh1g30 (554014), h3f3d (394076), pvalb2 (58028), hkdc1 (321224), and1 (794315), MGC174152 (564835), lyz (677744), ank2b (450043), hbbe1.1 (81538), gpm6aa (368223), tnnc1b (415175), zgc:56676 (393273), napg (436742), slc4a1a (84703), tnw (30234), si:ch73-21g5.7 (568516), murcb (494036), tnnt1 (353248), h3f3c (336231), dap1b (58094), MGC173646 (793447), casq1a (445226), insm1a (402941), zgc:123068 (403118), c9 (554141), col6a2 (567771), ldb3a (794339), emp2 (445506), zgc:86709 (570210), col1a1b (325675), mllt11 (563531), fn1b (334613), hbae3 (30601), zgc:113413 (503710), zgc:114041 (574425), zgc:85869 (406507), pmp22a (334817), LOC562946 (562946), LOC100536795 (100536795), apoa1b (100101640), mab21l2 (117234), h3f3b.1 (550262), si:ch211-218c6.8 (100001903), pnp4a (415192), tnni1al (415191), zgc:174153 (567623), myom1a (558671), slc27a2a (449925), hhatla (393861), atp5h (393675), alas2 (64607), anxa1c (494158), desma (30148), hapln1a (493635), he1a (569018), LOC100334525 (100334525), ism1 (497617), apoa2 (322327), triqk (100147776), dct (58074), mab21l1 (246091), murca (552940), rpl34 (394097), casq2 (114410), kera (567017), and2 (566702), pcdh1gc5 (503571), zgc:77112 (406459), cldni (569084), si:ch211-133n4.4 (559260), slc43a2a (393221), sb:cb252 (321177), fmoda (791822), her12 (402914), ctsl1b (30443), dao.1 (619259), slc2a11b (564970), zgc:114181 (619255), tmsb4x (793949), pcdh1gc6 (503572), wu:fa26c03 (564979), rbp1a (171477), neurod4 (266958), zgc:174855 (569326), LOC100536033 (100536033), myl10 (550569), islr2 (541396), LOC100330405 (100330405), tnc (30037), zgc:171775 (562552), grhprb (402985), pvalb4 (337731), zgc:174689 (795883), tpma (30324), si:ch211-226h8.14 (795554), zgc:86896 (415190), LOC573653 (573653), hbbe2 (405772), si:ch211-117m20.5 (566658), klhl41b (321064), zgc:66286 (393488), ptprsb (100526672), pvalb1 (402805), scel (368684), tmsb (402820), hao1 (402827), zgc:136410 (449685), fabp7a (58128), lmo7b (558333), rtn1a (323706), smyd1a (321245), crygmxl2 (554132), apobb (321166), si:ch211-251b21.1 (571720), si:ch1073-429i10.3 (100331798), lrrn1 (568527), pmela (321239), si:dkey-239j18.3 (100001785), ccnb1l (447870), col11a1b (555202), col4a6 (554268), slmapb (393146), apom (569714), krt4 (794486), micall1 (100149741) | 1479 | 10.5314 | **p-value** 0.001\*\* **permutation** 1000 x **adjusted CI** 2.3657 |
| TermSet 4 terms | obesity, hypertension, type 2 diabetes, osteoporosis |

- (p-value significance: \*\*\*: p < 0.001; \*\*: p < 0.005; \*: p < 0.01)
  

The one-to-all results.

| Gene v.s. all terms | Term v.s. all Genes |
| --- | --- |
| | Gene | Count | CI | | --- | --- | --- | |    apoea (553587) | 403 | 8.6582 | |    rbp4 (30077) | 132 | 7.0553 | |    col1a1a (337158) | 116 | 6.8704 | |    fabp2 (30708) | 73 | 6.2095 | |    f2 (325881) | 71 | 6.1699 | |    apoa1a (30355) | 66 | 6.0661 | |    cpe (407979) | 57 | 5.858 | |    c3a (321046) | 52 | 5.7279 | |    cav1 (323695) | 46 | 5.5546 | |    gstt1a (563972) | 42 | 5.4263 | |    fgb (337315) | 38 | 5.2854 | |    aldh2.2 (368239) | 34 | 5.1293 | |    akr1b1 (415138) | 33 | 5.0875 | |    LOC100333821 (100333821) | 33 | 5.0875 | |    mtp (406207) | 29 | 4.9069 | |    apoa4 (322543) | 29 | 4.9069 | |    zgc:194131 (570354) | 27 | 4.8074 | |    sparc (321357) | 25 | 4.7004 | |    serpina1 (322701) | 22 | 4.5236 | |    serpina1l (321195) | 22 | 4.5236 | |    col1a2 (336471) | 20 | 4.3923 | |    thbs1 (561901) | 20 | 4.3923 | |    tfa (30255) | 19 | 4.3219 | |    fga (378986) | 16 | 4.0875 | |    g6pca.2 (563180) | 14 | 3.9069 | |    nhlh2 (402968) | 14 | 3.9069 | |    gch2 (64263) | 11 | 3.585 | |    plg (322691) | 11 | 3.585 | |    cav3 (449679) | 10 | 3.4594 | |    ptk2bb (245950) | 10 | 3.4594 | |    ptk2.1 (142986) | 10 | 3.4594 | |    icn2 (100005083) | 9 | 3.3219 | |    bhmt (322228) | 9 | 3.3219 | |    icn (336655) | 9 | 3.3219 | |    tgm2b (323856) | 8 | 3.1699 | |    nr2f2 (30424) | 7 | 3.0 | |    slc7a8a (100007704) | 6 | 2.8074 | |    smyhc1 (321552) | 6 | 2.8074 | |    dcn (64698) | 6 | 2.8074 | |    fgg (406327) | 6 | 2.8074 | |    mdka (30277) | 6 | 2.8074 | |    anxa1a (334724) | 6 | 2.8074 | |    actc1b (58114) | 6 | 2.8074 | |    mdkb (65231) | 6 | 2.8074 | |    postnb (337176) | 6 | 2.8074 | |    tgfbi (321421) | 5 | 2.585 | |    fstl1a (325423) | 5 | 2.585 | |    agpat2 (777624) | 5 | 2.585 | |    gpib (246095) | 5 | 2.585 | |    tmem38a (393874) | 4 | 2.3219 | |    pygma (553655) | 4 | 2.3219 | |    ctsc (368704) | 4 | 2.3219 | |    ca2 (387526) | 4 | 2.3219 | |    cxcl14 (58151) | 4 | 2.3219 | |    tyrp1b (437022) | 4 | 2.3219 | |    slc4a4a (568631) | 4 | 2.3219 | |    col5a1 (100334431) | 4 | 2.3219 | |    ttna (317731) | 4 | 2.3219 | |    trpm5 (557722) | 3 | 2.0 | |    actn3a (58037) | 3 | 2.0 | |    ak1 (445486) | 3 | 2.0 | |    lum (415149) | 3 | 2.0 | |    col4a5 (323561) | 3 | 2.0 | |    slit3 (80354) | 3 | 2.0 | |    anxa2a (325557) | 3 | 2.0 | |    zgc:77517 (393540) | 3 | 2.0 | |    gamt (796865) | 3 | 2.0 | |    mat1a (334195) | 3 | 2.0 | |    actn1 (560400) | 2 | 1.585 | |    pls3 (436598) | 2 | 1.585 | |    ckmb (794752) | 2 | 1.585 | |    hinfp (406733) | 2 | 1.585 | |    pabpc4 (321035) | 2 | 1.585 | |    rps3a (337240) | 2 | 1.585 | |    col9a3 (567110) | 2 | 1.585 | |    tnnt3a (58084) | 2 | 1.585 | |    mylpfa (30429) | 2 | 1.585 | |    tnnc2 (58082) | 2 | 1.585 | |    papd4 (553626) | 2 | 1.585 | |    myl1 (336165) | 2 | 1.585 | |    ambp (394093) | 2 | 1.585 | |    slc39a1 (321324) | 2 | 1.585 | |    ces2 (566132) | 2 | 1.585 | |    h3f3a (406269) | 2 | 1.585 | |    runx1t1 (767809) | 2 | 1.585 | |    col5a2a (564821) | 2 | 1.585 | |    matn4 (497348) | 2 | 1.585 | |    acta1a (550445) | 2 | 1.585 | |    gatm (266799) | 2 | 1.585 | |    ckma (30095) | 2 | 1.585 | |    tnnt3b (100003572) | 2 | 1.585 | |    eef2k (437013) | 2 | 1.585 | |    cfl2 (403001) | 2 | 1.585 | |    LOC557250 (557250) | 2 | 1.585 | |    elavl3 (30732) | 2 | 1.585 | |    hbae1 (30597) | 1 | 1.0 | |    meis1 (170446) | 1 | 1.0 | |    rplp2 (335629) | 1 | 1.0 | |    rps15 (337148) | 1 | 1.0 | |    LOC100536875 (100536875) | 1 | 1.0 | |    mgst1.2 (431762) | 1 | 1.0 | |    myhz1.1 (58142) | 1 | 1.0 | |    tnni2b.1 (550249) | 1 | 1.0 | |    rac3b (795167) | 1 | 1.0 | |    myoz1b (436720) | 1 | 1.0 | |    LOC553326 (553326) | 1 | 1.0 | |    atp2a1l (494489) | 1 | 1.0 | |    atp2a1 (260440) | 1 | 1.0 | |    zgc:92868 (445040) | 1 | 1.0 | |    h2afy2 (558711) | 1 | 1.0 | |    tuba1l (573122) | 1 | 1.0 | |    cryba2b (436857) | 1 | 1.0 | |    agr2 (335616) | 1 | 1.0 | |    acta1b (407658) | 1 | 1.0 | |    myhz2 (246275) | 1 | 1.0 | |    lin9 (560867) | 1 | 1.0 | |    dpysl3 (553166) | 1 | 1.0 | |    lsm8 (566017) | 1 | 1.0 | |    hoxb9a (30344) | 1 | 1.0 | |    ivns1abpa (387591) | 1 | 1.0 | |    mylpfb (447930) | 1 | 1.0 | |    det1 (324089) | 1 | 1.0 | |    si:dkey-222f2.1 (568321) | 1 | 1.0 | |    zgc:92533 (445051) | 1 | 1.0 | |    cx30.3 (404725) | 1 | 1.0 | |    LOC100334599 (100334599) | 1 | 1.0 | |    rps9 (114420) | 1 | 1.0 | |    pgam2 (572733) | 1 | 1.0 | |    h1f0 (321618) | 1 | 1.0 | |    rps7 (393725) | 1 | 1.0 | |    pvalb9 (360209) | 1 | 1.0 | |    fkbp9 (445126) | 1 | 1.0 | |    si:ch211-5k11.2 (572729) | 1 | 1.0 | |    cryba2a (415139) | 1 | 1.0 | |    klhl31 (407079) | 1 | 1.0 | |    cyt1l (792062) | 1 | 1.0 | |    si:dkeyp-113d7.4 (553371) | 1 | 1.0 | |    aldoab (406496) | 1 | 1.0 | |    mybpc2b (541384) | 1 | 1.0 | |    nr2f1a (30418) | 1 | 1.0 | |    urah (751742) | 1 | 1.0 | |    tnni2a.4 (494164) | 1 | 1.0 | |    crybb1 (114418) | 1 | 1.0 | |    s100a10b (406276) | 1 | 1.0 | |    ns:zf-e68 (100008070) | 1 | 1.0 | |    pou3f2b (30397) | 1 | 1.0 | |    rpl11 (415229) | 1 | 1.0 | |    myhz1.2 (799300) | 1 | 1.0 | |    fam219aa (445197) | 0 | 0.0 | |    ldb3b (334100) | 0 | 0.0 | |    hbbe3 (30596) | 0 | 0.0 | |    tnnt2d (574000) | 0 | 0.0 | |    uba52 (641289) | 0 | 0.0 | |    si:busm1-118j2.5 (335781) | 0 | 0.0 | |    wu:fd11e11 (386799) | 0 | 0.0 | |    MGC174857 (563390) | 0 | 0.0 | |    ptmaa (335707) | 0 | 0.0 | |    wu:fc13c02 (561837) | 0 | 0.0 | |    he1b (407971) | 0 | 0.0 | |    mylz3 (58143) | 0 | 0.0 | |    hoxb3a (30339) | 0 | 0.0 | |    pcdh1g32 (554016) | 0 | 0.0 | |    pcdh1g31 (554015) | 0 | 0.0 | |    pcdh1g30 (554014) | 0 | 0.0 | |    h3f3d (394076) | 0 | 0.0 | |    pvalb2 (58028) | 0 | 0.0 | |    hkdc1 (321224) | 0 | 0.0 | |    and1 (794315) | 0 | 0.0 | |    MGC174152 (564835) | 0 | 0.0 | |    lyz (677744) | 0 | 0.0 | |    ank2b (450043) | 0 | 0.0 | |    hbbe1.1 (81538) | 0 | 0.0 | |    gpm6aa (368223) | 0 | 0.0 | |    tnnc1b (415175) | 0 | 0.0 | |    zgc:56676 (393273) | 0 | 0.0 | |    napg (436742) | 0 | 0.0 | |    slc4a1a (84703) | 0 | 0.0 | |    tnw (30234) | 0 | 0.0 | |    si:ch73-21g5.7 (568516) | 0 | 0.0 | |    murcb (494036) | 0 | 0.0 | |    tnnt1 (353248) | 0 | 0.0 | |    h3f3c (336231) | 0 | 0.0 | |    dap1b (58094) | 0 | 0.0 | |    MGC173646 (793447) | 0 | 0.0 | |    casq1a (445226) | 0 | 0.0 | |    insm1a (402941) | 0 | 0.0 | |    zgc:123068 (403118) | 0 | 0.0 | |    c9 (554141) | 0 | 0.0 | |    col6a2 (567771) | 0 | 0.0 | |    ldb3a (794339) | 0 | 0.0 | |    emp2 (445506) | 0 | 0.0 | |    zgc:86709 (570210) | 0 | 0.0 | |    col1a1b (325675) | 0 | 0.0 | |    mllt11 (563531) | 0 | 0.0 | |    fn1b (334613) | 0 | 0.0 | |    hbae3 (30601) | 0 | 0.0 | |    zgc:113413 (503710) | 0 | 0.0 | |    zgc:114041 (574425) | 0 | 0.0 | |    zgc:85869 (406507) | 0 | 0.0 | |    pmp22a (334817) | 0 | 0.0 | |    LOC562946 (562946) | 0 | 0.0 | |    LOC100536795 (100536795) | 0 | 0.0 | |    apoa1b (100101640) | 0 | 0.0 | |    mab21l2 (117234) | 0 | 0.0 | |    h3f3b.1 (550262) | 0 | 0.0 | |    si:ch211-218c6.8 (100001903) | 0 | 0.0 | |    pnp4a (415192) | 0 | 0.0 | |    tnni1al (415191) | 0 | 0.0 | |    zgc:174153 (567623) | 0 | 0.0 | |    myom1a (558671) | 0 | 0.0 | |    slc27a2a (449925) | 0 | 0.0 | |    hhatla (393861) | 0 | 0.0 | |    atp5h (393675) | 0 | 0.0 | |    alas2 (64607) | 0 | 0.0 | |    anxa1c (494158) | 0 | 0.0 | |    desma (30148) | 0 | 0.0 | |    hapln1a (493635) | 0 | 0.0 | |    he1a (569018) | 0 | 0.0 | |    LOC100334525 (100334525) | 0 | 0.0 | |    ism1 (497617) | 0 | 0.0 | |    apoa2 (322327) | 0 | 0.0 | |    triqk (100147776) | 0 | 0.0 | |    dct (58074) | 0 | 0.0 | |    mab21l1 (246091) | 0 | 0.0 | |    murca (552940) | 0 | 0.0 | |    rpl34 (394097) | 0 | 0.0 | |    casq2 (114410) | 0 | 0.0 | |    kera (567017) | 0 | 0.0 | |    and2 (566702) | 0 | 0.0 | |    pcdh1gc5 (503571) | 0 | 0.0 | |    zgc:77112 (406459) | 0 | 0.0 | |    cldni (569084) | 0 | 0.0 | |    si:ch211-133n4.4 (559260) | 0 | 0.0 | |    slc43a2a (393221) | 0 | 0.0 | |    sb:cb252 (321177) | 0 | 0.0 | |    fmoda (791822) | 0 | 0.0 | |    her12 (402914) | 0 | 0.0 | |    ctsl1b (30443) | 0 | 0.0 | |    dao.1 (619259) | 0 | 0.0 | |    slc2a11b (564970) | 0 | 0.0 | |    zgc:114181 (619255) | 0 | 0.0 | |    tmsb4x (793949) | 0 | 0.0 | |    pcdh1gc6 (503572) | 0 | 0.0 | |    wu:fa26c03 (564979) | 0 | 0.0 | |    rbp1a (171477) | 0 | 0.0 | |    neurod4 (266958) | 0 | 0.0 | |    zgc:174855 (569326) | 0 | 0.0 | |    LOC100536033 (100536033) | 0 | 0.0 | |    myl10 (550569) | 0 | 0.0 | |    islr2 (541396) | 0 | 0.0 | |    LOC100330405 (100330405) | 0 | 0.0 | |    tnc (30037) | 0 | 0.0 | |    zgc:171775 (562552) | 0 | 0.0 | |    grhprb (402985) | 0 | 0.0 | |    pvalb4 (337731) | 0 | 0.0 | |    zgc:174689 (795883) | 0 | 0.0 | |    tpma (30324) | 0 | 0.0 | |    si:ch211-226h8.14 (795554) | 0 | 0.0 | |    zgc:86896 (415190) | 0 | 0.0 | |    LOC573653 (573653) | 0 | 0.0 | |    hbbe2 (405772) | 0 | 0.0 | |    si:ch211-117m20.5 (566658) | 0 | 0.0 | |    klhl41b (321064) | 0 | 0.0 | |    zgc:66286 (393488) | 0 | 0.0 | |    ptprsb (100526672) | 0 | 0.0 | |    pvalb1 (402805) | 0 | 0.0 | |    scel (368684) | 0 | 0.0 | |    tmsb (402820) | 0 | 0.0 | |    hao1 (402827) | 0 | 0.0 | |    zgc:136410 (449685) | 0 | 0.0 | |    fabp7a (58128) | 0 | 0.0 | |    lmo7b (558333) | 0 | 0.0 | |    rtn1a (323706) | 0 | 0.0 | |    smyd1a (321245) | 0 | 0.0 | |    crygmxl2 (554132) | 0 | 0.0 | |    apobb (321166) | 0 | 0.0 | |    si:ch211-251b21.1 (571720) | 0 | 0.0 | |    si:ch1073-429i10.3 (100331798) | 0 | 0.0 | |    lrrn1 (568527) | 0 | 0.0 | |    pmela (321239) | 0 | 0.0 | |    si:dkey-239j18.3 (100001785) | 0 | 0.0 | |    ccnb1l (447870) | 0 | 0.0 | |    col11a1b (555202) | 0 | 0.0 | |    col4a6 (554268) | 0 | 0.0 | |    slmapb (393146) | 0 | 0.0 | |    apom (569714) | 0 | 0.0 | |    krt4 (794486) | 0 | 0.0 | |    micall1 (100149741) | 0 | 0.0 | | | Term | Count | CI | | --- | --- | --- | |    obesity | 553 | 9.1137 | |    hypertension | 496 | 8.9571 | |    type 2 diabetes | 390 | 8.611 | |    osteoporosis | 184 | 7.5314 | |

**100** of the **1479** PubMed papers involved are listed below (sorted by relevance):

| PubMed ID | Title |
| --- | --- |
| 22028770 | APOE genotype-function relationship: evidence of -491 A/T promoter polymorphism modifying transcription control but not type 2 diabetes risk.BACKGROUND: The apolipoprotein E gene (APOE) coding polymorphism modifies the risks of Alzheimer's disease, type 2 diabetes, and coronary heart disease. Aside from the coding variants, single nucleotide polymorphism (SNP) of the APOE promoter has also been shown to modify the risk of Alzheimer's disease. METHODOLOGY/PRINCIPAL FINDINGS: In this study we investigate the genotype-function relationship of APOE promoter polymorphism at molecular level and at physiological level: i.e., in transcription control of the gene and in the risk of type 2 diabetes. In molecular studies, the effect of the APOE -491A/T (rs449647) polymorphism on gene transcription was accessed by dual-luciferase reporter gene assays. The -491 A to T substitution decreased the activity (p<0.05) of the cloned APOE promoter (-1017 to +406). Using the -501 to -481 nucleotide sequence of the APOE promoter as a 'bait' to screen the human brain cDNA library by yeast one-hybrid system yielded ATF4, an endoplasmic reticulum stress response gene, as one of the interacting factors. Electrophoretic-mobility-shift assays (EMSA) and chromatin immuno-precipitation (ChIP) analyses further substantiated the physical interaction between ATF4 and the APOE promoter. Over-expression of ATF4 stimulated APOE expression whereas siRNA against ATF4 suppressed the expression of the gene. However, interaction between APOE promoter and ATF4 was not -491A/T-specific. At physiological level, the genotype-function relationship of APOE promoter polymorphism was studied in type 2 diabetes. In 630 cases and 595 controls, three APOE promoter SNPs -491A/T, -219G/T (rs405509), and +113G/C (rs440446) were genotyped and tested for association with type 2 diabetes in Hong Kong Chinese. No SNP or haplotype association with type 2 diabetes was detected. CONCLUSIONS/SIGNIFICANCE: At molecular level, polymorphism -491A/T and ATF4 elicit independent control of APOE gene expression. At physiological level, no genotype-risk association was detected between the studied APOE promoter SNPs and type 2 diabetes in Hong Kong Chinese. |
| 19501859 | Factors that influence retinol-binding protein 4-transthyretin interaction are not altered in overweight subjects and overweight subjects with type 2 diabetes mellitus.Retinol-binding protein 4 (RBP4) is an adipokine bound in plasma to transthyretin (TTR), which prevents its glomerular filtration and subsequent catabolism in the kidney. Alterations of this interaction have been suggested to be implicated in the elevation of RBP4 that are thought to contribute to the development of insulin resistance associated with obesity and type 2 diabetes mellitus (T2DM). However, the factors linking RBP4 to TTR in humans are not clear. Therefore, this study evaluated parameters influencing the RBP4-TTR interaction and their relation to obesity and T2DM. The RBP4 and TTR levels were quantified in plasma of 16 lean controls, 28 overweight controls, and 14 overweight T2DM patients by enzyme-linked immunosorbent assay. Transthyretin isoforms involved in RBP4 binding were determined by linear matrix-assisted laser desorption/ionization-time of flight-mass spectrometry after RBP4 coimmunoprecipitation. Holo-RBP4 (retinol-bound) and apo-RBP4 (retinol-free) were assessed by immunoblotting using nondenaturating polyacrylamide gel electrophoresis. Plasma levels of both RBP4 and TTR did not differ among the groups of lean controls, overweight controls, and overweight T2DM subjects. Using RBP4 immunoprecipitation, 4 mass signals were observed for TTR representing native, S-cysteinylated, S-cysteinglycinylated, and S-glutathionylated TTR. No differences in peak intensity of TTR isoforms were observed among the groups. Moreover, no differences in the ratio of holo- and apo-RBP4 were evident. The results suggest that circulating RBP4 and TTR were not affected by human obesity or T2DM, which might be attributed to the absence of alterations of TTR isoforms and the ratio of holo- and apo-RBP4 that might modify the TTR-RBP4 interaction. |
| 17923260 | Evaluation of aortic remodeling in apolipoprotein E-deficient mice and renovascular hypertensive mice.BACKGROUND: The apolipoprotein E-deficient mouse (ApoE) spontaneously develops hypercholesterolemia and atherosclerotic lesions in large arteries. It is also known that angiotensin II-induced hypertension accelerates the development of atherosclerosis in ApoE mice. The objective of this study was to evaluate the aortic remodeling process in ApoE mice during the early phase of atherosclerosis in two-kidney one-clip hypertensive (2K1C) mice and in mice with the coexistence of atherosclerosis and arterial hypertension. METHODS: Renovascular hypertension was induced in 8- to 9-week-old C57BL/6 (C57) and ApoE and compared to sham animals 28 days later. C57-2K1C and ApoE-2K1C mice showed hypertension, tachycardia, and cardiac hypertrophy of similar magnitude. RESULTS: ApoE and ApoE-2K1C mice showed high levels of plasma cholesterol (4.8- and 3.6-fold) and aorta lipid deposition (85- and 101-fold) compared to C57 mice. The aorta lumen area was increased in C57-2K1C and ApoE-2K1C mice (0.57 +/- 0.04 and 0.55 +/- 0.02 mm(2)) compared to C57 mice (0.50 +/- 0.02 mm(2), p <0.05). The aorta wall area was increased by 20% in C57-2K1C and by 12% in ApoE-2K1C mice compared to C57 and ApoE. CONCLUSIONS: The main finding of this study was the absence of aorta remodeling in ApoE mice at the early stage of atherosclerosis and an outward remodeling of similar magnitude in C57-2K1C and ApoE-2K1C mice. |
| 20503483 | Retinol-binding protein 4 in neonates born small for gestational age.BACKGROUND: Retinol-binding protein 4 (RBP4) is an adipocyte-derived 'signal' that may contribute to the pathogenesis of insulin resistance and Type 2 diabetes. The relationship of RBP4 with insulin resistance and metabolic risk in human beings has been the subject of several studies. Subjects born small for gestational age (SGA) are at risk of insulin resistance and Type 2 diabetes. Though RBP4 could represent an early marker of insulin resistance, to date, none have determined RBP4 in SGA children. AIM: Our aim was to measure RBP4 concentrations in cord blood of SGA newborns compared with those in children born with a birth weight appropriate for gestational age (AGA) and to determine whether serum RBP4 levels at birth correlate with insulin sensitivity markers. SUBJECTS AND METHODS: Sixty-four newborns, 17 born SGA (mean gestational age: 36.4+/-2.1 weeks), and 47 born AGA (mean gestational age: 37.0+/-3.6 weeks) were studied. The main outcome measures included anthropometry, lipid profile, insulin, homeostasis model assessment, quantitative insulin-sensitivity check index, adiponectin, and RBP4. RESULTS: RBP4 concentrations were significantly reduced in SGA newborns (p<0.002). No relationship was found between RBP4 and insulin sensitivity parameters. Stepwise regression analysis revealed that birth weight was the major predictor of RBP4 serum concentrations (p<0.001). CONCLUSION: RBP4 is reduced in SGA newborns, birth weight representing the major determinant of RBP4 concentrations, and is not related to insulin sensitivity. No significant difference in adiponectin levels and insulin sensitivity markers was found between SGA and AGA neonates. |
| 9662053 | Organization of the human carboxypeptidase E gene and molecular scanning for mutations in Japanese subjects with NIDDM or obesity.Insulin is synthesized in the pancreatic beta cell as a larger precursor molecule proinsulin which is converted to insulin and C-peptide by the concerted action of prohormone convertase 2 (PC2), prohormone convertase 3 (PC3) and carboxypeptidase E (CPE). One of the features of non-insulin-dependent diabetes mellitus (NIDDM) is an elevation in the proinsulin level and/or proinsulin/insulin molar ratio suggesting that mutations in these three proinsulin processing enzymes might contribute to the development of NIDDM. The identification of a mutation in the CPE gene of the fat/fat mouse which leads to marked hyperproinsulinaemia and late-onset obesity and diabetes is consistent with a possible role for mutations in CPE in the development of diabetes and obesity in humans. In order to test this hypothesis, we have isolated and characterized the human CPE gene and screened it for mutations in a group of Japanese subjects with NIDDM and obesity. The human CPE gene consists of 9 exons spanning more than 60 kb. Primer extension analysis identified the transcriptional start site at -141 bp from the translational start site. Single strand conformational polymorphism analysis and nucleotide sequencing of the promoter and entire coding region of the CPE gene in 269 Japanese subjects with NIDDM, 28 nondiabetic obese subjects and 104 nonobese and nondiabetic controls revealed three nucleotide changes, a G-to-T substitution at nucleotide -53, a G-to-A substitution at nucleotide -144 (relative to start of transcription) in the promoter region and a silent G-to-A substitution in codon 219. None of the nucleotide substitutions were associated with NIDDM or obesity. Thus, genetic variation in the CPE gene does not appear to play a major role in the pathogenesis of NIDDM or obesity in Japanese subjects. |
| 11445660 | The expression of SPARC in adipose tissue and its increased plasma concentration in patients with coronary artery disease.OBJECTIVE: Adipocytes secrete various cytokines and matrix proteins. Several of them precipitate in obesity-associated diseases, including atherosclerosis. In the current study, we have examined the expression of secreted protein, acidic and rich in cysteine (SPARC) in adipose tissue and its significance in obesity and coronary artery disease (CAD). RESEARCH METHODS AND PROCEDURES: The SPARC mRNA expressions both in vivo and in vitro were detected by Northern blot analysis. Plasma SPARC concentrations were measured by enzyme immunosorbent assay. First, we investigated the plasma SPARC levels of 88 unrelated adult Japanese subjects (62 men and 26 women; average age: [+/- SD] 50 +/- 12 years; body mass index [BMI]: 16 to 46 kg/m(2)). Additionally 31 subjects with CAD diagnosed by coronary angiography (20 men and 11 women) were also investigated. RESULTS: Human adipose tissues expressed abundant SPARC mRNA. SPARC expression in adipose tissues was upregulated in obese db/db mice. Markedly enhanced expression of SPARC mRNA was observed in 3T3-L1 fibroblasts during adipocyte differentiation. Consistent with these results, plasma SPARC levels proved a positive correlation with BMI in humans (r = 0.27; p < 0.01). Interestingly, plasma SPARC concentrations were significantly elevated in age- and BMI-matched subjects with CAD (p < 0.05). DISCUSSION: SPARC was expressed in adipose tissues and its expression was enhanced in obese mice. In human, plasma SPARC levels were elevated in obesity and CAD patients. This elevated SPARC may be involved in the progression of CAD. |
| 22330623 | Association of manganese superoxide dismutase and glutathione S-transferases genotypes with carotid atherosclerosis in patients with diabetes mellitus type 2.AIM: The aim of the present study was to test the association between genetic polymorphisms with functional effects on redox regulation: Ala16Val of manganese superoxide dismutase (MnSOD or SOD2), polymorphic deletions of glutathione S-transferases M1 (GSTM1) and T1 (GSTT1) and Ile105Val of the GSTP1 and carotid atherosclerosis in patients with type 2 diabetes. METHODS: The study enrolled 287 subjects with type 2 diabetes. Carotid atherosclerosis was quantified by ultrasonography as carotid intima-media thickness (CITM), plaque score from 0 to 6 and plaque type from 1 to 5. Genotypes were determined by polymerase chain reaction (PCR) and restriction fragment length polymorphism (RFLP). RESULTS: The highest triglyceride level was observed in patients with MnSOD Val/Val genotype. Other polymorphisms did not show significant association with clinical parameters. We did not observe significant differences in MnSOD, GSTM1 and GSTP1 genotypes distribution according to CIMT, plaque type or plaque score. After adjustment for age, sex, smoking, BMI, lipid parameters and duration of hypertension and diabetes carriers of GSTT1-0 genotype showed an increased risk for higher plaque score (OR=2.29; p=0.012), but no association with CIMT and plaque stability was observed. Carrying of both GSTM1-0 and GSTT1-0 did not influence clinical parameters but increased risk for higher plaque score (OR=2.59; P=0.018). CONCLUSION: We did not find a significant association between the MnSOD, GSTM1 and GSTP1 polymorphisms and carotid atherosclerosis. The GSTT1-0 genotype and GSTT1-0/GSTM1-0 haplotype might be a potential determinants of susceptibility to advanced atherosclerosis in patients with type 2 diabetes mellitus. |
| 17339547 | Pulmonary arterial hypertension is linked to insulin resistance and reversed by peroxisome proliferator-activated receptor-gamma activation.BACKGROUND: Patients with pulmonary arterial hypertension (PAH) have reduced expression of apolipoprotein E (apoE) and peroxisome proliferator-activated receptor-gamma in lung tissues, and deficiency of both has been linked to insulin resistance. ApoE deficiency leads to enhanced platelet-derived growth factor signaling, which is important in the pathobiology of PAH. We therefore hypothesized that insulin-resistant apoE-deficient (apoE-/-) mice would develop PAH that could be reversed by a peroxisome proliferator-activated receptor-gamma agonist (eg, rosiglitazone). METHODS AND RESULTS: We report that apoE-/- mice on a high-fat diet develop PAH as judged by elevated right ventricular systolic pressure. Compared with females, male apoE-/- were insulin resistant, had lower plasma adiponectin, and had higher right ventricular systolic pressure associated with right ventricular hypertrophy and increased peripheral pulmonary artery muscularization. Because male apoE-/- mice were insulin resistant and had more severe PAH than female apoE-/- mice, we treated them with rosiglitazone for 4 and 10 weeks. This treatment resulted in markedly higher plasma adiponectin, improved insulin sensitivity, and complete regression of PAH, right ventricular hypertrophy, and abnormal pulmonary artery muscularization in male apoE-/- mice. We further show that recombinant apoE and adiponectin suppress platelet-derived growth factor-BB-mediated proliferation of pulmonary artery smooth muscle cells harvested from apoE-/- or C57Bl/6 control mice. CONCLUSIONS: We have shown that insulin resistance, low plasma adiponectin levels, and deficiency of apoE may be risk factors for PAH and that peroxisome proliferator-activated receptor-gamma activation can reverse PAH in an animal model. |
| 18467438 | Tumor necrosis factor-alpha-mediated suppression of adipocyte apolipoprotein E gene transcription: primary role for the nuclear factor (NF)-kappaB pathway and NFkappaB p50.The adipose tissue inflammation accompanying obesity has important consequences for adipocyte lipid metabolism, and increased adipose tissue TNFalpha plays an important role for mediating the effect of inflammation on adipocyte function. Recent studies have shown that apolipoprotein E (apoE) is highly expressed in adipose tissue where it plays an important role in modulating adipocyte triglyceride metabolism, triglyceride mass, and adipocyte size. We have previously reported that TNFalpha reduces adipocyte apoE, and the current studies were undertaken to evaluate the molecular mechanism for this regulation. TNFalpha repression of adipocyte apoE gene expression required an intact nuclear factor (NF)-kappaB binding site at -43 in the apoE promoter. Site-directed mutagenesis at this site completely eliminated TNFalpha regulation of an apoE gene reporter. TNFalpha treatment activated binding of NFkappaB p50, isolated from adipocyte nuclei, to the apoE promoter. Two structurally distinct inhibitors of NFkappaB complex activation or translocation abrogated the TNFalpha effect on the apoE gene. Using chromatin immunoprecipitation assays, we demonstrated that treatment of adipocytes with TNFalpha led to increased binding of NFkappaB p50, and decreased binding of p65 and Sp1, to this region of the apoE promoter in living cells. The key role played by increased p50 binding was confirmed by p50 knockdown experiments. Reduction of p50 expression using small interference RNA completely eliminated TNFalpha-mediated reduction of endogenous adipocyte apoE gene expression. These results establish the molecular link between adipose tissue inflammation and apoE gene expression in adipocytes. The suppression of adipocyte apoE by the proinflammatory adipose tissue milieu associated with obesity will have important downstream effects on adipocyte triglyceride turnover and content. |
| 17003346 | Retinol-binding protein 4 in human obesity.Studies in mice suggest that adipocytes serve as glucose sensors and regulate systemic glucose metabolism through release of serum retinol-binding protein 4 (RBP4). This model has not been validated in humans. RBP4 was highly expressed in isolated mature human adipocytes and secreted by differentiating human adipocytes. In contrast to the animal data, RBP4 mRNA was downregulated in subcutaneous adipose tissue of obese women, and circulating RBP4 concentrations were similar in normal weight, overweight, and obese women (n = 74). RBP4 was positively correlated with GLUT4 expression in adipose tissue, independent of any obesity-associated variable. Five percent weight loss slightly decreased adipose RBP4 expression but did not influence circulating RBP4. In another set of experiments, we stratified patients (n = 14) by low or high basal fasting interstitial glucose concentrations, as determined by the microdialysis technique. Venous glucose concentrations were similar throughout oral glucose tolerance testing, and basal RBP4 expression in adipose tissue and serum RBP4 concentrations were similar in the groups with higher and lower interstitial glucose levels. Our findings point to profound differences between rodents and humans in the regulation of adipose or circulating RBP4 and challenge the notion that glucose uptake by adipocytes has a dominant role in the regulation of RBP4. |
| 19003725 | Visceral obesity is associated with the metabolic syndrome and elevated plasma retinol binding protein-4 level in obstructive sleep apnea syndrome.Obstructive sleep apnea syndrome (OSAS) is related to the increased prevalence of cardiovascular disease and metabolic syndrome (MS). A novel adipokine, retinol binding protein-4 (RBP4), was reported to be associated with insulin resistance and the prevalence of type 2 diabetes. To examine whether plasma RBP4 is associated with insulin resistance and MS development in OSAS, we measured plasma RBP4 levels in 181 Japanese men (24 healthy controls and 40 mild, 64 moderate, and 53 severe OSAS) of whom 26 had mild glucose intolerance with HbA1c < or = 6.0%. After a full polysomnography, blood was collected between 06:00 and 07:00 AM. Plasma RBP4 levels in moderate/severe OSAS patients were higher than in control subjects. Plasma RBP4 was not correlated with apnea variables, HOMA-IR, or blood pressure. However, it was positively correlated with visceral fat areas and plasma triglyceride levels. The prevalence of MS was higher in severe OSAS patients than in mild/moderate OSAS and control subjects. Plasma RBP4 was higher in OSAS patients with MS than in those without MS. This study indicates that plasma RBP4 is associated with dyslipidemia, but not with insulin resistance, glucose intolerance, or hypertension in patients with OSAS. Visceral obesity may play key roles in increasing the plasma RBP4 level and MS development in OSAS. |
| 19408098 | Apolipoprotein A1 gene polymorphisms as risk factors for hypertension and obesity.Several polymorphisms in apolipoprotein A1 (APOA1) gene have been associated with metabolic diseases. Increased transcription efficiency was observed in -75A allele carriers compared to -75G allele homozygotes. +83C allele was associated with higher body mass index and waist-to-hip ratio in type II diabetes subjects. -75G/A and +83C/T polymorphisms were analyzed by RFLP-PCR in 334 individuals from a Brazilian elderly cohort. APOA1 polymorphisms were associated with age-related morbidities, as well as with triglycerides, total cholesterol, HDL, VLDL, LDL, creatinine, urea, albumin, glycated hemoglobin and fasting glucose serum levels. Allele frequencies were 0.102 and 0.21, respectively, for -75A and +83T. -75G allele showed significant association with hypertension (P = 0.001). An association between +83C allele and obesity was observed (P = 0.040) and this allele also showed an association with hypertension in the presence of cardiovascular disease (P = 0.047). Moreover, +83T allele was associated with lower glycated hemoglobin values (P = 0.026). To our knowledge, there is no data associating this polymorphism with glycated hemoglobin. Furthermore, individuals carrying AT haplotype have lower risk for developing hypertension (P = 0.0002), while GT haplotype carriers present decreased risk to develop obesity comparing to GC haplotype (P = 0.025). APOA1 polymorphisms analysis may be a useful tool to identify risk factors for subjects and families and clarify the physiopathological role of these polymorphisms in age-related diseases, such as hypertension and obesity. |
| 19509023 | Regulation of the fibrosis and angiogenesis promoter SPARC/osteonectin in human adipose tissue by weight change, leptin, insulin, and glucose.OBJECTIVE: Matricellular Secreted Protein, Acidic and Rich in Cysteine (SPARC), originally discovered in bone as osteonectin, is a mediator of collagen deposition and promotes fibrosis. Adipose tissue collagen has recently been found to be linked with metabolic dysregulation. Therefore, we tested the hypothesis that SPARC in human adipose tissue is influenced by glucose metabolism and adipokines. RESEARCH DESIGN AND METHODS: Serum and adipose tissue biopsies were obtained from morbidly obese nondiabetic subjects undergoing bariatric surgery and lean control subjects for analysis of metabolic markers, SPARC, and various cytokines (RT-PCR). Additionally, 24 obese subjects underwent a very-low-calorie diet of 1,883 kJ (450 kcal)/day for 16 weeks and serial subcutaneous-abdominal-adipose tissue (SCAT) biopsies (weight loss: 28 +/- 3.7 kg). Another six lean subjects underwent fast-food-based hyperalimentation for 4 weeks (weight gain: 7.2 +/- 1.6 kg). Finally, visceral adipose tissue explants were cultured with recombinant leptin, insulin, and glucose, and SPARC mRNA and protein expression determined by Western blot analyses. RESULTS: SPARC expression in human adipose tissue correlated with fat mass and was higher in SCAT. Weight loss induced by very-low-calorie diet lowered SPARC expression by 33% and increased by 30% in adipose tissue of subjects gaining weight after a fast-food diet. SPARC expression was correlated with leptin independent of fat mass and correlated with homeostasis model assessment-insulin resistance. In vitro experiments showed that leptin and insulin potently increased SPARC production dose dependently in visceral adipose tissue explants, while glucose decreased SPARC protein. CONCLUSIONS: Our data suggest that SPARC expression is predominant in subcutaneous fat and its expression and secretion in adipose tissue are influenced by fat mass, leptin, insulin, and glucose. The profibrotic effects of SPARC may contribute to metabolic dysregulation in obesity. |
| 22787137 | Glucotoxicity induces glucose-6-phosphatase catalytic unit expression by acting on the interaction of HIF-1alpha with CREB-binding protein.The activation of glucose-6-phosphatase (G6Pase), a key enzyme of endogenous glucose production, is correlated with type 2 diabetes. Type 2 diabetes is characterized by sustained hyperglycemia leading to glucotoxicity. We investigated whether glucotoxicity mechanisms control the expression of the G6Pase catalytic unit (G6pc). We deciphered the transcriptional regulatory mechanisms of the G6pc promoter by glucotoxicity in a hepatoma cell line then in primary hepatocytes and in the liver of diabetic mice. High glucose exposure induced the production of reactive oxygen species (ROS) and, in parallel, induced G6pc promoter activity. In hepatocytes, glucose induced G6pc gene expression and glucose release. The decrease of ROS concentrations by antioxidants eliminated all the glucose-inductive effects. The induction of G6pc promoter activity by glucose was eliminated in the presence of small interfering RNA, targeting either the hypoxia-inducible factor (HIF)-1alpha or the CREB-binding protein (CBP). Glucose increased the interaction of HIF-1alpha with CBP and the recruitment of HIF-1 on the G6pc promoter. The same mechanism might occur in hyperglycemic mice. We deciphered a new regulatory mechanism induced by glucotoxicity. This mechanism leading to the induction of HIF-1 transcriptional activity may contribute to the increase of hepatic glucose production during type 2 diabetes. |
| 23420326 | Retinol-binding protein 4 as a risk factor for cholesterol gallstone formation.Retinol-binding protein 4 (RBP4) is closely associated with a variety of abnormal glycolipid metabolism diseases such as insulin resistance, obesity, diabetes mellitus, and metabolic syndrome. Part 1 Twenty-four male C57/B6 mice were randomly divided into a normal control group (group C, n = 8), a gallstone group (group G, n = 8), and a medication group (group M, n = 8). Serum RBP4 levels were measured by ELISA. RBP4 expression in liver and adipose tissue was detected by western blotting. The transcription of RBP-4 mRNA, PPAR-gamma mRNA in the liver was measured using real-time PCR. The incidences of gallstone formation were 0/8 (group C), 7/8 (group G), and 2/8 (group M) (p < 0.01). Group G had significantly higher body weight, adipose mass, fasting glucose (FG), serum RBP4, total cholesterol (TC), triglycerides (TG), and high-density lipoprotein cholesterol (HDL-C) than group C. In group M, pioglitazone decreased body weight, adipose mass, levels of serum RBP4, FG, TC, and TG, and it increased levels of HDL-C. Pioglitazone downregulated RBP4 expression in hepatic and adipose tissue and prevented decreases in PPAR-gammamRNA levels induced by lithogenic diet. Part 2 Metabolism indices, including serum RBP4, FG, TC, TG, HDL-C, low-density lipoprotein cholesterol (LDL-C), alanine aminotransferase (ALT), and aspartate aminotransferase (AST) of 110 human cholesterol gallstone patients and 73 healthy controls were collected for further analysis. Patients with gallstones had elevated levels of serum RBP4, FG, TC, TG, ALT, and AST, and had decreased HDL-C levels compared to those of healthy controls. Elevated RBP4 is associated with the morbidity of cholesterol gallstones and metabolic syndrome. RBP4 may play an important role in the course of cholesterol gallstone formation. |
| 16207841 | APOE-epsilon4 predisposes to cognitive dysfunction following uncomplicated carotid endarterectomy.BACKGROUND: Between 9% and 23% of patients undergoing otherwise uncomplicated carotid endarterectomy (CEA) develop subtle cognitive decline 1 month postoperatively. The APOE-epsilon4 allele has been associated with worse outcome following stroke. OBJECTIVE: To investigate the ability of APOE-epsilon4 to predict post-CEA neurocognitive dysfunction. METHODS: Seventy-five patients with CEA undergoing elective CEA were prospectively recruited in this nested cohort study and demographic variables were recorded. Patients were evaluated before and 1 month after surgery with a standard battery of five neuropsychological tests. APOE genotyping was performed by restriction fragment length polymorphism analysis in all patients. Neuropsychological deficits were identified by comparing changes (before to 1 month post-operation) in individual performance on the test battery. Logistic regression was performed for APOE-epsilon4 and previously identified risk factors. RESULTS: Twelve of 75 (16%) CEA patients possessed the APOE-epsilon4 allele. Eight of 75 (11%) patients experienced neurocognitive dysfunction on postoperative day 30. One month post-CEA, APOE-epsilon4-positive patients were more likely to be cognitively injured (42%) than APOE-epsilon4-negative patients (5%) (p = 0.002). In multivariate analysis, the presence of the APOE-epsilon4 allele increased the risk of neurocognitive dysfunction at 1 month 62-fold (62.28, 3.15 to 1229, p = 0.007). Diabetes (51.42, 1.94 to 1363, p = 0.02), and obesity (24.43, 1.41 to 422.9, p = 0.03) also predisposed to injury. CONCLUSION: The APOE-epsilon4 allele is a robust independent predictor of neurocognitive decline 1 month following CEA. |
| 17299074 | Retinol-binding protein 4 is associated with insulin resistance and body fat distribution in nonobese subjects without type 2 diabetes.BACKGROUND: Adipose tissue is responsible for releasing various adipokines that have been related to insulin resistance. Understanding the relationship of these adipokines to insulin resistance may foster the development of new treatments for diabetes. OBJECTIVES: The primary objective of this study was to determine whether an association between retinol-binding protein 4 (RBP4) and insulin resistance exists in nonobese individuals without a family history or diagnosis of diabetes. The secondary objective was to determine by a dual energy x-ray absorptiometry scan which adipose tissue depot most closely relates to RBP4 levels. DESIGN: Cross-sectional analysis of 92 study participants ranging in age from 20 to 83 yr was performed. The range of body mass index (BMI) was from 18 to 30 kg/m(2). Exclusion criteria were a BMI greater than 30 kg/m(2), family history of diabetes, or a diagnosis of diabetes. Insulin sensitivity was determined by a hyperinsulinemic euglycemic clamp. Body fat was measured by dual energy x-ray absorptiometry scan. RESULTS: RBP4 values were lower in females (35.8 +/- 1.7 microg/ml) compared with males (39.9 +/- 1.4 microg/ml; P = 0.06). RBP4 levels were found to correlate negatively with insulin sensitivity (r = -0.32; P = 0.002) and positively with age (r = 0.38; P < 0.001). RBP4 levels did not correlate with BMI (r = -0.13; P = 0.22), trunk fat (r = 0.16; P = 0.22), or percent body fat (r = 0.07; P = 0.65). However, RBP4 levels did correlate with percent trunk fat (r = 0.36; P = 0.001). CONCLUSION: These findings indicate a relationship between RBP4, insulin sensitivity, and percent trunk fat in individuals who may not have features of insulin resistance. |
| 9090387 | Hypogonadism and obesity in mice with a targeted deletion of the Nhlh2 gene.The family of basic helix-loop-helix (bHLH) genes comprises transcription factors involved in many aspects of growth and development. We have previously described two bHLH transcription factors, Nhlh1 and Nhlh2 (originally named NSCL1 and NSCL2). The nucleotide and predicted protein sequences of Nhlh1 and Nhlh2 are homologous within their bHLH domain where there are only three conservative amino acid differences. During murine embryogenesis, Nhlh1 and Nhlh2 share an overlapping but distinct pattern of expression in the developing nervous system. To improve our understanding of the role of these genes during neurogenesis, we have generated mice containing targeted deletions of both genes and here describe our results for Nhlh2. Loss of Nhlh2 results in a disruption of the hypothalamic-pituitary axis in mice. Male Nhlh2-/- mice are microphallic, hypogonadal and infertile with alterations in circulating gonadotropins, a defect in spermatogenesis and a loss of instinctual male sexual behaviour. Female Nhlh2-/- mice reared alone are hypogonadal, but when reared in the presence of males, their ovaries and uteri develop normally and they are fertile. Both male and female homozygotes exhibit progressive adult-onset obesity. Nhlh2 is expressed in the ventral-medial and lateral hypothalamus, Rathke's pouch and in the anterior lobe of the adult pituitary. Our results support a role for Nhlh2 in the onset of puberty and the regulation of body weight metabolism. |
| 15118095 | Metabolic syndrome without obesity: Hepatic overexpression of 11beta-hydroxysteroid dehydrogenase type 1 in transgenic mice.In obese humans and rodents there is increased expression of the key glucocorticoid (GC) regenerating enzyme, 11beta-hydroxysteroid dehydrogenase type 1 (11beta-HSD1), in adipose tissue. This increased expression appears to be of pathogenic importance because transgenic mice overexpressing 11beta-HSD1 selectively in adipose tissue exhibit a full metabolic syndrome with visceral obesity, dyslipidemia, insulin-resistant diabetes, and hypertension. In this model, while systemic plasma GC levels are unaltered, GC delivery to the liver via the portal vein is increased. 11beta-HSD1 is most highly expressed in liver where inhibition or deficiency of its activity improves glucose and lipid homeostasis. To determine the potential contribution of elevated intrahepatic GCs alone toward development of insulin-resistant syndromes we generated transgenic mice expressing increased 11beta-HSD1 activity selectively in the liver under transcriptional control of hepatic regulatory sequences derived from the human apoE gene (apoE-HSD1). Transgenic lines with 2- and 5-fold-elevated 11beta-HSD1 activity exhibited mild insulin resistance without altered fat depot mass. ApoE-HSD1 transgenic mice exhibited fatty liver and dyslipidemia with increased hepatic lipid synthesis/flux associated with elevated hepatic LXRalpha and PPARalpha mRNA levels as well as impaired hepatic lipid clearance. Further, apoE-HSD1 transgenic mice have a marked, transgene-dose-associated hypertension paralleled by incrementally increased liver angiotensinogen expression. These data suggest that elevated hepatic expression of 11beta-HSD1 may relate to the pathogenesis of specific fatty liver, insulin-resistant, and hypertensive syndromes without obesity in humans as may occur in, for example, myotonic dystrophy, and possibly, the metabolically obese, normal-weight individual. |
| 21664420 | Melanocortin 4 receptor is a transcriptional target of nescient helix-loop-helix-2.Melanocortin 4 receptor (Mc4r/MC4R) is a G-Protein coupled receptor that is expressed in the hypothalamus and implicated in body weight control. Mutations in MC4R are the most frequent cause of monogenetic forms of human obesity. Despite its importance, the MC4R signaling pathways and transcriptional regulation underlying the melanocortin pathway are far from being fully understood. The transcription factor nescient helix-loop-helix 2 (Nhlh2) influences the melanocortin pathway through transcriptional regulation of prohormone convertase I, which influences the production of melanocortin peptides. In the present study, Nhlh2's role as a transcriptional regulator of Mc4r has been demonstrated. Nhlh2 knockout mice have reduced hypothalamic expression of Mc4r mRNA, suggesting that it could be a direct or indirect transcriptional regulator of the Mc4r promoter. To demonstrate direct transcriptional regulation, chromatin immunoprecipitation and electrophoretic gel shift assays show that Nhlh2 binds to the E-Boxes located at -551, -366 and +54 on the Mc4r promoter. Leptin-induced transactivation of the Mc4r promoter is significantly higher in the presence of exogenously added Nhlh2. siRNA knockdown of Nhlh2 leads to significantly reduced endogenous Mc4r mRNA expression levels in N29/2 cell line. Transactivation using promoters with mutations in each of the E-Boxes results in significantly reduced transactivation efficiency compared to the WT Mc4r promoter, suggesting that Nhlh2 regulates Mc4r transcription through these sites. Findings from these studies, combined with previous work implicating Nhlh2 as a transcriptional regulator of both the Mc4r gene and the melanocortin pathway, suggest that Nhlh2's transcriptional activity directly influences the human and rodent body weight control pathways. |
| 10821138 | Association of polymorphism in the promoter region of the apolipoprotein E gene with diastolic blood pressure in normotensive Japanese.The epsilon4 allele of apolipoprotein E (APOE) is reported to be a genetic risk factor of atherosclerosis through hyperlipidemia and late-onset Alzheimer's dementia. A recent report showed that a genetic variant (A -491T) in the promoter region of the APOE gene increases the risk of Alzheimer's disease. In the present study, we examined whether these APOE polymorphisms were genetically involved in essential hypertension. Japanese hypertensives (n=180) with a family history of hypertension and normotensive controls (n=195, sex and age matched with hypertensives) were recruited from the outpatients of Osaka University Hospital, and an informed consent to participate in the study was obtained from each person. APOE polymorphisms were determined using polymerase chain reaction-restriction fragment length polymorphism (PCR-RFLP). The frequencies of the A -491 allele in hypertensives and normotensives were 0.98 and 0.97, respectively, and the TT/-491 genotype was not found in either group. No significant differences between hypertensives and normotensives were observed in allele frequencies in either APOE polymorphism; however, the mean diastolic blood pressure in normotensive subjects with AA/-491 was significantly higher than in the subjects with AT/-491 (p < 0.01). These results suggest that the presence of the APOE promoter polymorphism is not a major risk factor for hypertension but that it does have some minor effect on basal blood pressure variation. |
| 11879722 | Association of plasma lipid levels with apolipoprotein E polymorphism in Type 2 diabetes.OBJECTIVE: To evaluate the distribution of apolipoprotein E polymorphism in patients with Type 2 diabetes and their impact on plasma lipid levels. Subjects: Unrelated Type 2 diabetic patients (n = 298) treated by diet and sulfonylurea and not receiving lipid-lowering regimens, elderly (n = 98) and young (n = 101)unrelated healthy control subjects in Hungary. METHODS: Apolipoprotein E genotypes were identified by PCR amplification and subsequent restriction endonuclease digestion. RESULTS: The distribution of the most frequent genotypes in the diabetes group was E2/3 8.7%, E3/3 78.2%, E3/4 12.8%, in the elderly group E2/3 9.2%, E3/3 78.6%, E3/4 12.2% and in the young group E2/3 11.9%, E3/3 62.4%, E3/4 24.8%. The frequencies of allele e4 in the diabetes and in the elderly control group were significantly lower than in the young control group (both P < 0.05). Associations were found between the e4 allele and increased triglyceride level in the diabetes group, the e2 allele and decreased total cholesterol and LDL-cholesterol levels both in the elderly and young control groups (both P < 0.01). CONCLUSION: The lower frequency of allele e4 in both the elderly and diabetes groups, may be explained by an increased morbidity and mortality in middle-aged carriers of apo e4 allele. The increased risk of e4 carriers in Type 2 diabetes may be partly mediated by a higher triglyceride level. |
| 12461395 | COL9A3 gene polymorphism and obesity in intervertebral disc degeneration of the lumbar spine: evidence of gene-environment interaction.STUDY DESIGN: Cross-sectional. OBJECTIVES: To evaluate the interaction between the COL9A3 gene polymorphism and persistent obesity in relation to lumbar disc degeneration. SUMMARY OF BACKGROUND DATA: Obesity has been suggested to be a risk factor for disc degeneration. There is some indication for an association between collagen IX genes and lumbar disc disease characterized by sciatica. However, the interaction between those factors in their influences on the risk of disc degeneration has not been studied. METHODS: Blood samples from 135 middle-aged men who had undergone magnetic resonance imaging (MRI) of the lumbar spine were analyzed for the presence of an arginine to tryptophan change in the COL9A3 gene (Trp3 allele). The men represented three occupations: 41 were machine drivers, 42 were carpenters, and 52 were office workers. The discs L2/L3-L5/S1 were evaluated on MRI, using decreased signal intensity of the nucleus pulposus, posterior disc bulges, and decreased disc height as signs of disc degeneration. Based on self-reports on body height and weight currently and at the age of 25 years, obesity history was classified as no obesity, persistent obesity, and other. Rothman's synergy index was used as a measure of interaction between two factors. RESULTS: The Trp3 allele and persistent obesity acted synergistically to increase the risk of dark nucleus pulposus, posterior disc bulge, and decreased disc height at L4/L5; of multilevel posterior disc bulges; and of decreased disc height. From 45% to 71% of disc degeneration among persistently obese individuals with the Trp3 allele could be attributed to the synergism of these two factors. CONCLUSION: The effect of obesity on lumbar disc degeneration seems to be modified by the collagen IX gene polymorphism, so that people who carry the Trp3 allele are at increased risk if they are persistently obese. |
| 19556974 | Retinol-binding protein-4 in women with untreated essential hypertension.BACKGROUND: Retinol-binding protein-4 (RBP4) is a novel adipokine able to modulate the action of insulin in several tissues. A variable degree of insulin resistance characterizes the vast majority of hypertensive (HYP) patients. The aim of this study was to evaluate the relationship between RBP4 and essential hypertension, exploring potential links between RBP4 and other adipokines with some proxies of early vascular damage in female naive HYP patients. METHODS: Serum RBP4, leptin, adiponectin, and resistin levels were determined in 35 HYP and 35 normotensive lean women with normal glucose tolerance paired by age and body mass index (BMI) served as controls (CTL); carotid intima-media thickness (IMT) was also measured. RESULTS: A striking difference was observed in RBP4 levels between HYP and CTL with significantly higher levels in the former than in the latter. No relationship was observed between glomerular filtration rate (GFR) and RBP4. Adiponectin levels were slightly but significantly lower in HYP than in CTL, whereas no differences were observed in resistin and leptin concentrations between the two groups of women. In the whole study group, a strong linear relationship was observed between IMT value and both RBP4 (rho = 0.321, P = 0.0076) and resistin (rho = 0.340, P = 0.0048); these two adipocytokines, together with cholesterol, were the only variables independently related to IMT (r(2) = 0.24; P = 0.004) by a stepwise analysis. CONCLUSIONS: RBP4 levels are increased in naive HYP women and correlated with the degree of IMT suggesting a participation of this adipocytokine in the modulation of the atherosclerotic process exerted by the adipose tissue as endocrine organ. |
| 19738092 | Genetic modifiers of liver disease in cystic fibrosis.CONTEXT: A subset (approximately 3%-5%) of patients with cystic fibrosis (CF) develops severe liver disease with portal hypertension. OBJECTIVE: To assess whether any of 9 polymorphisms in 5 candidate genes (alpha(1)-antitrypsin or alpha(1)-antiprotease [SERPINA1], angiotensin-converting enzyme [ACE], glutathione S-transferase [GSTP1], mannose-binding lectin 2 [MBL2], and transforming growth factor beta1 [TGFB1]) are associated with severe liver disease in patients with CF. DESIGN, SETTING, AND PARTICIPANTS: Two-stage case-control study enrolling patients with CF and severe liver disease with portal hypertension (CFLD) from 63 CF centers in the United States as well as 32 in Canada and 18 outside of North America, with the University of North Carolina at Chapel Hill as the coordinating site. In the initial study, 124 patients with CFLD (enrolled January 1999-December 2004) and 843 control patients without CFLD were studied by genotyping 9 polymorphisms in 5 genes previously studied as modifiers of liver disease in CF. In the second stage, the SERPINA1 Z allele and TGFB1 codon 10 genotype were tested in an additional 136 patients with CFLD (enrolled January 2005-February 2007) and 1088 with no CFLD. MAIN OUTCOME MEASURES: Differences in distribution of genotypes in patients with CFLD vs patients without CFLD. RESULTS: The initial study showed CFLD to be associated with the SERPINA1 Z allele (odds ratio [OR], 4.72; 95% confidence interval [CI], 2.31-9.61; P = 3.3 x 10(-6)) and with TGFB1 codon 10 CC genotype (OR, 1.53; 95% CI, 1.16-2.03; P = 2.8 x 10(-3)). In the replication study, CFLD was associated with the SERPINA1 Z allele (OR, 3.42; 95% CI, 1.54-7.59; P = 1.4 x 10(-3)) but not with TGFB1 codon 10. A combined analysis of the initial and replication studies by logistic regression showed CFLD to be associated with SERPINA1 Z allele (OR, 5.04; 95% CI, 2.88-8.83; P = 1.5 x 10(-8)). CONCLUSIONS: The SERPINA1 Z allele is a risk factor for liver disease in CF. Patients who carry the Z allele are at greater risk (OR, approximately 5) of developing severe liver disease with portal hypertension. |
| 20585107 | Meta-analysis of genome-wide gene expression differences in onset and maintenance phases of genetic hypertension.Gene expression differences accompany both the onset and established phases of hypertension. By an integrated genome-transcriptome approach we performed a meta-analysis of data from 74 microarray experiments available on public databases to identify genes with altered expression in the kidney, adrenal, heart, and artery of spontaneously hypertensive and Lyon hypertensive rats. To identify genes responsible for the onset of hypertension we used a statistical approach that sought to eliminate expression differences that occur during maturation unrelated to hypertension. Based on this adjusted fold-difference statistic, we found 36 genes for which the expression differed between the prehypertensive phase and established hypertension. Genes having possible relevance to hypertension onset included Actn2, Ankrd1, ApoE, Cd36, Csrp3, Me1, Myl3, Nppa, Nppb, Pln, Postn, Spp1, Slc21a4, Slc22a2, Thbs4, and Tnni3. In established hypertension 102 genes exhibited altered expression after Bonferroni correction (P<0.05). These included Atp5o, Ech1, Fabp3, Gnb3, Ldhb, Myh6, Lpl, Pkkaca, Vegfb, Vcam1, and reduced nicotinamide-adenine dinucleotide dehydrogenases. Among the genes identified, there was an overrepresentation of gene ontology terms involved in energy production, fatty acid and lipid metabolism, oxidation, and transport. These could contribute to increases in reactive oxygen species. Our meta-analysis has revealed many new genes for which the expression is altered in hypertension, so pointing to novel potential causative, maintenance, and responsive mechanisms and pathways. |
| 22396741 | Common variants of the liver fatty acid binding protein gene influence the risk of type 2 diabetes and insulin resistance in Spanish population.SUMMARY: The main objective was to evaluate the association between SNPs and haplotypes of the FABP1-4 genes and type 2 diabetes, as well as its interaction with fat intake, in one general Spanish population. The association was replicated in a second population in which HOMA index was also evaluated. METHODS: 1217 unrelated individuals were selected from a population-based study [Hortega study: 605 women; mean age 54 y; 7.8% with type 2 diabetes]. The replication population included 805 subjects from Segovia, a neighboring region of Spain (446 females; mean age 52 y; 10.3% with type 2 diabetes). DM2 mellitus was defined in a similar way in both studies. Fifteen SNPs previously associated with metabolic traits or with potential influence in the gene expression within the FABP1-4 genes were genotyped with SNPlex and tested. Age, sex and BMI were used as covariates in the logistic regression model. RESULTS: One polymorphism (rs2197076) and two haplotypes of the FABP-1 showed a strong association with the risk of DM2 in the original population. This association was further confirmed in the second population as well as in the pooled sample. None of the other analyzed variants in FABP2, FABP3 and FABP4 genes were associated. There was not a formal interaction between rs2197076 and fat intake. A significant association between the rs2197076 and the haplotypes of the FABP1 and HOMA-IR was also present in the replication population. CONCLUSIONS: The study supports the role of common variants of the FABP-1 gene in the development of type 2 diabetes in Caucasians. |
| 11423496 | Association of the Ala54-Thr polymorphism in the intestinal fatty acid-binding protein with 2-h postchallenge insulin levels in the Framingham Offspring Study.OBJECTIVE: To investigate the association of variants of the intestinal fatty acid-binding protein gene (FABP2) with fasting and postchallenge glucose and insulin levels, HbA(1c), and prevalence of type 2 diabetes in a separate sample of men and women. RESEARCH DESIGN AND METHODS: Subjects were participants in the Framingham Offspring Study, a long-term community-based prospective observational study of risk factors for cardiovascular disease. The study sample consisted of 762 men and 922 women. RESULTS: In women, carriers of the Thr54 allele had significantly higher 2-h postchallenge insulin levels than noncarriers (104.4 +/- 73.0 vs. 93.4 +/- 61.5 microU/ml; P = 0.0139). This relationship remained significant after adjustment for familial relationship, age, BMI, triglycerides, APOE genotype, smoking, alcohol intake, the use of beta-blockers, menopausal status, and estrogen therapy. No such significant association was observed in men. In both men and women, there were no statistical associations between the FABP2 polymorphism and BMI, fasting glucose, fasting insulin, 2-h postchallenge glucose levels, HbA(1c), and prevalence of type 2 diabetes. CONCLUSIONS: These results suggest that the FABP2 Thr54 allele may have a minor contribution to the insulin resistance syndrome in a white general population. |
| 16396963 | Intrinsic gluconeogenesis is enhanced in renal proximal tubules of Zucker diabetic fatty rats.Recent studies indicate that renal gluconeogenesis is substantially stimulated in patients with type 2 diabetes, but the mechanism that is responsible for such stimulation remains unknown. Therefore, this study tested the hypothesis that renal gluconeogenesis is intrinsically elevated in the Zucker diabetic fatty rat, which is considered to be an excellent model of type 2 diabetes. For this, isolated renal proximal tubules from diabetic rats and from their lean nondiabetic littermates were incubated in the presence of physiologic gluconeogenic precursors. Although there was no increase in substrate removal and despite a reduced cellular ATP level, a marked stimulation of gluconeogenesis was observed in diabetic relative to nondiabetic rats, with near-physiologic concentrations of lactate (38%), glutamine (51%) and glycerol (66%). This stimulation was caused by a change in the fate of the substrate carbon skeletons resulting from an increase in the activities and mRNA levels of the key gluconeogenic enzymes that are common to lactate, glutamine, and glycerol metabolism, i.e., mainly of phosphoenolpyruvate carboxykinase and, to a lesser extent, of glucose-6-phosphatase and fructose-1,6-bisphosphatase. Experimental evidence suggests that glucocorticoids and cAMP were two factors that were responsible for the long-term stimulation of renal gluconeogenesis observed in the diabetic rats. These data provide the first demonstration in an animal model that renal gluconeogenesis is upregulated by a long-term mechanism during type 2 diabetes. Together with the increased renal mass (38%) observed, they lend support to the view so far based only on in vivo studies performed in humans that renal gluconeogenesis may be stimulated by and crucially contribute to the hyperglycemia of type 2 diabetes. |
| 19102712 | Glutathione S-transferase T1- and M1-null genotypes and coronary artery disease risk in patients with Type 2 diabetes mellitus.INTRODUCTION: Since long-term exposure to oxidative stress is strongly implicated in the pathogenesis of diabetic complications, polymorphic genes of detoxifying enzymes must be involved in the development of coronary artery disease (CAD). We assessed the potential glutathione S-transferase (GST) gene-gene (GSTM1(null)-GSTT1(null)) and gene-smoking interactions on the development of CAD in patients with Type 2 diabetes. MATERIALS & METHODS: In a case-only design, we enrolled 231 patients with Type 2 diabetes (147 male, 66.1 +/- 9.7 years) referred to our institute for coronary angiography investigation. CAD was diagnosed if there was over 50% obstruction of one or more major vessels. RESULTS: Coronary angiography revealed significant CAD in 184 patients (80%). Male gender (p < 0.001), smoking habits (p = 0.003) and GSTT1(null) genotype (p = 0.003) were significantly correlated with the increasing extent of the coronary atherosclerosis. Case-only analysis revealed that patients with both M(null)-T(null) genotypes had the highest risk for 3-vessel CAD compared with patients who express both GST genes (odds ratio: 3.1; 95% confidence interval: 1.0-10.3, p = 0.04). A nearly threefold interaction existed between cigarette smoking and M(null)-T(null) genotypes (odds ratio: 2.9, 95% confidence interval: 1.7-7.8, p = 0.03). A significant interaction between M(null)-T(null) genotypes and smoking was also observed on the increasing number of coronary vessels that were diseased (chi(2) = 14.0; p = 0.03). CONCLUSION: These data suggest that polymorphisms in GSTM1 and GSTT1 genes are risk factors for CAD in Type 2 diabetic patients, especially among smokers. These genetic markers may permit the targeting of preventive and early intervention on high-risk patients to reduce their cardiovascular risk. |
| 20135074 | A genetic variant in the gene encoding fibrinogen beta chain predicted development of hypertension in Chinese men.Fibrinogen, a major determinant of blood viscosity, is an acute phase protein associated with cardiovascular disease. We studied the association of hypertension with single nucleotide polymorphisms (SNPs) in the gene encoding the fibrinogen beta chain (FGB). Three tagging SNPs (rs1025154, rs4220 and rs1044291) were selected from the HapMap database on Han Chinese. Genotypes were determined in 1,294 unrelated subjects from the Hong Kong Cardiovascular Risk Factor Prevalence Study cohort. There were 199 hypertensive subjects at baseline. Among 1,095 subjects normotensive at baseline, 178 developed hypertension during a median follow-up period of 6.4 years. Among the three tagging SNPs, rs4220 showed significant association with hypertension at both baseline (odds ratio [OR]=1.49, p=0.004) and at follow-up (OR=1.32, p=0.013). The minor A allele of this SNP was associated with higher plasma fibrinogen level (beta=0.144, p<0.001 at baseline and beta=0.130, p<0.001 at follow-up). Among subjects normotensive at baseline, this SNP was also associated with the development of hypertension in men (OR=1.52, p=0.022), but not in women. The SNP rs4220 in FGB , which leads to the substitution of arginine by lysine at position 448, is independently associated with plasma fibrinogen level and hypertension in Hong Kong Chinese. This suggests a possible causal role of fibrinogen in hypertension development, especially in men. |
| 22232206 | Exercise Engagement as a Moderator of the Effects of APOE Genotype on Amyloid Deposition.OBJECTIVE: APOE epsilon4 status has been associated with greater cortical amyloid deposition, whereas exercise has been associated with less in cognitively normal adults. The primary objective here was to examine whether physical exercise moderates the association between APOE genotype and amyloid deposition in cognitively normal adults. DESIGN: APOE genotyping data and answers to a questionnaire on physical exercise engagement over the last decade were obtained in conjunction with cerebrospinal fluid (CSF) samples and amyloid imaging with carbon 11-labeled Pittsburgh Compound B ([(11)C]PiB) positron emission tomography. Participants were classified as either low or high exercisers based on exercise guidelines of the American Heart Association. SETTING: Knight Alzheimer's Disease Research Center at Washington University, St Louis, Missouri. PARTICIPANTS: A total of 201 cognitively normal adults (135 of whom were women) aged 45 to 88 years were recruited from the Knight Alzheimer's Disease Research Center. Samples of CSF were collected from 165 participants. Amyloid imaging was performed for 163 participants. Results: APOE epsilon4 carriers evidenced higher [(11)C]PiB binding (P<.001) and lower CSF Abeta42 levels (P<.001) than did noncarriers. Our previous findings of higher [(11)C]PiB binding (P=.005) and lower CSF Abeta42 levels (P=.009) in more sedentary individuals were replicated. Most importantly, we observed a novel interaction between APOE status and exercise engagement for [(11)C]PiB binding (P=.008) such that a more sedentary lifestyle was significantly associated with higher [(11)C]PiB binding for epsilon4 carriers (P=.013) but not for noncarriers (P=.20). All findings remained significant after controlling for age; sex; educational level; body mass index; the presence or history of hypertension, diabetes mellitus, heart problems, or depression; and the interval between assessments. CONCLUSION: Collectively, these results suggest that cognitively normal sedentary APOE epsilon4-positive individuals may be at augmented risk for cerebral amyloid deposition. |
| 23026212 | Two single nucleotide polymorphisms in the human nescient helix-loop-helix 2 (NHLH2) gene reduce mRNA stability and DNA binding.Nescient helix-loop-helix-2 (NHLH2) is a basic helix-loop-helix transcription factor, which has been implicated, using mouse knockouts, in adult body weight regulation and fertility. A scan of the known single nucleotide polymorphisms (SNPs) in the NHLH2 gene revealed one in the 3' untranslated region (3'UTR), which lies within an AUUUA RNA stability motif. A second SNP is nonsynonymous within the coding region of NHLH2, and was found in a genome-wide association study for obesity. Both of these SNPs were examined for their effect on NLHL2 by creating mouse mimics and examining mRNA stability, and protein function in mouse hypothalamic cell lines. The 3'UTR SNP causes increased instability and, when the SNP-containing Nhlh2 3'UTR is attached to luciferase mRNA, reduced protein levels in cells. The nonsynonymous SNP at position 83 in the protein changes an alanine residue, conserved in NHLH2 orthologs through the Drosophila sp. to a proline residue. This change affects migration of the protein on an SDS-PAGE gel, and appears to alter secondary structure of the protein, as predicted using in silico methods. These results provide functional information on two rare human SNPs in the NHLH2 gene. One of these has been linked to human obese phenotypes, while the other is present in a relatively high proportion of individuals. Given their effects on NHLH2 protein levels, both SNPs deserve further analysis in whether they are causative and/or additive for human body weight and fertility phenotypes. |
| 23505183 | Retinol binding protein-4 circulating levels were higher in nonalcoholic fatty liver disease vs. histologically normal liver from morbidly obese women.OBJECTIVE: We aimed to analyze the retinol binding protein-4 (RBP4) messenger RNA (mRNA) expression profiles in adipose tissues and liver of morbidly obese (MO) women with or without nonalcoholic fatty liver disease (NAFLD), and to study the relationships with other pro- and anti-inflammatory adipokines in vivo and in vitro. DESIGN AND METHODS: We performed a cross-sectional analysis of subcutaneous adipose tissue (SAT), visceral adipose tissue (VAT) and liver samples from four lean and 45 MO women with or without NAFLD by enzyme-linked immunosorbent assay and real-time reverse transcription-PCR. We also studied RBP4 expression in HepG2 hepatocytes under various inflammatory stimuli. RESULTS: Circulating RBP4 levels were higher in MO women, and specifically, in MO subjects with NAFLD compared with normal liver controls (lean and MO). RBP4 liver expression was higher in nonalcoholic steatohepatitis (NASH)-moderate/severe than in NASHmild. Overall RBP4 gene expression was higher in liver than in adipose tissues. Among them, the higher expression corresponded to SAT. VAT expression was lower in the MO cohort. In HepG2, RBP4 mRNA expression was reduced by tumor necrosis factor (TNF)-alpha and increased by adiponectin treatment. CONCLUSIONS: The results obtained in MO women with NAFLD, brings up the use of RBP4 and other adipokines as a panel of noninvasive molecular biomarkers when NAFLD is suspected. Further studies are needed with other obesity groups. |
| 15012590 | Obesity and diabetes in transgenic mice expressing proSAAS.ProSAAS is a neuroendocrine peptide precursor that potently inhibits prohormone convertase 1 in vitro. To explore the function of proSAAS and its derived peptides, transgenic mice were created which express proSAAS using the beta-actin promoter. The body weight of transgenic mice was normal until approximately 10-12 weeks, and then increased 30-50% over wild-type littermates. Adult transgenic mice had a fat mass approximately twice that of wild-type mice, and fasting blood glucose levels were slightly elevated. In the pituitary, the levels of several fully processed peptides in transgenic mice were not reduced compared with wild-type mice, indicating that the proSAAS transgene did not affect prohormone convertase 1 activity in this tissue. Because the inhibitory potency of proSAAS-derived peptides towards prohormone convertase 1 is much greater in the absence of carboxypeptidase E activity, the proSAAS transgene was also expressed in carboxypeptidase E-deficient Cpe (fat/fat) mice. Although the transgenic mice were born in the expected frequency, 21 of 22 proSAAS transgenic Cpe (fat/fat) mice died between 11 and 26 weeks of age, presumably due to greatly elevated blood glucose. The levels of several pituitary peptides were significantly reduced in the proSAAS transgenic Cpe (fat/fat) mice relative to non-transgenic Cpe (fat/fat) mice, suggesting that the transgene inhibited prohormone convertase 1 in these mice. Taken together, these results are consistent with a role for proSAAS-derived peptides as neuropeptides that influence body weight independently of their function as inhibitors of prohormone convertase 1. |
| 19143970 | Relationship between SP1 polymorphism and osteoporosis in beta-thalassemia major patients.BACKGROUND: beta-Thalassemia is an autosomal recessive disease characterized by defective beta-globin chain production. Osteoporosis is an important cause of morbidity in patients with beta-thalassemia major. The pathogenesis of reduced bone mineral density (BMD) is multifactorial. A range of genetics factors have been implicated in other populations of patients with osteoporosis. Polymorphism at the Sp1 binding site of the collagen type I A1 (COLIA1) gene is thought to be an important factor in the development of osteoporosis. METHODS: Alleles S and s, detected by presence of a G or T nucleotide, respectively in a regulatory site of the COLIA1 gene were investigated in 37 beta-thalassemia major patients with osteoporosis and 92 controls without osteoporosis or osteopenia using polymerase chain reaction-restriction fragment length polymorphism. RESULTS: Fifteen and nine beta-thalassemia major patients displayed SS and Ss genotypes, respectively, whereas 13 were found to have an ss genotype. The mean BMD of the beta-thalassemia major patients with ss genotype was similar to those with the Ss and SS genotypes. In the control group, 77 and 15 subjects had SS and Ss genotypes, respectively, with no ss genotype. Allelic and genotypic distribution in patients were significantly different from controls. CONCLUSION: Determining base substitutions at the Sp1 binding site on the COLIA1 gene in early years may be important in preventing osteoporosis in children with beta-thalassemia major. |
| 20299617 | Onset of obesity in carboxypeptidase E-deficient mice and effect on airway responsiveness and pulmonary responses to ozone.When compared with lean, wild-type mice, obese Cpefat mice, 14 wk of age and older, manifest innate airway hyperresponsiveness (AHR) to intravenous methacholine and enhanced pulmonary inflammation following acute exposure to ozone (O3). The purpose of this study was to examine the onset of these augmented pulmonary responses during the onset of obesity. Thus airway responsiveness and O3-induced pulmonary inflammation and injury were examined in 7- and 10-wk-old Cpefat and age-matched, wild-type, C57BL/6 mice. Compared with age-matched controls, 7- and 10-wk-old Cpefat mice were approximately 25 and 61% heavier, respectively. Airway responsiveness to intravenous methacholine was assessed via forced oscillation in unexposed Cpefat and wild-type mice. The 10- but not 7-wk-old Cpefat mice exhibited innate AHR. O3 exposure (2 ppm for 3 h) increased markers of pulmonary inflammation and injury in the bronchoalveolar lavage fluid of all mice. However, most markers were greater in Cpefat vs. wild-type mice, regardless of age. Serum levels of leptin, a satiety hormone and proinflammatory cytokine, were increased in Cpefat vs. wild-type mice of both age groups, but the serum levels of other systemic inflammatory markers were greater only in 10-wk-old Cpefat vs. wild-type mice. These results demonstrate that a 25% increase in body weight is sufficient to augment pulmonary responses to O3, but innate AHR is not manifest until the mice become much heavier. These results suggest that the mechanistic bases for these responses are different and may develop according to the nature and degree of the chronic systemic inflammation that is present. |
| 20533025 | Associations of APOE gene polymorphisms with bone mineral density and fracture risk: a meta-analysis.To determine the association of the Apolipoprotein E (APOE) E4 gene polymorphism with bone mineral density (BMD) and fractures we conducted a meta-analysis of 17 reports. Despite lower trochanteric and lumbar BMD in APOE4 carriers, there is insufficient evidence to support a consistent association of APOE with bone health. INTRODUCTION: APOE has been studied for its potential role in osteoporosis risk. It is hypothesized that genetic variation at APOE locus, known as E2, E3, and E4, may modulate BMD through its effects on lipoproteins and vitamin K transport. The purpose of this study was to determine the association of the APOE-E4 gene polymorphism with bone-related phenotypes. METHODS: We conducted a meta-analysis that combined newly analyzed individual data from two community-based cohorts, the Framingham Offspring Study (N = 1,495) and the vitamin K clinical trial (N = 377), with 15 other eligible published reports. Bone phenotypes included BMD measurements of the hip (total hip and trochanteric and femoral neck sites) and lumbar spine (from the L2 to L4 vertebrae) and prevalence or incidence of vertebral, hip, and other fractures. RESULTS: In sex-pooled analyses, APOE4 carriers had a 0.018 g/cm(2) lower weighted mean trochanteric BMD than non carriers (p = 0.0002) with no evidence for between-study heterogeneity. A significant association was also detected with lumbar spine BMD (p = 0.006); however, inter-study heterogeneity was observed. Associations with lumbar spine and trochanteric BMD were observed predominantly in women and became less significant in meta-regression (p = 0.055 and 0.01, respectively). There were no consistent associations of APOE4 genotype with BMD at other skeletal sites or with fracture risk. CONCLUSIONS: Based on these findings, there is insufficient evidence to support a strong and consistent association of the APOE genotype with BMD and fracture incidence. |
| 22194859 | Complement C3 deficiency attenuates chronic hypoxia-induced pulmonary hypertension in mice.BACKGROUND: Evidence suggests a role of both innate and adaptive immunity in the development of pulmonary arterial hypertension. The complement system is a key sentry of the innate immune system and bridges innate and adaptive immunity. To date there are no studies addressing a role for the complement system in pulmonary arterial hypertension. METHODOLOGY/PRINCIPAL FINDINGS: Immunofluorescent staining revealed significant C3d deposition in lung sections from IPAH patients and C57Bl6/J wild-type mice exposed to three weeks of chronic hypoxia to induce pulmonary hypertension. Right ventricular systolic pressure and right ventricular hypertrophy were increased in hypoxic vs. normoxic wild-type mice, which were attenuated in C3-/- hypoxic mice. Likewise, pulmonary vascular remodeling was attenuated in the C3-/- mice compared to wild-type mice as determined by the number of muscularized peripheral arterioles and morphometric analysis of vessel wall thickness. The loss of C3 attenuated the increase in interleukin-6 and intracellular adhesion molecule-1 expression in response to chronic hypoxia, but not endothelin-1 levels. In wild-type mice, but not C3-/- mice, chronic hypoxia led to platelet activation as assessed by bleeding time, and flow cytometry of platelets to determine cell surface P-selectin expression. In addition, tissue factor expression and fibrin deposition were increased in the lungs of WT mice in response to chronic hypoxia. These pro-thrombotic effects of hypoxia were abrogated in C3-/- mice. CONCLUSIONS: Herein, we provide compelling genetic evidence that the complement system plays a pathophysiologic role in the development of PAH in mice, promoting pulmonary vascular remodeling and a pro-thrombotic phenotype. In addition we demonstrate C3d deposition in IPAH patients suggesting that complement activation plays a role in the development of PAH in humans. |
| 17717598 | Discovery of common human genetic variants of GTP cyclohydrolase 1 (GCH1) governing nitric oxide, autonomic activity, and cardiovascular risk.GTP cyclohydrolase 1 (GCH1) is rate limiting in the provision of the cofactor tetrahydrobiopterin for biosynthesis of catecholamines and NO. We asked whether common genetic variation at GCH1 alters transmitter synthesis and predisposes to disease. Here we undertook a systematic search for polymorphisms in GCH1, then tested variants' contributions to NO and catecholamine release as well as autonomic function in twin pairs. Renal NO and neopterin excretions were significantly heritable, as were baroreceptor coupling (heart rate response to BP fluctuation) and pulse interval (1/heart rate). Common GCH1 variant C+243T in the 3'-untranslated region (3'-UTRs) predicted NO excretion, as well as autonomic traits: baroreceptor coupling, maximum pulse interval, and pulse interval variability, though not catecholamine secretion. In individuals with the most extreme BP values in the population, C+243T affected both diastolic and systolic BP, principally in females. In functional studies, C+243T decreased reporter expression in transfected 3'-UTRs plasmids. We conclude that human NO secretion traits are heritable, displaying joint genetic determination with autonomic activity by functional polymorphism at GCH1. Our results document novel pathophysiological links between a key biosynthetic locus and NO metabolism and suggest new strategies for approaching the mechanism, diagnosis, and treatment of risk predictors for cardiovascular diseases such as hypertension. |
| 18178156 | Complement 3 activates the KLF5 gene in rat vascular smooth muscle cells.We have shown that spontaneously hypertensive rat (SHR)-derived vascular smooth muscle cells (VSMCs) change to the synthetic phenotype and show increased expression of complement 3 (C3) and that C3 plays a role in the change to the synthetic phenotype. To determine the mechanisms underlying the effects of C3 on this phenotypic change, we examined the effects of C3a on transcription factors involved in VSMC phenotype and found that C3a increased the expression of Kruppel-like zinc-finger transcription factor 5 (KLF5) mRNA. C3a increased KLF5 promoter activity in a concentration-dependent manner. Deletion analysis of the promoter region of the KLF5 gene revealed that the region between nucleotides-991 and -699 contains the transcriptional regulatory element stimulated by C3a. C3a induced extracellular signal-regulated kinase (ERK) phosphorylation, and C3a-increased KLF5 promoter activity was completely inhibited by the MEK inhibitor U0126. These findings suggest that C3 increases KLF5 promoter activity and gene expression via ERK signaling. |
| 18645210 | Regulatory effects of arachidonate 5-lipoxygenase on hepatic microsomal TG transfer protein activity and VLDL-triglyceride and apoB secretion in obese mice.As 5-lipoxygenase (5-LO) is an emerging target in obesity and insulin resistance, we have investigated whether this arachidonate pathway is also implicated in the progression of obesity-related fatty liver disease. Our results show that 5-LO activity and 5-LO-derived product levels are significantly elevated in the liver of obese ob/ob mice with respect to wild-type controls. Treatment of ob/ob mice with a selective 5-LO inhibitor exerted a remarkable protection from hepatic steatosis as revealed by decreased oil red-O staining and reduced hepatic triglyceride (TG) concentrations. In addition, 5-LO inhibition in ob/ob mice downregulated genes involved in hepatic fatty acid uptake (i.e., L-FABP and FAT/CD36) and normalized peroxisome proliferator-activated receptor alpha (PPARalpha) and acyl-CoA oxidase expression, whereas the expression of lipogenic genes [i.e., fatty acid synthase (FASN) and SREBP-1c] remained unaltered. Furthermore, 5-LO inhibition restored hepatic microsomal TG transfer protein (MTP) activity in parallel with a stimulation of hepatic VLDL-TG and apoB secretion in ob/ob mice. Consistent with these findings, 5-LO products directly inhibited MTP activity and triggered cytosolic TG accumulation in CC-1 cells, a murine hepatocyte cell line. Taken together, these findings identify a novel steatogenic role for 5-LO in the liver through mechanisms involving the regulation of hepatic MTP activity and VLDL-TG and apoB secretion. |
| 19156719 | Association between functional FABP2 promoter haplotypes and body mass index: analyses of 8072 participants of the KORA cohort study.Studies in relatively small cohorts provide preliminary evidence that functional fatty acid binding protein 2 (FABP2) promoter haplotypes are associated with type 2 diabetes and BMI. Here, we studied the influence of the haplotypes on BMI by using 8072 male and female participants of the Kooperative Gesundheitsforschung in der Region Augsburg (KORA) cohort. By linear regression analysis, we found in males a reduction of -0.39 BMI units (95% CI: -0.73, -0.05, p = 0.024) in homozygous FABP2 promoter haplotype B carriers. Carriers of haplotype B showed a significant decrease in BMI of -0.19 BMI units (95% CI: -0.35, -0.02, p = 0.027). In accordance, a significant reduction in BMI of the minor haplotype carriers in the BMI point categories of 25-30 (BMI units: -0.10, 95% CI: -0.18, -0.01, p = 0.03) and < 30 (BMI units: -0.37, 95% CI: -0.67, -0.07, p = 0.02) were found. In summary, the minor FABP2 promoter haplotype B contributes to a reduced BMI in men. This provides evidence that functional FABP2 contributes to multifactorialy regulated body weight. |
| 19450584 | Apolipoprotein E limits oxidative stress-induced cell dysfunctions in human adipocytes.Oxidative stress in adipose tissue constitutes a pathological process involved in obesity-linked metabolic disorders. Apolipoprotein E (apoE), which exhibits antioxidant properties in plasma and brain, is highly produced by adipose tissue and adipocytes. In this study, we investigated the role of apoE in the human adipocyte response to oxidative stress. We first demonstrated that apoE secretion by adipocytes was stimulated by oxidative stress. We also observed that apoE overexpression protected adipocytes from hydrogen peroxide-induced damages, by mitigating intracellular oxidation and exerting extracellular antioxidant properties. Our findings clearly show a novel antioxidant role for apoE in adipose tissue. |
| 19628211 | Apolipoprotein E genotype predicts cardiovascular endpoints in dialysis patients with type 2 diabetes mellitus.OBJECTIVE: Cardiovascular disease (CVD) is the leading cause of death in patients with type 2 diabetes (T2DM) and end-stage renal disease (ESRD). Lipid metabolism is influenced by environmental and genetic factors. Among the latter, the apolipoprotein E (apoE) genotype is known to be associated with CVD risk and thus may affect cardiovascular outcome. METHODS AND RESULTS: Based on the German Diabetes and Dialysis Study evaluating 1255 T2DM patients on haemodialysis (HD) (median follow-up 4 years), the impact of the apoE genotype (available for 1177 patients) on pre-specified, centrally adjudicated endpoints was investigated: all-cause mortality (n=558), combined cardiovascular events (CVE: cardiac death, MI, stroke; n=442), and cardiac death (n=218). Patients with at least one epsilon4 allele (epsilon4+) showed a 30% increased risk for CVE (HR 1.299, 95%CI 1.045-1.615, p=0.018) and a 36% increased risk for cardiac death (HR 1.362, 95%CI 1.002-1.852, p=0.048) compared to patients with no epsilon4 allele. Consistently, addition of epsilon4+ to a multivariate ROC model for risk prediction of CVE including atorvastatin treatment, history of cardiovascular disease, dialysis and lipoprotein parameters, hsCRP, and NT-pro-BNP increased the area under the curve from 0.666 (95%CI 0.634-0.698) to 0.671 (95%CI 0.639-0.702), p=0.013. CONCLUSIONS: The presence of the epsilon4 allele increases the risk for CVE and cardiac death in patients with T2DM and ESRD. Whether treatment strategies guided by apoE genotype will improve outcome needs to be evaluated in the future. |
| 23213264 | Cognitive function in an elderly population: interaction between vitamin B12 status, depression, and apolipoprotein E epsilon4: the Hordaland Homocysteine Study.OBJECTIVE: To investigate the cross-sectional relation between metabolic markers of vitamin B(12) status and cognitive performance, and possible effect modification by the presence of depression and apolipoprotein E (ApoE) epsilon4. METHODS: This is a population-based study of 1935 participants, aged 71 to 74 years, from Norway. Participants were administered a cognitive test battery, and vitamin B(12) status was assessed by measurements of plasma vitamin B(12), holotranscobalamin (holoTC), methylmalonic acid (MMA), and total homocysteine. RESULTS: The geometric mean (95% confidence interval) for vitamin B(12) was 348 pM (341-354), whereas 5.9% of participants had vitamin B(12) levels lower than 200 pM. In linear regression analyses, holoTC (p = .039) and the holoTC/vitamin B(12) ratio (p = .013) were positively related, whereas MMA (p = .010) was inversely related, to global cognition, after adjustment for sex, education, ApoE status, plasma creatinine, and history of diabetes, cardiovascular disease, hypertension, and depression. Among those positive for ApoE epsilon4, but not among those without the epsilon4 allele, plasma vitamin B(12) was positively associated with global cognition (p = .015), whereas MMA was inversely related to global cognition (p = .036) and executive function (p = .014). In participants with depression, MMA was inversely associated with global cognition (p < .001) and episodic memory (p = .001). CONCLUSIONS: Among the well-nourished elderly, low vitamin B(12) status is associated with cognitive deficit, particularly in those with the ApoE epsilon4 allele or with depression. |
| 8770919 | Impaired processing of brain proneurotensin and promelanin-concentrating hormone in obese fat/fat mice.Mice homozygous for the fat mutation exhibit marked hyperpro-insulinemia and develop late onset obesity. The fat mutation was recently mapped to the gene encoding carboxypeptidase E (CpE), a processing enzyme involved in trimming C-terminal paired basic residues from prohormone-derived peptides. The mutation resulted in a loss of CpE activity that correlated with aberrant proinsulin processing. Neurotensin (NT) and melanin-concentrating hormone (MCH) are two neuropeptides that, among other central effects, inhibit food intake. Here, using RIA techniques coupled to reverse phase HPLC, we analyzed the processing products derived from the NT and MCH precursors in the brain of +/fat and fat/fat mice. Compared to control hypothalamic and brain extracts, fat/fat extracts had markedly reduced levels (>80%) of NT and neuromedin N (NN), another active pro-NT-derived peptide. In contrast, they exhibited high concentrations of biologically inactive NT-KR and NN-KR (NT and NN with a C-terminal Lys-Arg extension), two peptides that were undetectable in control extracts. MCH, which is located at the C-terminus of its precursor, was present in 2- to 3-fold higher amounts in fat/fat than in +/fat hypothalamus. Neuropeptide-Glu-Ile, another pro-MCH-derived neuropeptide separated from MCH by an Arg-Arg sequence, was present in amounts similar to those of MCH in control extracts. In contrast, neuropeptide-Glu-Ile was more than 10 times less abundant than MCH in extracts from obese mice. Our data are consistent with a deficit in CpE activity affecting the maturation of both pro-NT and pro-MCH. This suggests that abnormal neuropeptide and hormone precursor processing is a general phenomenon in fat/fat mice and supports the idea that defects in the production of neuropeptide involved in the control of feeding might lead to the development of obesity in these animals. |
| 11719374 | Regulation of tissue factor gene expression in obesity.Altered expression of proteins of the fibrinolytic and coagulation cascades in obesity may contribute to the cardiovascular risk associated with this condition. In spite of this, the zymogenic nature of some of the molecules and the presence of variable amounts of activators, inhibitors, and cofactors that alter their activity have made it difficult to accurately monitor changes in the activities of these proteins in tissues where they are synthesized. Thus, as a first approach to determine whether tissue factor (TF) expression is altered in obesity, this study examined changes in TF mRNA in various tissues from lean and obese (ob/ob and db/db) mice. TF gene expression was elevated in the brain, lung, kidney, heart, liver, and adipose tissues of both ob/ob and db/db mice compared with their lean counterparts. In situ hybridization analysis indicated that TF mRNA was elevated in bronchial epithelial cells in the lung, in myocytes in the heart, and in adventitial cells lining the arteries including the aortic wall. Obesity is associated with insulin resistance and hyperinsulinemia, and administration of insulin to lean mice induced TF mRNA in the kidney, brain, lung, and adipose tissue. These observations suggest that the hyperinsulinemia associated with insulin-resistant states, such as obesity and noninsulin-dependent diabetes mellitus, may induce local TF gene expression in multiple tissues. The elevated TF may contribute to the increased risk of atherothrombotic disease that accompanies these conditions. |
| 14517226 | Endothelin-1 stimulates arterial VCAM-1 expression via NADPH oxidase-derived superoxide in mineralocorticoid hypertension.Although hypertension is a major risk factor for atherosclerosis, its underlying mechanisms remain to be delineated. We have recently reported that both endothelin-1 (ET-1) and vascular cellular adhesion molecule-1 (VCAM-1) levels, key early markers of atherosclerosis, are significantly elevated in carotid arteries of deoxycorticosterone acetate (DOCA)-salt hypertensive rats, a model known for its suppressed plasma renin levels. This study tested the hypothesis that ET-1 augments arterial VCAM-1 expression through NADPH oxidase-derived superoxide (O2-). Carotid arteries of DOCA-salt or sham-operated rats were transduced ex vivo with extracellular superoxide dismutase (EC-SOD), dominant negative HA-tagged N17Rac1 that inhibits Rac1, the small GTPase component of NADPH oxidase, or beta-galactosidase (beta-gal) reporter gene (5x10(10) plaque formation units [pfu]/mL), and the effect of transgene expression on O2- and VCAM-1 levels was assayed 24 hours afterward. The arterial activity of NADPH oxidase but not xanthine oxidase was significantly higher in DOCA-salt than in sham rats, which was abolished by the selective ETA receptor antagonist ABT-627 (3x10(-8) mol/L), NADPH oxidase inhibitor apocynin (10(-4) mol/L), or dominant negative Rac1 gene transfer. The levels of O2- and VCAM-1 were significantly increased in arteries of DOCA-salt rats, an effect that was ameliorated after EC-SOD or dominant negative Rac1 but not beta-gal reporter gene transfer. ABT-627 and apocynin also significantly reduced elevated VCAM-1 levels in ET-1-treated arteries of normal rats and arteries of DOCA-salt rats. The results of this study indicate that ET-1 stimulates arterial VCAM-1 expression by producing O2- from an ETA receptor/NADPH oxidase pathway in low-renin mineralocorticoid hypertension. |
| 17173705 | Severe combined hyperlipidaemia and retinal lipid infiltration in a patient with Type 2 diabetes mellitus.Severe combined hyperlipidaemia has occasionally been associated with infiltration of tissues in addition to arteries and the skin. We report a woman with Type 2 diabetes mellitus (DM) and severe combined hyperlipidaemia who developed retinal lipid infiltration, resulting in blindness. A 61-year-old woman with a 15-year history of Type 2 DM was admitted following a two-week history of progressive visual loss. Examination identified lipid infiltration into the retina. Phenotypically she had severe combined hyperlipidaemia with elevated IDL cholesterol and a broad beta band on lipoprotein electrophoresis, raising the possibility of familial dysbetalipoproteinaemia. However, gene sequencing analysis indicated that the patient was homozygous for the E3/E3 allele of the ApoE gene with no mutations detected in either the coding region or intron-exon boundaries. Her lipid profile improved following dietary therapy and gemfibrozil treatment, but this had little effect on either her fundal appearances or her visual acuity. Type 2 DM plays a vital role both in allowing expression of severe combined hyperlipoproteinaemia, in addition to serving as a risk factor for complications such as tissue infiltration. |
| 17897305 | Risk of venous thrombosis: obesity and its joint effect with oral contraceptive use and prothrombotic mutations.In the Multiple Environmental and Genetic Assessment of risk factors for venous thrombosis (MEGA study), body weight, height and body mass index (BMI) were evaluated as risk factors. Additionally, the joint effect of obesity together with oral contraceptive use and prothrombotic mutations on the risk of venous thrombosis were analysed. Three-thousand eight-hundred and thirty-four patients with a first venous thrombosis and 4683 control subjects were included, all non-pregnant and without active malignancies. Relative to those with a normal BMI (<25 kg/m(2)), overweight (BMI > or = 25 and BMI < 30 kg/m(2)) increased the risk of venous thrombosis 1.7-fold [odds ratio (OR)(adj(age and sex)) 1.70, 95% confidence interval (CI) 1.55-1.87] and obesity (BMI > or = 30 kg/m(2)) 2.4-fold (OR(adj) 2.44, 95% CI 2.15-2.78). An increase in body weight and body height also individually increased thrombotic risk. Obese women who used oral contraceptives had a 24-fold higher thrombotic risk (OR(adj) 23.78, 95% CI 13.35-42.34) than women with a normal BMI who did not use oral contraceptives. Relative to non-carriers of normal BMI, the joint effect of factor V Leiden and obesity led to a 7.9-fold increased risk (OR(adj) 7.86, 95% CI 4.70-13.15); for prothrombin 20210A this was a 6.6-fold increased risk (OR(adj) 6.58, 95% CI 2.31-18.69). Body height, weight and obesity increase the risk of venous thrombosis, especially obesity in women using oral contraceptives. |
| 18477980 | [The role of angiotensin-converting enzyme and apolipoprotein E in the development of intracranial aneurysms.]The distribution of allele and genotype frequencies of the Alu-insertion polymorphism of the angiotensin-converting enzyme (ACE) gene and missence mutations leading to the substitution of arginine to cysteine in positions 112 and 158 of apolipoprotein E (APOE) has been studied in 166 patients with brain intracranial aneurysms and in 192 controls of Russian origin from Ural region. Brain vascular aneurysms with hypertension were associated with the D\*D\* ACE genotype in men and with the e2 allele and the e2/e3 APOE genotype in women. The association was also observed between the e2 allele and the e2/e3 APOE genotype and family history of stroke, hemorrhages and aneurysms in patients. Men with the I\*D\* ACE genotype and the e4 APOE allele were at lower risk. |
| 20709332 | Telomere length and ApoE polymorphism in mild cognitive impairment, degenerative and vascular dementia.BACKGROUND: Clarifying the aetiology of dementia is of crucial importance in the management of patients as well as for research purposes but it is not always possible clinically. Therefore the identification of biological markers should complement clinical approaches. Telomere shortening is emerging as an important mechanism in vascular aging and the pathogenesis of hypertension and atherosclerosis. Thus, telomere length could be a potential candidate to accurately separate vascular from degenerative cognitive impairment. OBJECTIVES: To evaluate the usefulness of telomere length alone or combined with ApoE polymorphism in diagnosing mild cognitive impairment (MCI) and in differentiating Alzheimer's disease (AD) from vascular (VaD) and mixed dementia (MD). METHODS: Telomere length in peripheral blood lymphocytes was performed by flow cytometry in 439 patients (mean age, 85.1 years): 204 cognitively normal, 187 demented patients: 80 AD, 86 MD, and 21 with VaD; and 48 patients with MCI. Simple and multiple ordered logistic regressions were used to predict the risk of dementia from telomere length, ApoE polymorphism and age. RESULTS: ApoEepsilon4 was statistically associated with patients with dementia (p<0.001) compared to cognitively normal or MCI patients; but not with the aetiologies of dementia (AD, VaD and MD) (p=0.385). No significant differences in telomere length were found among patients with different aetiologies or severities of dementia. In the global model, the combination of telomere length and ApoE polymorphism did not confer a significantly higher dementia risk (OR=0.95, 95% CI=0.69-1.32; p=0.784) than APOEepsilon4 alone (OR=2.12, 95% CI=1.15-3.9; p=0.016). CONCLUSION: This longitudinal study in very old patients provided no evidence suggesting that telomere length alone could be used to distinguish between the different types of dementia or MCI, nor combined with the ApoE polymorphism. |
| 11156623 | Genetic analysis of case/control data using estimated haplotype frequencies: application to APOE locus variation and Alzheimer's disease.There is growing debate over the utility of multiple locus association analyses in the identification of genomic regions harboring sequence variants that influence common complex traits such as hypertension and diabetes. Much of this debate concerns the manner in which one can use the genotypic information from individuals gathered in simple sampling frameworks, such as the case/control designs, to actually assess the association between alleles in a particular genomic region and a trait. In this paper we describe methods for testing associations between estimated haplotype frequencies derived from multilocus genotype data and disease endpoints assuming a simple case/control sampling design. These proposed methods overcome the lack of phase information usually associated with samples of unrelated individuals and provide a comprehensive way of assessing the relationship between sequence or multiple-site variation and traits and diseases within populations. We applied the proposed methods in a study of the relationship between polymorphisms within the APOE gene region and Alzheimer's disease. Cases and controls for this study were collected from the United States and France. Our results confirm the known association between the APOE locus and Alzheimer's disease, even when the epsilon 4 polymorphism is not contained in the tested haplotypes. This suggests that, in certain situations, haplotype information and linkage disequilibrium-induced associations between polymorphic loci that neighbor loci harboring functional sequence variants can be exploited to identify disease-predisposing alleles in large, freely mixing populations via estimated haplotype frequency methods. |
| 12077730 | Effects of apolipoprotein E genotype on dietary-induced changes in high-density lipoprotein cholesterol in obese postmenopausal women.Lipid responses to a dietary intervention are highly variable between individuals. Part of this variation may be accounted for by individual differences in lipid-regulating genes that interact with diet to induce changes in lipoprotein metabolism. This study determined whether apolipoprotein E (APOE) genotype affects lipid responses to a low-fat, low-cholesterol diet in obese, postmenopausal women. Body weight and lipoprotein lipid responses to a 10-week, dietary intervention (American Heart Association [AHA] Step I) were compared in 61 women with the APOE 2/3 and APOE 3/3 genotype (APOE4-) and 18 women with the APOE 3/4 genotype (APOE4+) of a similar age, body composition, and maximal aerobic capacity. Body weight decreased by 2% in both groups, but changes in body weight correlated only with changes in low-density lipoprotein-cholesterol (LDL-C) (r =.27, P <.05). The dietary intervention decreased total cholesterol and LDL-C to a similar degree in both genotype groups. However, APOE4- women decreased high-density lipoprotein-cholesterol (HDL-C) by 17% +/- 11% and increased triglycerides by 20% +/- 41% in response to the diet, while APOE4+ women had a smaller decrease in HDL-C (-8% +/- 12%) and no change in plasma triglyceride. These group differences were significant for HDL-C (P <.01) and approached significance for triglycerides (P =.08). Moreover, APOE4- women decreased HDL(2)-C by 32% +/- 45%, while APOE4+ women increased HDL(2)-C by 12% +/- 62% (P <.01 between groups). It may be prudent to genotype older women before initiating low-fat diet therapy, as those with the APOE4 allele benefit the most, while the lipid profile could worsen in women without the APOE4 allele. |
| 16002819 | The Ala54Thr polymorphism of the fatty acid-binding protein 2 gene is associated with a change in insulin sensitivity after a change in the type of dietary fat.BACKGROUND: Insulin resistance, a condition associated with type 2 diabetes, results from the interaction of environmental and genetic factors. OBJECTIVE: We examined the influence of the intestinal fatty acid-binding protein 2 (FABP2) Ala54Thr polymorphism on insulin sensitivity. DESIGN: Fifty-nine healthy young subjects (28 were Ala54/Ala54, 27 were Ala54/Thr54, and 4 were Thr54/Thr54) completed 3 diets, each of which lasted 4 wk. The first diet, which all subjects consumed, was a high-saturated fatty acid (SFA) diet (38% of energy as fat and 20% of energy as SFAs). The second and third diets were administered according to a randomized crossover design, and they consisted of a low-fat and high-carbohydrate diet (CHO diet; 28% of energy from fat and <10% of energy from SFAs) and a high-monounsaturated fatty acid (MUFA) diet (called the Mediterranean diet; 38% of energy from fat and 22% of energy from MUFAs). All food and drinks were prepared and provided in the research kitchen. We determined in vivo insulin resistance by using the insulin suppression test with somatostatin. RESULTS: Steady state plasma glucose concentrations were significantly higher in Ala54Thr subjects after the SFA diet than after the CHO diet or the Mediterranean diet. The plasma free fatty acid concentrations in these subjects were significantly lower after the CHO and Mediterranean diets than after the SFA diet. However, no significant differences between the 3 diets were observed in the Ala54 allele homozygotes. CONCLUSION: Insulin sensitivity decreased in subjects with the Thr54 allele of the FABP2 polymorphism when SFAs were replaced by MUFAs and carbohydrates. |
| 16868749 | Functional and molecular alterations of the glomerular barrier in long-term diabetes in mice.AIMS/HYPOTHESIS: Despite the fact that diabetic nephropathy is an increasingly common disorder that may lead to uraemia, the underlying mechanisms are still poorly understood and there is no specific therapy. To clarify whether long-term diabetes alters glomerular size- or charge-selectivity or both, we studied non-obese diabetic mice for up to 40 weeks. MATERIALS AND METHODS: During the study period, spot urine was collected and blood pressure measured. At weeks 10 and 40, the right kidney was isolated and perfused at 8 degrees C to inhibit tubular function, allowing for analysis of glomerular selectivity with albumin and Ficoll clearance. The left kidney was removed for further investigation using electron microscopy and molecular biology. Real-time PCR with low-density arrays was done to evaluate renal cortex mRNA expression of proteoglycans and other components in the glomerular barrier. After 40 weeks of diabetes, kidneys showed morphological changes typical of diabetic complications. RESULTS: At 40 weeks, the fractional clearance for negatively charged albumin was three times higher in the diabetic animals (0.0160) than in controls (0.0051, p<0.001), while fractional clearance for neutral Ficoll 35.5 A with a Stokes Einstein radius similar to that of albumin was unaffected. In addition, protein and mRNA levels for versican and decorin were downregulated after 40 weeks of diabetes. CONCLUSIONS/INTERPRETATION: We conclude that glomerular charge- but not size-selectivity was impaired in the diabetic animals with proteinuria. Also, glomerular components such as versican, decorin and fibromodulin were found to be downregulated after 40 weeks of diabetes. |
| 18815487 | Nhlh2: a basic helix-loop-helix transcription factor controlling physical activity.In mice, targeted deletion of the basic helix-loop-helix transcription factor, nescient helix-loop-helix 2 (Nhlh2), leads to adult-onset obesity and reduced physical activity. We propose the novel hypothesis that transcriptional activity by Nhlh2 (NHLH2 in humans) controls either the ability or the motivation for exercise. |
| 19187685 | [Influence of the APOE genotypes in some atherosclerotic risk factors].The aim of this work was to study the distribution of apolipoprotein E (APOE) genotypes and their association with some atherosclerotic risk factors, all of them modifiable: total, HDL and LDL cholesterol, triglycerides, systolic and diastolic blood pressure, BMI, waist circumference and smoking. The sample population was constituted of 672 healthy subjects recruited in the Lisbon area. Lipids were quantified by usual automatic enzymatic methods and the APOE genotypes performed in accordance with Hixson and Vernier. Blood pressure measurement and hypertension classification followed international specifications. The frequency distribution of APOE alleles was: epsilon2 = 6.4%, epsilon3 = 83.6% and epsilon4 = 10.0% and the more prevalent genotypes were epsilon2/epsilon3, epsilon3/epsilon3 and epsilon3/epsilon4 respectively 11.0%, 70.1% and 16.1%. We could only observe associations among the most prevalent genotypes and lipids, always statistically significant, specially when the epsilon4 allele was present which was even proved by an higher prevalence of epsilon4 in dyslipidemic subjects with the only exception of those with low HDL-c values. A stronger intervention in the epsilon4 carriers is so recommended through appropriate intervention measures on the connected modifiable risk factors. |
| 20808139 | The relationship of cardiovascular risk factors to Alzheimer disease in Choctaw Indians.OBJECTIVES: To test the hypothesis that cardiovascular risk factors (CRFs) influence predisposition to and the clinical course of Alzheimer disease (AD), the authors compared Choctaw Indians, a group with known high CRF with white persons with AD. In addition to CRF history, the authors investigated the frequency of apolipoprotein E4 (apoE4) genotype andplasma homocysteine (HC) levels. METHOD: The authors compared 39 Choctaw Indians with AD and 39 Choctaw Indians without AD to 39 white persons with AD with all groups similar in age. CRF history included diabetes, hypertension, high cholesterol or hypolipidemic agent use, or myocardial infarction. The authors also compared plasma HC concentration and apoE4 allele frequency. RESULTS: Choctaw persons with AD differed significantly from white persons with AD in history of hypertension, diabetes, and in HC values but not from Indians without AD. There was a significantly lower apoE4 allele frequency in Choctaw Indian AD than white persons with AD, and both AD groups had an affected first degree relative significantly more often than Indian controls. There was no relationship between the number of CRF and age at onset among Indians or whites, whereas HC concentration was associated with significantly earlier age of onset for Choctaw Indians but not for whites. CONCLUSIONS: This small study suggests that in Choctaw Indians modifiable risk factors may play more of a role in disease pathogenesis than in whites and that nonmodifiable risk factors such as apoE4 may play less of a role. |
| 22921891 | Is there any association of apolipoprotein E gene polymorphism with obesity status and lipid profiles? Tehran Lipid and Glucose Study (TLGS).AIMS: Considering the key role played by the apolipoprotein E (Apo E) gene in the regulation of lipid metabolism and obesity, the current study has evaluate the association between abdominal obesity and Apo E gene polymorphism in a population of Tehran. MATERIALS AND METHODS: A cross-sectional study was performed on 345 men and 498 women, aged 19-86 years, selected from among participants of the Tehran Lipid and Glucose Study. The RFLP-PCR technique was employed to investigate polymorphism in the gene fragments. Based on the national survey of risk factors for non-communicable diseases of Iran, waist circumference (WC) cut off was set at 89 cm for men and 91 cm for women. The risk effect of obesity related variables and lipid profiles in two groups of WC were examined by logistic regression. For body mass index (BMI), waist to hip ratio (WHR), high-density lipoprotein-cholesterol (HDL-C), triglyceride (TG), fasting blood sugar (FBS), total cholesterol (TC), low-density lipoprotein-cholesterol (LDL-C), and blood pressure (BP), the standard risk cut-offs were applied. RESULTS: Frequencies of E2, E3, and E4 alleles were 9.7, 73, and 14.6%, respectively. The presence of the E3 allele was significantly associated with higher TG level in subjects with high WC, while, the presence of E4 allele decreased the plasma HDL-C (E2:52.1+/-13.1 vs., E3:48.9+/-11.2 vs., E4:44.6+/-10.6 mg/dl, p<0.05), HDL-C2 (E2:20.4+/-9.2 vs., E3:19.1+/-8.8 vs., E4:16.3+/-7.9 mg/dl, p<0.05), and HDL-C3 (E2:32.1+/-7.4 vs., E3:30.3+/-6.2 vs., E4:28.3+/-6.1 mg/dl, p<0.05) in normal WC subjects. The presence of the E3 carrier increased the risk of having higher plasma TG, compared with the E2 carrier (95% CI OR=1.91, 1.02-3.57; p=0.04). CONCLUSION: According to the results of this study, the E3 carrier, caused an approximately 90% increase in the levels of TG in the group with abdominal obesity. |
| 8786074 | Substitution of glycine-661 by serine in the alpha1(I) and alpha2(I) chains of type I collagen results in different clinical and biochemical phenotypes.We have characterised a point mutation causing the substitution of serine for glycine at position 661 of the alpha1(I) chain of type I collagen in a child with a severe form of osteogenesis imperfecta. An identical glycine substitution in the alpha2(I) chain was previously detected in a woman with post-menopausal osteoporosis. Two of her sons were heterozygous for the mutation and the third son was homozygous as a result of uniparental isodisomy. Biochemical profiles of the type I collagen heterotrimers were studied in each of the patients and compared with a control. Medium and cell-layer collagens were overmodified in all patients. Overmodification was obvious in the patient with the alpha 1(I) mutation but mild in the patients with the alpha 2(I) mutation, being slightly less evident in the heterozygote than in the homozygote. Investigation of the melting curves of the mutant collagen trimers in all three patients showed the same slight decrease in thermal stability and, hence, a lack of correlation with phenotypic severity. In contrast, the degree of overmodification of the collagen alpha chains was correlated with the phenotypic severity. The clinical observations in these patients illustrate the possibly predominant role of mutations in the collagen alpha1(I) chains over the same mutations in the alpha2(I) chains in determining the clinical outcome. |
| 9229117 | The genetics of obesity.Despite the influence of obesity in predisposing to many diseases, and evidence for high heritability, efforts to identify human genes with major effects on bodyweight have not yet been successful. In contrast, remarkable progress has been made in the identification and characterization of the genes mutated in five monogenic mouse models of obesity. These genes have led to new insights into the etiology of obesity and provide promising targets for therapeutic intervention. |
| 11371715 | Effects of parathyroid hormone on bone formation in a rat model for chronic alcohol abuse.BACKGROUND: Alcoholism is a risk factor for osteoporosis and it is not clear whether the detrimental effects of alcohol on bone are reversible. Parathyroid hormone (PTH) is a potent stimulator of bone matrix synthesis and is being investigated as a therapeutic agent to reverse bone loss. The present investigation was designed to determine the effects of PTH on bone formation in a rat model for chronic alcohol abuse. METHODS AND RESULTS: Alcohol was administered in the diet of female rats (35% caloric intake) for 2 weeks. Human (1-34) PTH (80 microg/kg/day) was administered subcutaneously during the second week of the study. Alcohol resulted in a transient reduction in steady-state mRNA levels for the bone matrix proteins type 1 collagen, osteocalcin, and osteonectin compared with rats that were fed an alcohol-free (control) diet. As expected, alcohol decreased and PTH increased histologic indices of bone formation. Additionally, two-way ANOVA demonstrated that alcohol antagonized PTH-induced bone formation. Despite antagonism, bone formation and mRNA levels for bone matrix proteins in alcohol-fed rats treated with PTH greatly exceeded the values in rats fed the control diet. CONCLUSIONS: The results of this study contribute to a growing body of evidence that alcohol-induced bone loss is primarily due to reduced bone formation. We conclude that alcohol does not prevent the stimulatory effects of PTH on bone formation. This is evidence that the effects of alcohol on the skeleton are reversible. Additionally, the positive effects on bone formation in rats that consumed high concentrations of alcohol suggested that PTH may be useful as an intervention to treat alcohol-induced osteoporosis. |
| 11433351 | Apolipoprotein B secretion and atherosclerosis are decreased in mice with phospholipid-transfer protein deficiency.Increased secretion and levels of ApoB-containing lipoproteins (BLp) commonly occur in familial hyperlipidemia, obesity and diabetes. The plasma phospholipid-transfer protein (PLTP) is known to mediate transfer of phospholipids between BLp and HDL during their intravascular metabolism. To address a possible role of PLTP in dyslipidemia and atherogenesis, we bred mice deficient in the gene encoding PLTP (PLTP-deficient mice) using different hyperlipidemic mouse strains. In ApoB-transgenic and ApoE-deficient backgrounds, PLTP deficiency resulted in reduced production and levels of BLp and markedly decreased atherosclerosis. BLp secretion was diminished in hepatocytes from ApoB-transgenic PLTP-deficient mice, a defect that was corrected when PLTP was reintroduced in adenovirus. The studies reveal a major, unexpected role of PLTP in regulating the secretion of BLp and identify PLTP as a therapeutic target. |
| 11955026 | High risk for hyperlipidemia and the metabolic syndrome after an episode of hypertriglyceridemia during 13-cis retinoic acid therapy for acne: a pharmacogenetic study.BACKGROUND: Administration of 13-cis retinoic acid (isotretinoin) for acne is occasionally accompanied by hyperlipidemia. It is not known why some persons develop this side effect. OBJECTIVE: To determine whether isotretinoin triggers a familial susceptibility to hyperlipidemia and the metabolic syndrome. DESIGN: Cross-sectional comparison. SETTING: University hospital in Lausanne, Switzerland. PARTICIPANTS: 102 persons in whom triglyceride levels increased at least 1.0 mmol/L (> or =89 mg/dL) (hyperresponders) and 100 persons in whom triglyceride levels changed 0.1 mmol/L (< or =9 mg/dL) or less (nonresponders) during isotretinoin therapy for acne. Parents of 71 hyperresponders and 60 nonresponders were also evaluated. MEASUREMENTS: Waist-to-hip ratio; fasting glucose, insulin, and lipid levels; and apoE genotype. RESULTS: Hyperresponders and nonresponders had similar pretreatment body weight and plasma lipid levels. When reevaluated approximately 4 years after completion of isotretinoin therapy, hyperresponders were more likely to have hypertriglyceridemia (triglyceride level > 2.0 mmol/L [>177 mg/dL]; odds ratio [OR], 4.8 [95% CI, 1.6 to 13.8]), hypercholesterolemia (cholesterol level > 6.5 mmol/L [>252 mg/dL]; OR, 9.1 [CI, 1.9 to 43]), truncal obesity (waist-to-hip ratio > 0.90 [OR, 11.0 (CI, 2.0 to 59]), and hyperinsulinemia (insulin-glucose ratio > 7.2; OR, 3.0 [CI, 1.6 to 5.7]). In addition, more hyperresponders had at least one parent with hypertriglyceridemia (OR, 2.6 [CI, 1.2 to 5.7]) or a ratio of total to high-density lipoprotein cholesterol that exceeded 4.0 (OR, 3.5 [CI, 1.5 to 8.0]). Lipid response to isotretinoin was closely associated with the apoE gene. CONCLUSION: Persons who develop hypertriglyceridemia during isotretinoin therapy for acne, as well as their parents, are at increased risk for future hyperlipidemia and the metabolic syndrome. |
| 15239601 | [Polymorphisms of APO-E in Colombian women with osteoporosis: correlation with clinical and social risk variables].Several studies have reported an association between apolipoprotein E polymorphisms and osteoporosis, specially the genotype APO-E4. In order to analyze the APO-E polymorphisms and to identify their association with clinical and social variables, a descriptive study was undertaken that included 32 women with osteoporosis, from different regions of Colombia. The polymorphisms were detected by PCR and RFLP methods. In osteopenia and osteoporosis combined with osteopenia were observed the genotype epsilon3/epsilon3 in the 84% (n=27), and 16% (epsilon3/epsilon4=12.5%, n=4; epsilon4/epsilon4=3.1%, n=1) for the genotypes bearing the epsilon4 allele. The same tendency was observed by age of the menopause, epsilon3/epsilon3 in the 83% (n=25), and the genotypes bearing the epsilon4 allele in the 17% (n=5) (epsilon3/epsilon4=13.3%, n=4; epsilon4/epsilon4=3.3%, n=1). No association of APO-E4 was detected with socioeconomic stratum, fracture, illness, surgeries, and milk consumption. No significant differences were observed in the bone mineral density (BMD) of the lumbar column between the genotypes with or without the epsilon4 allele epsilon4+/- (epsilon3/epsilon4 0.96+/-0.14 g/cm2); epsilon4+/+ (epsilon4/epsilon4 0.87+/-0.0 g/cm2); epsilon4-/- (epsilon3/epsilon3 0.86+/-0.16 g/cm2); p=0.49, and femoral bone mineral density epsilon4+/- (epsilon3/epsilon4 0.84+/-0.03 g/cm2); epsilon4+/+ (epsilon4/epsilon4 0.84+/-0.0 g/cm2); epsilon4-/- (epsilon3/epsilon3 0.74+/-0.01 g/cm2); p=0.014. However, when exploring the differences of BMD in the femoral neck, a significant difference was observed (t=4.17, p=0.05). These results confirm epsilon4 allele frequencies similar to those reported for caucasian and Japanese, subjects. Larger studies are necessary to elucidate the effect of APO-E in bone marrow and the dose-effect relation. |
| 16332384 | Cholesterol and Alzheimer's disease--is there a relation?The predominating theory on the pathophysiology of Alzheimer's disease (AD) concerns the mis-metabolism of amyloid precursor protein (APP). As a result of this mis-metabolism, there is an increased production of the 42 amino acid form of beta-amyloid (Abeta42) that rapidly will form oligomers that initiates a cascade of events leading to the accumulation of amyloid plaques. Commonly recognised as vascular factors, hypertension, hypercholesterolemia and diabetes and the inheritance of the epsilon4 allele of the APOE gene, are also risk factors for AD. These risks have been found to promote the production of Abeta42. An association between cholesterol and the development of AD was suggested in the early 1990s and ever since, an increasing amount of research has confirmed that there is a link between cholesterol and the development of AD. A high cholesterol levels in mid-life is a risk for AD and statins, i.e., cholesterol-lowering drugs, reduce this risk. Statins may not only inhibit enzymes involved in the endogenous synthesis of cholesterol but also affect enzymes involved in Abeta metabolism, i.e., alpha-secretase and beta-secretase. This normalises the breakdown of APP thereby promoting the non-amyloidogenic pathway. In this review, investigations focusing on cholesterol and Alzheimer's disease are presented. |
| 16799405 | Apolipoprotein E genotype and plasma lipid levels in Caucasian diabetic patients.OBJECTIVE: Apo E polymorphism has been shown to affect lipid profiles in non-diabetic and diabetic populations. We evaluated the relationship between Apo E phenotype and fasting lipid plasma levels in type 2 diabetes patients. METHODS: Two hundred and ten French type 2 diabetic patients (115 men and 95 women) without any lipid lowering drugs were studied. Fasting lipids were measured by usual methods and Apo E genotype was established for each patient: PCR was followed by digestion of the amplification product with restriction enzymes and separation of the fragments by polyacrylamide gel. RESULTS: Genotypes epsilon3/epsilon3, epsilon2/epsilon3 and epsilon3/epsilon4, epsilon2/epsilon2 and epsilon2/epsilon4 were found in 68.1%, 14.8%, 15.7%, 1.0% and 0.5%, respectively. No patient had the epsilon4/epsilon4 genotype. Lipid plasma levels were compared between E3 group (epsilon3/epsilon3) as a reference and E2 (epsilon2/epsilon2 or epsilon2/epsilon3) or E4 (epsilon3/epsilon4 or epsilon2/epsilon4). Total cholesterol, LDL cholesterol and Apo B levels were lower in the E2 group. Total cholesterol, LDL cholesterol and Apo B levels were higher in the E4 group. HDL cholesterol levels were increased in the E4 group, as only previously observed in Japanese populations. CONCLUSION: These results agree with those already reported in diabetic patients of several western European countries. E4 allele carriers have a greater cardio-vascular risk and this could be partially explained by the metabolic variation in lipid metabolism induced by E4 with higher LDL cholesterol and Apo B levels. These results observed in French diabetic subjects without any lipid-lowering drugs may be used as a reference for other studies performed in France. |
| 19371798 | The -9247 T/C polymorphism in the SOST upstream regulatory region that potentially affects C/EBPalpha and FOXA1 binding is associated with osteoporosis.Accumulating evidence shows that genes that cause monogenic diseases also contribute to similar complex disease in the general population. We sought to determine whether the allelic variation in seven monogenic bone disease genes (CLCN7, TCIRGI, SOST, CA2, CSTK, TGFB1 and SLC26A2) contributes to osteoporosis/bone mineral density (BMD) variation in the normal Chinese population. We conducted a gene-wide tag SNP-based association study in 1243 Chinese subjects with low BMD (Z-scores < or = -1.28, equivalent to the lowest 10% of the population) and high BMD (Z-score > or = +1.0). Twenty-two tag SNPs were selected and genotyped by using the high-throughput Sequenom genotyping platform. Allelic and haplotype association tests were conducted by Haploview and binary logistic regression analyses. The -9247 polymorphism rs1230399 in the upstream regulatory region of the sclerostin gene showed significant genotypic/allelic associations with spine, femoral neck, trochanter and total hip BMD (P=0.03-0.004). The T-allele of rs1230399 increased the risk of osteoporosis (OR=1.52, P=0.005). Computational analysis showed that rs1230399 is located at the core consensus recognition site of two cooperating transcription factors C/EBPalpha and FOXA1 that modulate estrogen receptor function. T-->C polymorphism abolishes the binding of both C/EBPalpha and FOXA1 to the sclerostin gene. Our data suggest a mechanistic link between rs1230399 and BMD through estrogen ERalpha/FOXA1 signaling pathways driven by long-distance enhancers. |
| 19668753 | Thrombophilic screening in retinal artery occlusion patients.BACKGROUND: Retinal artery occlusion (RAO) is an ischemic vascular damage of the retina, which frequently leads to sudden, mostly irreversible loss of vision. In this study, blood thrombophilic factors as well as cardiovascular risk factors were investigated for their relevance to this pathology. Thrombophilic risk factors so far not evaluated were included in the study. PATIENTS AND METHODS: 28 RAO patients and 81 matched control subjects were examined. From blood samples, protein C, protein S, antithrombinopathy, and factor V (Leiden) mutation (FV), factor II gene polymorphism, factor VIIIC level, plasminogen activity, lipoprotein(a) and fibrinogen levels, hyperhomocysteinemia and presence of anticardiolipin - antiphospholipid antibodies were investigated. Possibly relevant pathologies such as diabetes mellitus, hypertension, and ischemic heart disease were also registered. Statistical analysis by logistic regression was performed with 95% confidence intervals. RESULTS: In the group of patients with RAO only the incidence of hypertension (OR: 3.33, 95% CI: 1.30-9.70, p = 0.014) as an average risk factor showed significant difference, but thrombophilic factors such as hyperfibrinogenemia (OR: 2.9, 95% CI: 1.29-6.57, p = 0.010) and the presence of FV (Leiden mutation) (OR: 3.9, 95% CI: 1.43-10.96, p = 0.008) increased the chances of developing this disease. CONCLUSIONS: Our results support the assumption that thrombophilia may contribute to the development of RAO besides vascular damage due to the presence of cardiovascular risk factors. Further studies are needed, however, to justify the possible use of secondary prophylaxis in form of anticoagulant/antiplatelet therapy. |
| 22054870 | A genome-wide scan for common variants affecting the rate of age-related cognitive decline.Age-related cognitive decline is likely promoted by accumulated brain injury due to chronic conditions of aging, including neurodegenerative and vascular disease. Because common neuronal mechanisms may mediate the adaptation to diverse cerebral insults, we hypothesized that susceptibility for age-related cognitive decline may be due in part to a shared genetic network. We have therefore performed a genome-wide association study using a quantitative measure of global cognitive decline slope, based on repeated measures of 17 cognitive tests in 749 subjects from the Religious Orders Study. Top results were evaluated in 3 independent replication cohorts, consisting of 2279 additional subjects with repeated cognitive testing. As expected, we find that the Alzheimer's disease (AD) susceptibility locus, APOE, is strongly associated with rate of cognitive decline (P(DISC) = 5.6 x 10(-9); P(JOINT)= 3.7 x 10(-27)). We additionally discover a variant, rs10808746, which shows consistent effects in the replication cohorts and modestly improved evidence of association in the joint analysis (P(DISC) = 6.7 x 10(-5); P(REP) = 9.4 x 10(-3); P(JOINT) = 2.3 x 10(-5)). This variant influences the expression of 2 adjacent genes, PDE7A and MTFR1, which are potential regulators of inflammation and oxidative injury, respectively. Using aggregate measures of genetic risk, we find that known susceptibility loci for cardiovascular disease, type 2 diabetes, and inflammatory diseases are not significantly associated with cognitive decline in our cohort. Our results suggest that intermediate phenotypes, when coupled with larger sample sizes, may be a useful tool to dissect susceptibility loci for age-related cognitive decline and uncover shared molecular pathways with a role in neuronal injury. |
| 23863634 | AMPK, insulin resistance, and the metabolic syndrome.Insulin resistance (IR) and hyperinsulinemia are hallmarks of the metabolic syndrome, as are central adiposity, dyslipidemia, and a predisposition to type 2 diabetes, atherosclerotic cardiovascular disease, hypertension, and certain cancers. Regular exercise and calorie restriction have long been known to increase insulin sensitivity and decrease the prevalence of these disorders. The subsequent identification of AMP-activated protein kinase (AMPK) and its activation by exercise and fuel deprivation have led to studies of the effects of AMPK on both IR and metabolic syndrome-related diseases. In this review, we evaluate this body of literature, with special emphasis on the hypothesis that dysregulation of AMPK is both a pathogenic factor for these disorders in humans and a target for their prevention and therapy. |
| 23956345 | Elevated fetal adipsin/acylation-stimulating protein (ASP) in obese pregnancy: novel placental secretion via Hofbauer cells.CONTEXT AND OBJECTIVE: Obesity in pregnancy is associated with increased risks of obesity in the offspring. We investigated the relationship between obesity in pregnancy and circulating maternal and fetal levels of adipose tissue-derived factors adipsin and acylation stimulating protein (ASP) in lean and obese mothers. DESIGN: Paired peripheral and cord blood samples were taken. Paired fat and placenta tissue were taken for explant culture. Media were assayed for secreted adipsin and ASP. Clinical parameters assayed included fasting insulin, glucose, and adipsin. SETTING: The study was conducted at a university hospital maternity unit. PATIENTS: Patients included 35 lean [body mass index (BMI) 19-25 kg/m(2), mean age 32 years and 39 obese (BMI) > 30 kg/m(2), mean age 32.49 years] pregnant Caucasian women, delivered by cesarean section at term. MAIN OUTCOME MEASURE: Identification of placental macrophages [Hofbauer cells (HBCs)], as a source of adipsin and ASP was determined. RESULTS: HBCs secreted both adipsin and ASP. Cord levels of adipsin (1663.78 +/- 52.76 pg/mL) and ASP (354.48 +/- 17.17 ng/mL) were significantly elevated in the offspring of obese mothers compared with their lean controls [1354.66 +/- 33.87 pg/mL and 302.63 +/- 14.98 ng/mL, respectively (P < .05 for both)]. Placentae from obese mothers released significantly more adipsin and ASP than placentae from lean mothers [546.0 +/- 44 pg/mL . g vs 284.56 +/- 43 pg/mL . g and 5485.75 +/- 163.32 ng/mL . g vs 2399.16 +/- 181.83 ng/mL . g, respectively (P < .05 for both)]. Circulating fetal adipsin and ASP positively correlated with maternal BMI (r = 0.611, P < .0001, and r = 0.391, P < .05, respectively). Fetal adipsin correlated positively with maternal (r = 0.482, P < .01) and fetal homeostasis model assessment of insulin resistance (r = 0.465, P < .01). CONCLUSIONS: We demonstrate novel secretion of adipsin and ASP by placental HBCs. |
| 11683425 | Association between COLIA1 Sp1 alleles and femoral neck geometry.Genetic factors play an important role in the pathogenesis of osteoporosis by affecting bone mineral density and other predictors of osteoporotic fracture risk such as ultrasound properties of bone and skeletal geometry. We previously identified a polymorphism of a Sp1 binding site in the Collagen Type 1 Alpha 1 gene (COLIA1) that has been associated with reduced BMD and an increased risk of osteoporotic fractures in several populations. Here we looked for evidence of an association between COLIA1 Sp1 alleles and femoral neck geometry. The study group comprised 153 patients with hip fracture, and 183 normal subjects drawn at random from the local population. Femoral neck geometry was assessed by analysis of pelvic radiographs in the fracture patients and DXA scan printouts in the population-based subjects. The COLIA1 genotypes were detected by polymerase chain reaction and were in Hardy Weinberg equilibrium: "SS" = 222 (66%); "Ss" = 105 (31.3%); and "ss" = 9 (2.7%). There was no significant difference in hip axis length or femoral neck width between the genotype groups, but femoral neck-shaft angle was increased by about 2 degrees in the Ss/ss genotype groups (n = 114) when compared with SS homozygotes (n = 222) (P = 0.001). Previous studies have suggested that an increased femoral neck-shaft angle may increase the risk of hip fracture in the event of a sideways fall by influencing the forces that act on the femoral neck. The association COLIAI genotype and increased femoral neck angle noted here may therefore contribute to the BMD-independent increase in hip fracture risk noted in previous studies of individuals who carry the 's' allele. |
| 16343576 | Nifedipine represses ion channels, transporters and Ca(2+)-binding proteins in hearts of spontaneously hypertensive rats.The Ca(2+) antagonists nifedipine has been used for more than three decades to treat hypertension, but its effects on the transcriptional regulation of cardiac genes are basically unknown. We therefore studied expression of genes coding for ion channels, ion transporters and associated partners as well as Ca(2+)-binding proteins in ventricular tissue of normotensive and spontaneously hypertensive (SH) rats after repeated intraperitoneally (i.p.) dosing of nifedipine. Notably, we observed significant (P < 0.05) repression in transcript levels of most of the genes investigated, including cardiac Na(+), K(+), Ca(2+)-channels (L-type Ca(2+)-channel, K(ir)3.4, K(ir)6.1, Na(v)1.5), ATP-driven ion exchangers (Na(+)-K(+)-ATPase, NCX-1, PMCA 2 and 4, SERCA 2a and 2b) and their associated partners (phospholamban, RyR-2) as well as cytoskeletal proteins (alpha and beta-MHC, alpha cardiac and alpha skeletal actin, troponin T and I). Repression in transcript levels was, however, only seen in ventricular tissue of hypertensive animals. This points to fundamental differences in the mode of action of nifedipine in diseased and healthy animals. Indeed, this preponderance of repressed genes will promote disturbed ion homeostasis to result in contractile dysfunction. It is of considerable importance that repressed gene expression was also seen in end-stage human heart failure. We propose repression of cardiac-specific gene expression as a hallmark of nifedipine treatment in hypertrophic hearts. |
| 17339538 | Genetic variations associated with echocardiographic left ventricular traits in hypertensive blacks.Echocardiographic measures of cardiac target organ damage, including left ventricular mass and relative wall thickness, are powerful predictors of heart disease morbidity and mortality. The aim of this study is to investigate whether single nucleotide polymorphisms in candidate genes for hypertension and heart disease have effects on quantitative measures of hypertensive cardiac target organ damage, independent of their actions on blood pressure levels, in a cohort of hypertensive black sibships. To detect replication of genetic effects across samples, this study took advantage of the affected sibling pair design and created 2 samples, each with 448 unrelated individuals. As part of the Genetic Epidemiology Network of Arteriopathy Study, subjects were screened using 2D echocardiography, and 395 single nucleotide polymorphisms in 80 candidate genes were genotyped. Linear regression was used to test for single nucleotide polymorphisms significantly associated with left ventricular mass index (g/m(2.7)) or relative wall thickness after adjusting for associated covariates. Significant single nucleotide polymorphisms were subsequently tested for consistent directionality in genotype-phenotype relationships across samples. Three single nucleotide polymorphisms, 1 each in the APOE, SCN7A, and SLC20A1 genes, were significantly associated in both samples with left ventricular mass index and had replicate genotype-phenotype relationships. One in the ADRB1 gene was significantly associated with relative wall thickness with replicate effects in both samples. We identified genetic variation that significantly influences left ventricular traits with replicable effects in a cohort of hypertensive, black siblings. |
| 17485234 | L-FABP T94A is associated with fasting triglycerides and LDL-cholesterol in women.To determine the possible role of the common FABP1 T94A polymorphism in modulating susceptibility to traits of the metabolic syndrome, we analysed a random sample of 826 subjects from the European Prospective Investigation into Cancer and Nutrition (EPIC)-Potsdam cohort. Multivariate adjusted linear trend regression analysis of metabolic, anthropometric and blood pressure variables in FABP1 T94A genotypes were performed in both genders. In women, a significant trend of higher plasma triglyceride (P=0.01) and LDL-cholesterol (P=0.02) concentrations were seen for A-allele carriers after adjustment for age, menopausal status, hormone intake and Apo E genotype. Because elevated triglyceride and cholesterol levels are important risk factors of cardiovascular disease (CVD) and type 2 diabetes mellitus (T2DM), we additionally analysed the association of the T94A variant and disease risks in two studies enrolling 220 incident CVD and 192 incident T2DM patients of the EPIC-Potsdam cohort. After adjusting for age, sex, BMI and other covariates, we found no association between FABP1 T94A and CVD or T2DM. In conclusion, our study provides evidence for an association of the FABP1 T94A polymorphism and fasting triglycerides and LDL-cholesterol levels in females. These results support previous findings in fenofibrate-treated individuals and thereby provide some additional indication of the functional relevance of the FABP1 T94A SNP in hepatic fatty acid and lipid metabolism in humans. |
| 18597674 | Differential transferrin expression in placentae from normal and abnormal pregnancies: a pilot study.BACKGROUND: The placenta is an important site for iron metabolism in humans. It transfers iron from the mother to the fetus. One of the major iron transport proteins is transferrin, which is a blood plasma protein crucial for iron uptake. Its localization and expression may be one of the markers to distinguish placental dysfunction. METHODS: In the experimental study we used antibody preparation, mass spectrometric analysis, biochemical and immunocytochemical methods for characterization of transferrin expression on the human choriocarcinoma cell line JAR (JAR cells), placental lysates, and cryostat sections. Newly designed monoclonal antibody TRO-tf-01 to human transferrin was applied on human placentae from normal (n = 3) and abnormal (n = 9) pregnancies. RESULTS: Variations of transferrin expression were detected in villous syncytiotrophoblast, which is in direct contact with maternal blood. In placentae from normal pregnancies, the expression of transferrin in the syncytium was significantly lower (p < 0.001) when compared to placentae from abnormal ones (gestational diabetes, pregnancy induced hypertension, drug abuse). CONCLUSION: These observations suggest that in the case of abnormal pregnancies, the fetus may require higher levels of transferrin in order to prevent iron depletion due to the stress from the placental dysfunction. |
| 18752569 | Factor V Leiden is associated with pre-eclampsia but not with fetal growth restriction: a genetic association study and meta-analysis.BACKGROUND: Adverse pregnancy outcomes have been related to environmental and/or genetic factors. Of interest are genes associated with the clotting system as any perturbation in the balance of thrombotic and thrombolytic cascades could affect the placental circulation and hence the viability of the developing fetus. Several previous reports using relatively small numbers of cases and controls have suggested that there is a relationship between poor pregnancy outcomes and two polymorphisms, one in the factor V gene, the 1691G to A change (rs6025) located on chromosome 1q23 (factor V Leiden, FVL), and the other in the prothrombin gene, 20210G to A change (rs1799963) on chromosome 11p11-q12 (PT). These results, however, are conflicting. METHODS: We genotyped 6755 mother/infant pairs from the Avon Longitudinal Study of Parents and Children (ALSPAC) to determine whether maternal or fetal FVL or PT, either alone or in combination, are associated with fetal growth restriction (FGR) or pre-eclampsia (PE). We also added the present results to previous cohort studies using meta-analysis. RESULTS: Smoking, primiparity and lower body mass index (BMI) were all associated with FGR, but neither maternal nor fetal FVL or PT, singly or in combination, were associated with FGR in the ALSPAC cohort. Meta-analysis confirmed the lack of association between maternal FVL and FGR with a pooled odds ratio (OR) of 1.15 [95% confidence interval (CI) 0.95-1.39]. High BMI, primiparity, diabetes and chronic hypertension were all associated with pre-eclampsia. Combining ALSPAC results with previous studies in ameta-analysis indicated that maternal FVL is significantly associated with pre-eclampsia, with a pooled OR of 1.49 (95% CI 1.13-1.96). CONCLUSION: Neither maternal nor fetal FVL or PT, singly or in combination, are associated with FGR; this contradicts previous case-control studies and meta-analyses based on these studies. In a meta-analysis of all published cohort studies to date, maternal FVL appears to increase the risk of pre-eclampsia by almost 50%. This result is robust, homogeneous and does not appear to be affected by publication bias. |
| 18980783 | Effects of trans10cis12CLA-induced insulin resistance on retinol-binding protein 4 concentrations in abdominally obese men.In this randomized, placebo-controlled, double-blind study of 57 abdominally obese middle-aged men, conjugated linoleic acid (CLA) did not induce changes in retinol-binding protein 4 concentrations (RBP4), despite marked induced insulin resistance. Further, there were no associations between CLA-induced insulin resistance and changes in RBP4. |
| 20839008 | The majority of the genetic risk for Paget's disease of bone is explained by genetic variants close to the CSF1, OPTN, TM7SF4, and TNFRSF11A genes.Paget's disease of bone (PDB) is one of the most frequent metabolic bone disorders (1-5%), next to osteoporosis, affecting individuals above age 55. Sequestosome1 mutations explain a part of the PDB patients, but still the disease pathogenesis in the remaining PDB patients is largely unknown. Therefore, association studies investigating the relationship between genetic polymorphisms and sporadic PDB have been performed to find the genetic risk variants. Previously such studies indicated a role of the OPG and RANK gene. The latter was recently confirmed in a genome-wide association study (GWAS) which also indicated the involvement of chromosomal regions harbouring the CSF1 and OPTN gene. In this study, we sought to replicate these findings in a Belgian and a Dutch population. Similar significant results were obtained for the single nucleotide polymorphisms and the haplotypes. The most significant results are found in the CSF1 gene region, followed by the OPTN and TNFRSF11A gene region (p values ranging from 1.3 x 10(-4) to 3.8 x 10(-8), OR = 1.523-1.858). We next obtained significant association with a polymorphism from the chromosomal region around the TM7SF4 gene (p = 2.7 x 10(-3), OR = 1.427), encoding DC-STAMP which did not reach genome-wide significance in the GWAS, but based on its function in osteoclasts it can be considered a strong candidate gene. After meta-analysis with the GWAS data, p values ranged between 2.6 x 10(-4) and 8.8 x 10(-32). The calculated cumulative population attributable risk of these four loci turned out to be about 67% in our two populations, indicating that most of the genetic risk for PDB is coming from genetic variants close to these four genes. |
| 21210148 | Metabolic, inflammatory, endothelial and haemostatic markers in a group of Italian obese children and adolescents.Childhood obesity and its related comorbidities are increasingly recognised in children, predisposing them to early cardiovascular disease and metabolic syndrome. The objective of the study was to investigate markers of metabolism, inflammation and haemostasis in a group of Italian obese children and adolescents. Fifty-nine obese and 40 non-obese subjects were recruited. Fasting glucose and insulin, total cholesterol, HDL and LDL cholesterol, triglycerides, high-sensitivity C-reactive protein (hsCRP), tumour necrosis factor alpha (TNF-alpha), and adiponectin were measured. Hypercoagulability was assessed by measuring the circulating levels of thrombin-antithrombin complex (TAT), D: -dimer, fibrinogen, plasminogen activator inhibitor 1 (PAI-1) and von Willebrand Factor (vWF). A significant degree of insulin resistance was present in obese subjects compared with controls (p < 0.0001). The obese showed higher levels of total cholesterol, LDL cholesterol and triglycerides, and lower levels of HDL cholesterol than controls (p < 0.0001). Circulating levels of hsCRP and TNF-alpha were significantly higher in obese than in controls while serum adiponectin levels were significantly lower in obese than non-obese subjects (p < 0.001; p = 0.031; p < 0.0001, respectively). vWF, TAT, D-dimer, fibrinogen and PAI-1 levels were significant higher in obese subjects compared with control group (p = 0.02; p < 0.0001; p = 0.0037; p < 0.0001; p = 0.017, respectively). In conclusion, our results suggest that childhood obesity per se is associated with a proinflammatory and prothrombotic state. |
| 23731511 | Histological and molecular characterisation of feline humeral condylar osteoarthritis.BACKGROUND: Osteoarthritis (OA) is a clinically important and common disease of older cats. The pathological changes and molecular mechanisms which underpin the disease have yet to be described. In this study we evaluated selected histological and transcriptomic measures in the articular cartilage and subchondral bone (SCB) of the humeral condyle of cats with or without OA. RESULTS: The histomorphometric changes in humeral condyle were concentrated in the medial aspect of the condyle. Cats with OA had a reduction in articular chondrocyte density, an increase in the histopathological score of the articular cartilage and a decrease in the SCB porosity of the medial part of the humeral condyle. An increase in LUM gene expression was observed in OA cartilage from the medial part of the humeral condyle. CONCLUSIONS: Histopathological changes identified in OA of the feline humeral condyle appear to primarily affect the medial aspect of the joint. Histological changes suggest that SCB is involved in the OA process in cats. Differentiating which changes represent OA rather than the aging process, or the effects of obesity and or bodyweight requires further investigation. |
| 12660865 | Z-2 aldose reductase allele and diabetic retinopathy in India.Genetic factors have been identified that regulate the severity and the rapidity of onset of retinopathy in diabetic patients. Polymorphisms in (CA)( n) present upstream of the promoter of the aldose reductase (ALR2 ) gene have been shown to be associated with retinopathy in different ethnic populations. We aimed to study the association between the (CA)( n) polymorphism and type 2 diabetic patients with and without retinopathy in the Asian Indian population. We screened 105 diabetic patients with retinopathy (DR) and 109 diabetic patients without retinopathy (DNR) for the (CA)( n) polymorphism and compared the results with those of an unrelated healthy control group (CT). We identified 13 alleles in our diabetic population. The Z-2 allele (136 bp) showed an association with the DR group (13.81%) with a significant p value (p = 0.029) when compared with the DNR group (7.34%). The Z-2 allele also showed a significant association with those DR patients who had proliferative retinopathy (PDR) and maculopathy (MAC) (p = 0.004). The Z-2 allele is, therefore, a high-risk allele for diabetic retinopathy in the Asian Indian patients. |
| 17933712 | Tubular atrophy, interstitial fibrosis, and inflammation in type 2 diabetic db/db mice. An accelerated model of advanced diabetic nephropathy.OBJECTIVE: Advanced diabetic nephropathy (DN) is difficult to address experimentally in mice because available models of DN lack global glomerulosclerosis and major tubulointerstitial pathology. Accelerating the development of DN in mice would be desirable for feasible experimental validation of potential targets that mediate the progression to late stage DN. METHODS: 6 week old male db/db mice underwent uninephrectomy and the development of nephropathy was compared to wild-type mice and sham-operated db/db mice. RESULTS: Uninephrectomy at young age was associated with increased albuminuria and severe glomerulosclerosis in 37% of glomeruli at 24 weeks of age as compared to sham-operated db/db mice (8%). Uninephrectomy also increased the number of glomerular macrophages in db/db mice. The uninephrectomy-related acceleration of glomerular damage was associated with significant tubulointerstitial injury as indicated by an increase in indices of tubular cell damage, tubular dilatation, and expansion of interstitial volume. Uninephrectomy markedly increased the renal mRNA expression of Mcp-1/Ccl2, Tgf-beta, and collagen I. CONCLUSION: Early uninephrectomy can accelerate the development of advanced DN in db/db mice which may be instrumental in the design of interventional studies that intend to focus on the molecular pathology of the progression to late stage DN. |
| 18304430 | [The effect of calorie restriction on the expression of liver's gluconeogenesis genes of rats fed a high fat diet].OBJECTIVE: To observe the effect of calorie restriction on the high fat diet rats mRNA expressions of liver forkhead box O1(FoxO1), phosphoenolpyruvate carboxykinase (PEPCK), glucose-6-phosphatase (G-6-P) and to explore the possible mechanisms. METHODS: 24 normal 6-week-old male Wistar rats were randomly divided into three groups: normal chow group (NC, n = 7), high fat diet group (HF, n = 9) and calorie restriction group (CR, n = 8). They were fed for 12 weeks. At the end of the experiment, the rats were sacrificed and their fasting blood glucose (FBG), insulin (INS), triglycerides (TG), total cholesterol (TC) were measured. Their visceral fat (VF) and body weight (BW) were also measured and VF/BW was calculated. Gene expression was investigated by using semi-quantitative RT-PCR methods. Liver histology was studied with HE stained slides. RESULTS: Compared with the NC group, HF group rats developed visceral obesity which was accompanied by higher FBG, plasma INS, TG, and TC. The levels of FoxO1, PEPCK, and G-6-P increased by 18.9%, 33.8%, and 24.6%, respectively (P less than 0.01). Liver steatosis was observed with microscopy. The BW, VF FBG, INS, TG and TC of the CR group rats were lower in comparison to those of the HF group. The levels of FoxO1, PEPCK and G-6-P were lower by 26.6%, 35.0%, 34.3% (P less than 0.01). Meanwhile, liver steatosis was also milder. CONCLUSION: Calorie restriction can inhibit the expressions of FoxO1, PEPCK and G-6-P, strengthen insulin signal conduction, suppress gluconeogenesis and thus regulate glycometabolism. |
| 20160192 | Context-dependent associations between variation in risk of ischemic heart disease and variation in the 5' promoter region of the apolipoprotein E gene in Danish women.OBJECTIVE: Variations in the noncoding single-nucleotide polymorphisms (SNPs) at positions 560 and 832 in the 5' promoter region of the apolipoprotein E gene define genotypes that distinguish between high and low concentrations of plasma total and high-density lipoprotein cholesterol and triglycerides. We addressed whether these genotypes improve the prediction of ischemic heart disease (IHD) in subsamples of individuals defined by traditional risk factors and the genotypes defined by the epsilon(2), epsilon(3), and epsilon(4) alleles in exon 4 of the apolipoprotein E gene. METHODS AND RESULTS: In a sample of 3686 female and 2772 male participants of the Copenhagen City Heart Study who were free of IHD events, 576 individuals (257 women, 7.0% and 319 men, 11.5%) were diagnosed as having developed IHD in 6.5 years of follow-up. Using a stepwise Patient Rule-Induction Method modeling strategy that acknowledges the complex pathobiology of IHD, we identified a subsample of 764 elderly women (> or =65 years) with hypertriglyceridemia who had a history of smoking, a history of hypertension, or a history of both in which the A(560)T(832)/A(560)T(832) and A(560)T(832)/A(560)G(832) 5' 2-SNP genotypes had a higher cumulative incidence of IHD (172/1000) compared to the incidence of 70/1000 in the total sample of women. CONCLUSIONS: Our study validates that 5' apolipoprotein E genotypes improve the prediction of IHD and documents that the improvement is greatest in a subset defined by a particular combination of traditional risk factors in Copenhagen City Heart Study female participants. We discuss the use of these genotypes in medical risk assessment of IHD in the population represented by the Copenhagen City Heart Study. |
| 20173020 | Cardiovascular and metabolic risk profile and acylation-stimulating protein levels in children with Prader-Willi syndrome and effects of growth hormone treatment.CONTEXT: Reports on the cardiovascular and metabolic risk profile in children with Prader-Willi syndrome (PWS) and the effects of GH treatment are scarce. Acylation-stimulating protein (ASP) stimulates glucose uptake and triglyceride storage in adipose tissue. OBJECTIVES: The aim was to study the metabolic and cardiovascular risk profile and ASP levels and to investigate the effects of GH treatment. DESIGN: We conducted a randomized controlled GH trial. Infants and prepubertal children were assigned to receive GH (1 mg/m(2) . d) or to serve as controls for 12 and 24 months, respectively. PATIENTS: Eighty-five children with PWS (mean +/- sd age of 4.9 +/- 3.0 yr) participated in the study. MAIN OUTCOME MEASURES: We measured fat percentage (fat%) with dual-energy x-ray absorptiometry, blood pressure, fasting insulin and glucose levels, serum lipids, and ASP levels. RESULTS: Mean +/- SD fat% was 28.4 +/- 6.2 in infants and 36.9 +/- 8.5 in prepubertal children. Fat% sd score (SDS) was above 2 SDS in 95% of prepubertal children. In addition, 63% of infants and 73% of prepubertal children demonstrated at least one cardiovascular risk factor, defined as hypertension or dyslipidemia. The metabolic syndrome was demonstrated in 5% of all children. Mean +/- sd baseline ASP was 107 +/- 45 nmol/liter (normal < 58 nmol/liter) and correlated with fat mass and TG levels. GH improved fat%SDS and the HDLc/LDLc ratio (P < 0.0001 and P = 0.04). GH had no effect on mean ASP levels in this population. CONCLUSIONS: Many children with PWS had dyslipidemia and high ASP levels. GH improved fat% and high-density lipoprotein cholesterol/low-density lipoprotein cholesterol, but not ASP. High ASP levels may prevent complete normalization of fat%SDS during GH treatment but may contribute in keeping glucose and insulin levels within normal range. |
| 21496886 | Effect of dibutyryl cyclic adenosine monophosphate on the gene expression of plasminogen activator inhibitor-1 and tissue factor in adipocytes.INTRODUCTION: Hypertrophic adipocytes in obese states express the elevated levels of plasminogen activator inhibitor-1 (PAI-1) and tissue factor (TF). An increase in the intracellular concentration of cyclic adenosine monophosphate (cAMP) promotes triglyceride hydrolysis and may improve dysregulation of adipocyte metabolism. Here, we investigate the effect of dibutyryl-cAMP (a phosphodiesterase-resistant analog of cAMP) on the gene expression of PAI-1 and TF in adipocytes. MATERIALS AND METHODS: Differentiated 3T3-L1 adipocytes were treated with dibutyryl-cAMP and agents that would be expected to elevate intracellular cAMP, including cilostazol (a phosphodiesterase inhibitor with anti-platelet and vasodilatory properties), isoproterenol (a beta adrenergic agonist) and forskolin (an adenylyl cyclase activator). The levels of PAI-1 and TF mRNAs were measured using real-time quantitative reverse transcription-PCR. RESULTS AND CONCLUSIONS: The treatment of adipocytes with dibutyryl-cAMP resulted in the inhibition of both lipid accumulation and TF gene expression. However, PAI-1 gene expression was slightly but significantly increased by dibutyryl-cAMP. On the other hand, cilostazol inhibited the expression of PAI-1 without affecting lipid accumulation. When the adipocytes were treated with cilostazol in combination with isoproterenol or forskolin, the inhibitory effect of cilostazol on PAI-1 gene expression was counteracted, thus suggesting that inhibition by cilostazol may not be the result of intracellular cAMP accumulation by phosphodiesterase inhibition. These results suggest the implication of cAMP in regulation of the gene expression of TF and PAI-1 in adipocytes. Our findings will serve as a useful basis for further research in therapy for obesity-associated thrombosis. |
| 22403621 | Identification of periostin as a critical marker of progression/reversal of hypertensive nephropathy.Progression of chronic kidney disease (CKD) is a major health issue due to persistent accumulation of extracellular matrix in the injured kidney. However, our current understanding of fibrosis is limited, therapeutic options are lacking, and progressive degradation of renal function prevails in CKD patients. Uncovering novel therapeutic targets is therefore necessary.We have previously demonstrated reversal of renal fibrosis with losartan in experimental hypertensive nephropathy. Reversal was achieved provided that the drug was administered before late stages of nephropathy, thereby determining a non-return point of CKD progression. In the present study, to identify factors critically involved in the progression of renal fibrosis, we introduced losartan at the non-return point in L-NAME treated Sprague Dawley rats. Our results showed either reversal or progression of renal disease with losartan, defining 2 groups according to the opposite evolution of renal function. We took advantage of these experimental conditions to perform a transcriptomic screening to identify novel factors potentially implicated in the mechanisms of CKD progression. A secondary analysis of selected markers was thereafter performed. Among the targets identified, periostin, an extracellular matrix protein, presented a significant 3.3-fold higher mRNA expression in progression compared to reversal group. Furthermore, independent of blood pressure, periostin was strongly correlated with plasma creatinine, proteinuria and renal blood flow, hallmarks of hypertensive renal disease severity. Periostin staining was predominant in the injured regions, both in experimental hypertensive and human nephropathy.These results identify periostin as a previously unrecognized marker associated with disease progression and regression in hypertensive nephropathy and suggest measuring periostin may be a sensitive tool to evaluate severity, progression and response to therapy in human kidney disease associated to hypertension. |
| 22575419 | Investigation of the mechanism(s) involved in decreasing increased fibrinogen activity in hyperglycemic conditions using L-lysine supplementation.INTRODUCTION: Fibrinogen is a plasma glycoprotein that participates in the hemostasis system. Its malfunction has been reported as a consequence of diabetic complications. In this study, the inhibitory effect of L-Lysine (Lys) on the nonenzymatic glycation of fibrinogen was investigated in both in vitro and in vivo conditions. MATERIALS AND METHODS: Fibrinogen was incubated with glucose in the presence or absence of Lys. Then, its structure was studied by fluorescence spectroscopy, circular dichroism, and electrophoresis. The Clauss method was used to determine fibrinogen activity. In addition, one of the two groups of type 2 diabetic patients receiving ordinary treatment was additionally treated with Lys for 3 months. Fibrinogen activity and some other parameters were evaluated in their plasma. RESULTS: The results indicated increases in the activity of glycated fibrinogen in both of the in vivo and in vitro experiments. Advanced glycation end products were increased by time, as shown using fluorometry in both the plasma of the diabetic patients and the incubation medium of protein with glucose. The circular dichroism spectra showed some changes in the fibrinogen secondary and tertiary structures after glycation. The electrophoretic mobility of the glycated fibrinogen changed and the cross-link formation between the fibrinogen subunits due to glycation was observed. Lys inhibited all of the mentioned fibrinogen changes both in the in vitro experiments and after its administration to the diabetic patients. CONCLUSION: Lys, as an inhibitor of protein glycation, improved fibrinogen's structure and function, both in vitro and in vivo. |
| 22768840 | Wip1-dependent regulation of autophagy, obesity, and atherosclerosis.Obesity and atherosclerosis-related diseases account for over one-third of deaths in the western world. Controlling these conditions remains a major challenge due to an incomplete understanding of the molecular pathways involved. Here, we show that Wip1 phosphatase, a known negative regulator of Atm-dependent signaling, plays a major role in controlling fat accumulation and atherosclerosis in mice; specifically, Wip1 deficiency prevents both conditions. In the course of atherosclerosis, deletion of Wip1 results in suppression of macrophage conversion into foam cells, thus preventing the formation of atherosclerotic plaques. This process appears to be independent of p53 but rely on a noncanonical Atm-mTOR signaling pathway and on selective autophagy in regulation of cholesterol efflux. We propose that the Wip1-dependent control of autophagy and cholesterol efflux may provide avenues for treating obesity and atherosclerosis. |
| 1466268 | Expression of kallikrein-binding protein and alpha 1-antitrypsin genes in response to sex hormones, growth, inflammation and hypertension.We have recently purified rat kallikrein-binding protein (RKBP) and alpha 1-antitrypsin (alpha 1-AT) to homogeneity and isolated, sequenced cDNAs encoding these potential regulators of tissue kallikreins. Characterization of the cDNA and the gene has established the identity of the kallikrein-binding protein as a new member of the serpin (serine proteinase inhibitor) superfamily. Using the cDNA probes in Northern blot hybridization, we found a differential regulation of RKBP and alpha 1-AT gene expression in the liver. Ovariectomy results in a 67% reduction of RKBP mRNA levels but a 30% increase of alpha 1-AT mRNA levels. Estradiol or progesterone treatment of the ovariectomized rats increases RKBP transcripts by 2.5- and 6.5-fold, respectively, but reduces alpha 1-AT mRNA level by 30% and 45%, respectively. In contrast to kininogen expression, both RKBP and alpha 1-AT mRNA levels in the liver are at the lowest at birth and rapidly increase during growth and development. Rats injected with endotoxin from 4 to 24 h show a time-dependent decrease of RKBP mRNA levels while the same treatment induces alpha 1-AT gene expression. RKBP mRNA levels in the normotensive Wistar Kyoto (WKY) rats are higher than those in the spontaneously hypertensive rats (SHR) while there are no differences of alpha 1-AT mRNA levels between SHR and WKY.(ABSTRACT TRUNCATED AT 250 WORDS) |
| 18463231 | Transcription factor PU.1 is expressed in white adipose and inhibits adipocyte differentiation.PU.1 transcription factor is a critical regulator of hematopoiesis and leukemogenesis. Because PU.1 interacts with transcription factors GATA-2 and C/EBPalpha, and both are involved in the regulation of adipogenesis, we investigated whether PU.1 plays a role in the regulation of adipocyte differentiation. Our data indicate that PU.1 is expressed in white adipose tissue. PU.1 protein can also be detected in cultured 3T3-L1 adipocytes. Forced expression of PU.1 in 3T3-L1 cells inhibits adipocyte differentiation, whereas deletion of the transactivation domain of PU.1 abolishes this effect. The inhibition of adipocyte differentiation by PU.1 is achieved, at least in part, through repression of the transcriptional activity of C/EBPalpha and C/EBPbeta. Furthermore, GATA-2 and PU.1 have an additive inhibitory effect on C/EBP transactivation and adipogenesis. Finally, the expression of PU.1 is increased in white adipose of obese mice. |
| 18565099 | Cathepsin S genotypes are associated with Apo-A1 and HDL-cholesterol in lean and obese French populations.Cathepsin S (CTSS) is a cysteine protease that has a central role in remodeling the extracellular matrix and, as such, has been implicated in the etiology of cardiovascular disease. This study used five tag single nucleotide polymorphisms (tSNPs) to screen the CTSS gene in healthy lean (n = 1891) and obese French populations (n = 477) for their association with various phenotypes: body mass index, waist-to-hip ratio, glycemia, total cholesterol, triglycerides, high-density lipoprotein cholesterol (HDL-C), apolipoprotein A1 (Apo-A1) and apolipoprotein B. Significant associations were identified between rs11576175 tSNP (A/G) and Apo-A1 and HDL-C plasma levels in a sex-specific manner. Lean female subjects homozygous for the minor A-allele had higher levels of circulating Apo-A1 (p = 0.0003), while lean male A/A carriers had higher levels of HDL-C (p = 0.007) compared with the other genotypes. In the obese cohort, associations were found between three tSNPs and Apo-A1 levels in adult female subjects: rs10888390 (G/A), p = 0.01; rs10888394 (T/C), p = 0.03; and rs1136774 (C/T), p = 0.02; however, only rs10888390 remained significant in a combined model (p = 0.03). These results provide the first evidence that CTSS sequence variations are associated with two human metabolic risk factors for cardiovascular diseases: plasma Apo-A1 and HDL-C concentrations. |
| 21782034 | Signaling by vitamin A and retinol-binding protein in regulation of insulin responses and lipid homeostasis.Vitamin A, retinol, circulates in blood bound to serum retinol binding protein (RBP) and is transported into cells by a membrane protein termed stimulated by retinoic acid 6 (STRA6). It was reported that serum levels of RBP are elevated in obese rodents and humans, and that increased level of RBP in blood causes insulin resistance. A molecular mechanism by which RBP can exert such an effect is suggested by the recent discovery that STRA6 is not only a vitamin A transporter but also functions as a surface signaling receptor. Binding of RBP-ROH to STRA6 induces the phosphorylation of a tyrosine residue in the receptor C-terminus, thereby activating a JAK/STAT signaling cascade. Consequently, in STRA6-expressing cells such as adipocytes, RBP-ROH induces the expression of STAT target genes, including SOCS3, which suppresses insulin signaling, and PPARgamma, which enhances lipid accumulation. RBP-retinol thus joins the myriad of cytokines, growth factors and hormones which regulate gene transcription by activating cell surface receptors that signal through activation of Janus kinases and their associated transcription factors STATs. This article is part of a Special Issue entitled Retinoid and Lipid Metabolism. |

  
  
----- Star papers (those papers include more than 100 genes) -----  

|  |  |
| --- | --- |
| 15621726 | Bone morphogenetic proteins.Bone morphogenetic proteins (BMPs) are multi-functional growth factors that belong to the transforming growth factor beta (TGFbeta) superfamily. The roles of BMPs in embryonic development and cellular functions in postnatal and adult animals have been extensively studied in recent years. Signal transduction studies have revealed that Smad1, 5 and 8 are the immediate downstream molecules of BMP receptors and play a central role in BMP signal transduction. Studies from transgenic and knockout mice and from animals and humans with naturally occurring mutations in BMPs and related genes have shown that BMP signaling plays critical roles in heart, neural and cartilage development. BMPs also play an important role in postnatal bone formation. BMP activities are regulated at different molecular levels. Preclinical and clinical studies have shown that BMP-2 can be utilized in various therapeutic interventions such as bone defects, non-union fractures, spinal fusion, osteoporosis and root canal surgery. Tissue-specific knockout of a specific BMP ligand, a subtype of BMP receptors or a specific signaling molecule is required to further determine the specific role of a BMP ligand, receptor or signaling molecule in a particular tissue. BMPs are members of the TGFbeta superfamily. The activity of BMPs was first identified in the 1960s (Urist, M.R. (1965) "Bone formation by autoinduction", Science 150, 893-899), but the proteins responsible for bone induction remained unknown until the purification and sequence of bovine BMP-3 (osteogenin) and cloning of human BMP-2 and 4 in the late 1980s (Wozney, J.M. et al. (1988) "Novel regulators of bone formation: molecular clones and activities", Science 242, 1528-1534; Luyten, F.P. et al. (1989) "Purification and partial amino acid sequence of osteogenin, a protein initiating bone differentiation", J. Biol. Chem. 264, 13377-13380; Wozney, J.M. (1992) "The bone morphogenetic protein family and osteogenesis", Mol. Reprod. Dev. 32, 160-167). To date, around 20 BMP family members have been identified and characterized. BMPs signal through serine/threonine kinase receptors, composed of type I and II subtypes. Three type I receptors have been shown to bind BMP ligands, type IA and IB BMP receptors (BMPR-IA or ALK-3 and BMPR-IB or ALK-6) and type IA activin receptor (ActR-IA or ALK-2) (Koenig, B.B. et al. (1994) "Characterization and cloning of a receptor for BMP-2 and BMP-4 from NIH 3T3 cells", Mol. Cell. Biol. 14, 5961-5974; ten Dijke, P. et al. (1994) "Identification of type I receptors for osteogenic protein-1 and bone morphogenetic protein-4", J. Biol. Chem. 269, 16985-16988; Macias-Silva, M. et al. (1998) "Specific activation of Smad1 signaling pathways by the BMP7 type I receptor, ALK2", J. Biol. Chem. 273, 25628-25636). Three type II receptors for BMPs have also been identified and they are type II BMP receptor (BMPR-II) and type II and IIB activin receptors (ActR-II and ActR-IIB) (Yamashita, H. et al. (1995) "Osteogenic protein-1 binds to activin type II receptors and induces certain activin-like effects", J. Cell. Biol. 130, 217-226; Rosenzweig, B.L. et al. (1995) "Cloning and characterization of a human type II receptor for bone morphogenetic proteins", Proc. Natl Acad. Sci. USA 92, 7632-7636; Kawabata, M. et al. (1995) "Cloning of a novel type II serine/threonine kinase receptor through interaction with the type I transforming growth factor-beta receptor", J. Biol. Chem. 270, 5625-5630). Whereas BMPR-IA, IB and II are specific to BMPs, ActR-IA, II and IIB are also signaling receptors for activins. These receptors are expressed differentially in various tissues. Type I and II BMP receptors are both indispensable for signal transduction. After ligand binding they form a heterotetrameric-activated receptor complex consisting of two pairs of a type I and II receptor complex (Moustakas, A. and C.H. Heldi (2002) "From mono- to oligo-Smads: the heart of the matter in TGFbeta signal transduction" Genes Dev. 16, 67-871). The type I BMP receptor substrates include a protein family, the Smad proteins, that play a central role in relaying the BMP signal from the receptor to target genes in the nucleus. Smad1, 5 and 8 are phosphorylated by BMP receptors in a ligand-dependent manner (Hoodless, P.A. et al. (1996) "MADR1, a MAD-related protein that functions in BMP2 signaling pathways", Cell 85, 489-500; Chen Y. et al. (1997) "Smad8 mediates the signaling of the receptor serine kinase", Proc. Natl Acad. Sci. USA 94, 12938-12943; Nishimura R. et al. (1998) "Smad5 and DPC4 are key molecules in mediating BMP-2-induced osteoblastic differentiation of the pluripotent mesenchymal precursor cell line C2C12", J. Biol. Chem. 273, 1872-1879). After release from the receptor, the phosphorylated Smad proteins associate with the related protein Smad4, which acts as a shared partner. This complex translocates into the nucleus and participates in gene transcription with other transcription factors (). A significant advancement about the understanding of in vivo functions of BMP ligands, receptors and signaling molecules has been achieved in recent years.  Figure 1 BMP signaling and its regulation. BMP signals are mediated by <span class="highlight\_termB">type</span> I and II BMP receptors and their downstream molecules Smad1, 5 and 8. Phosphorylated Smad1, 5 and 8 proteins form a complex with Smad4 and then are translocated into the nucleus where they interact with other transcription factors, such as Runx2 in osteoblasts. BMP signaling is regulated at different molecular levels: (1) Noggin and other cystine knot-containing BMP antagonists bind with BMP-2, 4 and 7 and block BMP signaling. Over-expression of noggin in mature osteoblasts causes <span class="highlight\_termB">osteoporosis</span> in mice (<citeref rid="bib9">Devlin et al., 2003</citeref>; <citeref rid="bib65">Wu et al., 2003</citeref>). (2) Smad6 binds <span class="highlight\_termB">type</span> I BMP receptor and prevents Smad1, 5 and 8 to be activated (<citeref rid="bib22">Imamura et al., 1997</citeref>). Over-expression of Smad6 in chondrocytes causes delays in chondrocyte differentiation and maturation (<citeref rid="bib21">Horiki et al., 2004</citeref>). (3) Tob interacts specifically with BMP activated Smad proteins and inhibits BMP signaling. In Tob null mutant mice, BMP signaling is enhanced and bone formation is increased (<citeref rid="bib71">Yoshida et al., 2000</citeref>). (4) Smurf1 is a Hect domain E3 ubiquitin ligase. It interacts with Smad1 and 5 and mediates the degradation of these Smad proteins (<citeref rid="bib76">Zhu et al., 1999</citeref>). (5) Smurf1 also recognizes bone-specific transcription factor Runx2 and mediates Runx2 degradation (<citeref rid="bib74">Zhao et al., 2003</citeref>). (6) Smurf1 also forms a complex with Smad6, is exported from the nucleus and targeted to the <span class="highlight\_termB">type</span> I BMP receptors for their degradation (<citeref rid="bib40">Murakami et al., 2003</citeref>). Over-expression of Smurf1 in osteoblasts inhibits postnatal bone formation in mice (<citeref rid="bib75">Zhao et al., 2004</citeref>). |
| 18256137 | Regulation of Fto/Ftm gene expression in mice and humans.Two recent, large whole-genome association studies (GWAS) in European populations have associated a approximately 47-kb region that contains part of the FTO gene with high body mass index (BMI). The functions of FTO and adjacent FTM in human biology are not clear. We examined expression of these genes in organs of mice segregating for monogenic obesity mutations, exposed to underfeeding/overfeeding, and to 4 degrees C. Fto/Ftm expression was reduced in mesenteric adipose tissue of mice segregating for the Ay, Lep ob, Lepr db, Cpe fat, or tub mutations, and there was a similar trend in other tissues. These effects were not due to adiposity per se. Hypothalamic Fto and Ftm expression were decreased by fasting in lean and obese animals and by cold exposure in lean mice. The fact that responses of Fto and Ftm expression to these manipulations were almost indistinguishable suggested that the genes might be coregulated. The putative overlapping regulatory region contains at least two canonical CUTL1 binding sites. One of these nominal CUTL1 sites includes rs8050136, a SNP associated with high body mass. The A allele of rs8050136 preferentially bound CUTL1[corrected] in human fibroblast DNA. 70% knockdown of CUTL1 expression in human fibroblasts decreased FTO and FTM expression by 90 and 65%, respectively. Animals and humans with various genetic interruptions of FTO or FTM have phenotypes reminiscent of aspects of the Bardet-Biedl obesity syndrome, a confirmed "ciliopathy." FTM has recently been shown to be a ciliary basal body protein. |
| 20384434 | Combining genetic markers and clinical risk factors improves the risk assessment of impaired glucose metabolism.BACKGROUND: Although several candidate gene polymorphisms (SNPs) have been associated with increased risk of type 2 diabetes mellitus (T2DM), relatively few studies have assessed the ability of T2DM candidate genes to assess the risk of impaired fasting glucose (IFG), impaired glucose tolerance (IGT), and T2DM beyond the information provided by clinical risk factors. OBJECTIVE: To test whether the inclusion of genetic markers in a regression model provides a better assessment of the risk of IFG, IGT, and T2DM than a model based only on non-genetic risk factors commonly assessed in clinical settings. METHODS: Subjects (n = 485; 213 parents, 272 offspring) from the Quebec Family Study, not known to haveT2DM, were measured for several risk factors and underwent an oral glucose tolerance test. Thirty-eight SNPs in 25 susceptibility/ candidate genes previously reported to be associated with T2DM were genotyped. In order to identify risk factors associated with IFG/IGT/T2DM, two logistic regression models were tested: a full model (FM) including age, sex, body mass index (BMI), systolic and diastolic blood pressure, smoking status, and the 38 SNPs; and a reduced model (RM), in which the SNPs were dropped, which allowed us to test the null-hypothesis that the markers are not associated with the risk of IFG/IGT/T2DM. Performances of the models were compared by using a likelihood ratio test and the receiver-operating characteristic curves (ROC).The area under the curve (AUC) was calculated from the ROC curve. RESULTS: The analyses showed that age (P < 0.0001), BMI (P < 0.0001), and six variants (IGF2BP2 rs4402960, P = 0.002; ADIPOQ+276 G>T, P = 0.004; UCP2Ala55Val, P = 0.01; CDKN2AI2B rs3731201, P = 0.02; rs495490, P = 0.02, and rsl 0811661, P = 0.03) were significantly associated with the risk of IFG/IGT/T2DM. Dropping genetic markers from the analysis significantly reduced the fit of the model to the data (chi-square = 38.98, P < 0.00001 contrasting RM to FM), suggesting that the genetic markers are significantly associated with the risk of IFG/IGT/T2DM. Furthermore, the AUC was higher for FM than for RM (0.85 (95% CI 0.81-0.89) versus 0.81 (95% CI 0.76-0.85), P = 0.004). CONCLUSION: Our results suggest that combining genetic markers with traditional clinical risk factors has the potential to improve our ability to assess the risk of complex diseases such as T2DM. |

Copyright © CoCiter 2011-2013. >>
Designed by QIAO Nan & HUANG Yi >>
Hanlab
