# Supplementary material for: Partial depletion of yolk during zebrafish embryogenesis changes the dynamics of methionine cycle and metabolic genes
Source: BMC Genomics. 2015 Jun 4;16(1):427. doi: 10.1186/s12864-015-1654-6 (PMC4455928; doi:10.1186/s12864-015-1654-6)
Supplement: Additional file 9: — CoCiter output of differentially expressed genes at 24 hpf. RNAseq outcome associated to the terms “hypertension”, “obesity”, “type 2 diabetes” and “osteoporosis”. [file 12864_2015_1654_MOESM9_ESM.html]

CoCiter v1.1


**CoCiter v1.1**

## Co-citation analysis on genes/terms

Home
Gene-Gene
Gene-Term
Term-Term
ViewJob
HanLab

## Your search results:

**Genes/Terms involved in mistake below are not calculated in this result.**
Gene 100329628 in the Gene Set is not included in the database of that species!

| Set | Genes/Terms | PubMed Count | CI | Significance |
| --- | --- | --- | --- | --- |
| GeneSet 110 genes | gstt1a (563972), slc2a3a (436916), angptl4 (492647), pck2 (406473), txnipa (368359), txnipb (448858), hbl4 (100137128), g6pca.2 (563180), mgea5 (324487), nod2 (777696), tgm2b (323856), cyp24a1 (100004700), gpd1b (325181), ppp1r3b (327285), si:dkey-8k3.2 (794635), pgm1 (394000), LOC100006895 (100006895), hdc (793609), lum (415149), LOC570613 (570613), krt18 (352912), tcn2l (407646), egln3 (406602), timm8b (793663), rcan1a (445222), gskip (406744), tp63 (260407), dmgdh (100141496), abat (378968), si:ch211-89f7.1 (558964), krt1-19d (664718), slc16a9a (795588), cdk13 (559027), ppp1r3cb (393807), zgc:77439 (378987), LOC563738 (563738), hao2 (393455), pik3ip1 (386643), myha (100149148), map1lc3b (322425), pabpc1b (393856), slc16a9b (445158), fam129bb (556540), si:ch1073-473i7.3 (793799), bbc3 (751763), ppp1r3ca (436649), psph (606663), ppdpfb (336303), gtpbp4 (334050), fam169ab (553309), gpd1c (406615), zgc:153018 (767701), LOC100536075 (100536075), uroc1 (556744), akr1a1a (445326), LOC100330677 (100330677), arrdc3b (447866), pfkfb4l (386663), LOC100538217 (100538217), ckmt1 (321892), thy1 (336617), si:ch211-251f6.6 (794692), cbx7a (550551), irx7 (140746), LOC100007703 (100007703), rcan2 (407988), zgc:101549 (492779), aqp12 (436844), LOC100536440 (100536440), LOC795591 (795591), zgc:174237 (100126125), mt (30282), ndrg1b (393665), zgc:113413 (503710), cldnf (81585), zgc:112994 (553736), gmpr (553639), rgra (550575), zgc:175094 (100000715), mt2 (100174951), LOC100535848 (100535848), si:dkey-238o13.4 (560780), klf11a (560787), itgb3a (564266), srpx2 (100332730), zgc:174688 (795020), rpe65b (100002865), zgc:173443 (100126130), atp1a1a.3 (64614), ponzr5 (556869), bloc1s6 (791215), zgc:113263 (503753), slc43a2a (393221), LOC793794 (793794), zgc:198419 (100006523), hoga1 (405806), dcxr (550282), hsd3b7 (327462), p4ha1b (100003675), zgc:110251 (553602), fam20a (564308), si:ch211-284e20.8 (100093712), zgc:174689 (795883), LOC100537515 (100537515), mpdu1a (447806), slc25a25a (406541), zgc:174938 (100126020), si:ch211-124e16.2 (566059), zgc:158494 (386720), abcb5 (798527) | 242 | 7.9248 | **p-value** 0.105 **permutation** 1000 x **adjusted CI** 1.3509 |
| TermSet 4 terms | type 2 diabetes, obesity, hypertension, osteoporosis |

- (p-value significance: \*\*\*: p < 0.001; \*\*: p < 0.005; \*: p < 0.01)
  

The one-to-all results.

| Gene v.s. all terms | Term v.s. all Genes |
| --- | --- |
| | Gene | Count | CI | | --- | --- | --- | |    gstt1a (563972) | 42 | 5.4263 | |    slc2a3a (436916) | 27 | 4.8074 | |    angptl4 (492647) | 24 | 4.6439 | |    pck2 (406473) | 24 | 4.6439 | |    txnipa (368359) | 20 | 4.3923 | |    txnipb (448858) | 20 | 4.3923 | |    hbl4 (100137128) | 18 | 4.2479 | |    g6pca.2 (563180) | 14 | 3.9069 | |    mgea5 (324487) | 10 | 3.4594 | |    nod2 (777696) | 8 | 3.1699 | |    tgm2b (323856) | 8 | 3.1699 | |    cyp24a1 (100004700) | 7 | 3.0 | |    gpd1b (325181) | 6 | 2.8074 | |    ppp1r3b (327285) | 5 | 2.585 | |    si:dkey-8k3.2 (794635) | 5 | 2.585 | |    pgm1 (394000) | 4 | 2.3219 | |    LOC100006895 (100006895) | 4 | 2.3219 | |    hdc (793609) | 4 | 2.3219 | |    lum (415149) | 3 | 2.0 | |    LOC570613 (570613) | 3 | 2.0 | |    krt18 (352912) | 3 | 2.0 | |    tcn2l (407646) | 3 | 2.0 | |    egln3 (406602) | 3 | 2.0 | |    timm8b (793663) | 2 | 1.585 | |    rcan1a (445222) | 2 | 1.585 | |    gskip (406744) | 2 | 1.585 | |    tp63 (260407) | 2 | 1.585 | |    dmgdh (100141496) | 2 | 1.585 | |    abat (378968) | 2 | 1.585 | |    si:ch211-89f7.1 (558964) | 1 | 1.0 | |    krt1-19d (664718) | 1 | 1.0 | |    slc16a9a (795588) | 1 | 1.0 | |    cdk13 (559027) | 1 | 1.0 | |    ppp1r3cb (393807) | 1 | 1.0 | |    zgc:77439 (378987) | 1 | 1.0 | |    LOC563738 (563738) | 1 | 1.0 | |    hao2 (393455) | 1 | 1.0 | |    pik3ip1 (386643) | 1 | 1.0 | |    myha (100149148) | 1 | 1.0 | |    map1lc3b (322425) | 1 | 1.0 | |    pabpc1b (393856) | 1 | 1.0 | |    slc16a9b (445158) | 1 | 1.0 | |    fam129bb (556540) | 1 | 1.0 | |    si:ch1073-473i7.3 (793799) | 1 | 1.0 | |    bbc3 (751763) | 1 | 1.0 | |    ppp1r3ca (436649) | 1 | 1.0 | |    psph (606663) | 1 | 1.0 | |    ppdpfb (336303) | 1 | 1.0 | |    gtpbp4 (334050) | 1 | 1.0 | |    fam169ab (553309) | 1 | 1.0 | |    gpd1c (406615) | 0 | 0.0 | |    zgc:153018 (767701) | 0 | 0.0 | |    LOC100536075 (100536075) | 0 | 0.0 | |    uroc1 (556744) | 0 | 0.0 | |    akr1a1a (445326) | 0 | 0.0 | |    LOC100330677 (100330677) | 0 | 0.0 | |    arrdc3b (447866) | 0 | 0.0 | |    pfkfb4l (386663) | 0 | 0.0 | |    LOC100538217 (100538217) | 0 | 0.0 | |    ckmt1 (321892) | 0 | 0.0 | |    thy1 (336617) | 0 | 0.0 | |    si:ch211-251f6.6 (794692) | 0 | 0.0 | |    cbx7a (550551) | 0 | 0.0 | |    irx7 (140746) | 0 | 0.0 | |    LOC100007703 (100007703) | 0 | 0.0 | |    rcan2 (407988) | 0 | 0.0 | |    zgc:101549 (492779) | 0 | 0.0 | |    aqp12 (436844) | 0 | 0.0 | |    LOC100536440 (100536440) | 0 | 0.0 | |    LOC795591 (795591) | 0 | 0.0 | |    zgc:174237 (100126125) | 0 | 0.0 | |    mt (30282) | 0 | 0.0 | |    ndrg1b (393665) | 0 | 0.0 | |    zgc:113413 (503710) | 0 | 0.0 | |    cldnf (81585) | 0 | 0.0 | |    zgc:112994 (553736) | 0 | 0.0 | |    gmpr (553639) | 0 | 0.0 | |    rgra (550575) | 0 | 0.0 | |    zgc:175094 (100000715) | 0 | 0.0 | |    mt2 (100174951) | 0 | 0.0 | |    LOC100535848 (100535848) | 0 | 0.0 | |    si:dkey-238o13.4 (560780) | 0 | 0.0 | |    klf11a (560787) | 0 | 0.0 | |    itgb3a (564266) | 0 | 0.0 | |    srpx2 (100332730) | 0 | 0.0 | |    zgc:174688 (795020) | 0 | 0.0 | |    rpe65b (100002865) | 0 | 0.0 | |    zgc:173443 (100126130) | 0 | 0.0 | |    atp1a1a.3 (64614) | 0 | 0.0 | |    ponzr5 (556869) | 0 | 0.0 | |    bloc1s6 (791215) | 0 | 0.0 | |    zgc:113263 (503753) | 0 | 0.0 | |    slc43a2a (393221) | 0 | 0.0 | |    LOC793794 (793794) | 0 | 0.0 | |    zgc:198419 (100006523) | 0 | 0.0 | |    hoga1 (405806) | 0 | 0.0 | |    dcxr (550282) | 0 | 0.0 | |    hsd3b7 (327462) | 0 | 0.0 | |    p4ha1b (100003675) | 0 | 0.0 | |    zgc:110251 (553602) | 0 | 0.0 | |    fam20a (564308) | 0 | 0.0 | |    si:ch211-284e20.8 (100093712) | 0 | 0.0 | |    zgc:174689 (795883) | 0 | 0.0 | |    LOC100537515 (100537515) | 0 | 0.0 | |    mpdu1a (447806) | 0 | 0.0 | |    slc25a25a (406541) | 0 | 0.0 | |    zgc:174938 (100126020) | 0 | 0.0 | |    si:ch211-124e16.2 (566059) | 0 | 0.0 | |    zgc:158494 (386720) | 0 | 0.0 | |    abcb5 (798527) | 0 | 0.0 | | | Term | Count | CI | | --- | --- | --- | |    type 2 diabetes | 113 | 6.8329 | |    obesity | 81 | 6.3576 | |    hypertension | 58 | 5.8826 | |    osteoporosis | 10 | 3.4594 | |

**100** of the **242** PubMed papers involved are listed below (sorted by relevance):

| PubMed ID | Title |
| --- | --- |
| 22236479 | Thioredoxin interacting protein genetic variation is associated with diabetes and hypertension in the Brazilian general population.OBJECTIVE: To investigate the relationship between TXNIP polymorphisms, diabetes and hypertension phenotypes in the Brazilian general population. METHODS: Five hundred seventy-six individuals randomly selected from the general urban population according to the MONICA-WHO project guidelines were phenotyped for cardiovascular risk factors. A second, independent, sample composed of 487 family-trios from a different site was also selected. Nine TXNIP polymorphisms were studied. The potential association between TXNIP variability and glucose-phenotypes in children was also explored. TXNIP expression was quantified by real-time PCR in 53 samples from human smooth muscle cells primary culture. RESULTS: TXNIP rs7211 and rs7212 polymorphisms were significantly associated with glucose and blood pressure related phenotypes. In multivariate logistic regression models the studied markers remained associated with diabetes even after adjustment for covariates. TXNIP rs7211 T/rs7212 G haplotype (present in approximately 17% of individuals) was significantly associated to diabetes in both samples. In children, the TXNIP rs7211 T/rs7212 G haplotype was associated with fasting insulin concentrations. Finally, cells harboring TXNIP rs7212 G allele presented higher TXNIP expression levels compared with carriers of TXNIP rs7212 CC genotype (p=0.02). CONCLUSION: Carriers of TXNIP genetic variants presented higher TXNIP expression, early signs of glucose homeostasis derangement and increased susceptibility to chronic metabolic conditions such as diabetes and hypertension. Our data suggest that genetic variation in the TXNIP gene may act as a "common ground" modulator of both traits: diabetes and hypertension. |
| 17979505 | Combined glutathione S-transferase T1 and M1 positive genotypes afford protection against type 2 diabetes in Japanese.INTRODUCTION: Diabetes mellitus is associated with an increased production of reactive oxygen species and a reduction in antioxidant defenses. The aim of this study is to determine the association between the incidence of Type 2 diabetes and gene polymorphisms of glutathione S-transferase (GST), which modulates oxidative stress. MATERIALS & METHODS: The associations between the incidence of Type 2 diabetes and the GSTT1 and GSTM1 genotypes were analyzed in 469 Japanese participants in a health-screening program. RESULTS: The clinical characteristics and smoking status were obtained from the health screening record. The incidence of diabetes was 1.5-fold higher in the GSTT1 and GSTM1 null (-) genotype than the GSTT1 and GSTM1 present (+) genotype, respectively. Although the effect of each null genotype was not significant, the combined GSTT1+/GSTM1+ genotypes conferred a significant reduction in risk of diabetes in comparison with the other combinations of genotypes (adjusted odds ratio [OR]: 0.30; 95% confidence interval [CI]: 0.12-0.71). In stratified analyses by smoking status, the incidence of diabetes was significantly higher in never-smokers with the GSTT1- genotype than those with the GSTT1+ genotype (OR: 2.85; 95% CI: 1.17-6.94) and increased significantly in current smokers (OR: 5.91; 95% CI: 1.96-17.88). The effect of the GSTM1- genotype was significant only in current smokers. CONCLUSION: This study demonstrated that the GSTT1- and GSTT1-/GSTM1- genotypes are independent risk factors for development of Type 2 diabetes regardless of the smoking status of the patient, and that these genotypes and current smoking were interactively associated with the incidence of Type 2 diabetes. |
| 17381501 | Genetic variation in thioredoxin interacting protein (TXNIP) is associated with hypertriglyceridaemia and blood pressure in diabetes mellitus.AIMS: Thioredoxin interacting protein (TXNIP) is an attractive candidate gene for diabetes or diabetic dyslipidaemia, since TXNIP is the strongest glucose-responsive gene in pancreatic B-cells, TXNIP deficiency in a mouse model is associated with hyperlipidaemia and TXNIP is located in the 1q21-1q23 chromosomal Type 2 diabetes mellitus (DM) locus. We set out to investigate whether metabolic effects of TXNIP that were previously reported in a murine model are also relevant in human Type 2 DM. METHODS: The frequency distribution of a 3' UTR single nucleotide polymorphism (SNP) in TXNIP was investigated in subjects with normal glucose tolerance (NGT; n = 379), impaired glucose tolerance (IGT; n = 228) and Type 2 DM (n = 230). Metabolic data were used to determine the effect of this SNP on parameters associated with lipid and glucose metabolism. RESULTS: The frequency of the TXNIP variation did not differ between groups, but within the group of diabetic subjects, carriers of the TXNIP-T variant had 1.6-fold higher triglyceride concentrations (P = 0.015; n = 136) and a 5.5-mmHg higher diastolic blood pressure (P = 0.02; n = 212) than homozygous carriers of the common C-allele, whereas in non-diabetic subjects fasting glucose was 0.26 mmol/l lower (P = 0.002; n = 478) in carriers of the T-allele. Moreover, a significant interaction between plasma glucose concentrations and TXNIP polymorphism on plasma triglycerides was observed (P = 0.012; n = 544). CONCLUSION: This is the first report to implicate TXNIP in a human disorder of energy metabolism, Type 2 diabetes. The effect of TXNIP on triglycerides is influenced by plasma glucose concentrations, suggesting that the biological relevance of TXNIP variations may be particularly relevant in recurrent episodes of hyperglycaemia. |
| 19258488 | Diabetes induces and calcium channel blockers prevent cardiac expression of proapoptotic thioredoxin-interacting protein.Cardiomyocyte apoptosis is a critical process in the pathogenesis of ischemic and diabetic cardiomyopathy, but the mechanisms are not fully understood. Thioredoxin-interacting protein (TXNIP) has recently been shown to have deleterious effects in the cardiovascular system and we therefore investigated whether it may also play a role in diabetes-associated cardiomyocyte apoptosis. In fact, TXNIP expression was increased in H9C2 cardiomyocytes incubated at high glucose, and cardiac expression of TXNIP and cleaved caspase-3 were also elevated in vivo in streptozotocin- and obesity-induced diabetic mice. Together, these findings not only suggest that TXNIP is involved in diabetic cardiomyopathy but also that it may represent a novel therapeutic target. Surprisingly, testing putative TXNIP modulators revealed that calcium channel blockers reduce cardiomyocyte TXNIP transcription and protein levels in a dose-dependent manner. Oral administration of verapamil for 3 wk also reduced cardiac TXNIP expression in mice even in the face of severe diabetes, and these reduced TXNIP levels were associated with decreased apoptosis. To determine whether lack of TXNIP can mimic the verapamil-induced decrease in apoptosis, we used TXNIP-deficient HcB-19 mice, harboring a natural nonsense mutation in the TXNIP gene. Interestingly, we found significantly reduced cleaved caspase-3 levels in HcB-19 hearts, suggesting that TXNIP plays a critical role in cardiac apoptosis and that the verapamil effects were mediated by TXNIP reduction. Thus our results suggest that TXNIP reduction is a powerful target to enhance cardiomyocyte survival and that agents such as calcium channel blockers may be useful in trying to achieve this goal and prevent diabetic cardiomyopathy. |
| 20299477 | Deletion of the alpha-arrestin protein Txnip in mice promotes adiposity and adipogenesis while preserving insulin sensitivity.OBJECTIVE: Thioredoxin interacting protein (Txnip), a regulator of cellular oxidative stress, is induced by hyperglycemia and inhibits glucose uptake into fat and muscle, suggesting a role for Txnip in type 2 diabetes pathogenesis. Here, we tested the hypothesis that Txnip-null (knockout) mice are protected from insulin resistance induced by a high-fat diet. RESEARCH DESIGN AND METHODS: Txnip gene-deleted (knockout) mice and age-matched wild-type littermate control mice were maintained on a standard chow diet or subjected to 4 weeks of high-fat feeding. Mice were assessed for body composition, fat development, energy balance, and insulin responsiveness. Adipogenesis was measured from ex vivo fat preparations, and in mouse embryonic fibroblasts (MEFs) and 3T3-L1 preadipocytes after forced manipulation of Txnip expression. RESULTS: Txnip knockout mice gained significantly more adipose mass than controls due to a primary increase in both calorie consumption and adipogenesis. Despite increased fat mass, Txnip knockout mice were markedly more insulin sensitive than controls, and augmented glucose transport was identified in both adipose and skeletal muscle. RNA interference gene-silenced preadipocytes and Txnip(-/-) MEFs were markedly adipogenic, whereas Txnip overexpression impaired adipocyte differentiation. As increased adipogenesis and insulin sensitivity suggested aspects of augmented peroxisome proliferator-activated receptor-gamma (PPARgamma) response, we investigated Txnip's regulation of PPARgamma function; manipulation of Txnip expression directly regulated PPARgamma expression and activity. CONCLUSIONS: Txnip deletion promotes adiposity in the face of high-fat caloric excess; however, loss of this alpha-arrestin protein simultaneously enhances insulin responsiveness in fat and skeletal muscle, revealing Txnip as a novel mediator of insulin resistance and a regulator of adipogenesis. |
| 22058002 | Influence of glutathione S-transferase polymorphisms on type-2 diabetes mellitus risk.Glutathione S-transferase (GST) protects cells against oxidative stress. We evaluated the effect of genetic polymorphisms of the GST gene family on the risk of developing type-2 diabetes mellitus and on glycemic control. We also investigated the effects of smoking combined with these polymorphisms on type-2 diabetes mellitus risk. We enrolled 100 type-2 diabetes mellitus patients and 100 healthy controls matched for age, gender and origin, from the Sinai area of Egypt. Fasting serum glucose, HbA(1c) and lipid profiles were determined. Two polymorphisms were identified by multiplex PCR within the GST genes: GSTM1 and GSTT1. The proportion of the GSTT1- and GSTM1-null genotypes was significantly greater in diabetic patients when compared to controls. Patients carrying both null polymorphisms had a 3.17-fold increased risk of having type-2 diabetes mellitus compared to those with normal genotypes of these two genes (P = 0.009). Additionally, patients with the GSTT1-null genotype had higher levels of triglycerides and very low-density lipoprotein cholesterol compared to those with the GSTT1-present genotype. On the other hand, patients with the GSTM1- null genotype had significantly higher levels of HbA(1c) and significantly higher diastolic blood pressure compared to those with the GSTM1- present genotype. The interaction between these genotypes and smoking status was not significant. These results give evidence that the GSTT1- and GSTM1-null genotypes, alone or combined, are associated with increased risk of type-2 diabetes mellitus, regardless of smoking status. Only the GSTM1-null genotype had an effect on glycemic control. |
| 22068616 | Reduction of insulin signaling upregulates angiopoietin-like protein 4 through elevated free fatty acids in diabetic mice.BACKGROUND: Angiopoietin-like protein 4 (Angptl4) is thought to cause an increase in serum triglyceride levels. In the present study, we elucidated Angptl4 expression in the mouse models of type 1 and type 2 diabetes mellitus, and investigated the possible mechanisms involved. METHODS: Type 1 diabetes was induced in C57BL/6 J mice by treating them with streptozotocin (STZ). Type 2 diabetes was induced by feeding the mice a high-fat diet (HFD) for 18 weeks. RESULTS: The levels of Angptl4 mRNA expression in liver, white adipose tissue (WAT), and brown adipose tissue (BAT) were found to increase in the STZ diabetic mice relative to control mice. This effect was attenuated by insulin administration. In the HFD diabetic mice, the Angptl4 mRNA expression levels were increased in liver, WAT, and BAT. Treatment with metformin for 4 weeks attenuated the increased levels of Angptl4 mRNA. Fatty acids (FAs) such as palmitate and linoleate induced Angptl4 mRNA expression in H4IIE hepatoma cells and 3T3-L1 adipocytes. Treatment with insulin but not metformin attenuated FA-induced Angptl4 mRNA expression in H4IIE. Both insulin and metformin did not influence the effect of FAs in 3T3-L1 cells. CONCLUSION: These observations demonstrated that Angptl4 mRNA expression was increased through the elevated free FAs in diabetic mice. |
| 23152583 | Posttranslational regulation of thioredoxin-interacting protein.Thioredoxin-interacting protein (Txnip) is a metabolic regulator, which modulates insulin sensitivity and likely plays a role in type 2 diabetes. We studied the regulation of Txnip in 3T3-L1 adipocytes. Cells were incubated under different conditions and Txnip was measured by immunoblotting. We confirmed that high glucose markedly increases Txnip expression by promoting transcription. Insulin decreases Txnip protein levels. Rapamycin under most conditions decreased Txnip, suggesting that mTOR complex-1 is involved. The acute effects of insulin are mainly posttranscriptional; insulin (100 nM) accelerates Txnip degradation more than tenfold. This effect is cell type specific. It works in adipocytes, preadipocytes and in L6 myotubes but not in HepG2 or in HEK 293 cells or in a pancreatic beta-cell line. The ubiquitin/proteasome pathway is involved. Degradation of Txnip occurred within 15 min in the presence of 3 nM insulin and overnight with 0.6 nM insulin. Proteasomal Txnip degradation is not mediated by a cysteine protease or an anti-calpain enzyme. Okadaic acid (OKA), an inhibitor of phosphoprotein phosphatases (pp), markedly reduced Txnip protein and stimulated its further decrease by insulin. The latter occurred after incubation with 1 or 1000 nM OKA, suggesting that insulin enhances the phosphorylation of a pp2A substrate. Incubation with 0.1 muM Wortmannin, a PI3 kinase inhibitor, increased Txnip protein twofold and significantly inhibited its insulin-induced decrease. Thus, while OKA mimics the effect of insulin, Wortmannin opposes it. In summary, insulin stimulates Txnip degradation by a PI3 kinase-dependent mechanism, which activates the ubiquitin/proteasome pathway and likely serves to mitigate insulin resistance. |
| 10866049 | Glucose-6-phosphatase flux in vitro is increased in type 2 diabetes.Despite the effects of hyperinsulinemia and hyperglycemia, 2 factors known to inhibit endogenous glucose production (EGP) in nondiabetic subjects, increased EGP is a consistent feature of type 2 diabetes. Recent studies have suggested that increased glucose-6-phosphatase (G6Pase) and/or decreased glucokinase (GK) may explain the increase in EGP. However, no studies to date have clearly established this relationship in type 2 diabetes. The present studies were designed to determine rates of EGP and the activities of G6Pase and GK in obese patients scheduled for gastric bypass surgery. The study group consisted of 14 obese nondiabetic subjects and 13 patients with type 2 diabetes (BMI 53.7 +/- 2.4 vs. 50.1 +/- 1.6 kg/m2). Rates of EGP were determined after an overnight fast with a 4-h infusion of [6,6]-D-glucose, and they were significantly higher in the type 2 diabetic patients (85.9 +/- 10.0 vs. 137.8 +/- 14.4 mg x m(-2) x min(-1), P < 0.001) despite greater plasma glucose (5.1 +/- 0.1 vs. 12.0 +/- 1.1 mmol/l) and similar insulin concentrations (130.8 +/- 19.8 vs. 112.8 +/- 16.2 pmol/l, NS). Moreover, resistance to insulin-induced suppression of EGP was observed in the patients with type 2 diabetes when insulin concentrations were increased from approximately 120 to 180 pmol/l. Hepatic G6Pase activity determined from freshly isolated microsomes was significantly increased in the type 2 diabetic patients compared with the obese control subjects (0.16 +/- 0.02 vs. 0.09 +/- 0.01 micromol x min(-1) x mg(-1) protein, P < 0.02), whereas levels of GK were decreased (1.20 +/- 0.16 vs. 2.01 +/- 0.01 micromol x min(-1) x mg(-1) protein, P < 0.01). Net flux through G6Pase was significantly increased in type 2 diabetic patients (P < 0.01). We conclude that increased EGP is mediated in part by increased G6Pase flux in type 2 diabetes. |
| 16132948 | Association of a polymorphism in the gene encoding phosphoenolpyruvate carboxykinase 1 with high-density lipoprotein and triglyceride levels.AIMS/HYPOTHESIS: Phosphoenolpyruvate carboxykinase (PCK) is the key enzyme involved in the regulation of gluconeogenesis. The aim of this study was to identify genetic polymorphisms in potential candidate genes for type 2 diabetes by sequencing all exons in the PCK genes (PCK1 and PCK2), and examining the association with type 2 diabetes and diabetic phenotypes in a Korean population (775 type 2 diabetic patients and 316 normal control subjects). MATERIALS AND METHODS: Twenty-two polymorphisms in PCK1 and PCK2 were identified in a Korean population (n=24) by direct DNA sequencing. The TaqMan genotyping method was applied for genotyping the remainder of the study population. Associations of PCK polymorphisms with the risk of type 2 diabetes and diabetic phenotypes were analysed using logistic and multiple regressions, adjusting for age, sex and BMI. RESULTS: Although no significant associations between the genetic polymorphisms in PCK genes and the risk of type 2 diabetes were detected, in further haplotype analysis, one of the common haplotypes, PCK1 ht3, revealed susceptibility to type 2 diabetes (p=0.006). One 3' untranslated region (UTR) single nucleotide polymorphism (SNP) also showed an association with HDL levels among non-diabetic control subjects: individuals homozygous for the major allele (T/T) had the lowest HDL level (1.11+/-0.32 mmol/l), heterozygotes (T/C) had an intermediate level (1.27+/-0.37 mmol/l), and those homozygous for the minor allele (C/C) had the highest level (1.39+/-0.28 mmol/l) (p=0.000003). This 3' UTR SNP was also associated with triglyceride levels, with a lower triglyceride level observed among individuals who were homozygous for the minor allele (C/C) than among those who were not. CONCLUSIONS/INTERPRETATION: The strong genetic association of HDL and triglyceride levels with variation/haplotype information identified in this study would be useful for further genetic epidemiological studies of this important gene. |
| 21596930 | Serum angiopoietin-like 4 protein levels and expression in adipose tissue are inversely correlated with obesity in monozygotic twins.Animal studies have suggested that angiopoietin-like 4 (Angptl4) regulates adiposity through central and peripheral mechanisms. The aim of this study was to investigate whether serum concentration and adipose tissue expression of Angptl4 are associated with obesity-related parameters in humans. Altogether, 75 dizygotic (DZ) and 46 monozygotic (MZ) twin pairs were studied, from the FinnTwin12 and FinnTwin16 cohorts. Among the MZ pairs, 21 were discordant for body mass index (BMI) (intra-pair BMI-difference >2.5 kg/m(2), age 23-33 years). Serum Angptl4 (s-Angptl4) levels were measured by ELISA, and adipose tissue gene expression was analyzed by genome-wide transcript profiling. In MZ twin pairs discordant for BMI, s-Angptl4 and adipose tissue ANGPTL4 mRNA (at-ANGPTL4) levels were significantly decreased (P = 0.04 and P = 0.03, respectively) in obese twins as compared with their nonobese cotwins. In all twins, intra-pair differences in s-Angptl4 levels were inversely correlated with intra-pair differences in BMI (r = -0.27, P = 0.003). In individual MZ twins, at-ANGPTL4 expression was inversely correlated with BMI (r = -0.44, P = 0.001) and positively correlated with at-LIPE (r = 0.24, P = 0.01) and at-ABHD5 (r = 0.41, P = 0.005) expression. Our results demonstrated that variation in Angptl4 concentration was only modestly accounted for by genetic factors and suggest a role for Angptl4 in acquired obesity in humans. |
| 16620271 | Association of the promoter polymorphism -232C/G of the phosphoenolpyruvate carboxykinase gene (PCK1) with Type 2 diabetes mellitus.AIMS: The phosphoenolpyruvate carboxykinase gene (PCK1) is a potential candidate gene in the pathogenesis of Type 2 diabetes mellitus. A -232C/G promoter polymorphism of PCK1 has been associated with an increased risk of Type 2 diabetes in a Canadian population. The purpose of the present study was to examine this association in a German Caucasian population. METHODS: We investigated 397 subjects with Type 2 diabetes [227 men, 170 women, age 63 +/- 11 years, body mass index (BMI) 28.7 +/- 5.1 kg/m2] and 431 control subjects without diabetes (247 men, 184 women, age 64 +/- 7 years, BMI 26.5 +/- 3.7 kg/m2) matched for sex and age. RESULTS: In the diabetic and control groups, the CC genotype frequencies were 18.1 and 18.3%, the CG 48.6 and 48.7% and the GG 33.2 and 32.9%, respectively (P = 0.995). The allelic frequencies were 0.51 and 0.57 for the G allele and 0.49 and 0.43 for the C allele, respectively. In a logistic regression model only BMI and family history, but not the polymorphism, were predictors of Type 2 diabetes. In both the control and diabetic subjects, there were no significant differences in BMI or blood pressure between the groups with or without the polymorphism. The variant also had no significant influence on the presence of atherosclerotic disease, while the influence of other known cardiovascular risk factors was confirmed. CONCLUSIONS: The present data suggest that, in a German Caucasian population, the -232C/G polymorphism of the PEPCK gene is not associated with Type 2 diabetes. |
| 23176178 | Linking nutritional regulation of Angptl4, Gpihbp1, and Lmf1 to lipoprotein lipase activity in rodent adipose tissue.BACKGROUND: Lipoprotein lipase (LPL) hydrolyzes triglycerides in lipoproteins and makes fatty acids available for tissue metabolism. The activity of the enzyme is modulated in a tissue specific manner by interaction with other proteins. We have studied how feeding/fasting and some related perturbations affect the expression, in rat adipose tissue, of three such proteins, LMF1, an ER protein necessary for folding of LPL into its active dimeric form, the endogenous LPL inhibitor ANGPTL4, and GPIHBP1, that transfers LPL across the endothelium. RESULTS: The system underwent moderate circadian oscillations, for LPL in phase with food intake, for ANGPTL4 and GPIHBP1 in the opposite direction. Studies with cycloheximide showed that whereas LPL protein turns over rapidly, ANGPTL4 protein turns over more slowly. Studies with the transcription blocker Actinomycin D showed that transcripts for ANGPTL4 and GPIHBP1, but not LMF1 or LPL, turn over rapidly. When food was withdrawn the expression of ANGPTL4 and GPIHBP1 increased rapidly, and LPL activity decreased. On re-feeding and after injection of insulin the expression of ANGPTL4 and GPIHBP1 decreased rapidly, and LPL activity increased. In ANGPTL4(-/-) mice adipose tissue LPL activity did not show these responses. In old, obese rats that showed signs of insulin resistance, the responses of ANGPTL4 and GPIHBP1 mRNA and of LPL activity were severely blunted (at 26 weeks of age) or almost abolished (at 52 weeks of age). CONCLUSIONS: This study demonstrates directly that ANGPTL4 is necessary for rapid modulation of LPL activity in adipose tissue. ANGPTL4 message levels responded very rapidly to changes in the nutritional state. LPL activity always changed in the opposite direction. This did not happen in Angptl4(-/-) mice. GPIHBP1 message levels also changed rapidly and in the same direction as ANGPTL4, i.e. increased on fasting when LPL activity decreased. This was unexpected because GPIHBP1 is known to stabilize LPL. The plasticity of the LPL system is severely blunted or completely lost in insulin resistant rats. |
| 23635032 | Age-of-onset-dependent influence of NOD2 gene variants on disease behaviour and treatment in Crohn's disease.BACKGROUND: Influence of genetic variants in the NOD2 gene may play a more important role in disease activity, behaviour and treatment of pediatric- than adult-onset Crohn's disease (CD). METHODS: 85 pediatric- and 117 adult-onset CD patients were tested for the three main NOD2 CD-associated variants (p.R702W, p.G908R and p.10007fs) and clinical data of at least two years of follow-up were compared regarding disease behaviour and activity, response to therapy and bone mineral density (BMD). RESULTS: Chronic active and moderate to severe course of CD is associated in patients with pediatric-onset (p=0.0001) and NOD2 variant alleles (p=0.0001). In pediatric-onset CD the average PCDAI-Score was significantly higher in patients carrying NOD2 variants (p=0.0008). In addition, underweight during course of the disease (p=0.012) was associated with NOD2 variants. Interestingly, osteoporosis was found more frequently in patients carrying NOD2 variant alleles (p=0.033), especially in pediatric-onset CD patients with homozygous NOD2 variants (p=0.037). Accordingly, low BMD in pediatric-onset CD is associated with a higher PCDAI (p=0.0092), chronic active disease (p=0.0148), underweight at diagnosis (p=0.0271) and during follow-up (p=0.0109). Furthermore, pediatric-onset CD patients with NOD2 variants are more frequently steroid-dependent or refractory (p=0.048) and need long-term immunosuppressive therapy (p=0.0213). CONCLUSIONS: These data suggests that the presence of any of the main NOD2 variants in CD is associated with osteoporosis and an age of onset dependent influence towards underweight, higher disease activity and a more intensive immunosuppressive therapy. This observation supports the idea for an early intensive treatment strategy in children and adolescent CD patients with NOD2 gene variants. |
| 19628874 | Angiopoietin-like 4 (ANGPTL4, fasting-induced adipose factor) is a direct glucocorticoid receptor target and participates in glucocorticoid-regulated triglyceride metabolism.Glucocorticoids are important regulators of lipid homeostasis, and chronically elevated glucocorticoid levels induce hypertriglyceridemia, hepatic steatosis, and visceral obesity. The occupied glucocorticoid receptor (GR) is a transcription factor. However, those genes regulating lipid metabolism under GR control are not fully known. Angiopoietin-like 4 (ANGPTL4, fasting-induced adipose factor), a protein inhibitor of lipoprotein lipase, is synthesized and secreted during fasting, when circulating glucocorticoid levels are physiologically increased. We therefore tested whether the ANGPTL4 gene (Angptl4) is transcriptionally controlled by GR. We show that treatment with the synthetic glucocorticoid dexamethasone increased Angptl4 mRNA levels in primary hepatocytes and adipocytes (2-3-fold) and in the livers and white adipose tissue of mice (approximately 4-fold). We tested the mechanism of this increase in H4IIE hepatoma cells and found that dexamethasone treatment increased the transcriptional rate of Angptl4. Using bioinformatics and chromatin immunoprecipitation, we identified a GR binding site within the rat Angptl4 sequence. A reporter plasmid containing this site was markedly activated by dexamethasone, indicative of a functional glucocorticoid response element. Dexamethasone treatment also increased histone H4 acetylation and DNase I accessibility in genomic regions near this site, further supporting that it is a glucocorticoid response element. Glucocorticoids promote the flux of triglycerides from white adipose tissue to liver. We found that mice lacking ANGPTL4 (Angptl4(-/-)) had reductions in dexamethasone-induced hypertriglyceridemia and hepatic steatosis, suggesting that ANGPTL4 is required for this flux. Overall, we establish that ANGPTL4 is a direct GR target that participates in glucocorticoid-regulated triglyceride metabolism. |
| 19782926 | The role of glutathione S- transferase M1 and T1 gene polymorphisms and oxidative stress-related parameters in Egyptian patients with essential hypertension.BACKGROUND: Essential hypertension is a complex, multifactorial, polygenic disease in which the underlying genetic components remain unknown. Glutathione S-transferase (GST) enzyme is involved in detoxification of reactive oxygen species. This study aimed to investigate GSTM1 and GSTT1 gene polymorphisms in Egyptian essential hypertensive patients and their relationship with oxidative stress-related parameters. METHODS: The study included 40 newly-diagnosed, untreated, essential hypertensive patients and 40 normotensive subjects. Plasma levels of malondialdehyde (MDA), and nitrate/nitrite and erythrocyte reduced glutathione (GSH), activities of catalase (CAT), superoxide dismutase (SOD), glutathione peroxidase (GSH-Px), and glutathione S-transferase (GST) were measured. Genotyping for GSTM1 and GSTT1 was performed. RESULTS: The frequency of GSTM1+ve/GSTT1+ve in hypertensives (5%) was lower than in normotensives (37.5%).The frequency of GSTM1-ve/GSTT1-ve was elevated in hypertensives (35%) as compared to normotensives (7.5%). Plasma MDA was higher and nitrate/nitrite was lower in hypertensives than in normotensives. Erythrocyte GSH, activities of CAT, SOD, GSH-Px, and GST of hypertensives were lower than normotensives. Moreover, GST activity was lower in subjects with GSTM1-ve/GSTT1-ve than in those with GSTM1+ve/GSTT1+ve. In hypertensives, both systolic and diastolic blood pressures were negatively correlated with activities of CAT, GSH-Px, and GST. CONCLUSIONS: GSTM1-ve/GSTT1-ve is a potential genetic factor to predict development of essential hypertension and permit early therapeutic intervention. The significant association between blood pressure and oxidative stress-related parameters indicates the pathogenic role of oxidative stress in hypertension. Antioxidants could be useful in the management of essential hypertension to prevent progressive deterioration and target organ damage however, further studies involving long-term clinical trials may help to assess the efficacy of these therapeutic agents. |
| 22048269 | Opposite effects of GSTM1--and GSTT1: gene deletion variants on bone mineral density.Oxidative stress is associated with osteoporosis. The glutathione S-transferases form the major detoxifying group of enzymes responsible for eliminating products of oxidative stress. We have therefore proposed GSTM1 and GSTT1 genes as candidates for studying the genetics of osteoporosis. The aim of the present study was to examine possible association of GSTM1 and GSTT1 gene deletion polymorphisms, alone or in combination, with bone mineral density at femoral neck (BMD\_fn), lumbar spine (BMD\_ls) and total hip (BMD\_th) in Slovenian elderly women and men.GSTM1 and GSTT1 gene deletion polymorphisms in 712 elderly people were analyzed using the triplex PCR method for the presence of GSTM1 and GSTT1 gene segments. BMD\_fn, BMD\_ls and BMD\_th were measured by the dual-energy X-ray absorptiometry (DEXA) method. Results were analyzed using univariate statistic model adjusted for sex, body mass index (BMI) and age. Our results showed the significant differences in BMD\_th, BMD\_ls and BMD\_fn values (p=0.031, 0.017 and 0.023, respectively) in subgroups of GSTT1 gene deletion polymorphism. For GSTM1 gene deletion polymorphism borderline significant association was found with BMD\_ls (p=0.100). Furthermore, subjects with homozygous deletion of GSTT1 gene showed higher BMD values on all measured skeletal sites and, in contrast, subjects with homozygous deletion of GSTM1 gene showed lower BMD values. Moreover, a gene-gene interaction study showed significant association of GSTM1-null and GSTT1-null polymorphisms with BMD\_ls values (p=0.044). Carriers with a combination of the presence of GSTT1 gene and the homozygous absence of GSTM1 gene fragment were associated with the lower BMD values at all skeletal sites. The significant association of combination of GSTT1 gene presence and homozygous absence of GSTM1 gene with BMD was demonstrated, suggesting that it could be used, if validated in other studies, as genetic marker for low BMD. |
| 21709421 | Fatty acids and hypoxia stimulate the expression and secretion of the adipokine ANGPTL4 (angiopoietin-like protein 4/ fasting-induced adipose factor) by human adipocytes.BACKGROUND/AIMS: Hypoxia occurs in white adipose tissue in obesity, modulating the expression and release of specific inflammation-related adipokines. ANGPTL4 (angiopoietin-like protein 4/fasting-induced adipose factor), which is implicated in angiogenesis, lipid metabolism and glucose homeostasis, is a major hypoxia-sensitive gene; recent studies indicate that ANGPTL4 expression is also regulated by fatty acids. We have examined the effects of hypoxia and fatty acids, alone and together, on the expression and release of ANGPTL4 by human adipocytes. METHODS: Human adipocytes were differentiated and incubated with fatty acids (250 muM) in normoxia (21% O(2)) or hypoxia (1% O(2)). ANGPTL4 mRNA was measured by real-time PCR and the protein in the medium determined by ELISA. RESULTS: In normoxia, ANGPTL4 gene expression was upregulated by palmitic, oleic, arachidonic and eicosapentaenoic acids, and ANGPTL4 release was increased. In contrast, there was no effect of lauric or myristic acids. Hypoxia alone increased the expression and secretion of ANGPTL4, and lauric, myristic, arachidonic and eicosapentaenoic acids each further increased expression and release in hypoxic adipocytes. CONCLUSION: The expression and secretion of ANGPTL4 by human adipocytes is upregulated by both hypoxia and fatty acids. The stimulatory effect of fatty acids on ANGPTL4 production is augmented under hypoxic conditions. |
| 21728793 | Glutathione S-transferase M1 and T1 gene polymorphisms and risk of hypertension in tea garden workers of North-East India.AIMS: Polymorphisms of genes encoding phase II metabolic enzymes, for example, glutathione S-transferase, have been linked with hypertension. The present study aimed at finding out the association between GSTM1 and GSTT1 polymorphism and hypertension in a population from North-East India. MATERIALS AND METHODS: We carried out a case-control study in tea garden workers of Assam. A total of 223 hypertensive cases and 236 normotensive control subjects were recruited with a record of socio-demographic information, blood pressure, and anthropometric data. Fasting venous blood samples from all subjects were obtained and subjected to DNA extraction and polymerase chain reaction to detect polymorphism of the GSTM1 and GSTT1 genes. RESULTS: The null genotype was prevalent in 38.1% and 28.1% of the study participants (cases and controls) for GSTM1 and GSTT1 genes, respectively. Hypertensive subjects had a significantly higher prevalence of the GSTM1 null genotype (43% vs. 33.5%, than normotensive control subjects, p=0.035). Association between the GSTM1 null genotype and hypertension was significant in younger subjects. Tobacco users with the GSTT1 null genotype were at an increased risk for hypertension. CONCLUSION: The knowledge of GSTM1 and GSTT1 variant status will be useful to predict the risk of hypertension in a population. |
| 22377702 | GSTT1 null genotype is a risk factor for diabetic retinopathy in Caucasians with type 2 diabetes, whereas GSTM1 null genotype might confer protection against retinopathy.AIM: Substantial data indicate that oxidative stress is involved in the development of diabetic retinopathy (DR). The aim of the present study was to investigate whether the genetic polymorphisms: polymorphic deletions of glutathione S-transferases M1 (GSTM1) and T1 (GSTT1) and Ile105Val of the GSTP1 are associated with DR in Slovenian patients with type 2 diabetes. METHODS: In this cross sectional case-control study 604 unrelated Slovene subjects (Caucasians) with type 2 diabetes mellitus were enrolled: 284 patients with DR (cases) and the control group of 320 subjects with type 2 diabetes of more than 10 years' duration who had no clinical signs of DR. Genotypes were determined by polymerase chain reaction (PCR) and restriction fragment length polymorphism (RFLP). RESULTS: In our study, the deletion of the GSTM1 was found less frequent in cases with DR than in the controls (27.5% versus 44.4%; P < 0.001), whereas the deletion of GSTT1 was found significantly more often in cases than in the controls (49.3% versus 29.7%; P < 0.001). We did not find statistically significant differences in the genotype distribution in GSTP1 (Ile105Val) polymorphism between cases and controls (40.5% versus 46.0%). CONCLUSIONS: We may conclude that individuals homozygous for the deletion of GSTT1 are at an approximately 2-fold-greater risk of DR, whereas the GSTM1 deficiency is associated with lower frequency of DR in type 2 diabetics. |
| 22858312 | Association of manganese superoxide dismutase and glutathione S-transferases genotypes with myocardial infarction in patients with type 2 diabetes mellitus.AIM: In the present study we investigated the association between genetic polymorphisms with functional effects on redox regulation: Val16Ala of manganese superoxide dismutase (MnSOD), polymorphic deletions of glutathione S-transferases M1 (GSTM1) and T1 (GSTT1) and Ile105Val of glutathione S-transferase P1 (GSTP1) and myocardial infarction (MI) in a group of patients with type 2 diabetes mellitus. METHODS: The study population consisted of 463 Caucasian subjects with type 2 diabetes mellitus of more than 10 years' duration: 206 patients with MI and 257 patients with no history of coronary artery disease (CAD). Genotypes were determined by polymerase chain reaction (PCR) with restriction fragment length polymorphism (RFLP) and with multiplex PCR. RESULTS: The genotype distributions of tested single nucleotide polymorphisms did not show significant difference between cases and controls. After adjustment for age, gender, smoking, BMI, duration of diabetes and lipid parameters carriers of GSTM1/GSTT1-null haplotype showed an increased risk for MI (OR=3.22, 95% CI 1.37-5.04, p=0.03). CONCLUSIONS: The GSTM1/GSTT1 haplotype might be a genetic risk factor for MI in patients with type 2 diabetes mellitus. |
| 23119081 | CYP24A1 exacerbated activity during diabetes contributes to kidney tubular apoptosis via caspase-3 increased expression and activation.Decreases in circulating 25,hydroxyl-vitamin D3 (25 OH D3) and 1,25,dihydroxyl-vitamin D3 (1,25 (OH)2 D3) have been extensively documented in patients with type 2 diabetes. Nevertheless, the molecular reasons behind this drop, and whether it is a cause or an effect of disease progression is still poorly understood. With the skin and the liver, the kidney is one of the most important sites for vitamin D metabolism. Previous studies have also shown that CYP24A1 (an enzyme implicated in vitamin D metabolism), might play an important role in furthering the progression of kidney lesions during diabetic nephropathy. In this study we show a link between CYP24A1 increase and senescence followed by apoptosis induction in the renal proximal tubules of diabetic kidneys. We show that CYP24A1 expression was increased during diabetic nephropathy progression. This increase derived from protein kinase C activation and increased H(2)O(2) cellular production. CYP24A1 increase had a major impact on cellular phenotype, by pushing cells into senescence, and later into apoptosis. Our data suggest that control of CYP24A1 increase during diabetes has a beneficial effect on senescence induction and caspase-3 increased expression. We concluded that diabetes induces an increase in CYP24A1 expression, destabilizing vitamin D metabolism in the renal proximal tubules, leading to cellular instability and apoptosis, and thereby accelerating tubular injury progression during diabetic nephropathy. |
| 9600248 | High frequency of polymorphism but no mutations found in the GLUT1 glucose transporter gene in NIDDM and familial obesity by SSCP analysis.To evaluate whether a structural defect in the human glucose transporter gene GLUT1 could be involved in the aetiology of insulin resistance, a key factor of non-insulin-dependent diabetes mellitus (NIDDM) and obesity, we performed single-strand conformation polymorphism (SSCP) analysis in 40 subjects (20 NIDDM patients and 20 subjects with familial obesity). The GLUT1 gene, which is involved in basal glucose transport in most tissues, consists of ten exons and encodes a 492 amino acid protein. Population studies have shown a strong association between the X1 allele of an XbaI restriction fragment length polymorphism of the GLUT1 gene and NIDDM. We therefore performed SSCP analysis in NIDDM subjects known to carry at least one X1 allele. Variant SSCP patterns were detected in exons 2, 4, 5 and 9. Sequence analysis of the SSCP variants revealed the presence, in all exons examined, of silent mutations consisting of single-nucleotide substitutions with no amino acid changes. Both NIDDM and obese patients showed a high frequency of polymorphism in the sequence (50% and 35%, respectively). We conclude that the GLUT1 gene is unlikely to play a role in the aetiology of NIDDM and obesity. However, the strong association between the GLUT1 gene and NIDDM, together with the recent family studies showing linkage between chromosome 1p and NIDDM warrant further studies on this chromosomal region. |
| 16126724 | Dual specificity MAPK phosphatase 3 activates PEPCK gene transcription and increases gluconeogenesis in rat hepatoma cells.Insulin is a key hormone that controls glucose homeostasis. In liver, insulin suppresses gluconeogenesis by inhibiting the transcriptions of phosphoenolpyruvate carboxylase (PEPCK) and glucose-6-phosphatase (G6Pase) genes. In insulin resistance and type II diabetes there is an elevation of hepatic gluconeogenesis, which contributes to hyperglycemia. To search for novel genes that negatively regulate insulin signaling in controlling metabolic pathways, we screened a cDNA library derived from the white adipose tissue of ob/ob mice using a reporter system comprised of the PEPCK promoter placed upstream of the alkaline phosphatase gene. The mitogen-activated dual specificity protein kinase phosphatase 3 (MKP-3) was identified as a candidate gene that antagonized insulin suppression on PEPCK gene transcription from this screen. In this study, we showed that MKP-3 was expressed in insulin-responsive tissues and that its expression was markedly elevated in the livers of insulin-resistant obese mice. In addition, MKP-3 can activate PEPCK promoter in synergy with dexamethasone in hepatoma cells. Furthermore, ectopic expression of MKP-3 in hepatoma cells by adenoviral infection increased the expression of PEPCK and G6Pase genes and led to elevated glucose production. Taken together, our data strongly suggests that MKP-3 plays a role in regulating gluconeogenic gene expression and hepatic gluconeogenesis. Therefore, dysregulation of MKP-3 expression and/or function in liver may contribute to the pathogenesis of insulin resistance and type II diabetes. |
| 20883119 | Common polymorphisms in six genes of the methyl group metabolism pathway and obesity in European adolescents.OBJECTIVE: The goal of the present study was to assess the relationship between the genetic variability in six genes of methyl group (CH(3)) metabolism and the risk of obesity. METHODS: Single nucleotide polymorphisms (SNP) were selected among the methylene-tetrahydrofolate reductase (MTHFR), methionine synthase (MTR), methionine synthase reductase (MTRR), cystationine betha-syntase (CBS), transcobalamin-II (TCN2) and paraoxonase-1 (PON1) genes. The associations between SNPs and the risk of obesity were assessed in a case-control study of obese and normal-weight adolescents (age: 14.9+/-1.2 years), and the relationship between SNPs and body fat markers (i.e., body mass index [BMI], percentage body fat [BF%] and waist circumference [WC]) in a cross-sectional study of 1 155 European adolescents (age: 14.8+/-1.4 years). Genotyping was performed on an Illumina system and plasma folate level was determined by immunoassay. RESULTS: In the case-control study, there was no evidence for any association between SNPs of MTHFR, MTR, CBS, TCN2 and PON1 and obesity (all p values >/=0.08). In contrast, two SNPs of MTRR were associated with a higher (rs10520873, Odds Ratio: 1.68 [1.18-2.39]; p=0.004) or lower (rs1801394, 0.61 [0.42-0.87]; p=0.007) risk of obesity. In the cross-sectional sample, rs1801394 was associated with lower BMI (p=0.03) and lower waist circumference (p=0.02). However, after Bonferroni correction these associations were no longer significant. No other significant association or interaction between folate levels and SNPs were detected for anthropometric variables. CONCLUSION: Our findings do not support an association between MTHFR, MTR, CBS, TCN2 and PON1 SNPs and obesity in adolescence. Further investigations are necessary to confirm the possible association between the rs1801394 variant of MTRR and obesity. |
| 22450549 | TXNIP is highly regulated in bone biopsies from patients with Endogenous Cushing's Syndrome and related to bone turnover.Objective: Patients with Endogenous Cushing's Syndrome (CS), as long-time treated patients with exogenous glucocorticoids (GC), have severe systemic manifestations including secondary osteoporosis and low energy fractures. The aim of the study was to investigate the functional role of TXNIP in bone with focus on osteoblast differentiation and osteoblast mediated osteoclast activity and function in vitro.Design and Methods: Nine bone biopsies from CS before and after surgical treatment were screened for expressional candidate genes. Microarray analyses revealed that the gene encoding TXNIP ranked among the most up regulated genes. Subsequent in vitro and in vivo studies were performed.Results: We found that TXNIP gene in bone is regulated down in CS following surgical treatment. Furthermore, our in vivo data indicate novel associations between thioredoxin and TXNIP. Our in vitro studies showed that silencing TXNIP in osteoblasts was followed by increased differentiation and expression and secretion of osteocalcin as well as enhanced activity of alkaline phosphatase. Moreover, treating osteoclasts with silenced TXNIP osteoblast media, showed an increased osteoclast activity.Conclusions: TXNIP expression in bone is highly regulated during treatment of active CS, and by GC in bone cells in vitro. Our data indicate that TXNIP may mediate some of the detrimental effects of GC on osteoblast function as well as modulate osteoblast mediated osteoclastogenesis by regulating the OPG/RANKL ratio. |
| 19102712 | Glutathione S-transferase T1- and M1-null genotypes and coronary artery disease risk in patients with Type 2 diabetes mellitus.INTRODUCTION: Since long-term exposure to oxidative stress is strongly implicated in the pathogenesis of diabetic complications, polymorphic genes of detoxifying enzymes must be involved in the development of coronary artery disease (CAD). We assessed the potential glutathione S-transferase (GST) gene-gene (GSTM1(null)-GSTT1(null)) and gene-smoking interactions on the development of CAD in patients with Type 2 diabetes. MATERIALS & METHODS: In a case-only design, we enrolled 231 patients with Type 2 diabetes (147 male, 66.1 +/- 9.7 years) referred to our institute for coronary angiography investigation. CAD was diagnosed if there was over 50% obstruction of one or more major vessels. RESULTS: Coronary angiography revealed significant CAD in 184 patients (80%). Male gender (p < 0.001), smoking habits (p = 0.003) and GSTT1(null) genotype (p = 0.003) were significantly correlated with the increasing extent of the coronary atherosclerosis. Case-only analysis revealed that patients with both M(null)-T(null) genotypes had the highest risk for 3-vessel CAD compared with patients who express both GST genes (odds ratio: 3.1; 95% confidence interval: 1.0-10.3, p = 0.04). A nearly threefold interaction existed between cigarette smoking and M(null)-T(null) genotypes (odds ratio: 2.9, 95% confidence interval: 1.7-7.8, p = 0.03). A significant interaction between M(null)-T(null) genotypes and smoking was also observed on the increasing number of coronary vessels that were diseased (chi(2) = 14.0; p = 0.03). CONCLUSION: These data suggest that polymorphisms in GSTM1 and GSTT1 genes are risk factors for CAD in Type 2 diabetic patients, especially among smokers. These genetic markers may permit the targeting of preventive and early intervention on high-risk patients to reduce their cardiovascular risk. |
| 21270263 | Hyperglycemia activates caspase-1 and TXNIP-mediated IL-1beta transcription in human adipose tissue.OBJECTIVE: Obesity is characterized by elevated levels of proinflammatory cytokines, including interleukin (IL)-1beta, that contribute to the development of insulin resistance. In this study, we set out to investigate whether hyperglycemia drives IL-1beta production and caspase-1 activation in murine and human adipose tissue, thus inducing insulin resistance. RESEARCH DESIGN AND METHODS: ob/ob animals were used as a model to study obesity and hyperglycemia. Human adipose tissue fragments or adipocytes were cultured in medium containing normal or high glucose levels. Additionally, the role of thioredoxin interacting protein (TXNIP) in glucose-induced IL-1beta production was assessed. RESULTS: TXNIP and caspase-1 protein levels were more abundantly expressed in adipose tissue of hyperglycemic ob/ob animals as compared with wild-type mice. In human adipose tissue, high glucose resulted in a 10-fold upregulation of TXNIP gene expression levels (P < 0.01) and a 10% elevation of caspase-1 activity (P < 0.05), together with induction of IL-1beta transcription (twofold, P < 0.01) and a significant increase in IL-1beta secretion. TXNIP suppression in human adipocytes, either by a small interfering RNA approach or a peroxisome proliferator-activated receptor-gamma agonist, counteracted the effects of high glucose on bioactive IL-1 production (P < 0.01) mainly through a decrease in transcription levels paralleled by reduced intracellular pro-IL-1beta levels. CONCLUSIONS: High glucose activates caspase-1 in human and murine adipose tissue. Glucose-induced activation of TXNIP mediates IL-1beta mRNA expression levels and intracellular pro-IL-1beta accumulation in adipose tissue. The concerted actions lead to enhanced secretion of IL-1beta in adipose tissue that may contribute to the development of insulin resistance. |
| 11473059 | Susceptibility and negative epistatic loci contributing to type 2 diabetes and related phenotypes in a KK/Ta mouse model.The KK/Ta mouse strain serves as a suitable polygenic model for human type 2 diabetes. Using 93 microsatellite markers in 208 KK/Ta x (BALB/c x KK/Ta)F1 male backcross mice, we carried out a genome-wide linkage analysis of KK/Ta alleles contributing to type 2 diabetes and related phenotypes, such as obesity and dyslipidemia. We identified three major chromosomal intervals significantly contributing to impaired glucose metabolism: one quantitative trait locus for impaired glucose tolerance on chromosome 6 and two loci for fasting blood glucose levels on chromosomes 12 and 15. The latter two loci appeared to act in a complementary fashion. Two intervals showed significant linkages for serum triglyceride levels, one on chromosome 4 and the other on chromosome 8. The KK allele on chromosome 8 acts to promote serum triglyceride levels, whereas the KK allele on chromosome 4 acts to suppress this effect in a recessive fashion. In addition, it is suggested that the chromosome 4 locus also acts to downregulate body weight and that the chromosome 8 locus acts to upregulate serum insulin levels. Our data clearly showed that each disease phenotype of type 2 diabetes and related disorders in KK/Ta mice is under the control of separate genetic mechanisms. However, there appear to be common genes contributing to different disease phenotypes. There are potentially important candidate genes that may be relevant to the disease. |
| 16807249 | CCAAT/enhancer-binding protein alpha mediates induction of hepatic phosphoenolpyruvate carboxykinase by p38 mitogen-activated protein kinase.Excessive hepatic gluconeogenesis and glucose production are important contributors to hyperglycemia in both type 1 and type 2 diabetes. In diabetic humans and animal models, elevated levels of p38 mitogen-activated protein kinase (p38) are observed in several tissues. Our study shows that activity of p38 is significantly elevated in livers of db/db or streptozocin-induced type 1 diabetic mice. Using cultured hepatoma cells, we find that activation of p38 enhances expression of hepatic gluconeogenic gene phosphoenolpyruvate carboxykinase (PEPCK). Furthermore, our studies demonstrate that activation of p38 stimulates phosphorylation of CCAAT/enhancer-binding protein alpha (C/EBPalpha) at serine 21 and increases its transactivation activity in the context of PEPCK gene transcription. Our results indicate that C/EBPalpha mediates p38-stimulated PEPCK transcription in liver cells. |
| 18300949 | Association between glutathione S-transferase A1, M1 and T1 polymorphisms and hypertension.The importance of oxidative stress in hypertension has recently received increasing attention. The association between the incidence of hypertension and a super family of antioxidant enzymes, glutathione S-transferase (GST)A1, GSTM1 and GSTT1, polymorphisms was investigated in 468 Japanese participants in a health screening program. The frequency of the GSTA1\*B allele carriers was significantly higher in hypertensive patients than normotensive participants [adjusted odds ratio (OR): 1.8; 95% confidence interval (CI): 1.1-2.9]. The risk of hypertension was significantly increased in the GSTA1\*B allele carriers having also the GSTM1 null genotype or both the GSTM1 and GSTT1 null genotypes (adjusted OR: 2.4; 95% CI: 1.2-4.9; adjusted OR: 3.1; 95% CI: 1.0-9.5, respectively). This is the first report identifying the GSTA1\*B allele as a genetic risk factor for hypertension. The determination of the GST genotypes may help in identifying individuals at high-risk for hypertension. |
| 18701465 | Alpha2-macroglobulin is a mediator of retinal ganglion cell death in glaucoma.Glaucoma is defined as a chronic and progressive optic nerve neuropathy, characterized by apoptosis of retinal ganglion cells (RGC) that leads to irreversible blindness. Ocular hypertension is a major risk factor, but in glaucoma RGC death can persist after ocular hypertension is normalized. To understand the mechanism underlying chronic RGC death we identified and characterized a gene product, alpha2-macroglobulin (alpha2M), whose expression is up-regulated early in ocular hypertension and remains up-regulated long after ocular hypertension is normalized. In ocular hypertension retinal glia up-regulate alpha2M, which binds to low-density lipoprotein receptor-related protein-1 receptors in RGCs, and is neurotoxic in a paracrine fashion. Neutralization of alpha2M delayed RGC loss during ocular hypertension; whereas delivery of alpha2M to normal eyes caused progressive apoptosis of RGC mimicking glaucoma without ocular hypertension. This work adds to our understanding of the pathology and molecular mechanisms of glaucoma, and illustrates emerging paradigms for studying chronic neurodegeneration in glaucoma and perhaps other disorders. |
| 19822956 | XbaI GLUT1 gene polymorphism and the risk of type 2 diabetes with nephropathy.Altered expression of the facilitated glucose transporter GLUT1 affects pathways implicated in the pathogenesis of diabetic nephropathy. There is indication that variation of GLUT1 gene (SLC2A1) contributes to development of microangiopathy in diabetes mellitus type 2 (DM) patients. A genetic association study involving Caucasians was carried out to investigate the role of XbalphaI polymorphism in the GLUT1 gene in diabetic nephropathy (DN). Study population (n=240) consisted of 148 unrelated patients with DM (92 cases with diabetic nephropathy (DN)), and of 92 matched healthy control subjects. Diabetic nephropathy was defined as persistent albuminuria (>300 mg/24 h) and/or renal failure, in the absence of non-diabetes induced renal disease. The analysis showed that the risk of developing DM and DN in XbaI(-) carriers, when healthy individuals were considered as controls, was two-fold: odds ratio (OR) 2.08 [95% confidence interval (1.14-3.79)]. However, there was no evidence of association between XbaI(-) and DN when patients with DM and without DN were considered as controls: OR=1.12 (0.55-2.26). Thus, the GLUT1 XbaI(-) allele is associated with DM, and possibly with a more severe form of the disease that can lead to development of DN. |
| 20577053 | Metformin inhibits hepatic gluconeogenesis in mice independently of the LKB1/AMPK pathway via a decrease in hepatic energy state.Metformin is widely used to treat hyperglycemia in individuals with type 2 diabetes. Recently the LKB1/AMP-activated protein kinase (LKB1/AMPK) pathway was proposed to mediate the action of metformin on hepatic gluconeogenesis. However, the molecular mechanism by which this pathway operates had remained elusive. Surprisingly, here we have found that in mice lacking AMPK in the liver, blood glucose levels were comparable to those in wild-type mice, and the hypoglycemic effect of metformin was maintained. Hepatocytes lacking AMPK displayed normal glucose production and gluconeogenic gene expression compared with wild-type hepatocytes. In contrast, gluconeogenesis was upregulated in LKB1-deficient hepatocytes. Metformin decreased expression of the gene encoding the catalytic subunit of glucose-6-phosphatase (G6Pase), while cytosolic phosphoenolpyruvate carboxykinase (Pepck) gene expression was unaffected in wild-type, AMPK-deficient, and LKB1-deficient hepatocytes. Surprisingly, metformin-induced inhibition of glucose production was amplified in both AMPK- and LKB1-deficient compared with wild-type hepatocytes. This inhibition correlated in a dose-dependent manner with a reduction in intracellular ATP content, which is crucial for glucose production. Moreover, metformin-induced inhibition of glucose production was preserved under forced expression of gluconeogenic genes through PPARgamma coactivator 1alpha (PGC-1alpha) overexpression, indicating that metformin suppresses gluconeogenesis via a transcription-independent process. In conclusion, we demonstrate that metformin inhibits hepatic gluconeogenesis in an LKB1- and AMPK-independent manner via a decrease in hepatic energy state. |
| 22069254 | Persistent glucose transporter expression on pancreatic beta cells from longstanding type 1 diabetic individuals.BACKGROUND: Recent reports have established the notion that many patients with longstanding type 1 diabetes (T1D) possess a remnant population of insulin-producing beta cells. It remains questionable, however, whether these surviving cells can physiologically sense and respond to glucose stimuli. METHODS: Frozen pancreatic sections from non-diabetic donors (n=8), type 2 diabetic patients (n=4), islet autoantibody-positive non-diabetic patients (n=3), type 1 diabetic patients (n=10) and one case of gestational diabetes were obtained via the network for Pancreatic Organ Donors. All longstanding T1D samples were selected based on the detection of insulin-producing beta cells in the pancreas by immunohistochemistry. RNA was isolated from all sections followed by cDNA preparation and quantitative real-time polymerase chain reaction for insulin, glucose transporter 1 (GLUT1), GLUT2 and GLUT3. Finally, immunofluorescent staining was performed on consecutive sections for all four of these markers and a comparison was made between the expression of GLUT2 in humans versus NOD mice. RESULTS: In contrast to islets from the most widely used T1D model, the NOD mouse, human islets predominantly express GLUT1 and, to a much lesser extent, GLUT3 on their surface instead of GLUT2. Relative expression levels of these receptors do not significantly change in the context of the various (pre-)diabetic conditions studied. Moreover, in both species preservation of GLUT expression was observed even under conditions of substantial leucocyte infiltration or decades of T1D duration. CONCLUSIONS: These data suggest that despite being subjected to multiple years of physiological stress, the remaining beta-cell population in longstanding T1D patients retains a capacity to sense glucose via its GLUTs. |
| 22078753 | Effects of hyperinsulinemia on lipoprotein lipase, angiopoietin-like protein 4, and glycosylphosphatidylinositol-anchored high-density lipoprotein binding protein 1 in subjects with and without type 2 diabetes mellitus.Our aims were to compare the systemic effects of insulin on lipoprotein lipase (LPL) in tissues from subjects with different degrees of insulin sensitivity. The effects of insulin on LPL during a 4-hour hyperinsulinemic, euglycemic clamp were studied in skeletal muscle, adipose tissue, and postheparin plasma from young healthy subjects (YS), older subjects with type 2 diabetes mellitus (DS), and older control subjects (CS). In addition, we studied the effects of insulin on the expression of 2 recently recognized candidate genes for control of LPL activity: angiopoietin-like protein 4 (ANGPTL4) and glycosylphosphatidylinositol-anchored high-density lipoprotein binding protein 1. As an effect of insulin, LPL activity decreased by 20% to 25% in postheparin plasma and increased by 20% to 30% in adipose tissue in all groups. In YS, the levels of ANGPTL4 messenger RNA in adipose tissue decreased 3-fold during the clamp. In contrast, there was no significant change in DS or CS. Regression analysis showed that the ability of insulin to reduce the expression of ANGPTL4 was positively correlated with M-values and inversely correlated with factors linked to the metabolic syndrome. Expression of glycosylphosphatidylinositol-anchored high-density lipoprotein binding protein 1 tended to be higher in YS than in DS or CS, but the expression was not affected by insulin in any of the groups. Our data imply that the insulin-mediated regulation of LPL is not directly linked to the control of glucose turnover by insulin or to ANGPTL4 expression in adipose tissue or plasma. Interestingly, the response of ANGPTL4 expression in adipose tissue to insulin was severely blunted in both DS and CS. |
| 22129885 | Effects of glutamine supplementation on oxidative stress-related gene expression and antioxidant properties in rats with streptozotocin-induced type 2 diabetes.There are close links among hyperglycaemia, oxidative stress and diabetic complications. Glutamine (GLN) is an amino acid with immunomodulatory properties. The present study investigated the effect of dietary GLN on oxidative stress-relative gene expressions and tissue oxidative damage in diabetes. There were one normal control (NC) and two diabetic groups in the present study. Diabetes was induced by an intraperitoneal injection of nicotinamide followed by streptozotocin (STZ). Rats in the NC group were fed a regular chow diet. In the two diabetic groups, one group (diabetes mellitus, DM) was fed a common semi-purified diet while the other group received a diet in which part of the casein was replaced by GLN (DM-GLN). GLN provided 25 % of total amino acid N. The experimental groups were fed the respective diets for 8 weeks, and then the rats were killed for further analysis. The results showed that blood thioredoxin-interacting protein (Txnip) mRNA expression in the diabetic groups was higher than that in the NC group. Compared with the DM group, the DM-GLN group had lower glutamine fructose-6-phosphate transaminase 1, a receptor of advanced glycation end products, and Txnip gene expressions in blood mononuclear cells. The total antioxidant capacity was lower and antioxidant enzyme activities were altered by the diabetic condition. GLN supplementation increased antioxidant capacity and normalised antioxidant enzyme activities. Also, the renal nitrotyrosine level and Txnip mRNA expression were lower when GLN was administered. These results suggest that dietary GLN supplementation decreases oxidative stress-related gene expression, increases the antioxidant potential and may consequently attenuate renal oxidative damage in rats with STZ-induced diabetes. |
| 22955269 | Increased phosphoenolpyruvate carboxykinase gene expression and steatosis during hepatitis C virus subgenome replication: role of nonstructural component 5A and CCAAT/enhancer-binding protein beta.Chronic hepatitis C virus (HCV) infection greatly increases the risk for type 2 diabetes and nonalcoholic steatohepatitis; however, the pathogenic mechanisms remain incompletely understood. Here we report gluconeogenic enzyme phosphoenolpyruvate carboxykinase (PEPCK) transcription and associated transcription factors are dramatically up-regulated in Huh.8 cells, which stably express an HCV subgenome replicon. HCV increased activation of cAMP response element-binding protein (CREB), CCAAT/enhancer-binding protein (C/EBPbeta), forkhead box protein O1 (FOXO1), and peroxisome proliferator-activated receptor gamma coactivator 1alpha (PGC-1alpha) and involved activation of the cAMP response element in the PEPCK promoter. Infection with dominant-negative CREB or C/EBPbeta-shRNA significantly reduced or normalized PEPCK expression, with no change in PGC-1alpha or FOXO1 levels. Notably, expression of HCV nonstructural component NS5A in Huh7 or primary hepatocytes stimulated PEPCK gene expression and glucose output in HepG2 cells, whereas a deletion in NS5A reduced PEPCK expression and lowered cellular lipids but was without effect on insulin resistance, as demonstrated by the inability of insulin to stimulate mobilization of a pool of insulin-responsive vesicles to the plasma membrane. HCV-replicating cells demonstrated increases in cellular lipids with insulin resistance at the level of the insulin receptor, increased insulin receptor substrate 1 (Ser-312), and decreased Akt (Ser-473) activation in response to insulin. C/EBPbeta-RNAi normalized lipogenic genes sterol regulatory element-binding protein-1c, peroxisome proliferator-activated receptor gamma, and liver X receptor alpha but was unable to reduce accumulation of triglycerides in Huh.8 cells or reverse the increase in ApoB expression, suggesting a role for increased lipid retention in steatotic hepatocytes. Collectively, these data reveal an important role of NS5A, C/EBPbeta, and pCREB in promoting HCV-induced gluconeogenic gene expression and suggest that increased C/EBPbeta and NS5A may be essential components leading to increased gluconeogenesis associated with HCV infection. |
| 8636258 | Examination of the phosphoenolpyruvate carboxykinase gene promoter in patients with noninsulin-dependent diabetes mellitus.Expression of phosphoenolpyruvate carboxykinase (PEPCK), a rate-limiting enzyme in gluconeogenesis, is under dominant negative regulation by insulin. In this study, we sought to test the hypothesis that mutations in the PEPCK gene promoter may impair the ability of insulin to suppress hepatic glucose production, thereby contributing to both the insulin resistance and increased rate of gluconeogenesis characteristic of NIDDM. The proximal PEPCK promoter region in 117 patients with noninsulin-dependent diabetes mellitus and 20 obese Pima Indians was amplified by PCR and analyzed with single strand conformation polymorphism techniques. In addition, limited direct DNA sequencing was performed on the insulin response sequence and flanking regions. No DNA sequence polymorphisms were found in any patient. This result suggests that mutations in cis-acting PEPCK gene regulatory elements do not constitute a common cause of noninsulin-dependent diabetes mellitus. The significance of genetic variation in promoter regions to human disease is discussed. |
| 15448092 | Hepatic insulin resistance precedes the development of diabetes in a model of intrauterine growth retardation.Intrauterine growth retardation (IUGR) has been linked to the development of type 2 diabetes in adulthood. We developed an IUGR model in rats whereby at age 3-6 months the animals develop a diabetes that is associated with insulin resistance. Hyperinsulinemic-euglycemic clamp studies were performed at age 8 weeks, before the onset of obesity and diabetes. Basal hepatic glucose production (HGP) was significantly higher in IUGR than in control rats (14.6 +/- 0.4 vs. 12.3 +/- 0.3 mg. kg(-1). min(-1); P < 0.05). Insulin suppression of HGP was blunted in IUGR versus control rats (10.4 +/- 0.6 vs. 6.5 +/- 1.0 mg. kg(-1). min(-1); P < 0.01); however, rates of glucose uptake and glycogenolysis were similar between the two groups. Insulin-stimulated insulin receptor substrate 2 and Akt-2 phosphorylation were significantly blunted in IUGR rats. PEPCK and glucose-6-phosphatase mRNA levels were increased at least threefold in liver of IUGR compared with control rats. These studies suggest that an aberrant intrauterine milieu permanently impairs insulin signaling in the liver so that gluconeogenesis is augmented in the IUGR rat. These processes occur early in life, before the onset of hyperglycemia, and indicate that uteroplacental insufficiency causes a primary defect in gene expression and hepatic metabolism that leads to the eventual development of overt hyperglycemia. |
| 20730618 | Mitochondrial and nuclear gene mutations in the type 2 diabetes patients of Coimbatore population.Involvement of mitochondrial and nuclear gene mutations in the development of type 2 diabetes (T2D) has been established well in various populations around the world. Previously, we have found the mitochondrial A>G transition at nucleotide position 3243 and 8296 in the T2D patients of Coimbatore population. This study is aimed to screen for the presence of various mitochondrial and nuclear DNA mutations in the T2D patients of Coimbatore to identify most prevalent mutation. This helps in identifying the susceptible individuals based on their clinical phenotype in future. Blood samples were collected from 150 unrelated late-onset T2D patients and 100 age-matched unrelated control samples according to World Health Organization criteria. Genotyping for the selected genes was done by polymerase chain reaction-single strand confirmation polymorphism, direct sequencing, and polymerase chain reaction-restriction fragment length polymorphism. The mitochondrial T>C transition at 8356 and nuclear-encoded GLUT1 gene mutation were found in the selected T2D patients. The T8356C mutation was found in two patients (1.3%), and the clinical characteristics were found to be similar in both the patients whereas GLUT1 gene mutation was found in seven patients. Four out of seven patients showed homozygous (-) genotype and three patients showed heterozygous (+/-) genotype for the mutant allele XbaI. Among these three patients, one patient was found to have elevated level of urea and creatinine with the history of kidney dysfunction and chronic T2D. Our results suggest that the T8356C and GLUT1 gene mutations may have an important role in developing late-onset T2D in Coimbatore population. Particularly, individuals with GLUT1 gene may develop kidney dysfunction at their later age. |
| 23014993 | Study of the association between glutathione S-transferase (GSTM1, GSTT1, GSTP1) polymorphisms with type II diabetes mellitus in southern of Iran.Diabetes Mellitus is characterized by chronic hyperglycemia and associated with an increased production of reactive oxygen species (ROS). Oxidative stress is the result of accumulation of free radicals in tissues which specially affects beta cells in pancreas. Glutathione S-transferases (GSTs) are a family of antioxidant enzymes that include several classes of GSTs. These enzymes have important roles in decreasing of ROS species and act as a kind of antioxidant defense. To investigate the association between GSTs polymorphism with type 2 diabetes mellitus (T2DM), we investigated the frequency of GSTM1, T1 and P1 genotypes in patients with T2DM and controls. The genotypes of GSTT1, M1 and P1 were determined in 171 clinically documented T2DM patients and 169 normal cases (as controls) by multiplex polymerase chain reaction and PCR-RFLP. In diabetic patients, the frequency of GSTM1-null genotype was significantly (OR = 1.74; 95 % CI = 1.13-2.69, P = 0.016) higher than that in control. However, the frequency of GSTT1 (OR = 1.29; 95 % CI = 0.07-2.14, P = 0.367) and GSTP1 (OR = 0.83; 95 % CI = 0.53-1.30, P = 0.389) genotypes were not significantly different comparing both groups. Also, the frequency of both GSTT1-null and GSTM1-null genotypes in patients (19.88 %) was significantly higher compared to controls with the same genotypes (11.83 %, P = 0.022). Our results indicated that GSTM1 and GSTT1 genotypes might be involved in the pathogenesis of T2DM in south Iranian population. |
| 11436180 | Altered GLUT1 and GLUT3 gene expression and subcellular redistribution of GLUT4: protein in muscle from patients with acanthosis nigricans and severe insulin resistance.Multiple isoforms of glucose transporters are found in muscle, the tissue that normally accounts for 85% of insulin-stimulated glucose uptake. Glucose uptake into muscle cells in the fasting state is mediated primarily by GLUT1 and GLUT3 glucose transporters, whereas postprandial (insulin-stimulated) and exercise-related increments in muscle glucose uptake are mediated primarily by GLUT4. To determine if glucose transporters are abnormally expressed in muscle from insulin-resistant subjects, muscle samples were obtained from 10 normal subjects and 6 obese, nondiabetic subjects with severe insulin resistance and acanthosis nigricans. Both GLUT4 total protein and mRNA were normal in the insulin-resistant subjects. Muscle GLUT3 protein and mRNA were lower than controls by 62% and 71%, respectively. GLUT1 mRNA was twice normal, whereas GLUT1 protein content was not significantly increased. GLUT4 protein was markedly redistributed to the muscle plasma membrane in subjects with severe insulin resistance compared with normals (92% v 40% GLUT4 in plasma membrane-enriched fractions, P <.001), whereas the percentage of GLUT1 and GLUT3 protein found in the plasma membrane-enriched fractions was not different from controls. These data document differences in the expression of genes for GLUT1 and GLUT3 in muscle from normal and insulin-resistant subjects. Further, insulin resistance with fasting hyperinsulinemia was associated with a redistribution of GLUT4 to the muscle cell surface with no change in total GLUT4 protein. These data suggest that glucose transporter gene expression and their basal distribution in human muscle are related to insulin resistance and could be determinants of whole body insulin responsiveness. |
| 12359146 | Analysis of MGEA5 on 10q24.1-q24.3 encoding the beta-O-linked N-acetylglucosaminidase as a candidate gene for type 2 diabetes mellitus in Pima Indians.Several diseases including type 2 diabetes mellitus (T2DM) are associated with abnormal O-glycosylation of proteins. beta-O-linked N-acetylglucosaminidase (O-GlcNAcase) encoded by MGEA5 on 10g24.1-q24.3 removes N-acetylglucosamine (O-GlcNAc), and we investigated this locus in Pima Indians who have the world's highest prevalence of T2DM. We detected two variants but there was no association with parameters of insulin resistance or diabetes in approximately 1300 Pimas. We conclude that mutations in MGEA5 are unlikely to contribute to T2DM in this population. |
| 12716972 | Targeted deletion of histidine decarboxylase gene in mice increases bone formation and protects against ovariectomy-induced bone loss.Targeted disruption of the histidine decarboxylase gene (HDC(-/-)), the only histamine-synthesizing enzyme, led to a histamine-deficient mice characterized by undetectable tissue histamine levels, impaired gastric acid secretion, impaired passive cutaneous anaphylaxis, and decreased mast cell degranulation. We used this model to study the role of histamine in bone physiology. Compared with WT mice, HDC(-/-) mice receiving a histamine-free diet had increased bone mineral density, increased cortical bone thickness, higher rate of bone formation, and a marked decrease in osteoclasts. After ovariectomy, cortical and trabecular bone loss was reduced by 50% in HDC(-/-) mice compared with WT. Histamine deficiency protected the skeleton from osteoporosis directly, by inhibiting osteoclastogenesis, and indirectly, by increasing calcitriol synthesis. Quantitative RT-PCR showed elevated 25-hydroxyvitamin D-1alpha-hydroxylase and markedly decreased 25-hydroxyvitamin D-24-hydroxylase mRNA levels. Serum parameters confirming this indirect effect included elevated calcitriol, phosphorus, alkaline phosphatase, and receptor activator of NF-kappaB ligand concentrations, and suppressed parathyroid hormone concentrations in HDC(-/-) mice compared with WT mice. After ovariectomy, histamine-deficient mice were protected from bone loss by the combination of increased bone formation and reduced bone resorption. |
| 17095531 | Elevation of the post-translational modification of proteins by O-linked N-acetylglucosamine leads to deterioration of the glucose-stimulated insulin secretion in the pancreas of diabetic Goto-Kakizaki rats.Many nuclear and cytoplasmic proteins are O-glycosylated on serine or threonine residues with the monosaccharide beta-N-acetylglucosamine, which is then termed O-linked N-acetylglucosamine (O-GlcNAc). It has been shown that abnormal O-GlcNAc modification (O-GlcNAcylation) of proteins is one of the causes of insulin resistance and diabetic complications. In this study, in order to examine the relationship between O-GlcNAcylation of proteins and glucose-stimulated insulin secretion in noninsulin-dependent type (type 2) diabetes, we investigated the level of O-GlcNAcylation of proteins, especially that of PDX-1, and the expression of O-GlcNAc transferase in Goto-Kakizaki (GK) rats, which are an animal model of type-2 diabetes. By immunoblot and immunohistochemical analyses, the expression of O-GlcNAc transferase protein and O-GlcNAc-modified proteins in whole pancreas and islets of Langerhans of 15-week-old diabetic GK rats and nondiabetic Wistar rats was examined. The expression of O-GlcNAc transferase at the protein level and O-GlcNAc transferase activity were increased significantly in the diabetic pancreas and islets. The diabetic pancreas and islets also showed an increase in total cellular O-GlcNAc-modified proteins. O-GlcNAcylation of PDX-1 was also increased. In the diabetic GK rats, significant increases in the immunoreactivities of both O-GlcNAc and O-GlcNAc transferase were observed. PUGNAc, an inhibitor of O-GlcNAcase, induced an elevation of O-GlcNAc level and a decrease of glucose-stimulated insulin secretion in isolated islets. These results indicate that elevation of the O-GlcNAcylation of proteins leads to deterioration of insulin secretion in the pancreas of diabetic GK rats, further providing evidence for the role of O-GlcNAc in the insulin secretion. |
| 17898543 | Small artery remodeling and erythrocyte deformability in L-NAME-induced hypertension: role of transglutaminases.BACKGROUND: Hypertension is associated with inward remodeling of small arteries and decreased erythrocyte deformability, both impairing proper tissue perfusion. We hypothesized that these alterations depend on transglutaminases, cross-linking enzymes present in the vascular wall, monocytes/macrophages and erythrocytes. METHODS AND RESULTS: Wild-type (WT) mice and tissue-type transglutaminase (tTG) knockout (KO) mice received the nitric oxide inhibitor Nomega-nitro-L-arginine methyl ester hydrochloride (L-NAME) to induce hypertension. After 1 week, mesenteric arteries from hypertensive WT mice showed a smaller lumen diameter (-6.9 +/- 2.0%, p = 0.024) and a larger wall-to-lumen ratio (11.8 +/- 3.5%, p = 0.012) than controls, whereas inward remodeling was absent in hypertensive tTG KO mice. After 3 weeks, the wall-to-lumen ratio was increased in WT (20.8 +/- 4.8%, p = 0.005) but less so in tTG KO mice (11.7 +/- 4.6%, p = 0.026), and wall stress was normalized in WT but not in tTG KO mice. L-NAME did not influence expression of tTG or an alternative transglutaminase, coagulation factor XIII (FXIII). Suppression of FXIII by macrophage depletion was associated with increased tTG in the presence of L-NAME. L-NAME treatment decreased erythrocyte deformability in the WT mice (-15.3% at 30 dynes/cm(2), p = 0.014) but not in the tTG KO mice. CONCLUSION: Transglutaminases are involved in small artery inward remodeling and erythrocyte stiffening associated with nitric oxide inhibition-related hypertension. |
| 18382655 | Maternal TLR4 and NOD2 gene variants, pro-inflammatory phenotype and susceptibility to early-onset preeclampsia and HELLP syndrome.BACKGROUND: Altered maternal inflammatory responses play a role in the development of preeclampsia and the hemolysis, elevated liver enzymes and low platelets (HELLP) syndrome. We examined whether allelic variants of the innate immune receptors Toll-like receptor 4 (TLR4) and nucleotide-binding oligomerization domain 2 (NOD2), that impair the inflammatory response to endotoxin, are related to preeclampsia and HELLP syndrome. METHODS AND FINDINGS: We determined five common mutations in TLR4 (D299G and T399I) and NOD2 (R702W, G908R and L1007fs) in 340 primiparous women with a history of early-onset preeclampsia, of whom 177 women developed HELLP syndrome and in 113 women with a history of only uneventful pregnancies as controls. In addition, we assessed plasma levels of pro-inflammatory biomarkers C-reactive protein, interleukin-6, soluble intercellular adhesion molecule-1, fibrinogen and von Willebrand factor in a subset of 214 women included at least six months after delivery. After adjustment for maternal age and chronic hypertension, attenuating allelic variants of TLR4 were more common in women with a history of early-onset preeclampsia than in controls (OR 2.9 [95% CI 1.2-6.7]). Highest frequencies for TLR4 variants were observed in women who developed HELLP syndrome (adjusted OR 4.1 [95% CI 1.7-9.8]). In addition, high levels of interleukin-6 and fibrinogen were associated with a history of early-onset preeclampsia. Combined positivity for any of the TLR4 and NOD2 allelic variants and high levels of interleukin-6 was 6.9-fold more common in women with a history of early-onset preeclampsia (95% CI 2.1-23.2) compared to controls. CONCLUSIONS: We observed an association of common TLR4 and NOD2 gene variants, and pro-inflammatory phenotype with a history of early-onset preeclampsia and HELLP syndrome. These findings suggest involvement of the maternal innate immune system in severe hypertensive disorders of pregnancy. |
| 18728224 | Enhanced angiogenesis in obesity and in response to PPARgamma activators through adipocyte VEGF and ANGPTL4 production.PPARgamma activators such as rosiglitazone (RSG) stimulate adipocyte differentiation and increase subcutaneous adipose tissue mass. However, in addition to preadipocyte differentiation, adipose tissue expansion requires neovascularization to support increased adipocyte numbers. Paradoxically, endothelial cell growth and differentiation is potently inhibited by RSG in vitro, raising the question of how this drug can induce an increase in adipose tissue mass while inhibiting angiogenesis. We find that adipose tissue from mice treated with RSG have increased capillary density. To determine whether adipose tissue angiogenesis was stimulated by RSG, we developed a novel assay to study angiogenic sprout formation ex vivo. Angiogenic sprout formation from equally sized adipose tissue fragments, but not from aorta rings, was greatly increased by obesity and by TZD treatment in vivo. To define the mechanism involved in RSG-stimulated angiogenesis in adipose tissue, the expression of proangiogenic factors by adipocytes was examined. Expression of VEGFA and VEGFB, as well as of the angiopoietin-like factor-4 (ANGPTL4), was stimulated by in vivo treatment with RSG. To define the potential role of these factors, we analyzed their effects on endothelial cell growth and differentiation in vitro. We found that ANGPTL4 stimulates endothelial cell growth and tubule formation, albeit more weakly than VEGF. However, ANGPTL4 mitigates the growth inhibitory actions of RSG on endothelial cells in the presence or absence of VEGF. Thus, the interplay between VEGF and ANGPTL4 could lead to a net expansion of the adipose tissue capillary network, required for adipose tissue growth, in response to PPARgamma activators. |
| 19347605 | Frequency distribution of XbaIG > T and HaeIIIT > C GLUT1 polymorphisms among different Brazilian ethnic groups.GLUT is the major glucose transporter in mammalian cells. Single nucleotide polymorphisms (SNP) at GLUT1 promoter and regulatory regions have been associated to the risk of developing nephropathy in different type 1 and type 2 diabetic populations. It has been demonstrated that differences in allelic and genotypic frequencies of GLUT1 gene (SLC2A1) polymorphisms occur among different populations. Therefore, ethnic differences in distribution of GLUT1 gene polymorphisms may be an important factor in determining gene-disease association. In this study, we investigated the XbaIG > T and HaeIIIT > C polymorphisms in six different Brazilian populations: 102 individuals from Salvador population (Northern Brazil), 56 European descendants from Joinville (South Brazil), 85 Indians from Tiryio tribe (North Brazil) and 127 samples from Southern Brazil: 44 from European descendants, 42 from African descendants and 41 from Japanese descendants. Genotype frequencies from both sites did not differ significantly from those expected under the Hardy-Weinberg equilibrium. We verified that the allele frequencies of both polymorphisms were heterogeneous in these six Brazilian ethnic groups. |
| 19891555 | Genetic variation in CYP27B1 is associated with congestive heart failure in patients with hypertension.AIMS: We tested the hypothesis that genetic variation in vitamin D-dependent signaling is associated with congestive heart failure in human subjects with hypertension. MATERIALS & METHODS: Functional polymorphisms were selected from five candidate genes: CYP27B1, CYP24A1, VDR, REN and ACE. Using the Marshfield Clinic Personalized Medicine Research Project, we genotyped 205 subjects with hypertension and congestive heart failure, 206 subjects with hypertension alone and 206 controls (frequency matched by age and gender). RESULTS: In the context of hypertension, a SNP in CYP27B1 was associated with congestive heart failure (odds ratio: 2.14 for subjects homozygous for the C allele; 95% CI: 1.05-4.39). CONCLUSION: Genetic variation in vitamin D biosynthesis is associated with increased risk of heart failure. |
| 20739761 | Association of glutathione S-transferase (GSTM1, T1 and P1) gene polymorphisms with type 2 diabetes mellitus in north Indian population.BACKGROUND: Diabetes mellitus is associated with an increased production of reactive oxygen species (ROS) and a reduction in antioxidant defense. The oxidative stress becomes evident as a result of accumulation of ROS in conditions of inflammation and Type 2 diabetes mellitus (T2DM). The genes involved in redox balance, which determines the susceptibility to T2DM remain unclear. In humans, the glutathione S-transferase (GST) family comprises several classes of GST isozymes, the polymorphic variants of GSTM1, T1 and P1 genes result in decreased or loss of enzyme activity. AIMS: The present study evaluated the effect of genetic polymorphisms of the GST gene family on the risk of developing T2DM in the North Indian population. SETTINGS AND DESIGN: GSTM1, T1 and P1 polymorphisms were genotyped in 100 T2DM patients and 200 healthy controls from North India to analyze their association with T2DM susceptibility. MATERIALS AND METHODS: Analysis of GSTM1 and GSTT1 gene polymorphisms was performed by multiplex polymerase chain reaction (PCR) and GSTP1 by PCR-Restriction Fragment Length Polymorphism (RFLP). STATISTICAL ANALYSIS: Fisher's exact test and chi2 statistics using SPSS software (Version-15.0). RESULTS: We observed significant association of GSTM1 null (P=0.004, OR= 2.042, 95%CI= 1.254-3.325) and GSTP1 (I/V) (P=0.001, OR= 0.397, 95%CI=0.225-0.701) with T2DM and no significant association with GSTT1 (P=0.493). The combined analysis of the three genotypes GSTM1 null, T1 present and P1 (I/I) demonstrated an increase in T2DM risk (P= 0.005, OR= 2.431 95% CI=1.315-4.496). CONCLUSIONS: This is the first study showing the association of a combined effect of GSTM1, T1 and P1 genotypes in a representative cohort of Indian patients with T2DM. Since significant association was seen in GSTM1 null and GSTP1 (I/V) and multiple association in GSTM1 null, T1 present and P1 (I/I), these polymorphisms can be screened in the population to determine the diabetic risk. |
| 20797423 | C/EBPbeta is AMP kinase sensitive and up-regulates PEPCK in response to ER stress in hepatoma cells.Diabetes and obesity are associated with activation of endoplasmic reticulum (ER) stress; however a direct link between ER stress and increased hepatic gluconeogenesis remains unclear. Here we show that ER stress triggers a significant increase in expression of CCAAT/enhancer-binding protein (C/EBPbeta) and phosphorylated CREB together with reduced phospho-AMP-activated protein kinase (pAMPK) in hepatoma cells. ER stress contributed to transcriptional activation of the gluconeogenic phosphoenolpyruvate carboxykinase (PEPCK) promoter in Huh7 and HepG2 cells via cAMP binding motif (CRE site). Chromatin immunoprecipitation assays demonstrate that C/EBPbeta is recruited to the PEPCK promoter during ER stress and is reversed by pre-treatment with a JNK inhibitor that relieves ER stress. C/EBPbeta but not pCREB was suppressed by the AMPK-activator AICAR or constitutively active AMPK, while dominant negative AMPK increased C/EBPbeta expression. These data suggest that ER stress triggers suppression of AMPK while increasing C/EBPbeta and pCREB expression which activates PEPCK gene transcription. Understanding how ER stress suppresses AMPK activation and increases C/EBPbeta expression could lead to a potentially novel pathway for treatment of diabetes. |
| 22327174 | BMD Values and GSTM3 Gene Polymorphisms in Combination with GSTT1/GSTM1 Genes: A Genetic Association Study in Slovenian Elderly.Background: Much research suggests that oxidative stress is associated with osteoporosis development. Glutathione S-transferases mu3 (GSTM3) are an important group of detoxifying enzymes that eliminate oxidative stress-related products. Objectives: To examine the associations of functional GSTM3 gene polymorphisms (Val224Ile and insdelAGG), their haplotypes and, in combination with GSTT1-null and GSTM1-null polymorphisms, with bone mineral density (BMD) measured at femoral neck (\_fn), lumbar spine (\_ls) and total hip (\_th) and biochemical bone turnover markers in 593 Slovenian elderly women and 119 Slovenian elderly men. Methods: GSTM3, GSTT1-null and GSTM1-null gene polymorphisms using sizing denaturing high-performance liquid chromatography, triplex PCR method or real-time PCR; BMD\_fn, BMD\_ls, BMD\_th values using dual energy X-ray absorptiometry, and plasma osteocalcin, serum bone alkaline phosphatase and free soluble tumor necrosis factor (ligand) superfamily, member 11 (sRANKL) concentrations using a solid-phase, two-site chemiluminescent enzyme-labeled immunometric assay, radioimmunoassay or enzyme immunoassay were determined. Statistical analysis was performed using one-way and two-way ANCOVA with adjustment for potential confounders (age, height and weight). Results: The (borderline) significant differences in BMD\_th and BMD\_fn values between genotype subgroups of Val224Ile polymorphism of GSTM3 gene (p = 0.057 and 0.053, respectively) with the lowest BMD values among heterozygotes and between 224Ile-insAGG haplotype subgroups (p = 0.048 and 0.019, respectively) were found. Significant differences of BMD\_fn between the 224Ile-delAGG haplotype subgroups were observed (p = 0.012). Association of 224Val-insAGG with BMD\_fn was of borderline significance (p = 0.062). Conclusion: The results of our study demonstrate the genetic association between detoxifying enzyme GSTM3 and BMD variation, suggesting that the Val224Ile polymorphism and 224Ile-insAGG haplotype could be used for further evaluation of the impact of GSTs gene polymorphisms on osteoporosis, using larger cohorts in searching for osteoporosis risk markers. |
| 23350727 | Apoe, Mbl2, and Psp plasma protein levels correlate with diabetic phenotype in NZO mice--an optimized rapid workflow for SRM-based quantification.Male New Zealand Obese (NZO) mice progress through pathophysiological stages similar to humans developing obesity-associated type 2 diabetes (T2D). The current challenge is to establish quantitative proteomics from small plasma sample amounts. We established an analytical workflow that facilitates a reproducible depletion of high-abundance proteins, has high throughput applicability, and allows absolute quantification of proteins from mouse plasma samples by LC-SRM-MS. The ProteoMiner equalizing technology was adjusted to the small sample amount, and reproducibility of the identifications was monitored by spike proteins. Based on the label-free relative quantification of proteins in depleted plasma of a test set of NZO mice, assays for potential candidates were designed for the setup of a targeted selected reaction monitoring (SRM) approach and absolute quantification. We could demonstrate that apolipoprotein E (Apoe), mannose-binding lectin 2 (Mbl2), and parotid secretory protein (Psp) are present at significantly different quantities in depleted plasma of diabetic NZO mice compared to non-diabetic controls using AQUA peptides. Quantification was validated for Mbl2 using the ELISA technology on non-depleted plasma. We conclude that the depletion technique is applicable to restricted sample amounts and suitable for the identification of T2D signatures in plasma. |
| 7598707 | Analysis of the glucose transporter compliment of metabolically important tissues from the Milan hypertensive rat.Hypertension is frequently associated with peripheral insulin resistance. An expanding body of evidence has described aberrant expression of glucose transporters in the insulin resistance associated with diabetes mellitus. Therefore, we have investigated the relative levels of expression and subcellular distribution of four members of the facilitative glucose transporter family in metabolically important tissues from the hypertensive Milan rat. Skeletal muscle is the major site of peripheral glucose disposal; skeletal muscle membranes isolated from hypertensive animals exhibited a profoundly reduced level of GLUT4 protein compared to normotensive control animals This reduction was confined to the intracellular pool which exhibited a 50% lower level of GLUT4. In contrast, adipocytes, the other major site of peripheral glucose disposal, exhibited no change in the levels of expression of either GLUT1 or GLUT4 transporter isoforms. Hepatocytes from hypertensive animals exhibit similar levels of GLUT2 protein to the normotensive controls. Patterns of expression of GLUT1, GLUT3 and GLUT4 as determined by immunoblot analysis were profoundly altered in certain brain regions in the hypertensive state. Given the importance of the GLUT4 isoform in mediating the insulin-stimulated disposal of glucose into peripheral tissues, the observation that muscle exhibits profoundly decreased levels of this transporter has important implications for the insulin-resistance associated with hypertension in these animals. |
| 7665162 | Molecular mapping of SSRs for Pgm1 and C8b in the vicinity of the rat fatty locus.Recessive mutations at the rat fatty locus (fa, facp), which produce obesity, insulin resistance, and diabetes, provide useful experimental models for similar phenotypes in humans. The molecular pathogenesis of the metabolic phenotype in animals segregating for fa is unknown and difficult to study once the confounding metabolic effects of obesity are present. Although various experimental methods distinguish preobese from lean rats (phenotypic markers and molecular markers genetically linked to fatty), technical difficulties limit their utility. We report the identification of two (GT)n simple sequence repeats (SSRs) near the rat phosphoglucomutase gene (Pgm1) gene and two SSRs, (GA)n and (GT)n, near the rat complement component 8 beta gene (C8b). These SSRs map to an approximately 4-cM interval flanking the fatty locus on rat chromosome 5. Use of these molecular markers in combination offers an improved method for early assessment of gene dosage for fa and hence for studying the fundamental molecular physiology underlying the derangements of metabolism and behavior resulting from mutations in this gene. |
| 16717449 | Cloning, chromosome mapping and expression characteristics of porcine ANGPTL3 and -4.Angiopoietin-like protein 3 and -4 (ANGPTL3 and -4) are two members of angiopoietin-like proteins (ANGPTLs), which have the signature structure of the angiopoietin family but cannot bind to the TIE2 receptor. It has been reported that they both affect lipid metabolism by inhibiting the activity of lipoprotein lipase (LPL). Here we report the cDNA cloning, chromosome mapping and expression analysis of ANGPTL3 and -4 in pigs. Sequence analysis shows that ANGPTL3 contains an open reading frame of 1,389 bp, which encodes 462 amino acids, and ANGPTL4 contains a coding region of 1,239 bp, which encodes 412 amino acids. Porcine ANGPTL3 deduced amino acid sequence shares 83% and 73.7% identity with human and mouse, respectively, and ANGPTL4 shares 79.4% and 77.7% amino acid identity with human and mouse, respectively. Porcine ANGPTL3 and -4 were mapped to the 6q31-->q35 and 2q21-->q24 region, respectively, by radiation hybrid mapping. Tissue distribution analysis indicated that porcine ANGPTL3 mRNA was exclusively expressed in liver, and porcine ANGPTL4 was ubiquitously expressed with the highest abundance in white adipose tissue. Furthermore, the mRNA level of ANGPTL3 and -4 in liver and the mRNA level of ANGPTL4 in white adipose tissue were significantly higher in genetically obese pigs than in their lean counterparts. This is the first report of molecular cloning and characterization of ANGPTL3 and -4 in pigs, which will be helpful for a better understanding of the role of ANGPTLs in lipid metabolism. |
| 19479837 | NOD2-associated pediatric granulomatous arthritis, an expanding phenotype: study of an international registry and a national cohort in Spain.OBJECTIVE: To study the phenotype characteristics of the largest to date cohort of patients with pediatric granulomatous arthritis (PGA) and documented mutations in the NOD2 gene. METHODS: We analyzed merged data from 2 prospective cohorts of PGA patients, the International PGA Registry and a Spanish cohort. A systematic review of the medical records of interest was performed to identify phenotype characteristics. RESULTS: Forty-five patients with PGA (23 sporadic cases and 22 from familial pedigrees) and documented NOD2 mutations were identified and formed the basis of the study. Of these 45 patients, 18 had the R334W-encoding mutation, 18 had R334Q, 4 had E383K, 3 had R587C, 1 had C495Y, and 1 had W490L. The majority of patients manifested the typical triad of dermatitis, uveitis, and arthritis. In contrast, in 13 patients, the following "atypical" manifestations were noted: fever, sialadenitis, lymphadenopathy, erythema nodosum, leukocytoclastic vasculitis, transient neuropathy, granulomatous glomerular and interstitial nephritis, interstitial lung disease, arterial hypertension, hypertrophic cardiomyopathy, pericarditis, pulmonary embolism, hepatic granulomatous infiltration, splenic involvement, and chronic renal failure. In addition, 4 individuals who were asymptomatic carriers of a disease-causing mutation were documented. CONCLUSION: NOD2-associated PGA can be a multisystem disorder with significant visceral involvement. Treating physicians should be aware of the systemic nature of this condition, since some of these manifestations may entail long-term morbidity. |
| 22215653 | Plasma mannose-binding lectin is stimulated by PPARalpha in humans.The peroxisome proliferator activated receptor-alpha (PPARalpha) is a major transcriptional regulator of lipid metabolism in liver and represents the molecular target for hypolipidemic fibrate drugs. Effects of PPARalpha on lipid metabolism are partially mediated by circulating proteins such as FGF21 and ANGPTL4. The present study was undertaken to screen for and identify circulating proteins produced by human liver that are under the control of PPARalpha. Toward that aim, primary human hepatocytes were treated with the synthetic PPARalpha agonist Wy-14643 and whole genome expression data selected for secreted proteins. Expression of FGF21, ANGPTL4, and mannose-binding lectin (MBL), a soluble mediator of innate immunity and primary component of the lectin branch of the complement system, was markedly upregulated by Wy-14643 in primary human hepatocytes. Mice express two MBL isomers, Mbl1 and Mbl2. Mbl1 mRNA was weakly induced by Wy-14643 in primary mouse hepatocytes and remained unaltered by Wy-14643 in mouse liver. Mbl2 mRNA was unchanged by Wy-14643 in primary mouse hepatocytes and was strongly reduced by Wy-14643 in mouse liver. Remarkably, plasma Mbl1 levels were increased by chronic PPARalpha activation in lean and obese mice. Importantly, in two independent clinical trials, treatment with the PPARalpha agonist fenofibrate at 200 mg/day for 6 wk and 3 mo increased plasma MBL levels by 73 (P = 0.0016) and 86% (P = 0.017), respectively. It is concluded that hepatocyte gene expression and plasma levels of MBL are stimulated by PPARalpha and fenofibrate in humans, linking PPARalpha to regulation of innate immunity and complement activation in humans and suggesting a possible role of MBL in lipid metabolism. |
| 22528457 | Genetic Variation in Glutathione S-Transferase Genes and Risk of Nonfatal Cerebral Stroke in Patients Suffering from Essential Hypertension.Oxidative stress resulting from an increased amount of reactive oxygen species and an imbalance between oxidants and antioxidants has been implicated in pathogenesis of cerebral stroke. The purpose of this study was to investigate the relationship between common polymorphisms of glutathione S-transferase M1, T1, and P1 genes and risk of stroke in hypertensive individuals. A total of 667 unrelated Russian individuals with hypertension, including 306 hypertensives who suffered from cerebral stroke and 361 hypertensives who did not have cerebrovascular accidents, were recruited for the study. The deletion polymorphisms of GSTM1 and GSTT1 genes and polymorphism Ile105Val of the GSTP1 gene were genotyped by a multiplex polymerase chain reaction and restriction analyses, respectively. No differences in GSTM1 and GSTP1 genotype distributions between the cases and controls have been observed. The null GSTT1 genotype was found to be associated with increased risk of cerebral stroke after Bonferroni correction and adjusting for confounding variables such as gender, blood pressure, body mass index, and antihypertensive medication use (odds ratio 1.51 95 % CI 1.09-2.07, P = 0.01). The present study was the first to show the association of null genotype of the GSTT1 gene with increased risk of cerebral stroke. |
| 24175086 | Medullary nephrocalcinosis in an adult patient with idiopathic infantile hypercalcaemia and a novel CYP24A1 mutation.Idiopathic infantile hypercalcaemia (IIH) is an autosomal recessively inherited disease, presented in the first year of life with hypercalcaemia, precipitated by normal amounts of vitamin D supplementation. Recently loss-of-function mutations in the CYP24A1 gene, which encodes the vitamin D-metabolizing enzyme 24-hydroxylase, have been found in these patients. We describe a young man homozygous for a novel missense mutation (c.628T>C) of the CYP24A1 gene. He had suffered from severe hypercalcaemia in early childhood. At age 29 he presented with medullary nephrocalcinosis, chronic kidney disease (CKD) stage 2, microalbuminuria, mild hypertension and nephrogenic diabetes insipidus. He had mild hypercalcaemia and moderate hypercalciuria. As a novel finding, fibroblast growth factor 23 (FGF23) was elevated. |
| 12205028 | Role of transglutaminase 2 in glucose tolerance: knockout mice studies and a putative mutation in a MODY patient.Transglutaminase 2 (TGase 2) is a Ca+2-dependent enzyme that catalyzes both intracellular and extracellular cross-linking reactions by transamidation of specific glutamine residues. TGase 2 is known to be involved in the membrane-mediated events required for glucose-stimulated insulin release from the pancreatic beta cells. Here we show that targeted disruption of TGase 2 impairs glucose-stimulated insulin secretion. TGase 2-/- mice show glucose intolerance after intraperitoneal glucose loading. TGase 2-/- mice manifest a tendency to develop hypoglycemia after administration of exogenous insulin as a consequence of enhanced insulin receptor substrate 2 (IRS-2) phosphorylation. We suggest that the increased peripheral sensitivity to insulin partially compensates for the defective secretion in this animal model. TGase 2-/- mouse phenotype resembles that of the maturity-onset diabetes of young (MODY) patients. In the course of screening for human TGase 2 gene in Italian subjects with the clinical features of MODY, we detected a missense mutation (N333S) in the active site of the enzyme. Collectively, these results identify TGase 2 as a potential candidate gene in type 2 diabetes. |
| 12663463 | Elevation in Tanis expression alters glucose metabolism and insulin sensitivity in H4IIE cells.Increased hepatic glucose output and decreased glucose utilization are implicated in the development of type 2 diabetes. We previously reported that the expression of a novel gene, Tanis, was upregulated in the liver during fasting in the obese/diabetic animal model Psammomys obesus. Here, we have further studied the protein and its function. Cell fractionation indicated that Tanis was localized in the plasma membrane and microsomes but not in the nucleus, mitochondria, or soluble protein fraction. Consistent with previous gene expression data, hepatic Tanis protein levels increased more significantly in diabetic P. obesus than in nondiabetic controls after fasting. We used a recombinant adenovirus to increase Tanis expression in hepatoma H4IIE cells and investigated its role in metabolism. Tanis overexpression reduced glucose uptake, basal and insulin-stimulated glycogen synthesis, and glycogen content and attenuated the suppression of PEPCK gene expression by insulin, but it did not affect insulin-stimulated insulin receptor phosphorylation or triglyceride synthesis. These results suggest that Tanis may be involved in the regulation of glucose metabolism, and increased expression of Tanis could contribute to insulin resistance in the liver. |
| 15492856 | Glutathione S-transferase T1 deletion is a risk factor for developing end-stage renal disease in diabetic patients.Reactive oxygen metabolites may contribute to the development of end-stage renal disease (ESRD) in diabetic and hypertensive patients. In this study, we used multiplex polymerase chain reaction (PCR) to analyze polymorphisms of two endogenous antioxidant genes, glutathione S-transferase M1 (GSTM1) and glutathione S-transferase T1 (GSTT1), and to determine their role in the development of ESRD in diabetic and hypertensive patients. Our results showed that homozygous deletion of the GSTT1 gene is a risk factor for developing ESRD in diabetic patients (p=0.004, OR=2.18, 95% confidence interval (CI) =1.29-3.70), but not in hypertensive patients. No association between homozygous deletion of GSTM1 and the development of ESRD was found in either diabetic patients or hypertensive patients. These results indicate that genetic variations in enzymes involved in free radical metabolism are associated with the development of ESRD in diabetes mellitus (DM) patients, and may permit the targeting of preventive and early intervention strategies to high-risk individuals. |
| 16306366 | The lack of beta-adrenoceptors results in enhanced insulin sensitivity in mice exhibiting increased adiposity and glucose intolerance.We and others have previously shown that triple knockout mice lacking the beta1/beta2/beta3-adrenoceptors (beta-less mice) developed a progressive obesity at adulthood. Here, we studied the glucose homeostasis in beta-less mice before the onset of obesity. We show that beta-less mice have increased fat mass and are glucose intolerant. In addition, we observed that beta-less mice have impaired glucose-induced insulin secretion and exhibit an increase in liver PEPCK gene expression in the fed state, suggesting that they have increased gluconeogenesis. Although these characteristics are usually associated with insulin resistance, beta-less mice exhibit enhanced insulin sensitivity during insulin tolerance tests. This is keeping with the results obtained during euglycemic-hyperinsulinemic clamps showing that beta-less mice display increased insulin responsiveness with normal suppression of hepatic glucose production. Altogether, our results suggest that an intact beta-adrenergic system is required to regulate overall glucose homeostasis and, in particular, insulin-mediated glucose uptake, most likely at the level of muscles and adipose tissue. |
| 19139380 | Impaired vasodilator activity in deoxycorticosterone acetate-salt hypertension is associated with increased protein O-GlcNAcylation.Hyperglycemia, which increases O-linked beta-N-acetylglucosamine (O-GlcNAc) proteins, leads to changes in vascular reactivity. Because vascular dysfunction is a key feature of arterial hypertension, we hypothesized that vessels from deoxycorticosterone acetate and salt (DOCA-salt) rats exhibit increased O-GlcNAc proteins, which is associated with increased reactivity to constrictor stimuli. Aortas from DOCA rats exhibited increased contraction to phenylephrine (E(max) [mN]=17.6+/-4 versus 10.7+/-2 control; n=6) and decreased relaxation to acetylcholine (47.6+/-6% versus 73.2+/-10% control; n=8) versus arteries from uninephrectomized rats. O-GlcNAc protein content was increased in aortas from DOCA rats (arbitrary units=3.8+/-0.3 versus 2.3+/-0.3 control; n=5). PugNAc (O-GlcNAcase inhibitor; 100 micromol/L; 24 hours) increased vascular O-GlcNAc proteins, augmented phenylephrine vascular reactivity (18.2+/-2 versus 10.7+/-3 vehicle; n=6), and decreased acetylcholine dilation in uninephrectomized (41.4+/-6 versus 73.2+/-3 vehicle; n=6) but not in DOCA rats (phenylephrine, 16.5+/-3 versus 18.6+/-3 vehicle, n=6; acetylcholine, 44.7+/-8 versus 47.6+/-7 vehicle, n=6). PugNAc did not change total vascular endothelial nitric oxide synthase levels, but reduced endothelial nitric oxide synthase(Ser-1177) and Akt(Ser-473) phosphorylation (P<0.05). Aortas from DOCA rats also exhibited decreased levels of endothelial nitric oxide synthase(Ser-1177) and Akt(Ser-473) (P<0.05) but no changes in total endothelial nitric oxide synthase or Akt. Vascular O-GlcNAc-modified endothelial nitric oxide synthase was increased in DOCA rats. Blood glucose was similar in DOCA and uninephrectomized rats. Expression of O-GlcNAc transferase, glutamine:fructose-6-phosphate amidotransferase, and O-GlcNAcase, enzymes that directly modulate O-GlcNAcylation, was decreased in arteries from DOCA rats (P<0.05). This is the first study showing that O-GlcNAcylation modulates vascular reactivity in normoglycemic conditions and that vascular O-GlcNAc proteins are increased in DOCA-salt hypertension. Modulation of increased vascular O-GlcNAcylation may represent a novel therapeutic approach in mineralocorticoid hypertension. |
| 21682752 | Synergistic effect of alcohol consumption and body mass on serum concentrations of cytokeratin-18.BACKGROUND: Cytokeratin-18 is an essential component of the cytoskeleton of epithelial cells (including hepatocytes). Serum concentrations of cytokeratin-18 (tissue polypeptide-specific antigen [TPS]) are used as a marker of epithelial neoplasms. Here, we investigated the potential interaction between alcohol and obesity in relation to serum TPS concentrations. METHODS: Alcohol consumption, body mass index, and components of metabolic syndrome were measured in a random sample (n = 420) of the adult population (aged 18 to 92 years, 45% men) from a single municipality. Regular alcohol intake of >20 g/d (women) or >30 g/d (men) was considered risky drinking. Serum TPS was measured with a commercial immunoassay. RESULTS: Risky drinking was associated with increased serum concentrations of TPS, which was particularly evident among obese individuals. Among individuals without risky drinking, TPS concentrations were similar for all levels of body mass. Conversely, among risky drinkers, serum TPS concentrations increased in parallel with body mass (p = 0.002). The odds ratio of a high (>100 U/l) TPS concentration for the combination of risky drinking and obesity was greater than the additive effect of the 2 separate factors, after adjusting for age and sex. A similar interaction was observed between risky drinking and abdominal adiposity, a major component of the metabolic syndrome. Serum TPS concentrations were correlated with markers of liver damage. Serum TPS was not superior to standard markers (gamma-glutamyl transferase and red blood cell mean volume) for the detection of risky drinking. CONCLUSIONS: There is a synergism between risky alcohol consumption and common metabolic disorders (particularly obesity) in relation to serum concentrations of cytokeratin-18 (TPS), which probably reflect liver disease. |
| 22616691 | Functional annotation of the human fat cell secretome.CONTEXT: Recent secretome analyses suggest that human fat cells secrete hundreds of proteins (adipokines). OBJECTIVE: We made an overall analysis of their potential functional importance. MATERIALS AND METHODS: A secretome of 347 adipokines was evaluated by in silico analysis of their expression during adipocyte differentiation, regulation by obesity and adipose region. The gene expression in human adipose tissue was investigated in microarray studies using samples from different adipose depots from lean or obese patients. RESULTS: 60% of the adipokines were regulated by obesity and 50% between visceral and subcutaneous adipose region. Eight adipokines, all novel, scored particularly high in the in silico analysis. Among those, four were both regulated by obesity and adipose region, namely WNT1-inducible-signaling pathway protein 2, transmembrane glycoprotein NMB, inter-alpha-trypsin inhibitor heavy chain H5, and complement C4-A. Furthermore, many adipokines were extracellular matrix proteins. CONCLUSION: Several novel adipokines have potential important functional features warranting in depth analysis. |
| 22649517 | Polymorphisms related to the serum 25-hydroxyvitamin D level and risk of myocardial infarction, diabetes, cancer and mortality. The Tromso Study.OBJECTIVE: Low serum 25(OH)D levels are associated with cardiovascular risk factors, and also predict future myocardial infarction (MI), type 2 diabetes (T2DM), cancer and all-cause mortality. Recently several single nucleotide polymorphisms (SNPs) associated with serum 25-hydroxyvitamin D (25(OH)D) level have been identified. If these relations are causal one would expect a similar association between these SNPs and health. METHODS: DNA was prepared from subjects who participated in the fourth survey of the Tromso Study in 1994-1995 and who were registered with the endpoints MI, T2DM, cancer or death as well as a randomly selected control group. The endpoint registers were complete up to 2007-2010. Genotyping was performed for 17 SNPs related to the serum 25(OH)D level. RESULTS: A total of 9528 subjects were selected for genetic analyses which were successfully performed for at least one SNP in 9471 subjects. Among these, 2025 were registered with MI, 1092 with T2DM, 2924 with cancer and 3828 had died. The mean differences in serum 25(OH)D levels between SNP genotypes with the lowest and highest serum 25(OH)D levels varied from 0.1 to 7.8 nmol/L. A genotype score based on weighted risk alleles regarding low serum 25(OH)D levels was established. There was no consistent association between the genotype score or individuals SNPs and MI, T2DM, cancer, mortality or risk factors for disease. However, for rs6013897 genotypes (located at the 24-hydroxylase gene (CYP24A1)) there was a significant association with breast cancer (P<0.05). CONCLUSION: Our results do not support nor exclude a causal relationship between serum 25(OH)D levels and MI, T2DM, cancer or mortality, and our observation on breast cancer needs confirmation. Further genetic studies are warranted, particularly in populations with vitamin D deficiency. TRIAL REGISTRATION: ClinicalTrials.gov NCT01395303. |
| 11477481 | Glutathione S-transferase genotypes in systemic sclerosis and their association with clinical manifestations in early disease.The glutathione S-transferases (GSTs) are a family of enzymes involved in limiting oxidative damage to tissues. Null alleles for one or more of the GST enzymes, especially GSTM1, reportedly occur more frequently in patients with Sjogren's syndrome and systemic lupus erythematosus who possess certain autoantibodies. Because systemic sclerosis (SSc) is a disease in which oxidative damage has been hypothesized to contribute both to immune dysfunction and tissue damage, we sought to determine if patients from a multi-ethnic cohort of SSc patients with early disease (< or =5 years) were more likely than ethnically-matched normal controls to have null alleles for GSTM1 (M1) and/or GSTT1 (T1), and if the null allele status correlated with any major disease features. The data show that while M1 and T1 null genotypes were not significantly increased in SSc compared to ethnically matched controls, their frequencies (especially T1 nulls) were significantly higher among SSc patients with hypertension and pulmonary involvement. This suggests that GST genotype may be a genetic factor that contributes to clinical disease expression in SSc. |
| 12397394 | Regenerating human muscle fibres express GLUT3 protein.The presence of the GLUT3 glucose transporter protein in human muscle cells is a matter of debate. The present study was designed to establish whether GLUT3 is expressed in mature human skeletal muscle fibres and, if so, whether its expression changes under different conditions, such as metabolic stress (obesity, obese non-insulin-dependent diabetes mellitus), hypertrophy (training), de- and reinnervation (amyotrophic lateral sclerosis) or regeneration (polymyositis). We used an immunohistochemical approach to detect and localise GLUT3. GLUT3 immunoreactivity was not detectable in adult skeletal muscle fibres, nor did metabolic stress, training or de- and re-innervation induce GLUT3 expression, while a few GLUT3 expressing fibres were seen in some cases of polymyositis. In contrast, GLUT4 was expressed in all investigated muscle fibres. GLUT3 immunoreactivity was found in perineural and endoneural cells, indicating that GLUT3 is important for glucose transport into nerves through the perineurium. Taken together, these data suggest that GLUT3 expression is restricted to regenerating muscle fibres and nerves in adult human muscle. Although the significance of GLUT3 in adult human muscle fibres appears limited, GLUT3 may be of importance for the glucose supply in fetal muscle fibres and regenerating adult muscle fibres. |
| 14665706 | A polymorphism in the CYP17 gene and intrauterine fetal growth restriction.Intrauterine fetal growth restriction is a multifactorial disorder, and its aetiology includes both environmental and genetic components. We aimed to investigate whether maternal genetic polymorphisms of metabolic enzymes affects fetal growth and pregnancy duration. Genomic DNA was obtained from 134 women who experienced singleton deliveries beyond 24 weeks of gestation. Maternal age, birth weight, gestational age at birth and frequencies of fetal growth restriction, prematurity and pregnancy-induced hypertension were compared among genotypic subgroups of cytochrome p450 (CYP) and glutathione S-transferase (GST) genes. The polymorphisms of CYP1A1 (MspI), CYP17 (MspAI) and GSTP1 (BsmAI) genotypes, and the presence or absence of GSTM1 and GSTT1 genes were analysed by PCR-based methods. The frequency of fetal growth restriction (<10th percentile/<-1.5 SD; 22.7%/11.4%) in 44 women who were homozygous for the A1 allele (A1A1) of CYP17 was significantly higher than that (7.8%/2.2%) in 90 women who carried the A2 allele (A1A2/A2A2) of CYP17 (P < 0.05), with an odds ratio =3.41 (95% confidence interval = 1.18-9.84). The gestational age at birth (mean +/- SD, 37.5 +/- 3.1 weeks) in 67 women with GSTM1 null genotype was significantly lower than that (38.5 +/- 2.4 weeks) in 67 women who carried GSTM1 (P < 0.05). The polymorphism of CYP17 that encodes the cytochrome p450c17alpha enzyme might be associated with the pathophysiology underlying fetal growth restriction. |
| 15081545 | High leptin level is accompanied with decreased long leptin receptor transcript in histamine deficient transgenic mice.Leptin is a multifunctional cytokine and hormone that primarily acts in the hypothalamus and plays a key role in regulation of food intake and energy expenditure. Leptin acts through its receptor (OBR), the product of db gene that activates the Jak/STAT pathway predominantly. To exert its functions, leptin interacts with histamine as well. Histamine is a downstream effector of leptin as its release, metabolism is enhanced by leptin and hypothalamic histamine reduces food intake. In a bi-directional regulatory loop histamine also influences leptin concentration by inhibiting its expression. In this study we demonstrate that histamine deficiency elevates serum leptin level and decreases full-length leptin receptor isoform with a slight increase of the short one and results in mild late onset obesity. These observation can help to elucidate further the bi-lateral interaction of leptin and histamine, and therefore provide useful data to understand the pathomechanism of obesity. |
| 15735652 | Hypothalamic sensing of circulating fatty acids is required for glucose homeostasis.Increased glucose production is a hallmark of type 2 diabetes and alterations in lipid metabolism have a causative role in its pathophysiology. Here we postulate that physiological increments in plasma fatty acids can be sensed within the hypothalamus and that this sensing is required to balance their direct stimulatory action on hepatic gluconeogenesis. In the presence of physiologically-relevant increases in the levels of plasma fatty acids, negating their central action on hepatic glucose fluxes through (i) inhibition of the hypothalamic esterification of fatty acids, (ii) genetic deletion (Sur1-deficient mice) of hypothalamic K(ATP) channels or pharmacological blockade (K(ATP) blocker) of their activation by fatty acids, or (iii) surgical resection of the hepatic branch of the vagus nerve led to a marked increase in liver glucose production. These findings indicate that a physiological elevation in circulating lipids can be sensed within the hypothalamus and that a defect in hypothalamic lipid sensing disrupts glucose homeostasis. |
| 18445751 | Cross-talk between GlcNAcylation and phosphorylation: roles in insulin resistance and glucose toxicity.O-linked beta-N-acetylglucosamine (O-GlcNAc) is a dynamic posttranslational modification that, analogous to phosphorylation, cycles on and off serine and/or threonine hydroxyl groups. Cycling of O-GlcNAc is regulated by the concerted actions of O-GlcNAc transferase and O-GlcNAcase. GlcNAcylation is a nutrient/stress-sensitive modification that regulates proteins involved in a wide array of biological processes, including transcription, signaling, and metabolism. GlcNAcylation is involved in the etiology of glucose toxicity and chronic hyperglycemia-induced insulin resistance, a major hallmark of type 2 diabetes. Several reports demonstrate a strong positive correlation between GlcNAcylation and the development of insulin resistance. However, recent studies suggest that inhibiting GlcNAcylation does not prevent hyperglycemia-induced insulin resistance, suggesting that other mechanisms must also be involved. To date, proteomic analyses have identified more than 600 GlcNAcylated proteins in diverse functional classes. However, O-GlcNAc sites have been mapped on only a small percentage (<15%) of these proteins, most of which were isolated from brain or spinal cord tissue and not from other metabolically relevant tissues. Mapping the sites of GlcNAcylation is not only necessary to elucidate the complex cross-talk between GlcNAcylation and phosphorylation but is also key to the design of site-specific mutational studies and necessary for the generation of site-specific antibodies, both of which will help further decipher O-GlcNAc's functional roles. Recent technical advances in O-GlcNAc site-mapping methods should now finally allow for a much-needed increase in site-specific analyses to address the functional significance of O-GlcNAc in insulin resistance and glucose toxicity as well as other major biological processes. |
| 20194081 | Traffic-related air pollution and QT interval: modification by diabetes, obesity, and oxidative stress gene polymorphisms in the normative aging study.BACKGROUND: Acute exposure to ambient air pollution has been associated with acute changes in cardiac outcomes, often within hours of exposure. OBJECTIVES: We examined the effects of air pollutants on heart-rate-corrected QT interval (QTc), an electrocardiographic marker of ventricular repolarization, and whether these associations were modified by participant characteristics and genetic polymorphisms related to oxidative stress. METHODS: We studied repeated measurements of QTc on 580 men from the Veterans Affairs Normative Aging Study (NAS) using mixed-effects models with random intercepts. We fitted a quadratic constrained distributed lag model to estimate the cumulative effect on QTc of ambient air pollutants including fine particulate matter <or= 2.5 microm in aerodynamic diameter (PM2.5), ozone (O3), black carbon (BC), nitrogen dioxide (NO2), carbon monoxide (CO), and sulfur dioxide (SO2) concentrations during the 10 hr before the visit. We genotyped polymorphisms related to oxidative stress and analyzed pollution-susceptibility score interactions using the genetic susceptibility score (GSS) method. RESULTS: Ambient traffic pollutant concentrations were related to longer QTc. An interquartile range (IQR) change in BC cumulative during the 10 hr before the visit was associated with increased QTc [1.89 msec change; 95% confidence interval (CI), -0.16 to 3.93]. We found a similar association with QTc for an IQR change in 1-hr BC that occurred 4 hr before the visit (2.54 msec change; 95% CI, 0.28-4.80). We found increased QTc for IQR changes in NO2 and CO, but the change was statistically insignificant. In contrast, we found no association between QTc and PM2.5, SO2, and O3. The association between QTc and BC was stronger among participants who were obese, who had diabetes, who were nonsmokers, or who had higher GSSs. CONCLUSIONS: Traffic-related pollutants may increase QTc among persons with diabetes, persons who are obese, and nonsmoking elderly individuals; the number of genetic variants related to oxidative stress increases this effect. |
| 21084378 | The Drosophila NR4A nuclear receptor DHR38 regulates carbohydrate metabolism and glycogen storage.Animals balance nutrient storage and mobilization to maintain metabolic homeostasis, a process that is disrupted in metabolic diseases like obesity and diabetes. Here, we show that DHR38, the single fly ortholog of the mammalian nuclear receptor 4A family of nuclear receptors, regulates glycogen storage during the larval stages of Drosophila melanogaster. DHR38 is expressed and active in the gut and body wall of larvae, and its expression levels change in response to nutritional status. DHR38 null mutants have normal levels of glucose, trehalose (the major circulating form of sugar), and triacylglycerol but display reduced levels of glycogen in the body wall muscles, which constitute the primary storage site for carbohydrates. Microarray analysis reveals that many metabolic genes are mis-regulated in DHR38 mutants. These include phosphoglucomutase, which is required for glycogen synthesis, and the two genes that encode the digestive enzyme amylase, accounting for the reduced amylase enzyme activity seen in DHR38 mutant larvae. These studies demonstrate that a critical role of nuclear receptor 4A receptors in carbohydrate metabolism has been conserved through evolution and that nutritional regulation of DHR38 expression maintains the proper uptake and storage of glycogen during the growing larval stage of development. |
| 9124334 | Glucose utilization and glucose transporter proteins GLUT-1 and GLUT-3 in brains of diabetic (db/db) mice.This study describes the effects of diabetes on brain growth, cerebral glucose utilization (CGU), and the glucose transporter proteins GLUT-1 and GLUT-3 in the genetically diabetic db/db mouse. Mice were studied at 5 and 10 wk of age and compared with age-matched nondiabetic littermates. At 5 wk, db/db mice were not yet hyperglycemic, but their body weights were 27.5% greater than those of their nondiabetic littermates. By 10 wk, db/db mice were both hyperglycemic (blood glucose values of 39.3 +/- 4.3 vs. 12.1 +/- 2.1 mmol/l for db/db and control, respectively) and obese, with a twofold increase in body weight. Significant reductions in brain weight were observed at 5 wk (15% decrease in brain wet wt), and no further brain growth was observed, such that by 10 wk, brains of db/db mice were 25% smaller than those of control mice; brain wet weight-to-dry weight ratios were slightly reduced. Global rates of CGU, as determined with 2-[14C]deoxyglucose, were significantly reduced in the 10-wk diabetic mice. Levels of brain glucose and brain-to-blood glucose ratios were increased in 5- and 10-wk db/db mice, reflecting adequate glucose delivery to the brain. Blood-brain barrier GLUT-1 levels were unchanged, and mRNA levels were regionally increased. The expression of the neuronal glucose transporter GLUT-3 was not reduced to a significant extent in brains of db/db mice. The results of this study indicate that the db/db mouse has markedly decelerated brain growth accompanied by global reductions in glucose metabolism that are not due to reductions in glucose transport capacity. |
| 10866690 | Peroxisome proliferator-activated receptor gamma target gene encoding a novel angiopoietin-related protein associated with adipose differentiation.The nuclear receptor peroxisome proliferator-activated receptor gamma regulates adipose differentiation and systemic insulin signaling via ligand-dependent transcriptional activation of target genes. However, the identities of the biologically relevant target genes are largely unknown. Here we describe the isolation and characterization of a novel target gene induced by PPARgamma ligands, termed PGAR (for PPARgamma angiopoietin related), which encodes a novel member of the angiopoietin family of secreted proteins. The transcriptional induction of PGAR follows a rapid time course typical of immediate-early genes and occurs in the absence of protein synthesis. The expression of PGAR is predominantly localized to adipose tissues and placenta and is consistently elevated in genetic models of obesity. Hormone-dependent adipocyte differentiation coincides with a dramatic early induction of the PGAR transcript. Alterations in nutrition and leptin administration are found to modulate the PGAR expression in vivo. Taken together, these data suggest a possible role for PGAR in the regulation of systemic lipid metabolism or glucose homeostasis. |
| 18254710 | An association study of sodium-lithium countertransport activity with glutathione S transferase (GST) T1 and GST M1 null polymorphisms in Greek dyslipidaemic patients and controls.BACKGROUND: Previous genomic linkage studies have produced evidence linking sodium-lithium countertransport activity (Na/Li CT) with various chromosomal regions including loci harbouring glutathione S transferase (GST) genes. The aim of this study was to examine the putative association of erythrocyte Na/Li CT activity with GST T1 and M1 gene null polymorphisms. METHODS: Na/Li CT activity was determined in erythrocytes isolated from 85 individuals, using a standard assay procedure employing atomic absorption spectroscopy. Genotyping of the GST T1 and GST M1 null polymorphisms was accomplished with a multiplex PCR method. A general linear model using age, sex, smoking, dyslipidaemia and hypertension as covariates was used to examine the association of Na/Li CT activity with the GST T1 and GST M1 genotypes. RESULTS: Individuals with the GST T1 null genotype displayed marginally significantly (p=0.049) lower values of Na/Li CT activity compared to those harbouring at least one copy of the GST T1 gene. The significance of this association was eliminated following adjustment for covariates (p=0.150), but survived as a trend when the sample was limited to normotensive and normolipidaemic individuals (p=0.070). No association was detected between the GST M1 null polymorphism and Na/Li CT activity. CONCLUSIONS: The suggestive association of the GST T1 null polymorphism with erythrocyte Na/Li CT activity is in line with previously published data from genetic linkage and biochemical analyses and may be of potential prognostic value as regards the behaviour of the countertransport and the development of related pathologies under conditions of oxidative insult. |
| 20472602 | Tissue-specific effects of valsartan on rstn and fiaf gene expression in the ob/ob mouse.The RAS is a novel target in the study of diabetes, and clinical trials have indicated that ARBs, such as valsartan, may exert some of their clinical effects through an influence on adipose tissue. We studied the effect of valsartan on adipokine genes resistin (rstn) and fasting-induced adipose factor (fiaf) using obese and diabetic ob/ob mice. In addition to visceral and subcutaneous fat, rstn and fiaf mRNA levels were also measured in several other tissues known to express these adipokines, including the pituitary, cerebral cortex and hypothalamus. The significant findings were that (a) fiaf gene expression was elevated two- to fourfold in visceral and subcutaneous fat from ob/ob mice, compared with lean controls; (b) the increase in fiaf mRNA in subcutaneous, but not visceral, fat from ob/ob mice was returned to lean control levels following 2 weeks of valsartan treatment; (c) fiaf expression was reduced in the hypothalamus, but not in the cortex or pituitary, of ob/ob mice; (d) rstn expression was greatly reduced in visceral fat from ob/ob mice, compared with lean controls, but this was unaffected by valsartan; and (e) rstn expression was unchanged in all other tissues from ob/ob mice, with or without valsartan treatment. |
| 20577625 | Lack of Association between Glutathione S-Transferase-M1, -T1, and -P1 Polymorphisms and Olanzapine-Induced Weight Gain in Korean Schizophrenic Patients.OBJECTIVE: Oxidative stress may be an important pathogenic mechanism in the obesity and metabolic syndrome. The aims of this study was to assess the possible association between the oxidative stress related Glutathione S-Transferase genes (GST-M1, GST-T1, and GST-P1) variants and the olanzapine-induced weight gain in Korean schizophrenic patients. METHODS: We categorized 78 schizophrenic patients into two groups the more than 7% weight gain from baseline (weight gain >/=7%) and the less weight gain (weight gain <7%) groups according to weight change between before and after long-term olanzapine treatment (440+/-288 days). All participants were genotyped for the GST-M1, GST-T1 and GST-P1 genes. Differences in allele frequencies between cohorts with different body weight changes were evaluated by a chi-square analysis and Fisher's exact test. The multifactor dimensionality reduction (MDR) approach was used to analyze gene-gene interactions. RESULTS: Mean body weight gain was 5.42 kg. There was no difference in the null genotype distribution of GST-M1 and -T1 between subjects with body weight gain >/=7% compared to subjects with body weight gain <7% (p>0.05). No significant difference in GST-P1 genotype and allele frequencies were observed between the groups (p>0.05). MDR analysis did not show a significant interaction between the three GST gene variants and susceptibility to weight gain (p>0.05). CONCLUSION: These findings do not support a relationship between the genetic variants of three GST genes (GST-M1, -T1 and -P1) and weight gain in Korean schizophrenic patients receiving olanzapine treatment. |
| 21550991 | Glucosylceramide synthase in the fat body controls energy metabolism in Drosophila.Glucosylceramide synthase (GlcT-1) catalyzes the synthesis of glucosylceramide (GlcCer), the core structure of major glycosphingolipids (GSLs). Obesity is a metabolic disorder caused by an imbalance between energy uptake and expenditure, resulting in excess stored body fat. Recent studies have shown that GSL levels are increased in obese rodents and that pharmacologically reducing GSL levels by inhibiting GlcCer synthesis improves adipocyte function. However, the molecular mechanism underlying these processes is still not clearly understood. Using Drosophila as a model animal, we report that GlcT-1 expression in the fat body, which is equivalent to mammalian adipose tissue, regulates energy metabolism. Overexpression of GlcT-1 increases stored nutrition (triacylglycerol and carbohydrate) levels. Conversely, reduced expression of GlcT-1 in the fat body causes a reduction of fat storage. This regulation occurs, at least in part, through the activation of p38-ATF2 signaling. Furthermore, we found that GlcCer is the sole GSL of the fat body, indicating that regulation of GlcCer synthesis by GlcT-1 in the fat body is responsible for regulating energy homeostasis. Both GlcT-1 and p38-ATF2 signaling are evolutionarily conserved, leading us to propose an evolutionary perspective in which GlcT-1 appears to be one of the key factors that control fat metabolism. |
| 22774989 | Urinary mannose-binding lectin is a biomarker for predicting the progression of immunoglobulin (Ig)A nephropathy.Complement system activation is associated with immunoglobulin A nephropathy (IgAN) activity and progression. The aim of the present study was to investigate the importance of urinary mannose-binding lectin (MBL), at the time of renal biopsy, for evaluating disease severity and predicting the progression of IgAN. A total of 162 patients with biopsy-proven IgAN were enrolled and 50 healthy individuals were selected as normal controls. Urinary MBL was measured by sandwich enzyme-linked immunosorbent assay (ELISA) and normalized for urinary creatinine concentration. Urinary MBL was significantly higher in IgAN patients than that in normal controls, and elevated as histopathological phenotypes upgraded. Urinary MBL was correlated significantly with the well-known clinical predictors for the prognosis of IgAN; that is, renal function (represented by serum creatinine and estimated glomerular filtration rate), proteinuria and arterial hypertension. Urinary MBL was demonstrated to be correlated with the histopathological parameters which have independent value in predicting renal outcome of IgAN according to the Oxford classification; that is, mesangial hypercellularity, segmental glomerulosclerosis, endocapillary hypercellularity and tubular atrophy/interstitial fibrosis. More importantly, non-remission patients at the end of follow-up had significantly higher levels of urinary MBL compared with patients in remission. In conclusion, urinary MBL can be a reliable non-invasive biomarker for evaluating disease severity and predicting the prognosis of IgAN. This is the first report on this issue. However, our conclusions should be verified further in large-scale studies with long-term follow-up. |
| 23040072 | TAp63 is a master transcriptional regulator of lipid and glucose metabolism.TAp63 prevents premature aging, suggesting a link to genes that regulate longevity. Further characterization of TAp63-/- mice revealed that these mice develop obesity, insulin resistance, and glucose intolerance similar to those seen in mice lacking two key metabolic regulators, Silent information regulator T1 (Sirt1) and AMPK. While the roles of Sirt1 and AMPK in metabolism have been well studied, their upstream regulators are not well understood. We found that TAp63 is important in regulating energy metabolism by accumulating in response to metabolic stress and transcriptionally activating Sirt1, AMPKalpha2, and LKB1, resulting in increased fatty acid synthesis and decreased fatty acid oxidation. Moreover, we found that TAp63 lowers blood glucose levels in response to metformin. Restoration of Sirt1, AMPKalpha2, and LKB1 in TAp63-/- mice rescued some of the metabolic defects of the TAp63-/- mice. Our study defines a role for TAp63 in metabolism and weight control. |
| 23315130 | Cannabinoid receptor 1 promotes hepatic lipid accumulation and lipotoxicity through the induction of SREBP-1c expression in zebrafish.The activated cannabinoid receptor 1 (CB1R) is exclusively responsible for food intake and weight gain and regulates several pathological features associated with obesity in mammals. However, the precise role of CB1R in non-mammalian model systems is poorly understood. To investigate the functions of CB1R in zebrafish liver, we conditionally expressed CB1R proteins using a liver-specific Tet(off) transgenic system. In this study, we found hepatic lipid accumulation in CB1R transgenic zebrafish (CB) without doxycycline treatment (-Dox) and a suppression of CB1R expression, resulting in the loss of lipid accumulation in the livers of CB fish that received doxycycline treatment (+Dox). Oil Red O (ORO)-stained hepatocytes were predominant in the liver buds of CB-Dox larvae, indicating that CB1R functionally promotes lipid accumulation during CB hepatogenesis. More than 73 % of CB-Dox adults showed increased lipid content, which leads, in turn, to steatosis. Liver histology and ORO staining of CB-Dox hepatocytes also indicated the accumulation of fatty droplets in the CB liver samples, consistent with the specific pathological features of liver steatosis or steatohepatitis. We also found that hepatic CB1R overexpression accompanies the stimulation of the lipogenic transcription factor SREBP-1c and its target enzymes, acetyl coenzyme-A carboxylase-1 (ACC1) and fatty acid synthase (FAS), and increases de novo fatty acid synthesis. This study is the first to report CB1R as a potential hepatic stimulator for zebrafish liver steatosis. |
| 23750697 | Glomerular mannose-binding lectin deposition is a useful prognostic predictor in immunoglobulin A nephropathy.There is accumulating evidence to support a hypothesis of the activation of the lectin complement pathway in immunoglobulin A nephropathy (IgAN). The glomerular deposition of mannose-binding lectin (MBL), an initiator of the lectin pathway, has been identified, but its clinical significance has not been defined consistently. The aim of the present study was to investigate the value of glomerular MBL deposition as a useful histological biomarker in evaluating the severity and predicting the prognosis of IgAN. We included all consecutive patients with biopsy-proven primary IgAN from December 2008 to July 2010. Renal deposition of MBL was detected by immunofluorescence. The biopsy material from 131 patients (72 men) was thus used for MBL staining. The deposition of MBL was observed in a predominantly mesangial pattern in 45 patients (34.35%), which presented as global or segmental deposition. Compared with the patients without glomerular MBL deposition, those with glomerular MBL deposition had more severe proteinuria, decreased renal function, lower levels of serum albumin and a greater possibility of hypertension at the time of renal biopsy; they had more severe histological changes according to the Oxford classification (i.e. mesangial hypercellularity, segmental glomerulosclerosis, endocapillary hypercellularity and tubular atrophy/interstitial fibrosis), and their ratio presented an increase as the histopathological phenotypes segregated according to Lee's classification; furthermore, the follow-up data demonstrated that they had a lower renal remission rate. In conclusion, glomerular MBL deposition may predict a poor prognosis, and thus can be a new prognostic factor in IgA nephropathy. |
| 21281477 | Mannose-binding lectin does not explain the course and outcome of pregnancy in rheumatoid arthritis.INTRODUCTION: Rheumatoid arthritis (RA) improves during pregnancy and flares after delivery. It has been hypothesized that high levels of the complement factor mannose-binding lectin (MBL) are associated with a favourable disease course of RA by facilitating the clearance of pathogenic immunoglobulin G (IgG) lacking galactose sugar moieties. During pregnancy, increased galactosylation of IgG and simultaneously increased MBL levels can be observed, with the latter being strictly related to maternal MBL genotypes. Therefore, increased MBL levels in concert with increased IgG galactosylation may be associated with pregnancy-induced improvement of RA. The objective of this study was to investigate whether MBL genotypes are associated with changes in RA disease activity and with changes in IgG galactosylation during pregnancy and in the postpartum period. We also studied the association between MBL genotypes and pregnancy outcomes in RA. METHODS: Serum from 216 patients with RA and 31 healthy controls participating in the Pregnancy-induced Amelioration of Rheumatoid Arthritis (PARA) Study was collected before, during and after pregnancy. IgG galactosylation was determined by performing matrix-assisted laser desorption/ionization time of flight mass spectrometry. Disease activity was determined using the internationally recognized Disease Activity Score 28 (DAS28). MBL genotypes were determined. The pregnancy outcome measures studied were gestational age, birth weight, miscarriage and hypertensive disorders. RESULTS: No association was found between the MBL genotype groups and changes in RA disease activity (P = 0.89) or changes in IgG galactosylation (patients, P = 0.75, and controls, P = 0.54) during pregnancy and in the postpartum period. Furthermore, MBL genotype groups were not related to the studied pregnancy outcome measures. CONCLUSIONS: This study does not provide evidence for a role for MBL in the improvement of RA during pregnancy or for a role for MBL in pregnancy outcome. |
| 22441934 | Overexpression and ratio disruption of DeltaNp63 and TAp63 isoform equilibrium in endometrial adenocarcinoma: correlation with obesity, menopause, and grade I/II tumors.PURPOSE: p63 plays an important role in several intracellular processes such as transcription activation and apoptosis. p63 has two N-terminal isoforms, TAp63 and DeltaNp63. TAp63 isoform has p53-like functions, while DeltaNp63 acts as a dominant negative inhibitor of the p53 family and is considered oncogenic. Although p63 and its isoforms are overexpressed in a wide variety of human malignancies such as cervical, head and neck, and lung cancer, their role in endometrial carcinoma has not been investigated. METHODS: We measured by quantitative real-time polymerase chain reaction the mRNA expression of TAp63 and DeltaNp63 in a series of 20 endometrioid adenocarcinomas paired with adjacent normal tissue. RESULTS: TAp63 isoform exhibited 1.8-fold overexpression in malignant samples, while DeltaNp63 was 4.3-fold overexpressed in cancer specimens. Further analysis revealed that the DeltaN/TA isoform ratio shifted from 0.5 in normal samples to 1.2 in tumor specimens. Statistical analysis also revealed an association of TAp63 expression with high body mass index (p = 0.034), late menopause (p = 0.020), and lower tumor grade (p = 0.034). DeltaNp63 was also correlated with grade I/II tumors (p = 0.044). CONCLUSIONS: These results indicate that both p63 isoforms and especially DeltaNp63 play an important role in the development and progression of grade I/II endometrial adenocarcinoma, especially in obese and late-menopause women. |

  
  
----- Star papers (those papers include more than 100 genes) -----  

|  |  |
| --- | --- |
| 20682687 | Common variants in 40 genes assessed for diabetes incidence and response to metformin and lifestyle intervention in the diabetes prevention program.OBJECTIVE: Genome-wide association studies have begun to elucidate the genetic architecture of type 2 diabetes. We examined whether single nucleotide polymorphisms (SNPs) identified through targeted complementary approaches affect diabetes incidence in the at-risk population of the Diabetes Prevention Program (DPP) and whether they influence a response to preventive interventions. RESEARCH DESIGN AND METHODS: We selected SNPs identified by prior genome-wide association studies for type 2 diabetes and related traits, or capturing common variation in 40 candidate genes previously associated with type 2 diabetes, implicated in monogenic diabetes, encoding type 2 diabetes drug targets or drug-metabolizing/transporting enzymes, or involved in relevant physiological processes. We analyzed 1,590 SNPs for association with incident diabetes and their interaction with response to metformin or lifestyle interventions in 2,994 DPP participants. We controlled for multiple hypothesis testing by assessing false discovery rates. RESULTS: We replicated the association of variants in the metformin transporter gene SLC47A1 with metformin response and detected nominal interactions in the AMP kinase (AMPK) gene STK11, the AMPK subunit genes PRKAA1 and PRKAA2, and a missense SNP in SLC22A1, which encodes another metformin transporter. The most significant association with diabetes incidence occurred in the AMPK subunit gene PRKAG2 (hazard ratio 1.24, 95% CI 1.09-1.40, P = 7 x 10(-4)). Overall, there were nominal associations with diabetes incidence at 85 SNPs and nominal interactions with the metformin and lifestyle interventions at 91 and 69 mostly nonoverlapping SNPs, respectively. The lowest P values were consistent with experiment-wide 33% false discovery rates. CONCLUSIONS: We have identified potential genetic determinants of metformin response. These results merit confirmation in independent samples. |
| 23378610 | Genetic variants associated with glycine metabolism and their role in insulin sensitivity and type 2 diabetes.Circulating metabolites associated with insulin sensitivity may represent useful biomarkers, but their causal role in insulin sensitivity and diabetes is less certain. We previously identified novel metabolites correlated with insulin sensitivity measured by the hyperinsulinemic-euglycemic clamp. The top-ranking metabolites were in the glutathione and glycine biosynthesis pathways. We aimed to identify common genetic variants associated with metabolites in these pathways and test their role in insulin sensitivity and type 2 diabetes. With 1,004 nondiabetic individuals from the RISC study, we performed a genome-wide association study (GWAS) of 14 insulin sensitivity-related metabolites and one metabolite ratio. We replicated our results in the Botnia study (n = 342). We assessed the association of these variants with diabetes-related traits in GWAS meta-analyses (GENESIS [including RISC, EUGENE2, and Stanford], MAGIC, and DIAGRAM). We identified four associations with three metabolites-glycine (rs715 at CPS1), serine (rs478093 at PHGDH), and betaine (rs499368 at SLC6A12; rs17823642 at BHMT)-and one association signal with glycine-to-serine ratio (rs1107366 at ALDH1L1). There was no robust evidence for association between these variants and insulin resistance or diabetes. Genetic variants associated with genes in the glycine biosynthesis pathways do not provide consistent evidence for a role of glycine in diabetes-related traits. |
| 12732844 | A study to survey susceptible genetic factors responsible for troglitazone-associated hepatotoxicity in Japanese patients with type 2 diabetes mellitus.BACKGROUND AND OBJECTIVE: Troglitazone is a 2,4-thiazolidinedione antidiabetic agent with insulin-sensitizing activities. This agent had been used efficiently in a large number of patients but was withdrawn from the market in March 2000 because of its association with idiosyncratic hepatotoxicity. To address the susceptible genetic factors responsible for the hepatotoxicity associated with this agent, we performed a genetic polymorphic analysis by a target gene approach in troglitazone-treated Japanese patients with type 2 diabetes mellitus. METHODS: One hundred ten patients treated with troglitazone were recruited into this study. The case patients (n = 25) were recruited through medical professionals who had previously reported abnormal increases in the levels of ALT or AST among their patients. The control patients (n = 85) were recruited through physicians prescribing troglitazone. For statistical accuracy, efforts were made to maximize the size of the case group. Genotype analysis was performed in 68 polymorphic sites of 51 candidate genes related to drug metabolism, apoptosis, roduction and elimination of reactive oxygen species, and signal transduction pathways of peroxisome proliferator-activated receptor gamma 2 and insulin. RESULTS: The strong correlation with transaminase elevations was observed in the combined glutathione-S-transferase GSTT1-GSTM1 null genotype (odds ratio, 3.692; 95% confidence interval, 1.354-10.066; P =.008). CONCLUSIONS: The double null mutation of GSTT1 and GSTM1 might influence troglitazone-associated abnormal increases of liver enzyme levels. |
| 15466941 | The cellular fate of glucose and its relevance in type 2 diabetes.Type 2 diabetes is a complex disorder with diminished insulin secretion and insulin action contributing to the hyperglycemia and wide range of metabolic defects that underlie the disease. The contribution of glucose metabolic pathways per se in the pathogenesis of the disease remains unclear. The cellular fate of glucose begins with glucose transport and phosphorylation. Subsequent pathways of glucose utilization include aerobic and anaerobic glycolysis, glycogen formation, and conversion to other intermediates in the hexose phosphate or hexosamine biosynthesis pathways. Abnormalities in each pathway may occur in diabetic subjects; however, it is unclear whether perturbations in these may lead to diabetes or are a consequence of the multiple metabolic abnormalities found in the disease. This review is focused on the cellular fate of glucose and relevance to human type 2 diabetes. |
| 17554300 | Genome-wide association study of 14,000 cases of seven common diseases and 3,000 shared controls.There is increasing evidence that genome-wide association (GWA) studies represent a powerful approach to the identification of genes involved in common human diseases. We describe a joint GWA study (using the Affymetrix GeneChip 500K Mapping Array Set) undertaken in the British population, which has examined approximately 2,000 individuals for each of 7 major diseases and a shared set of approximately 3,000 controls. Case-control comparisons identified 24 independent association signals at P < 5 x 10(-7): 1 in bipolar disorder, 1 in coronary artery disease, 9 in Crohn's disease, 3 in rheumatoid arthritis, 7 in type 1 diabetes and 3 in type 2 diabetes. On the basis of prior findings and replication studies thus-far completed, almost all of these signals reflect genuine susceptibility effects. We observed association at many previously identified loci, and found compelling evidence that some loci confer risk for more than one of the diseases studied. Across all diseases, we identified a large number of further signals (including 58 loci with single-point P values between 10(-5) and 5 x 10(-7)) likely to yield additional susceptibility loci. The importance of appropriately large samples was confirmed by the modest effect sizes observed at most loci identified. This study thus represents a thorough validation of the GWA approach. It has also demonstrated that careful use of a shared control group represents a safe and effective approach to GWA analyses of multiple disease phenotypes; has generated a genome-wide genotype database for future studies of common diseases in the British population; and shown that, provided individuals with non-European ancestry are excluded, the extent of population stratification in the British population is generally modest. Our findings offer new avenues for exploring the pathophysiology of these important disorders. We anticipate that our data, results and software, which will be widely available to other investigators, will provide a powerful resource for human genetics research. |
| 20628086 | Variation at the NFATC2 locus increases the risk of thiazolidinedione-induced edema in the Diabetes REduction Assessment with ramipril and rosiglitazone Medication (DREAM) study.OBJECTIVE: Thiazolidinediones are used to treat type 2 diabetes. Their use has been associated with peripheral edema and congestive heart failure-outcomes that may have a genetic etiology. RESEARCH DESIGN AND METHODS: We genotyped 4,197 participants of the multiethnic DREAM (Diabetes REduction Assessment with ramipril and rosiglitazone Medication) trial with a 50k single nucleotide polymorphisms (SNP) array, which captures approximately 2000 cardiovascular, inflammatory, and metabolic genes. We tested 32,088 SNPs for an association with edema among Europeans who received rosiglitazone (n = 965). RESULTS: One SNP, rs6123045, in NFATC2 was significantly associated with edema (odds ratio 1.89 [95% CI 1.47-2.42]; P = 5.32 x 10(-7), corrected P = 0.017). Homozygous individuals had the highest edema rate (hazard ratio 2.89, P = 4.22 x 10(-4)) when compared with individuals homozygous for the protective allele, with heterozygous individuals having an intermediate risk. The interaction between the SNP and rosiglitazone for edema was significant (P = 7.68 x 10(-3)). Six SNPs in NFATC2 were significant in both Europeans and Latin Americans (P < 0.05). CONCLUSIONS: Genetic variation at the NFATC2 locus contributes to edema among individuals who receive rosiglitazone. |
| 14610273 | Wnk1 kinase deficiency lowers blood pressure in mice: a gene-trap screen to identify potential targets for therapeutic intervention.The availability of both the mouse and human genome sequences allows for the systematic discovery of human gene function through the use of the mouse as a model system. To accelerate the genetic determination of gene function, we have developed a sequence-tagged gene-trap library of >270,000 mouse embryonic stem cell clones representing mutations in approximately 60% of mammalian genes. Through the generation and phenotypic analysis of knockout mice from this resource, we are undertaking a functional screen to identify genes regulating physiological parameters such as blood pressure. As part of this screen, mice deficient for the Wnk1 kinase gene were generated and analyzed. Genetic studies in humans have shown that large intronic deletions in WNK1 lead to its overexpression and are responsible for pseudohypoaldosteronism type II, an autosomal dominant disorder characterized by hypertension, increased renal salt reabsorption, and impaired K+ and H+ excretion. Consistent with the human genetic studies, Wnk1 heterozygous mice displayed a significant decrease in blood pressure. Mice homozygous for the Wnk1 mutation died during embryonic development before day 13 of gestation. These results demonstrate that Wnk1 is a regulator of blood pressure critical for development and illustrate the utility of a functional screen driven by a sequence-based mutagenesis approach. |
| 14662765 | ATP-citrate lyase deficiency in the mouse.ATP-citrate lyase (Acly) is one of two cytosolic enzymes that synthesize acetyl-coenzyme A (CoA). Because acetyl-CoA is an essential building block for cholesterol and triglycerides, Acly has been considered a therapeutic target for hyperlipidemias and obesity. To define the phenotype of Acly-deficient mice, we created Acly knockout mice in which a beta-galactosidase marker is expressed from Acly regulatory sequences. We also sought to define the cell type-specific expression patterns of Acly to further elucidate the in vivo roles of the enzyme. Homozygous Acly knockout mice died early in development. Heterozygous mice were healthy, fertile, and normolipidemic on both chow and high fat diets, despite expressing half-normal amounts of Acly mRNA and protein. Fibroblasts and hepatocytes from heterozygous Acly mice contained half-normal amounts of Acly mRNA and protein, but this did not perturb triglyceride and cholesterol synthesis or the expression of lipid biosynthetic genes regulated by sterol regulatory element-binding proteins. The expression of acetyl-CoA synthetase 1, another cytosolic enzyme for producing acetyl-CoA, was not up-regulated. As judged by beta-galactosidase staining, Acly was expressed ubiquitously but was expressed particularly highly in tissues with high levels of lipogenesis, such as in the livers of mice fed a high-carbohydrate diet. beta-Galactosidase staining was intense in the developing brain, in keeping with the high levels of de novo lipogenesis of the tissue. In the adult brain, beta-galactosidase staining was in general much lower, consistent with reduced levels of lipogenesis; however, beta-galactosidase expression remained very high in cholinergic neurons, likely reflecting the importance of Acly in generating acetyl-CoA for acetylcholine synthesis. The Acly knockout allele is useful for identifying cell types with a high demand for acetyl-CoA synthesis. |
| 20346360 | Genetic risk factors for hepatopulmonary syndrome in patients with advanced liver disease.BACKGROUND & AIMS: Hepatopulmonary syndrome (HPS) affects 10%-30% of patients with cirrhosis and portal hypertension and significantly increases mortality. Studies in experimental models indicate that pulmonary angiogenesis contributes to the development of HPS, but pathogenesis in humans is poorly understood. We investigated genetic risk factors for HPS in patients with advanced liver disease. METHODS: We performed a multicenter case-control study of patients with cirrhosis being evaluated for liver transplantation. Cases had an alveolar-arterial oxygen gradient > or = 15 mm Hg (or > or =20 mm Hg if age > 64 years) and contrast echocardiography with late appearance of microbubbles after venous injection of agitated saline (intrapulmonary vasodilatation); controls did not meet both criteria for case status. The study sample included 59 cases and 126 controls. We genotyped 1086 common single nucleotide polymorphisms (SNPs) in 94 candidate genes. RESULTS: Forty-two SNPs in 21 genes were significantly associated with HPS after adjustments for race and smoking. Eight genes had at least 2 SNPs associated with disease: CAV3, ENG, NOX4, ESR2, VWF, RUNX1, COL18A1, and TIE1. For example, rs237872 in CAV3 showed an odds ratio of 2.75 (95% confidence interval: 1.65-4.60, P = .0001) and rs4837192 in ENG showed an odds ratio of 0.35 (95% confidence interval: 0.14-0.89, P = .027). Furthermore, variation in CAV3 and RUNX1 was associated with HPS in gene-based analyses. CONCLUSIONS: Polymorphisms in genes involved in the regulation of angiogenesis are associated with the risk of HPS. Further investigation of these biologic pathways might elucidate the mechanisms that mediate the development of HPS in certain patients with severe liver disease. |
| 20654748 | High-density polymorphisms analysis of 23 candidate genes for association with bone mineral density.Osteoporosis is a bone disease characterized by low bone mineral density (BMD), a highly heritable and polygenic trait. Women are more prone than men to develop osteoporosis due to a lower peak bone mass and accelerated bone loss at menopause. Peak bone mass has been convincingly shown to be due to genetic factors with heritability up to 80%. Menopausal bone loss has been shown to have around 38% to 49% heritability depending on the site studied. To have more statistical power to detect small genetic effects we focused on premenopausal women. We studied 23 candidate genes, some involved in calcium and vitamin-D regulation and others because estrogens strongly induced their gene expression in mice where it was correlated with humerus trabecular bone density. High-density polymorphisms were selected to cover the entire gene variability and 231 polymorphisms were genotyped in a first sample of 709 premenopausal women. Positive associations were retested in a second, independent, sample of 673 premenopausal women. Ten polymorphisms remained associated with BMD in the combined samples and one was further associated in a large sample of postmenopausal women (1401 women). This associated polymorphism was located in the gene CSF3R (granulocyte colony stimulating factor receptor) that had never been associated with BMD before. The results reported in this study suggest a role for CSF3R in the determination of bone density in women. |

Copyright © CoCiter 2011-2013. >>
Designed by QIAO Nan & HUANG Yi >>
Hanlab
