# Supplementary material for: Partial depletion of yolk during zebrafish embryogenesis changes the dynamics of methionine cycle and metabolic genes
Source: BMC Genomics. 2015 Jun 4;16(1):427. doi: 10.1186/s12864-015-1654-6 (PMC4455928; doi:10.1186/s12864-015-1654-6)
Supplement: Additional file 10: — CoCiter output of differentially expressed genes at 32 hpf. RNAseq outcome associated to the terms “hypertension”, “obesity”, “type 2 diabetes” and “osteoporosis”. [file 12864_2015_1654_MOESM10_ESM.html]

CoCiter v1.1


**CoCiter v1.1**

## Co-citation analysis on genes/terms

Home
Gene-Gene
Gene-Term
Term-Term
ViewJob
HanLab

## Your search results:

**Genes/Terms involved in mistake below are not calculated in this result.**
Genes 100535681, 794874 in the Gene Set are not included in the database of that species!

| Set | Genes/Terms | PubMed Count | CI | Significance |
| --- | --- | --- | --- | --- |
| GeneSet 225 genes | apoeb (30314), rbp4 (30077), nos1 (60658), igf2bp2a (100136839), f2 (325881), c3a (321046), abca1b (100136868), gstt1a (563972), fgb (337315), itgb3b (100001836), pck1 (378727), mtp (406207), cp (84702), angptl4 (492647), pck2 (406473), txnipa (368359), si:ch211-236l14.3 (560000), fga (378986), nphs1 (692352), alox5 (567204), tbc1d1 (561193), g6pca.2 (563180), npc1 (553330), cfd (553553), ptch1 (30189), ctsba (406645), bhmt (322228), hsp90aa1.2 (565155), atp1b1b (64273), ctsla (321453), cfb (30604), atp6ap1b (195824), wu:fb66f03 (100000085), ppp1r3b (327285), c5 (565774), ctsd (65225), il13ra2 (368326), tfr1a (494477), arrdc3a (566685), hdc (793609), slc26a2 (557046), junbb (336038), a2ml (100006972), sulf2l (322056), LOC100006895 (100006895), vtna (553699), elovl5 (393425), vtnb (403026), spint1a (406426), mmp30 (678531), oxct1b (560273), hexa (323613), fmodb (797559), trpv6 (100000322), zgc:92480 (445091), s1pr1 (64617), lum (415149), dpysl5a (324416), slc43a2b (494042), diabloa (393317), psap (140811), mrc1a (100286774), f9b (678552), ces2 (566132), cited2 (450024), sesn3 (406840), ppifb (641328), ambp (394093), rfx2 (503590), ptrfb (561082), pla2g15 (335008), il1b (405770), osbpl2b (334206), rpia (407673), trdmt1 (449878), si:ch1073-473i7.3 (793799), hao2 (393455), slc16a9a (795588), si:ch211-5k11.2 (572729), atp6v1ab (337583), lix1 (558010), zgc:153913 (559700), a1cf (562916), LOC563738 (563738), ccdc6b (393211), ckba (564694), suclg2 (317746), myhz2 (246275), g3bp2 (556409), spns1 (799873), galns (791159), ptprna (324790), LOC100334657 (100334657), zgc:153405 (751661), tex2 (798401), man2b1 (541519), zgc:112084 (550460), spon2b (30202), pqlc2 (503709), mfsd8 (564342), ascc1 (550273), pabpc1b (393856), slc16a9b (445158), lix1l (572161), mst1 (259260), ppl (449936), ap4m1 (436945), ppm1h (768291), zgc:110216 (541350), ddhd2 (100321082), mknk2a (334167), sult2st1 (338214), slc38a7 (445254), sar1ab (550581), miox (571850), pi4k2a (406667), cox6b1 (436968), hbae1 (30597), tmem63ba (100334023), tktb (378713), LOC100334599 (100334599), LOC100535653 (100535653), c8a (445102), LOC100536856 (100536856), gpd1c (406615), apoc2 (568972), actc1a (408256), itpr3 (794666), LOC100536075 (100536075), iqgap2 (563030), LOC100538221 (100538221), si:busm1-118j2.5 (335781), wu:fd11e11 (386799), gbp1 (436615), si:ch211-140f21.1 (566347), pfkfb4l (386663), tubb1 (558552), ckmt1 (321892), si:ch211-251f6.6 (794692), rasd1 (393504), zgc:110239 (550326), LOC573653 (573653), hbbe1.1 (81538), gnmt (403338), sardh (394103), rcan2 (407988), pgk1 (406696), slc4a1a (84703), LOC100332590 (100332590), LOC792958 (792958), pla2g12b (406739), zgc:174237 (100126125), c9 (554141), LOC100330858 (100330858), fn1b (334613), si:ch211-129c21.1 (563087), zgc:103681 (449773), kng1 (449808), apoa1b (100101640), prss16 (556307), zgc:174904 (564690), spg20b (751741), plbd1 (569736), qsox1 (100004382), tmem47 (406640), ponzr3 (100004954), itgb1b.1 (566771), kti12 (751662), slc27a2a (449925), zgc:152945 (767787), sept10 (550129), abcb10 (100003744), rpz5 (100003142), dhrs13b (445116), LOC559569 (559569), fam20a (564308), si:ch211-284e20.8 (100093712), ankrd37 (793783), f7 (114423), cyp2aa9 (790957), LOC100333818 (100333818), atp1a1a.2 (64613), klf11b (559076), galca (449649), hm:zewp0073 (337770), zgc:162608 (100037332), LOC100001718 (100001718), apom (569714), serhl (322648), zgc:114181 (619255), MGC173646 (793447), sall1a (555721), dcxr (550282), si:ch211-117m20.5 (566658), abcb6a (564067), neurod4 (266958), p4ha1b (100003675), fetub (569489), zgc:174153 (567623), im:7148400 (100003770), abca12 (558335), amdhd2 (402981), sb:cb252 (321177), LOC100537515 (100537515), gsnb (336623), LOC100538072 (100538072), hbbe2 (405772), zgc:92380 (445086), rgs2 (436935), zgc:158494 (386720), LOC792966 (792966), hspb9 (100137108), slc25a25a (406541), hao1 (402827), rab14 (373104), zgc:158614 (791218), si:dkeyp-75b4.9 (100321606), apobb (321166), hs3st1l2 (562344), zgc:173443 (100126130), msgn1 (360135), si:dkey-239j18.3 (100001785), ponzr4 (557748), lygl1 (436979), si:dkeyp-75h12.2 (559449) | 1339 | 10.388 | **p-value** 0.002\*\* **permutation** 1000 x **adjusted CI** 2.6186 |
| TermSet 4 terms | obesity, type 2 diabetes, hypertension, osteoporosis |

- (p-value significance: \*\*\*: p < 0.001; \*\*: p < 0.005; \*: p < 0.01)
  

The one-to-all results.

| Gene v.s. all terms | Term v.s. all Genes |
| --- | --- |
| | Gene | Count | CI | | --- | --- | --- | |    apoeb (30314) | 403 | 8.6582 | |    rbp4 (30077) | 132 | 7.0553 | |    nos1 (60658) | 104 | 6.7142 | |    igf2bp2a (100136839) | 94 | 6.5699 | |    f2 (325881) | 71 | 6.1699 | |    c3a (321046) | 52 | 5.7279 | |    abca1b (100136868) | 52 | 5.7279 | |    gstt1a (563972) | 42 | 5.4263 | |    fgb (337315) | 38 | 5.2854 | |    itgb3b (100001836) | 37 | 5.2479 | |    pck1 (378727) | 37 | 5.2479 | |    mtp (406207) | 29 | 4.9069 | |    cp (84702) | 25 | 4.7004 | |    angptl4 (492647) | 24 | 4.6439 | |    pck2 (406473) | 24 | 4.6439 | |    txnipa (368359) | 20 | 4.3923 | |    si:ch211-236l14.3 (560000) | 19 | 4.3219 | |    fga (378986) | 16 | 4.0875 | |    nphs1 (692352) | 16 | 4.0875 | |    alox5 (567204) | 16 | 4.0875 | |    tbc1d1 (561193) | 15 | 4.0 | |    g6pca.2 (563180) | 14 | 3.9069 | |    npc1 (553330) | 14 | 3.9069 | |    cfd (553553) | 11 | 3.585 | |    ptch1 (30189) | 9 | 3.3219 | |    ctsba (406645) | 9 | 3.3219 | |    bhmt (322228) | 9 | 3.3219 | |    hsp90aa1.2 (565155) | 9 | 3.3219 | |    atp1b1b (64273) | 8 | 3.1699 | |    ctsla (321453) | 8 | 3.1699 | |    cfb (30604) | 7 | 3.0 | |    atp6ap1b (195824) | 6 | 2.8074 | |    wu:fb66f03 (100000085) | 6 | 2.8074 | |    ppp1r3b (327285) | 5 | 2.585 | |    c5 (565774) | 5 | 2.585 | |    ctsd (65225) | 5 | 2.585 | |    il13ra2 (368326) | 5 | 2.585 | |    tfr1a (494477) | 5 | 2.585 | |    arrdc3a (566685) | 4 | 2.3219 | |    hdc (793609) | 4 | 2.3219 | |    slc26a2 (557046) | 4 | 2.3219 | |    junbb (336038) | 4 | 2.3219 | |    a2ml (100006972) | 4 | 2.3219 | |    sulf2l (322056) | 4 | 2.3219 | |    LOC100006895 (100006895) | 4 | 2.3219 | |    vtna (553699) | 4 | 2.3219 | |    elovl5 (393425) | 4 | 2.3219 | |    vtnb (403026) | 4 | 2.3219 | |    spint1a (406426) | 3 | 2.0 | |    mmp30 (678531) | 3 | 2.0 | |    oxct1b (560273) | 3 | 2.0 | |    hexa (323613) | 3 | 2.0 | |    fmodb (797559) | 3 | 2.0 | |    trpv6 (100000322) | 3 | 2.0 | |    zgc:92480 (445091) | 3 | 2.0 | |    s1pr1 (64617) | 3 | 2.0 | |    lum (415149) | 3 | 2.0 | |    dpysl5a (324416) | 3 | 2.0 | |    slc43a2b (494042) | 2 | 1.585 | |    diabloa (393317) | 2 | 1.585 | |    psap (140811) | 2 | 1.585 | |    mrc1a (100286774) | 2 | 1.585 | |    f9b (678552) | 2 | 1.585 | |    ces2 (566132) | 2 | 1.585 | |    cited2 (450024) | 2 | 1.585 | |    sesn3 (406840) | 2 | 1.585 | |    ppifb (641328) | 2 | 1.585 | |    ambp (394093) | 2 | 1.585 | |    rfx2 (503590) | 2 | 1.585 | |    ptrfb (561082) | 2 | 1.585 | |    pla2g15 (335008) | 2 | 1.585 | |    il1b (405770) | 2 | 1.585 | |    osbpl2b (334206) | 2 | 1.585 | |    rpia (407673) | 2 | 1.585 | |    trdmt1 (449878) | 2 | 1.585 | |    si:ch1073-473i7.3 (793799) | 1 | 1.0 | |    hao2 (393455) | 1 | 1.0 | |    slc16a9a (795588) | 1 | 1.0 | |    si:ch211-5k11.2 (572729) | 1 | 1.0 | |    atp6v1ab (337583) | 1 | 1.0 | |    lix1 (558010) | 1 | 1.0 | |    zgc:153913 (559700) | 1 | 1.0 | |    a1cf (562916) | 1 | 1.0 | |    LOC563738 (563738) | 1 | 1.0 | |    ccdc6b (393211) | 1 | 1.0 | |    ckba (564694) | 1 | 1.0 | |    suclg2 (317746) | 1 | 1.0 | |    myhz2 (246275) | 1 | 1.0 | |    g3bp2 (556409) | 1 | 1.0 | |    spns1 (799873) | 1 | 1.0 | |    galns (791159) | 1 | 1.0 | |    ptprna (324790) | 1 | 1.0 | |    LOC100334657 (100334657) | 1 | 1.0 | |    zgc:153405 (751661) | 1 | 1.0 | |    tex2 (798401) | 1 | 1.0 | |    man2b1 (541519) | 1 | 1.0 | |    zgc:112084 (550460) | 1 | 1.0 | |    spon2b (30202) | 1 | 1.0 | |    pqlc2 (503709) | 1 | 1.0 | |    mfsd8 (564342) | 1 | 1.0 | |    ascc1 (550273) | 1 | 1.0 | |    pabpc1b (393856) | 1 | 1.0 | |    slc16a9b (445158) | 1 | 1.0 | |    lix1l (572161) | 1 | 1.0 | |    mst1 (259260) | 1 | 1.0 | |    ppl (449936) | 1 | 1.0 | |    ap4m1 (436945) | 1 | 1.0 | |    ppm1h (768291) | 1 | 1.0 | |    zgc:110216 (541350) | 1 | 1.0 | |    ddhd2 (100321082) | 1 | 1.0 | |    mknk2a (334167) | 1 | 1.0 | |    sult2st1 (338214) | 1 | 1.0 | |    slc38a7 (445254) | 1 | 1.0 | |    sar1ab (550581) | 1 | 1.0 | |    miox (571850) | 1 | 1.0 | |    pi4k2a (406667) | 1 | 1.0 | |    cox6b1 (436968) | 1 | 1.0 | |    hbae1 (30597) | 1 | 1.0 | |    tmem63ba (100334023) | 1 | 1.0 | |    tktb (378713) | 1 | 1.0 | |    LOC100334599 (100334599) | 1 | 1.0 | |    LOC100535653 (100535653) | 1 | 1.0 | |    c8a (445102) | 1 | 1.0 | |    LOC100536856 (100536856) | 1 | 1.0 | |    gpd1c (406615) | 0 | 0.0 | |    apoc2 (568972) | 0 | 0.0 | |    actc1a (408256) | 0 | 0.0 | |    itpr3 (794666) | 0 | 0.0 | |    LOC100536075 (100536075) | 0 | 0.0 | |    iqgap2 (563030) | 0 | 0.0 | |    LOC100538221 (100538221) | 0 | 0.0 | |    si:busm1-118j2.5 (335781) | 0 | 0.0 | |    wu:fd11e11 (386799) | 0 | 0.0 | |    gbp1 (436615) | 0 | 0.0 | |    si:ch211-140f21.1 (566347) | 0 | 0.0 | |    pfkfb4l (386663) | 0 | 0.0 | |    tubb1 (558552) | 0 | 0.0 | |    ckmt1 (321892) | 0 | 0.0 | |    si:ch211-251f6.6 (794692) | 0 | 0.0 | |    rasd1 (393504) | 0 | 0.0 | |    zgc:110239 (550326) | 0 | 0.0 | |    LOC573653 (573653) | 0 | 0.0 | |    hbbe1.1 (81538) | 0 | 0.0 | |    gnmt (403338) | 0 | 0.0 | |    sardh (394103) | 0 | 0.0 | |    rcan2 (407988) | 0 | 0.0 | |    pgk1 (406696) | 0 | 0.0 | |    slc4a1a (84703) | 0 | 0.0 | |    LOC100332590 (100332590) | 0 | 0.0 | |    LOC792958 (792958) | 0 | 0.0 | |    pla2g12b (406739) | 0 | 0.0 | |    zgc:174237 (100126125) | 0 | 0.0 | |    c9 (554141) | 0 | 0.0 | |    LOC100330858 (100330858) | 0 | 0.0 | |    fn1b (334613) | 0 | 0.0 | |    si:ch211-129c21.1 (563087) | 0 | 0.0 | |    zgc:103681 (449773) | 0 | 0.0 | |    kng1 (449808) | 0 | 0.0 | |    apoa1b (100101640) | 0 | 0.0 | |    prss16 (556307) | 0 | 0.0 | |    zgc:174904 (564690) | 0 | 0.0 | |    spg20b (751741) | 0 | 0.0 | |    plbd1 (569736) | 0 | 0.0 | |    qsox1 (100004382) | 0 | 0.0 | |    tmem47 (406640) | 0 | 0.0 | |    ponzr3 (100004954) | 0 | 0.0 | |    itgb1b.1 (566771) | 0 | 0.0 | |    kti12 (751662) | 0 | 0.0 | |    slc27a2a (449925) | 0 | 0.0 | |    zgc:152945 (767787) | 0 | 0.0 | |    sept10 (550129) | 0 | 0.0 | |    abcb10 (100003744) | 0 | 0.0 | |    rpz5 (100003142) | 0 | 0.0 | |    dhrs13b (445116) | 0 | 0.0 | |    LOC559569 (559569) | 0 | 0.0 | |    fam20a (564308) | 0 | 0.0 | |    si:ch211-284e20.8 (100093712) | 0 | 0.0 | |    ankrd37 (793783) | 0 | 0.0 | |    f7 (114423) | 0 | 0.0 | |    cyp2aa9 (790957) | 0 | 0.0 | |    LOC100333818 (100333818) | 0 | 0.0 | |    atp1a1a.2 (64613) | 0 | 0.0 | |    klf11b (559076) | 0 | 0.0 | |    galca (449649) | 0 | 0.0 | |    hm:zewp0073 (337770) | 0 | 0.0 | |    zgc:162608 (100037332) | 0 | 0.0 | |    LOC100001718 (100001718) | 0 | 0.0 | |    apom (569714) | 0 | 0.0 | |    serhl (322648) | 0 | 0.0 | |    zgc:114181 (619255) | 0 | 0.0 | |    MGC173646 (793447) | 0 | 0.0 | |    sall1a (555721) | 0 | 0.0 | |    dcxr (550282) | 0 | 0.0 | |    si:ch211-117m20.5 (566658) | 0 | 0.0 | |    abcb6a (564067) | 0 | 0.0 | |    neurod4 (266958) | 0 | 0.0 | |    p4ha1b (100003675) | 0 | 0.0 | |    fetub (569489) | 0 | 0.0 | |    zgc:174153 (567623) | 0 | 0.0 | |    im:7148400 (100003770) | 0 | 0.0 | |    abca12 (558335) | 0 | 0.0 | |    amdhd2 (402981) | 0 | 0.0 | |    sb:cb252 (321177) | 0 | 0.0 | |    LOC100537515 (100537515) | 0 | 0.0 | |    gsnb (336623) | 0 | 0.0 | |    LOC100538072 (100538072) | 0 | 0.0 | |    hbbe2 (405772) | 0 | 0.0 | |    zgc:92380 (445086) | 0 | 0.0 | |    rgs2 (436935) | 0 | 0.0 | |    zgc:158494 (386720) | 0 | 0.0 | |    LOC792966 (792966) | 0 | 0.0 | |    hspb9 (100137108) | 0 | 0.0 | |    slc25a25a (406541) | 0 | 0.0 | |    hao1 (402827) | 0 | 0.0 | |    rab14 (373104) | 0 | 0.0 | |    zgc:158614 (791218) | 0 | 0.0 | |    si:dkeyp-75b4.9 (100321606) | 0 | 0.0 | |    apobb (321166) | 0 | 0.0 | |    hs3st1l2 (562344) | 0 | 0.0 | |    zgc:173443 (100126130) | 0 | 0.0 | |    msgn1 (360135) | 0 | 0.0 | |    si:dkey-239j18.3 (100001785) | 0 | 0.0 | |    ponzr4 (557748) | 0 | 0.0 | |    lygl1 (436979) | 0 | 0.0 | |    si:dkeyp-75h12.2 (559449) | 0 | 0.0 | | | Term | Count | CI | | --- | --- | --- | |    obesity | 505 | 8.983 | |    type 2 diabetes | 471 | 8.8826 | |    hypertension | 469 | 8.8765 | |    osteoporosis | 51 | 5.7004 | |

**100** of the **1339** PubMed papers involved are listed below (sorted by relevance):

| PubMed ID | Title |
| --- | --- |
| 18029445 | Macrophage-derived apolipoprotein E ameliorates dyslipidemia and atherosclerosis in obese apolipoprotein E-deficient mice.Previous studies have demonstrated that macrophage-derived apolipoprotein E (apoE) reduces atherosclerotic lesion formation in lean apoE-deficient ((-/-)) mice. apoE has also been demonstrated to play a role in adipocyte differentiation and lipid accumulation. Because the prevalence of obesity has grown to epidemic proportions, we sought to determine whether macrophage-derived apoE could impact atherosclerotic lesion formation or adipose tissue expansion and inflammation in obese apoE(-/-) mice. To this end, we transplanted obese leptin-deficient (ob/ob) apoE(-/-) mice with bone marrow from either ob/ob;apoE(-/-) or ob/ob;apoE(+/+) donors. There were no differences in body weight, total body adipose tissue, or visceral fat pad mass between recipient groups. The presence of macrophage-apoE had no impact on adipose tissue macrophage content or inflammatory cytokine expression. Recipients of apoE(+/+) marrow demonstrated 3.7-fold lower plasma cholesterol (P < 0.001) and 1.7-fold lower plasma triglyceride levels (P < 0.01) by 12 wk after transplantation even though apoE was present in plasma at concentrations <10% of wild-type levels. The reduced plasma lipids reflected a dramatic decrease in very low density lipoprotein and a mild increase in high-density lipoprotein levels. Atherosclerotic lesion area was >10-fold lower in recipients of ob/ob;apoE(+/+) marrow (P < 0.005). Similar results were seen in leptin receptor-deficient (db/db) apoE(-/-) mice. Finally, when bone marrow transplantation was performed in 4-mo-old ob/ob;apoE(-/-) and db/db;apoE(-/-) mice with preexisting lesions, recipients of apoE(+/+) marrow had a 2.8-fold lower lesion area than controls (P = 0.0002). These results demonstrate that macrophage-derived apoE does not impact adipose tissue expansion or inflammatory status; however, even very low levels of macrophage-derived apoE are capable of reducing plasma lipids and atherosclerotic lesion area in obese mice. |
| 18443202 | Association analysis in african americans of European-derived type 2 diabetes single nucleotide polymorphisms from whole-genome association studies.OBJECTIVE: Several whole-genome association studies have reported identification of type 2 diabetes susceptibility genes in various European-derived study populations. Little investigation of these loci has been reported in other ethnic groups, specifically African Americans. Striking differences exist between these populations, suggesting they may not share identical genetic risk factors. Our objective was to examine the influence of type 2 diabetes genes identified in whole-genome association studies in a large African American case-control population. RESEARCH DESIGN AND METHODS: Single nucleotide polymorphisms (SNPs) in 12 loci (e.g., TCF7L2, IDE/KIF11/HHEX, SLC30A8, CDKAL1, PKN2, IGF2BP2, FLJ39370, and EXT2/ALX4) associated with type 2 diabetes in European-derived populations were genotyped in 993 African American type 2 diabetic and 1,054 African American control subjects. Additionally, 68 ancestry-informative markers were genotyped to account for the impact of admixture on association results. RESULTS: Little evidence of association was observed between SNPs, with the exception of those in TCF7L2, and type 2 diabetes in African Americans. One TCF7L2 SNP (rs7903146) showed compelling evidence of association with type 2 diabetes (admixture-adjusted additive P [P(a)] = 1.59 x 10(-6)). Only the intragenic SNP on 11p12 (rs9300039, dominant P [P(d)] = 0.029) was also associated with type 2 diabetes after admixture adjustments. Interestingly, four of the SNPs are monomorphic in the Yoruba population of the HAPMAP project, with only the risk allele from the populations of European descent present. CONCLUSIONS: Results suggest that these variants do not significantly contribute to interindividual susceptibility to type 2 diabetes in African Americans. Consequently, genes contributing to type 2 diabetes in African Americans may, in part, be different from those in European-derived study populations. High frequency of risk alleles in several of these genes may, however, contribute to the increased prevalence of type 2 diabetes in African Americans. |
| 20950077 | Retinol binding protein 4 (RBP4) is primarily associated with adipose tissue mass in children.OBJECTIVE: Retinol binding protein 4 (RBP4) is a novel adipocytokine that may link obesity and insulin resistance. We aimed to discriminate between primary and secondary associations of RBP4 with obesity and related disease. DESIGN: We applied clinical and experimental approaches to investigate the association of RBP4 levels with normal development, obesity, metabolic and cardiovascular parameters in 68 lean and 61 obese children. RESULTS: RBP4 significantly increased with age and pubertal development in healthy lean children. Obese children had significantly higher RBP4 levels compared with lean controls (30.5+/-1.4 vs. 26.3+/-1.1 mg/L, P<0.05) and there was a clear association with BMI independent of age (r=0.33, P<0.0001). RBP4 levels correlated significantly with parameters of lipid and glucose metabolism, as well as cardiovascular parameters in univariate analyses. Multiple regression analyses confirmed the strong association of RBP4 with BMI z-score and age, while the association with most metabolic and cardiovascular parameters was abolished. To assess whether the association of RBP4 with obesity may be attributable to adipogenesis, we evaluated RBP4 expression and secretion during adipocyte differentiation using the human SGBS cell line. In preadipocytes, RBP4 mRNA expression was nearly undetectable but increased during differentiation up to approximately 1600-fold (P<0.05). Likewise, RBP4 secretion was restricted to mature adipocytes, further indicating that RBP4 is strongly related to differentiation of adipocytes. CONCLUSION: RBP4 is a marker of adipose tissue mass and obesity already evident in children. The association of RBP4 with metabolic and cardiovascular sequelae of obesity appears to be secondary to the underlying relationship wtih body fat. |
| 15287858 | Nitric oxide synthase 1 is partly compensating for nitric oxide synthase 3 deficiency in nitric oxide synthase 3 knock-out mice and is elevated in murine and human cirrhosis.BACKGROUND: The role of endothelial nitric oxide synthase 3 (NOS-3) in the hyperdynamic circulation associated with cirrhosis is established but not that of the neuronal (NOS-1) isoform. We therefore investigated aortic NOS-1 levels in NOS-3 knock-out (KO) and wildtype (WT) mice and in hepatic arteries of patients. METHODS: Mice rendered cirrhotic by bile duct ligation (BDL) were compared with sham-operated controls. Hepatic arteries of cirrhotic patients were collected during liver transplantation; donor vessels served as controls. mRNA levels were quantified by real-time PCR, protein levels by Western blotting and NO production by Nomega-nitro-L-arginine methyl ester inhibitable arginine-citrulline assay. RESULTS: Aortae of NOS-3 KO mice exhibited higher NOS-1mRNA (5.6-fold, P < 0.004) and protein levels (8.8-fold) compared with WT. NO production in aortae of NOS-3 KO mice was 52% compared with WT (P = 0.002). BDL increased NOS-1 mRNA (2.4-fold, P = 0.01) and protein (7.1-fold) levels in aortae of WT, but no further in the NOS-3 KO mice. Hepatic artery NOS-1 mRNA levels in cirrhotic patients were markedly increased compared with controls (24.5-fold, P = 0.0007). CONCLUSIONS: Increased NOS-1 mRNA and protein levels and partially maintained in vitro NO-production in aortae of NOS-3 KO mice suggest that NOS-1 may partially compensate for NOS-3 deficiency. BDL-induced increase in aortic NOS-1 mRNA and protein levels hint that not only NOS-3, but also NOS-1 may be involved in the regulation of systemic hyperdynamic circulation and portal hypertension. Upregulation of NOS-1 mRNA levels in hepatic arteries of portal hypertensive patients suggests possible clinical significance for these experimental findings. |
| 17709878 | PCK1 and PCK2 as candidate diabetes and obesity genes.The PCK1 gene (Pck1 in rodents) encodes the cytosolic isozyme of phosphoenolpyruvate carboxykinase (PEPCK-C), which is well-known for its function as a gluconeogenic enzyme in the liver and kidney. Mouse studies involving whole body and tissue-specific Pck1 knockouts as well as tissue-specific over-expression of PEPCK-C have resulted in type 2 diabetes as well as several surprising phenotypes including obesity, lipodystrophy, fatty liver, and death. These phenotypes arise from perturbations not only in gluconeogenesis but in two additional metabolic functions of PEPCK-C: (1) cataplerosis which maintains metabolic flux through the Krebs cycle by removing excess oxaloacetate, and (2) glyceroneogenesis which produces glycerol-3-phosphate as a precursor for fatty acid esterification into triglycerides. PEPCK-C catalyzes the conversion of oxaloacetate + GTP to phosphoenolpyruvate + GDP + CO2. It is in part the tissue-specificity of this simple reaction that results in the variety of phenotypes listed above. Briefly: (1) A 7-fold over-expression of PEPCK-C in the livers of mice causes excessive glucose production. (2) Mice with a whole-body knockout of Pck1 die within 2-3 days of birth, not from hypoglycemia, but probably because the Krebs cycle slows to approximately 10% of normal in the absence of cataplerosis. (3) Mice with a liver-specific knockout have an inability to remove oxaloacetate from the Krebs cycle, which leads to a fatty liver following a fast. (4) An adipose-specific knockout of Pck1 results in a fraction of the mice developing lipodystrophy due to lost glyceroneogenesis and a consequent decrease in fatty acid re-esterification. (5) Finally, disregulated over-expression of PEPCK-C in adipose tissue increases fatty acid re-esterification leading to obesity. These varied experimental phenotypes in mice have led us to postulate that abnormal production of PEPCK isozymes encoded by two PEPCK genes, PCK1 and PCK2, in humans could have similar consequences (Beale, E. G. et al. (2004). Trends in Endocrinology and Metabolism, 15, 129-135). The purpose of this review is to further explore these possibilities. |
| 18694974 | Predicting type 2 diabetes based on polymorphisms from genome-wide association studies: a population-based study.OBJECTIVE: Prediction of type 2 diabetes based on genetic testing might improve identification of high-risk subjects. Genome-wide association (GWA) studies identified multiple new genetic variants that associate with type 2 diabetes. The predictive value of genetic testing for prediction of type 2 diabetes in the general population is unclear. RESEARCH DESIGN AND METHODS: We investigated 18 polymorphisms from recent GWA studies on type 2 diabetes in the Rotterdam Study, a prospective, population-based study among homogeneous Caucasian individuals of 55 years and older (genotyped subjects, n = 6,544; prevalent cases, n = 686; incident cases during follow-up, n = 601; mean follow-up 10.6 years). The predictive value of these polymorphisms was examined alone and in addition to clinical characteristics using logistic and Cox regression analyses. The discriminative accuracy of the prediction models was assessed by the area under the receiver operating characteristic curves (AUCs). RESULTS: Of the 18 polymorphisms, the ADAMTS9, CDKAL1, CDKN2A/B-rs1412829, FTO, IGF2BP2, JAZF1, SLC30A8, TCF7L2, and WFS1 variants were associated with type 2 diabetes risk in our population. The AUC was 0.60 (95% CI 0.57-0.63) for prediction based on the genetic polymorphisms; 0.66 (0.63-0.68) for age, sex, and BMI; and 0.68 (0.66-0.71) for the genetic polymorphisms and clinical characteristics combined. CONCLUSIONS: We showed that 9 of 18 well-established genetic risk variants were associated with type 2 diabetes in a population-based study. Combining genetic variants has low predictive value for future type 2 diabetes at a population-based level. The genetic polymorphisms only marginally improved the prediction of type 2 diabetes beyond clinical characteristics. |
| 19033397 | Replication study of candidate genes associated with type 2 diabetes based on genome-wide screening.OBJECTIVE: The present study was conducted to confirm possible associations between candidate genes from genome-wide association studies and type 2 diabetes in Japanese diabetic patients and a community-based general population. A total of 11 previously reported single-nucleotide polymorphisms (SNPs) from the TCF7L2, CDKAL1, HHEX, IGF2BP2, CDKN2A/B, SLC30A8, and KCNJ11 genes were analyzed. RESEARCH DESIGN AND METHODS: Candidate SNPs were genotyped in 506 type 2 diabetic patients and 402 control subjects and meta-analyzed with six previous association studies in Japanese patients. Associations with fasting plasma insulin levels were investigated in a general population sample (n = 1,963, 61 +/- 13 years). RESULTS: In our case-control subjects, susceptibility to type 2 diabetes was replicated in TCF7L2 (rs12255372), CDKAL1 (rs7756992, rs7754840), HHEX (rs7923837), IGF2BP2 (rs4402960 and rs1470579), CDKN2A/B (rs10811661), and SLC30A8 (rs13266634). In addition to these polymorphisms, meta-analysis confirmed the association of type 2 diabetes susceptibility with KCNJ11 rs5219, TCF7L2 rs7903146, and HHEX rs1111875. The TCF7L2 rs12255372 polymorphism showed the highest odds ratio (OR) for type 2 diabetes (OR 1.714 [1.298-2.263]). Odds ratio of other polymorphisms ranged from 1.13 to 1.41. The risk allele of CDKAL1 rs7756992 was significantly associated with lower insulin levels in type 2 diabetic patients after adjustment for other confounding factors. CONCLUSIONS: Type 2 diabetes susceptibility of seven candidate genes was confirmed in Japanese. Conservation of susceptible loci for type 2 diabetes was independent of ethnic background. |
| 22253430 | Quantitative measurement of full-length and C-terminal proteolyzed RBP4 in serum of normal and insulin-resistant humans using a novel mass spectrometry immunoassay.Serum retinol-binding protein 4 (RBP4) levels are increased in insulin-resistant humans and correlate with severity of insulin resistance in metabolic syndrome. Quantitative Western blotting (qWestern) has been the most accurate method for serum RBP4 measurements, but qWestern is technically complex and labor intensive. The lack of a reliable, high-throughput method for RBP4 measurements has resulted in variability in findings in insulin-resistant humans. Many commonly used ELISAs have limited dynamic range. Neither the current ELISAs nor qWestern distinguish among full-length and carboxyl terminus proteolyzed forms of circulating RBP4 that are altered in different medical conditions. Here, we report the development of a novel quantitative mass spectrometry immunoaffinity assay (qMSIA) to measure full-length and proteolyzed forms of RBP4. qMSIA and qWestern of RBP4 were performed in identical serum aliquots from insulin-sensitive/normoglycemic or insulin-resistant humans with impaired glucose tolerance or type 2 diabetes. Total RBP4 qMSIA measurements were highly similar to qWestern and correlated equally well with clinical severity of insulin resistance (assessed by clamp glucose disposal rate, r = -0.74), hemoglobin A1c (r = 0.63), triglyceride/high-density lipoprotein (r = 0.55), waist/hip (r = 0.61), and systolic blood pressure (r = 0.53, all P < 0.001). Proteolyzed forms of RBP4 accounted for up to 50% of total RBP4 in insulin-resistant subjects, and des(Leu)-RBP4 (cleavage of last leucine) correlated highly with insulin resistance (assessed by glucose disposal rate, r = -0.69). In multiple regression analysis, insulin resistance but not glomerular filtration rate was the strongest, independent predictor of serum RBP4 levels. Thus, qMSIA provides a novel tool for accurately measuring serum RBP4 levels as a biomarker for severity of insulin resistance and risk for type 2 diabetes and metabolic syndrome. |
| 10559023 | Hypertension and endothelial dysfunction in apolipoprotein E knockout mice.Mice lacking ApoE (Apoe(-/-)) develop initially hypercholesterolemia and lastly atherosclerosis. This study examined hemodynamics and endothelial function in 6-week-old Apoe(-/-) mice with hypercholesterolemia only, 7.5-months-old Apoe(-/-) mice with both hypercholesterolemia and atherosclerosis, and age matched controls. One day after implantation of catheters into the carotid artery, arterial pressure was measured in conscious, unrestrained mice. Compared with the respective controls, there was a significant increase in arterial pressure and the ratio of left ventricular weight to body weight in 7.5-month-old Apoe(-/-) mice but not in 6-week-old Apoe(-/-) mice. Histopathological analysis demonstrated significant renal artery disease in the form of extensive atheromatous plaques only in 7.5-month-old Apoe(-/-) mice, whereas no atherosclerotic lesions were found in 6-week-old Apoe(-/-) mice. For evaluation of endothelial function, a laser Doppler perfusion imager with a computer-controlled optical scanner was used to measure cutaneous blood perfusion on the dorsal side of one hind paw before and after topical application of mustard oil, which is known to induce nitric oxide-mediated vasodilation. The mustard oil treatment elicited a substantial increase in blood perfusion (P<0.01), which was similar between 6-week-old Apoe(-/-) mice and controls but significantly blunted in 7.5-month-old Apoe(-/-) mice versus control mice, suggesting nitric oxide-mediated vasodilation is diminished in 7.5-month-old Apoe(-/-) mice but not in 6-week-old Apoe(-/-) mice. In contrast, the increase in blood perfusion induced by topical administration of cilostazol, which induces vasodilation via cyclic adenosine monophosphate, was not different between 7.5-month-old Apoe(-/-) mice and controls. Thus hypertension and endothelial dysfunction observed in 7.5-month-old Apoe(-/-) mice may be due mainly to atherosclerosis. |
| 19190263 | Retinol-binding protein 4 is associated with impaired glucose tolerance but not with whole body or hepatic insulin resistance in Mexican Americans.Retinol-binding protein-4 (RBP4), a novel protein secreted mainly by adipose tissue, has been associated with insulin resistance in obese subjects and in individuals with type 2 diabetes mellitus (T2DM). We examined the relationship between plasma RBP4 levels, expression of RBP4 in skeletal muscle and adipose tissue, and insulin sensitivity in Mexican Americans with varying degrees of obesity and glucose tolerance. Seventy-two subjects [16 lean normal-glucose-tolerant (NGT), 17 obese NGT, and 39 subjects with impaired fasting glucose/impaired glucose tolerance/T2DM] received an oral glucose tolerance test (OGTT) and euglycemic-hyperinsulinemic clamp. Insulin secretion was measured as insulinogenic index during OGTT. In a subset of subjects, hepatic glucose production was measured by 3-[3H]glucose infusion, biopsies of the vastus lateralis muscle and subcutaneous adipose tissue were obtained under basal conditions, and quantitative RT-PCR was performed to measure the RBP4 mRNA gene expression. Plasma RBP4 was significantly elevated in impaired glucose tolerance/T2DM compared with NGT lean or obese subjects. Plasma RBP4 levels correlated with 2-h glucose, triglycerides, and hemoglobin A1c. There was no association between RBP4 levels and whole body insulin sensitivity measured with either the euglycemic insulin clamp or OGTT, basal hepatic glucose production rates, and the hepatic insulin resistance index. There was no correlation between plasma RBP4 levels and indexes of insulin secretion. RBP4 mRNA expression in skeletal muscle was similar in lean NGT subjects, obese NGT subjects, and T2DM subjects. There was no difference in RBP4 mRNA expression in adipose tissue between lean and obese NGT subjects or between NGT and T2DM individuals. Plasma RBP4 levels are elevated in T2DM and associated with impaired glucose tolerance, but not associated with obesity or insulin resistance or impaired insulin secretion in Mexican Americans. |
| 22384526 | Investigation of ABCA1 C69T polymorphism in patients with type 2 diabetes mellitus.INTRODUCTION: Non insulin dependent diabetes mellitus is the most common type of diabetes. Genetic factors, lipid profiles, hypertension are potential risk factors for diabetes mellitus. Adenosine binding cassette transporter proteins 1 (ABCA1) plays a role in cholesterol metabolism, especially high density lipoprotein (HDL-cholesterol). There are multiple mechanisms by which HDL-cholesterol can be atheroprotective, it is clear that the relative activity of ABCA1 plays a major role. We aimed to investigate association of ABCA1 C69T gene polymorphism with lipid levels in Turkish type 2 diabetic patients. MATERIALS AND METHODS: After isolation of DNA by ethanol precipitation we determined ABCA1 gene polymorphism by using polimerase chain reaction--restriction fragment lenght polymorphism (PCR-RFLP) method in 107 type 2 diabetic patients and 50 healthy controls. RESULTS: We have observed that the frequency of TT genotype is significantly higher in healthy controls compared to patients (14% vs. 3%; P = 0.008). Also frequency of T allele was higher in controls than in patients (34% vs. 21%; P = 0.020; OR (95% CI) = 0.52 (0.30-0.88)). There was no association of lipid levels and ABCA1 C69T polymorphism subgroups. CONCLUSION: We have found significantly higher frequency of both T allele and genotype in control group when compared to patients that made us think that T allele may be a protective factor against diabetes mellitus. But, we could not find a relationship between genotypes and lipid concentrations in our two groups. Larger studies will help us to understand the relationship between ABCA1 C69T genotype and lipid parameters in diabetes mellitus. |
| 18397979 | Retinol-binding protein 4 and its relation to insulin resistance in obese children before and after weight loss.CONTEXT: There are limited and controversial data concerning the relationships between retinol-binding protein 4 (RBP4), weight status, and insulin resistance in obese humans and especially in children. OBJECTIVE: Our objective was to study the longitudinal relationships among RBP4, insulin resistance and weight status in obese children. DESIGN, SETTING, AND PATIENTS: We conducted a 1-yr longitudinal follow-up study in a primary-care setting with 43 obese children (median age 10.8 yr) and 19 lean children of same the age and gender. INTERVENTION: Our outpatient 1-yr intervention program was based on exercise, behavior, and nutrition therapy. MAIN OUTCOMES MEASURES: Changes of weight status (body mass index sd score), RBP4, molar RBP4/serum retinol (SR) ratio, insulin resistance index homeostasis model assessment (HOMA), and quantitative insulin sensitivity check index (QUICKI). RESULTS: Obese children had significantly (P < 0.01) higher RBP4 concentrations and a higher RBP4/SR ratio compared with lean children. In multiple linear regression analyses adjusted to age, gender, and pubertal stage, RBP4 was significantly correlated to insulin and body mass index. Pubertal children demonstrated significantly decreased QUICKI and significantly increased HOMA index, insulin, and RBP4 concentrations compared with prepubertal children. Changes of RBP4 correlated significantly to changes of insulin (r = 0.29), HOMA index (r = 0.29), QUICKI (r = 0.22), and weight status (r = 0.31). Substantial weight loss in 25 children led to a significant (P < 0.001) decrease of RBP4, RBP4/SR, blood pressure, triglycerides, insulin, and HOMA index and an increase in QUICKI in contrast to the 18 children without substantial weight loss. CONCLUSION: RBP4 levels were related to weight status and insulin resistance in both cross-sectional and longitudinal analyses, suggesting a relationship between RBP4, obesity, and insulin resistance in children. |
| 20134411 | Lack of association between PCK1 polymorphisms and obesity, physical activity, and fitness in European Youth Heart Study (EYHS).Phosphoenolpyruvate carboxykinase-1 (PCK1) is the rate-limiting enzyme in the hepatic gluconeogenic pathway. Studies have shown that overexpression of Pck1 in mice results in obesity-related traits and higher levels of physical activity (PA). Therefore, our aims were to investigate whether common genetic variation in the PCK1 gene influences obesity-related traits, PA, and fitness, and to examine whether PA and fitness attenuate the influence of the PCK1 polymorphisms on obesity in children. Analyses were undertaken on data from Danish and Estonian children (958 boys and 1,104 girls) from the European Youth Heart Study (EYHS), a school-based, cross-sectional study of children (mean +/- s.d. age: 9.6 +/- 0.4 years) and adolescents (15.5 +/- 0.5 years). We genotyped eight polymorphisms that captured the common genetic variations in the PCK1 gene. The association between the PCK1 polymorphisms and BMI, waist circumference (WC), sum of four skinfolds, PA, and fitness was tested using an additive model adjusted for age, age-group, gender, maturity, and country. Interactions were tested by including interaction terms in the model. None of the polymorphisms were significantly associated with BMI, WC, sum of four skinfolds, PA, and fitness, and also with the risk of being overweight or obese (P > 0.05). The interactions between the polymorphisms and age-group, gender, PA, and fitness were not statistically significant. This is the first study to comprehensively examine the association of PCK1 polymorphisms with obesity, PA, and fitness. Despite strong evidence from animal studies, our study in the EYHS cohort failed to identify an association of PCK1 polymorphisms with obesity, PA, and fitness. |
| 21596930 | Serum angiopoietin-like 4 protein levels and expression in adipose tissue are inversely correlated with obesity in monozygotic twins.Animal studies have suggested that angiopoietin-like 4 (Angptl4) regulates adiposity through central and peripheral mechanisms. The aim of this study was to investigate whether serum concentration and adipose tissue expression of Angptl4 are associated with obesity-related parameters in humans. Altogether, 75 dizygotic (DZ) and 46 monozygotic (MZ) twin pairs were studied, from the FinnTwin12 and FinnTwin16 cohorts. Among the MZ pairs, 21 were discordant for body mass index (BMI) (intra-pair BMI-difference >2.5 kg/m(2), age 23-33 years). Serum Angptl4 (s-Angptl4) levels were measured by ELISA, and adipose tissue gene expression was analyzed by genome-wide transcript profiling. In MZ twin pairs discordant for BMI, s-Angptl4 and adipose tissue ANGPTL4 mRNA (at-ANGPTL4) levels were significantly decreased (P = 0.04 and P = 0.03, respectively) in obese twins as compared with their nonobese cotwins. In all twins, intra-pair differences in s-Angptl4 levels were inversely correlated with intra-pair differences in BMI (r = -0.27, P = 0.003). In individual MZ twins, at-ANGPTL4 expression was inversely correlated with BMI (r = -0.44, P = 0.001) and positively correlated with at-LIPE (r = 0.24, P = 0.01) and at-ABHD5 (r = 0.41, P = 0.005) expression. Our results demonstrated that variation in Angptl4 concentration was only modestly accounted for by genetic factors and suggest a role for Angptl4 in acquired obesity in humans. |
| 18973209 | Elevated serum retinol-binding protein 4 concentrations are associated with renal dysfunction and uric acid in type 2 diabetic patients.BACKGROUND: While some studies have reported that retinol-binding protein 4 (RBP4) might induce insulin resistance, other studies have demonstrated that the presence of albuminuria in diabetic patients and increased uric acid are related to insulin resistance. Therefore, this study attempted to further investigate the relationship among serum RBP4, serum uric acid, and the severity of albuminuria in diabetic patients. METHODS: A total of 95 type 2 diabetic patients and 16 healthy subjects participated in this study. Diabetic patients were classified into normoalbuminuria, microalbuminuria and macroalbuminuria groups according to their urine albumin-to-creatinine ratio (ACR). Serum RBP4 was measured by an enzyme-linked immunosorbent assay (ELISA). RESULTS: Serum RBP4 was significantly elevated in type 2 diabetic patients with normoalbuminuria (43.4 +/- 14.9 microg/mL), microalbuminuria (57.3 +/- 24.2 microg/mL) and macroalbuminuria (64.7 +/- 27.6 microg/mL) as compared with control patients (32.6 +/- 10.0 microg/mL). Serum RBP4 was also significantly elevated in type 2 diabetic patients with microalbuminuria or macroalbuminuria as compared with the normoalbuminuric group. Serum RBP4 in diabetic subjects was positively correlated with triglycerides, uric acid and ACR, and negatively correlated with low-density lipoprotein cholesterol and estimated glomerular filtration rate (eGFR; with age and gender adjustment in each parameter). Multiple stepwise linear regression analysis showed that uric acid and eGFR remained significantly associated with serum RBP4. CONCLUSIONS: Both eGFR and uric acid are significant determinants of serum RBP4, suggesting that the impaired renal clearance of early diabetic nephropathy affects RBP4 and indirectly supporting the hypothesized link among metabolic syndrome, uric acid and insulin resistance. |
| 19003725 | Visceral obesity is associated with the metabolic syndrome and elevated plasma retinol binding protein-4 level in obstructive sleep apnea syndrome.Obstructive sleep apnea syndrome (OSAS) is related to the increased prevalence of cardiovascular disease and metabolic syndrome (MS). A novel adipokine, retinol binding protein-4 (RBP4), was reported to be associated with insulin resistance and the prevalence of type 2 diabetes. To examine whether plasma RBP4 is associated with insulin resistance and MS development in OSAS, we measured plasma RBP4 levels in 181 Japanese men (24 healthy controls and 40 mild, 64 moderate, and 53 severe OSAS) of whom 26 had mild glucose intolerance with HbA1c < or = 6.0%. After a full polysomnography, blood was collected between 06:00 and 07:00 AM. Plasma RBP4 levels in moderate/severe OSAS patients were higher than in control subjects. Plasma RBP4 was not correlated with apnea variables, HOMA-IR, or blood pressure. However, it was positively correlated with visceral fat areas and plasma triglyceride levels. The prevalence of MS was higher in severe OSAS patients than in mild/moderate OSAS and control subjects. Plasma RBP4 was higher in OSAS patients with MS than in those without MS. This study indicates that plasma RBP4 is associated with dyslipidemia, but not with insulin resistance, glucose intolerance, or hypertension in patients with OSAS. Visceral obesity may play key roles in increasing the plasma RBP4 level and MS development in OSAS. |
| 22614118 | The transcription levels of ABCA1, ABCG1 and SR-BI are negatively associated with plasma CRP in Chinese populations with various risk factors for atherosclerosis.ATP binding cassette transporters (ABCA1, ABCG1) and scavenger receptor class B type I (SR-BI) are the three most important cellular cholesterol transporters that may prevent atherogenesis. The aim of this study was to investigate whether they were altered in Chinese populations with various risk factors for atherosclerosis and their potential associations with C-reactive protein (CRP). Healthy female controls (n = 30) and populations with various risk factors for atherosclerosis, such as type 2 diabetes (n = 17), hypertension (n = 12), overweight/obesity (n = 10), incipient nephropathy (n = 10), postmenopausal women (n = 9), male (n = 19), ageing male (n = 22), or smoking (n = 16), were recruited. ABCA1, ABCG1 and SR-BI mRNA levels in peripheral monocytes was determined. ABCG1 was decreased in all the risk populations except ageing. ABCA1 was decreased in all the risk populations except diabetes and male. SR-BI was decreased in those with overweight/obesity and incipient nephropathy. Circulating CRP was increased almost in all the risk populations except in males. The levels of ABCA1, ABCG1 and SR-BI were reduced in those with subclinically high CRP, and negatively associated with CRP level. These data indicates that ABCA1, ABCG1, and SR-BI are reduced in various populations under subclinically inflammatory conditions, which may potentially lead to impairing reverse cholesterol transport and developing atherosclerosis. |
| 23460908 | Interleukin-1beta downregulates RBP4 secretion in human adipocytes.AIMS/HYPOTHESIS: The excessive accumulation of adipose tissue in the obese state is linked to an altered secretion profile of adipocytes, chronic low-grade inflammation and metabolic complications. RBP4 has been implicated in these alterations, especially insulin resistance. The aim of the present study was to determine if a local inflammatory micro-environment in adipose tissue regulates RBP4 expression and secretion. METHODS: Human SGBS and primary adipocytes cultured with conditioned media from human THP-1 macrophages were used as an in vitro model for adipose inflammation. Adipocytes were exposed to recombinant TNF-alpha, IL-1beta, IL-6 or IL-8. In addition, coexpression of IL-1beta and RBP4 was measured in adipose tissue samples from 18 healthy females. RBP4 expression was studied by quantitative PCR and ELISA. RESULTS: RBP4 mRNA expression and secretion was significantly reduced upon incubation with macrophage-conditioned media in SGBS adipocytes and human primary adipocytes. Out of several factors studied we identified IL-1beta as a new factor regulating RBP4. IL-1beta significantly downregulated RBP4 mRNA and secretion in a time- and dose-dependent manner. IL-1beta mediated its inhibitory effects on RBP4 expression via IL-1 receptor and NF-kappaB, as incubation with the IL-1 receptor blocking antibody and the NF-kappaB inhibitors CAPE and SC-514 reversed its effect. Most interestingly, RBP4 mRNA was negatively correlated with IL-1beta mRNA in subcutaneous adipose tissue. CONCLUSIONS: Adipose tissue inflammation as found in the obese state might lead to a downregulation in local RBP4 levels. IL-1beta was identified as a major factor contributing to the decrease in RBP4. The increase in circulating RBP4 that often precedes the development of systemic insulin resistance is most likely unrelated to inflammatory processes in adipose tissue. |
| 24339435 | SULF2 strongly prediposes to fasting and postprandial triglycerides in patients with obesity and type 2 diabetes mellitus.Objective Hepatic overexpression of sulfatase-2 (SULF2), a heparan sulfate remodelling enzyme, strongly contributes to high triglyceride (TG) levels in obese, type 2 diabetic (T2DM) db/db mice. Nevertheless, data in humans are lacking. Here we sought to investigate the association of human hepatic SULF2 expression and SULF2 gene variants with TG metabolism in patients with obesity and/or T2DM. Design and Methods Liver biopsies from 121 obese subjects were analyzed for relations between hepatic SULF2 mRNA levels and plasma TG. Associations between seven SULF2 tagSNPs and TG levels were assessed in 210 obese T2DM subjects with dyslipidemia. Replication of positive findings was performed in 1316 independent obese T2DM patients. Postprandial TRL clearance was evaluated in 29 obese T2DM subjects stratified by SULF2 genotype. Results Liver SULF2 expression was significantly associated with fasting plasma TG (r = 0.271; p=0.003) in obese subjects. The SULF2 rs2281279(A>G) SNP was reproducibly associated with lower fasting plasma TG levels in obese T2DM subjects (p<0.05). Carriership of the minor G allele was associated with lower levels of postprandial plasma TG (P<0.05) and retinyl esters (RE) levels (P<0.001). Conclusions These findings implicate SULF2 as potential therapeutic target in the atherogenic dyslipidemia of obesity and T2DM. |
| 17299074 | Retinol-binding protein 4 is associated with insulin resistance and body fat distribution in nonobese subjects without type 2 diabetes.BACKGROUND: Adipose tissue is responsible for releasing various adipokines that have been related to insulin resistance. Understanding the relationship of these adipokines to insulin resistance may foster the development of new treatments for diabetes. OBJECTIVES: The primary objective of this study was to determine whether an association between retinol-binding protein 4 (RBP4) and insulin resistance exists in nonobese individuals without a family history or diagnosis of diabetes. The secondary objective was to determine by a dual energy x-ray absorptiometry scan which adipose tissue depot most closely relates to RBP4 levels. DESIGN: Cross-sectional analysis of 92 study participants ranging in age from 20 to 83 yr was performed. The range of body mass index (BMI) was from 18 to 30 kg/m(2). Exclusion criteria were a BMI greater than 30 kg/m(2), family history of diabetes, or a diagnosis of diabetes. Insulin sensitivity was determined by a hyperinsulinemic euglycemic clamp. Body fat was measured by dual energy x-ray absorptiometry scan. RESULTS: RBP4 values were lower in females (35.8 +/- 1.7 microg/ml) compared with males (39.9 +/- 1.4 microg/ml; P = 0.06). RBP4 levels were found to correlate negatively with insulin sensitivity (r = -0.32; P = 0.002) and positively with age (r = 0.38; P < 0.001). RBP4 levels did not correlate with BMI (r = -0.13; P = 0.22), trunk fat (r = 0.16; P = 0.22), or percent body fat (r = 0.07; P = 0.65). However, RBP4 levels did correlate with percent trunk fat (r = 0.36; P = 0.001). CONCLUSION: These findings indicate a relationship between RBP4, insulin sensitivity, and percent trunk fat in individuals who may not have features of insulin resistance. |
| 9486205 | Hypoxia induces type II NOS gene expression in pulmonary artery endothelial cells via HIF-1.Type II nitric oxide synthase (NOS) is upregulated in the pulmonary vasculature in a chronic hypoxia model of pulmonary hypertension. In situ hybridization analysis demonstrates that type II NOS RNA is increased in the endothelium as well as in the vascular smooth muscle in the lung. The current studies examine the role of hypoxia-inducible factor (HIF)-1 in regulating type II NOS gene expression in response to hypoxia in pulmonary artery endothelial cells. Northern blot analyses demonstrate a two fold increase in HIF-1 alpha but not in HIF-1 beta RNA with hypoxia in vivo and in vitro. Electrophoretic mobility shift assays show the induction of specific DNA binding activity when endothelial cells were subjected to hypoxia. This DNA binding complex was identified as HIF-1 using antibodies directed against HIF-1 alpha and HIF-1 beta. Transient transfection of endothelial cells resulted in a 2.7-fold increase in type II NOS promoter activity in response to hypoxia compared with nonhypoxic controls. Mutation or deletion of the HIF-1 site eliminated the response to hypoxia. These results demonstrate that HIF-1 is essential for the hypoxic regulation of type II NOS gene transcription in pulmonary endothelium. |
| 15118095 | Metabolic syndrome without obesity: Hepatic overexpression of 11beta-hydroxysteroid dehydrogenase type 1 in transgenic mice.In obese humans and rodents there is increased expression of the key glucocorticoid (GC) regenerating enzyme, 11beta-hydroxysteroid dehydrogenase type 1 (11beta-HSD1), in adipose tissue. This increased expression appears to be of pathogenic importance because transgenic mice overexpressing 11beta-HSD1 selectively in adipose tissue exhibit a full metabolic syndrome with visceral obesity, dyslipidemia, insulin-resistant diabetes, and hypertension. In this model, while systemic plasma GC levels are unaltered, GC delivery to the liver via the portal vein is increased. 11beta-HSD1 is most highly expressed in liver where inhibition or deficiency of its activity improves glucose and lipid homeostasis. To determine the potential contribution of elevated intrahepatic GCs alone toward development of insulin-resistant syndromes we generated transgenic mice expressing increased 11beta-HSD1 activity selectively in the liver under transcriptional control of hepatic regulatory sequences derived from the human apoE gene (apoE-HSD1). Transgenic lines with 2- and 5-fold-elevated 11beta-HSD1 activity exhibited mild insulin resistance without altered fat depot mass. ApoE-HSD1 transgenic mice exhibited fatty liver and dyslipidemia with increased hepatic lipid synthesis/flux associated with elevated hepatic LXRalpha and PPARalpha mRNA levels as well as impaired hepatic lipid clearance. Further, apoE-HSD1 transgenic mice have a marked, transgene-dose-associated hypertension paralleled by incrementally increased liver angiotensinogen expression. These data suggest that elevated hepatic expression of 11beta-HSD1 may relate to the pathogenesis of specific fatty liver, insulin-resistant, and hypertensive syndromes without obesity in humans as may occur in, for example, myotonic dystrophy, and possibly, the metabolically obese, normal-weight individual. |
| 16328015 | Association of a microsomal triglyceride transfer protein gene polymorphism with blood pressure in Japanese women.Genetic variants of the microsomal triglyceride transfer protein (MTP) have been associated with the serum concentration of low density lipoprotein-cholesterol, predisposition to coronary heart disease, or longevity. The relation of a -493Gright curved arrow T polymorphism in the promoter of MTP to blood pressure was examined in a population-based study. The subjects (1124 men, 1108 women) were aged 40-79 years and were randomly recruited to a population-based prospective cohort study of aging and age-related diseases in Japan. Blood pressure was measured at least twice with subjects in the sitting position. The serum lipid profile was determined after the subjects had fasted overnight. The -493Gright curved arrow T genotype of MTP was determined with a fluorescence-based allele-specific DNA primer assay system. There was no difference in the serum concentrations of total cholesterol, high density lipoprotein-cholesterol, low density lipoprotein-cholesterol, or triglycerides among MTP genotypes for men or for women. Systolic or diastolic blood pressure was not related to the -493Gright curved arrow T polymorphism in men. For women, however, systolic and diastolic blood pressures were significantly related to MTP genotype, with the T allele of the polymorphism being associated with low blood pressure. The relation between MTP genotype and the prevalence of hypertension was almost significant (P=0.055) for all women. Although MTP genotype was not associated with the prevalence of hypertension in premenopausal women, the relation between these parameters was significant (P=0.040) in postmenopausal women, with the TT genotype protecting against this condition. These results suggest that MTP genotype is a determinant of blood pressure in Japanese women. |
| 19285573 | Complement factors C3a and C5a have distinct hemodynamic effects in the rat.In the rat, C5a infusion mediates well-defined effects including hypotension and neutropenia. Conversely, the comparative effect of C3a in the rat is not yet defined. In the current study, we have investigated C3a receptor (C3aR) activation in the rat, using recombinant human C3a, the C3aR agonist WWGKKYRASKLGLAR, which is a C-terminal analogue of C3a, and a nonpeptide C3aR antagonist SB-290157, as pharmacological tools. In vitro, C3a and WWGKKYRASKLGLAR selectively bound to C3aRs and induced degranulation of C3aR-transfected RBL-2H3 cells. C3a or WWGKKYRASKLGLAR-induced degranulation was dose-dependently antagonized in a surmountable fashion by the nonpeptide C3aR antagonist. Intravenous infusion of C3a and WWGKKYRASKLGLAR to rats induced a rapid, transient and concentration-dependent hypertensive response, which was mediated by C3aR-induced prostanoid release. C3a and WWGKKYRASKLGLAR caused a small drop in circulating neutrophils, but a rise in circulating neutrophils was evident after 90-120 min. In contrast to C3a, C5a infusion resulted in hypotension, and rapid and transient neutropenia. These results demonstrate that C3a and C5a mediate distinct effects on blood pressure and circulating polymorphonuclear leukocytes in the rat. |
| 21228824 | Meta-based evidence for apolipoprotein E epsilon2/epsilon3/epsilon4 polymorphism in association with hypertension among Chinese.Mounting evidence suggests that hypertension is strongly linked to a variety of lipoprotein metabolism abnormalities. Apolipoprotein E gene (ApoE) is one such candidate with its common varepsilon2/varepsilon3/varepsilon4 polymorphism ranking high in hypertension association. To derive more specific information, we pinpoint our research scope in Chinese to test whether this polymorphism is associated with hypertension via a meta-analysis. Random-effects model was performed irrespective of the between study heterogeneity. Data and study quality were assessed in duplicate. Publication bias was evaluated using the fail-safe number. Overall, 12 studies with 14 study groups totalling 1532 hypertensive patients and 2172 controls were identified. Carriers of ApoE varepsilon2 allele had no significant increased risk for hypertension (pooled odds ratio (OR)=1.04; 95% confidence interval (CI): 0.80-1.35; P=0.78), compared with those carrying varepsilon3 allele, whereas those with varepsilon4 allele had a significant increased risk for hypertension (pooled OR=2.03; 95% CI: 1.61-2.55; P<0.00001). After excluding those with other small nationalities, we observed comparison of ApoE varepsilon2 with varepsilon3 allele yielded a pooled OR of 0.99 (95% CI: 0.82-1.19; P=0.89) among Han Chinese, and that of varepsilon4 with varepsilon3 yielded a pooled OR of 1.99 (95% CI: 1.48-2.67; P<0.00001). The fail-safe number at the level of 0.05 supported these significant associations. Taken together, our results expand previous findings and show that ApoE varepsilon4 allele is associated with a twofold increased risk of developing hypertension in Chinese. |
| 21325652 | Cerebrovascular risk factors and preclinical memory decline in healthy APOE epsilon4 homozygotes.OBJECTIVE: To characterize the effects of cerebrovascular (CV) risk factors on preclinical memory decline in cognitively normal individuals at 3 levels of genetic risk for Alzheimer disease (AD) based on APOE genotype. METHODS: We performed longitudinal neuropsychological testing on an APOE epsilon4 enriched cohort, ages 21-97. The long-term memory (LTM) score of the Auditory Verbal Learning Test (AVLT) was the primary outcome measure. Any of 4 CV risk factors (CVany), including hypercholesterolemia (CHOL), prior cigarette use (CIG), diabetes mellitus (DM), and hypertension (HTN), was treated as a dichotomized variable. We estimated the longitudinal effect of age using statistical models that simultaneously modeled the cross-sectional and longitudinal effects of age on AVLT LTM by APOE genotype, CVany, and the interaction between the two. RESULTS: A total of 74 APOE epsilon4 homozygotes (HMZ), 239 epsilon4 heterozygotes (HTZ), and 494 epsilon4 noncarriers were included. APOE epsilon4 carrier status showed a significant quadratic effect with age-related LTM decline in all models as previously reported. CVany was associated with further longitudinal AVLT LTM decline in APOE epsilon4 carriers (p=0.02), but had no effect in noncarriers. When epsilon4 HTZ and HMZ were considered separately, there was a striking effect in HMZ (p<0.001) but not in HTZ. In exploratory analyses, significant deleterious effects were found for CIG (p=0.001), DM (p=0.03), and HTN (p=0.05) in APOE epsilon4 carriers only that remained significant only for CIG after correction for multiple comparisons. CONCLUSION: CV risk factors influence age-related memory decline in APOE epsilon4 HMZ. |
| 21982743 | The arrestin domain-containing 3 protein regulates body mass and energy expenditure.A human genome-wide linkage scan for obesity identified a linkage peak on chromosome 5q13-15. Positional cloning revealed an association of a rare haplotype to high body-mass index (BMI) in males but not females. The risk locus contains a single gene, "arrestin domain-containing 3" (ARRDC3), an uncharacterized alpha-arrestin. Inactivating Arrdc3 in mice led to a striking resistance to obesity, with greater impact on male mice. Mice with decreased ARRDC3 levels were protected from obesity due to increased energy expenditure through increased activity levels and increased thermogenesis of both brown and white adipose tissues. ARRDC3 interacted directly with beta-adrenergic receptors, and loss of ARRDC3 increased the response to beta-adrenergic stimulation in isolated adipose tissue. These results demonstrate that ARRDC3 is a gender-sensitive regulator of obesity and energy expenditure and reveal a surprising diversity for arrestin family protein functions. |
| 22308842 | Retinol binding protein 4 is associated with adiposity-related co-morbidity risk factors in children.OBJECTIVE: In adults, elevated levels of retinol binding protein 4 (RBP4) have been associated with biochemical markers of adiposity-related co-morbidities including insulin resistance, dyslipidemia, hypertension, and abdominal obesity. This study examined the relationship between RBP4 and risk factors for co-morbidities of adiposity in a population of ethnically diverse children in early- to mid-adolescence in the public school system of New York City. MATERIALS/METHODS: We analyzed anthropometric (body mass index, % body fat, waist circumference), metabolic (lipids, glucose), and inflammatory (TNF-alpha, interleukin-6, C-reactive protein, adiponectin) markers for adiposity-related co-morbidities and serum alanine aminotransferase (ALT) in 106 school children (65 males, 41 females) 11-15 years of age (mean +/- SD = 13.0 +/- 0.1 years) who were enrolled in the Reduce Obesity and Diabetes (ROAD) project. Insulin sensitivity was assessed by quantitative insulin sensitivity check index. Insulin secretory capacity was measured as acute insulin response and glucose disposal index. RESULTS: Serum RBP4 was significantly correlated directly with ALT, triglycerides, and triglyceride z-score, and inversely correlated with adiponectin. Correlations with ALT and adiponectin remained significant when corrected for % body fat, age, and gender. There were significant ethnic differences in the relationship of RBP4 to ALT, glucose disposal index and adiponectin. CONCLUSIONS: In early- to mid-adolescents, circulating concentrations of RBP4 are correlated with multiple risk factors for adiposity-related co-morbidities. The observation that many associations persisted when corrected for % body fat, suggests that RBP4 can be viewed as an independent marker of adiposity-related co-morbidity risk in children. |
| 7514568 | Isolation and chromosomal localization of the human endothelial nitric oxide synthase (NOS3) gene.Nitric oxide (NO) is an important intercellular signaling molecule synthesized in diverse human tissues by proteins encoded by a family of NO synthase (NOS) genes. The similarity of sequence and cofactor binding sites has suggested that the NOS genes may also be related to cytochrome P450 reductase, as well as to plant and bacterial oxidoreductases. Endothelial NOS activity is a major determinant of vascular tone and blood pressure, and in several important (and sometimes hereditary) disease states, such as hypertension, diabetes, and atherosclerosis, the endothelial NO signaling system appears to be abnormal. To explore the relationship of the endothelial NOS gene to other similar genes, and to delineate the genetic factors involved in regulating endothelial NOS activity, we isolated the human gene encoding the endothelial NOS. Genomic clones containing the 5' end of this gene were identified in a human genomic library by applying a polymerase chain reaction (PCR)-based approach. Identification of the human gene for endothelial NOS (NOS3) was confirmed by nucleotide sequence analysis of the first coding exon, which was found to be identical to its cognate cDNA. The NOS3 gene spans at least 20 kb and appears to contain multiple introns. The transcription start site and promoter region of the NOS3 gene were identified by primer extension and ribonuclease protection assays. Sequencing of the putative promoter revealed consensus sequences for the shear stress-response element, as well as cytokine-responsive cis regulatory sequences, both possibly important to the roles played by NOS3 in the normal and the diseased cardiovascular system.(ABSTRACT TRUNCATED AT 250 WORDS) |
| 10821138 | Association of polymorphism in the promoter region of the apolipoprotein E gene with diastolic blood pressure in normotensive Japanese.The epsilon4 allele of apolipoprotein E (APOE) is reported to be a genetic risk factor of atherosclerosis through hyperlipidemia and late-onset Alzheimer's dementia. A recent report showed that a genetic variant (A -491T) in the promoter region of the APOE gene increases the risk of Alzheimer's disease. In the present study, we examined whether these APOE polymorphisms were genetically involved in essential hypertension. Japanese hypertensives (n=180) with a family history of hypertension and normotensive controls (n=195, sex and age matched with hypertensives) were recruited from the outpatients of Osaka University Hospital, and an informed consent to participate in the study was obtained from each person. APOE polymorphisms were determined using polymerase chain reaction-restriction fragment length polymorphism (PCR-RFLP). The frequencies of the A -491 allele in hypertensives and normotensives were 0.98 and 0.97, respectively, and the TT/-491 genotype was not found in either group. No significant differences between hypertensives and normotensives were observed in allele frequencies in either APOE polymorphism; however, the mean diastolic blood pressure in normotensive subjects with AA/-491 was significantly higher than in the subjects with AT/-491 (p < 0.01). These results suggest that the presence of the APOE promoter polymorphism is not a major risk factor for hypertension but that it does have some minor effect on basal blood pressure variation. |
| 15834589 | Variation in ITGB3 has sex-specific associations with plasma lipoprotein(a) and whole blood serotonin levels in a population-based sample.A recent genome-scan identified the Leu33Pro polymorphism in the beta3 integrin (ITGB3) gene as a quantitative trait locus for whole blood serotonin level in a large Hutterite pedigree. Because both the Leu33Pro polymorphism and the serotonin system have been implicated in cardiovascular disease (CVD) risk and treatment response, we studied additional variation in ITGB3 and its relationship to intermediate phenotypes associated with CVD in the same population. We examined associations between 15 single nucleotide polymorphisms (SNPs) across ITGB3 and five CVD-related traits in the Hutterites: plasma levels of high density lipoprotein-cholesterol (HDL-c), triglycerides (TG), low density lipoprotein-cholesterol (LDL-c), and lipoprotein(a) [Lp(a)] and blood pressure or hypertension. Seven of these SNPs in ITGB3 were associated with whole blood serotonin. Among the intermediate CVD-related phenotypes, only Lp(a) was associated with multiple ITGB3 SNPs, five of which were also associated with serotonin. A sex-stratified analysis revealed that the association between ITGB3 and Lp(a) is present only in females, whereas the association between ITGB3 and serotonin is concentrated in males. Our results suggest that variation in ITGB3 in addition to Leu33Pro could contribute to susceptibility to CVD and serotonin in a sex-specific manner. |
| 15857159 | Relationship among urinary albumin excretion rate, lipoprotein lipase PvuII polymorphism and plasma fibrinogen in type 2 diabetic patients.Plasma fibrinogen level represents a strong cardiovascular risk factor and is regulated by an interplay of genetic and environmental factors. Hyperfibrinogenemia frequently occurs in cluster with dyslipidemia within the frame of insulin resistance syndrome (IRS) and type 2 diabetes mellitus. Genetic variants with a pleiotropic effect have been proposed to cause IRS features including hyperfibrinogenemia. We studied the influence of polymorphisms in lipoprotein lipase (LPL) gene, beta-fibrinogen gene (FIBB) and environmental factors on plasma fibrinogen levels in type 2 diabetes patients. 131 type 2 diabetes patients (mean age 62+/-10 years, 33% male) were genotyped for polymorphisms in LPL gene (intron 6 PvuII, intron 8 HindIII) and FIBB gene (-148C/T, -455G/A) by PCR-RFLP method. Fibrinogen was measured by thrombin coagulation method, albuminuria by immunoturbidimetric assay. Polymorphism LPL PvuII showed a gene-dose effect on fibrinogen levels, with the highest fibrinogen in P-P- homozygotes (p = 0.05, analysis of variance). P-carriers (P-P- and P+P- combined) had significantly higher fibrinogen levels compared with P+P+ homozygotes (3.74+/-1.40 g/l vs 3.06+/-1.20 g/l, p=0.03). Other studied polymorphisms were not significantly related to fibrinogen levels. Age- and sex-adjusted fibrinogenemia correlated significantly with albuminuria (r = 0.48, p=0.001), serum uric acid (r = 0.42, p=0.006) and serum creatinine (r = 0.32, p=0.04). Multiple stepwise linear regression identified interaction term of LPL PvuII and albuminuria as an independent predictor of fibrinogen level, explaining 18% of fibrinogen variance. Albuminuria thus appears to be the best predictor of fibrinogen plasma levels in type 2 diabetic patients. Relationship between albuminuria and fibrinogenemia may be modified by the genotype LPL PvuII, which also shows a weak association with plasma fibrinogen level in type 2 diabetes patients. |
| 12941783 | Impaired cathepsin L gene expression in skeletal muscle is associated with type 2 diabetes.To identify abnormally expressed genes associated with muscle insulin resistance or type 2 diabetes, we screened the mRNA populations using cDNA differential display combined with relative RT-PCR analysis from muscle biopsies of diabetes-prone C57BL/6J and diabetes-resistant NMRI mice fed with a high-fat or normal diet for 3 or 15 months. Six abnormally expressed genes were isolated from the mice after a 3-month fat feeding; one of them was cathepsin L. No significant difference in mRNA levels of these genes was observed between fat- and normal-diet conditions in either strains. However, cathepsin L mRNA levels in muscle were higher in normal diet-fed C57BL/6J mice compared with normal diet-fed NMRI mice at 3 months (0.72 +/- 0.04 vs. 0.51 +/- 0.04 relative units, P < 0.01, n = 8-10) and at 15 months (0.41 +/- 0.05 vs. 0.27 +/- 0.04 relative units, P = 0.01, n = 9-10). Further, cathepsin L mRNA levels in muscle correlated inversely with plasma glucose in both strains regardless of diets at 3 (r = -0.49, P < 0.01, n = 31) and 15 (r = -0.42, P = 0.007, n = 39) months. To study whether cathepsin L plays a role in human diabetes, we measured cathepsin L mRNA levels in muscle biopsies taken before and after an insulin clamp from 12 monozygotic twin pairs discordant for type 2 diabetes and from 12 control subjects. Basal cathepsin L mRNA levels were not significantly different between the study groups. Insulin infusion increased cathepsin L mRNA levels in control subjects from 1.03 +/- 0.30 to 1.90 +/- 0.32 relative units (P = 0.03). Postclamp cathepsin L mRNA levels were lower in diabetic twins but similar in nondiabetic twins compared with control subjects (0.66 +/- 0.22, 1.16 +/- 0.18 vs. 1.38 +/- 0.21 relative units, P < 0.02, NS, respectively). Further, postclamp cathepsin L mRNA levels were correlated with insulin-mediated glucose uptake (r = 0.37, P = 0.03), particularly, with glucose oxidation (r = 0.37, P = 0.03), and fasting glucose concentrations (r = -0.45, P < 0.01) across all three study groups. In conclusion, muscle cathepsin L gene expression is increased in diabetes-prone mice and related to glucose tolerance. In humans, insulin-stimulated cathepsin L expression in skeletal muscle is impaired in diabetic but not in nondiabetic monozygotic twins, suggesting that the changes may be secondary to impaired glucose metabolism. |
| 15936464 | Apolipoprotein e4 allele and the risk of CAD death in type 2 diabetes mellitus with ischaemia electrocardiographic change.The presence of apolipoprotein (Apo) e4 allele is reported to be associated with the increased risk of coronary artery disease (CAD), as well as the impairment of endothelium-dependent arterial dilation in type 2 diabetes mellitus. Therefore, we hypothesized that Apo e4 allele increases the death risk from coronary artery disease in type 2 diabetes with ischaemia electrocardiographic change. From January 1993 to December 1999, 46 type 2 diabetic patients with e4/4 or e4/3, 96 with e3/3 and 45 with e2/2 or e3/2 genotypes were recruited. All subjects were unrelated elderly type 2 diabetic patients with ischaemia electrocardiographic change, aged 60-87 years, and their cardiac function were all the class I stage at their time of enrollment. A follow-up study of 3-10 years was undergone. The results are as follows: At baseline, serum total cholesterol and low-density lipoprotein (LDL) cholesterol concentrations were higher in subjects with e4/3 or e4/4 than in subjects with e2/2 or e3/2 (p<0.05). Lipoprotein(a) concentration was lower in subjects with e2/2 or e3/2 than in subjects with e3/3 and e4/3 or e4/4 (p < 0.05). During the 3-10 years follow-up period, a total of 55 patients who died from CAD were recorded in this sample. Compared with patients with e3/3 (p = 0.024) and patients with e2/2 or e2/3 genotypes (p = 0.002), the mortality rate of CAD in patients with e4/3 or e4/4 genotypes was the highest (47.8%). Stepwise discriminant analysis revealed that in the diabetic population studied Apo e4 allele was independently and significantly associated with CAD death (B = 0.65). However, the strength of the association decreased (B = 0.44) when total cholesterol, LDL-cholesterol and lipoprotein(a) were included in the model. Therefore, we concluded that Apo e4 allele increases the risk of CAD death in elderly type 2 diabetes mellitus with ischaemia electrocardiographic change. |
| 16599342 | Apolipoprotein E and cognition in community-based samples of African Americans and Caucasians.To compare relative frequencies of apolipo-protein E (APOE) alleles in African-American and Caucasian populations and test associations with cognition, we studied two community-based samples: one of 253 African Americans and another of 466 Caucasians age 60-84 years. Logistic regression, adjusting for age, sex, education, and history of hypertension and diabetes was used to associate APOE with five cognitive measures. The APOE-epsilon4 allele frequency was 29.5% in African Americans and 12.1% in Caucasians. In the African Americans, no association was found between the presence of the APOE-epsilon4 allele and any of the cognitive measures. Among Caucasians, APOE-epsilon4 carriers performed more poorly on three of the five tests. We also report a considerably higher frequency of the APOE-epsilon4 allele in our African-American sample compared to other US-based studies. |
| 18711366 | SNPs in KCNQ1 are associated with susceptibility to type 2 diabetes in East Asian and European populations.We conducted a genome-wide association study using 207,097 SNP markers in Japanese individuals with type 2 diabetes and unrelated controls, and identified KCNQ1 (potassium voltage-gated channel, KQT-like subfamily, member 1) to be a strong candidate for conferring susceptibility to type 2 diabetes. We detected consistent association of a SNP in KCNQ1 (rs2283228) with the disease in several independent case-control studies (additive model P = 3.1 x 10(-12); OR = 1.26, 95% CI = 1.18-1.34). Several other SNPs in the same linkage disequilibrium (LD) block were strongly associated with type 2 diabetes (additive model: rs2237895, P = 7.3 x 10(-9); OR = 1.32, 95% CI = 1.20-1.45, rs2237897, P = 6.8 x 10(-13); OR = 1.41, 95% CI = 1.29-1.55). The association of these SNPs with type 2 diabetes was replicated in samples from Singaporean (additive model: rs2237895, P = 8.5 x 10(-3); OR = 1.14, rs2237897, P = 2.4 x 10(-4); OR = 1.22) and Danish populations (additive model: rs2237895, P = 3.7 x 10(-11); OR = 1.24, rs2237897, P = 1.2 x 10(-4); OR = 1.36). |
| 19102712 | Glutathione S-transferase T1- and M1-null genotypes and coronary artery disease risk in patients with Type 2 diabetes mellitus.INTRODUCTION: Since long-term exposure to oxidative stress is strongly implicated in the pathogenesis of diabetic complications, polymorphic genes of detoxifying enzymes must be involved in the development of coronary artery disease (CAD). We assessed the potential glutathione S-transferase (GST) gene-gene (GSTM1(null)-GSTT1(null)) and gene-smoking interactions on the development of CAD in patients with Type 2 diabetes. MATERIALS & METHODS: In a case-only design, we enrolled 231 patients with Type 2 diabetes (147 male, 66.1 +/- 9.7 years) referred to our institute for coronary angiography investigation. CAD was diagnosed if there was over 50% obstruction of one or more major vessels. RESULTS: Coronary angiography revealed significant CAD in 184 patients (80%). Male gender (p < 0.001), smoking habits (p = 0.003) and GSTT1(null) genotype (p = 0.003) were significantly correlated with the increasing extent of the coronary atherosclerosis. Case-only analysis revealed that patients with both M(null)-T(null) genotypes had the highest risk for 3-vessel CAD compared with patients who express both GST genes (odds ratio: 3.1; 95% confidence interval: 1.0-10.3, p = 0.04). A nearly threefold interaction existed between cigarette smoking and M(null)-T(null) genotypes (odds ratio: 2.9, 95% confidence interval: 1.7-7.8, p = 0.03). A significant interaction between M(null)-T(null) genotypes and smoking was also observed on the increasing number of coronary vessels that were diseased (chi(2) = 14.0; p = 0.03). CONCLUSION: These data suggest that polymorphisms in GSTM1 and GSTT1 genes are risk factors for CAD in Type 2 diabetic patients, especially among smokers. These genetic markers may permit the targeting of preventive and early intervention on high-risk patients to reduce their cardiovascular risk. |
| 21600576 | Serum retinol-binding protein 4, leptin, and plasma asymmetric dimethylarginine levels in obese and nonobese young women with polycystic ovary syndrome.OBJECTIVE: To evaluate retinol-binding protein 4 (RBP4), leptin, and asymmetric dimethylarginine (ADMA) levels in young women with polycystic ovary syndrome (PCOS) and to investigate their relationship with each other and with clinical, metabolic, and hormonal parameters. DESIGN: Clinical study. SETTING: University hospital. PATIENT(S): Fifty-seven young women with PCOS (obese [n = 27] and nonobese [n = 30]) and 27 age-matched healthy controls. INTERVENTION(S): History and physical examination, peripheral venous blood sampling. MAIN OUTCOME MEASURE(S): Asymmetric dimethylarginine, RBP4, leptin, LH, FSH, DHEAS, total T, E(2), total cholesterol, high-density lipoprotein (HDL) cholesterol, low-density lipoprotein (LDL) cholesterol, triglyceride (TG), and homeostasis model assessment insulin resistance index (HOMA-IR). RESULT(S): Obese women with PCOS had significantly higher HOMA-IR, DHEAS, leptin, RBP4, and ADMA levels. Leptin levels were significantly increased in nonobese subjects with PCOS. Leptin and ADMA levels were positively correlated with HOMA-IR in PCOS. There was no correlation between RBP4 and HOMA-IR. Leptin, RBP4, and ADMA levels are positively correlated in PCOS. CONCLUSION(S): [1] Young obese women with PCOS have increased ADMA, RBP4, and leptin levels, and they are positively correlated with each other. [2] The increased levels of leptin are independent of obesity, and leptin seems to have an association with IR. [3] Levels of RBP4 may not reflect IR in PCOS. |
| 15207892 | Apolipoprotein E polymorphism is not associated with spinal bone mineral density in peri- and postmenopausal Greek women.OBJECTIVES: A number of studies have shown a positive relation between ApoE gene and osteoporosis or fracture risk but this finding has not been uniform in all populations studied. The aim of the present study was to determine the possible effect of ApoE gene polymorphism on spinal bone mineral density and metabolic bone markers in Greek women. METHODS: One hundred and forty-seven healthy peri- and postmenopausal women (mean age 54.3 +/- 7.8 years) participated in the study. In all participants, ApoE gene genotype was determined and spinal bone mineral density (BMD) as well as biochemical bone markers were measured. The ApoE genotypes distribution was 0.7% (n = 1) for E2/2, 5.4% (n = 8) for E2/3, 2% (n = 3) for E2/4, 73.5% (n = 108) for E3/3, 16.3% (n = 24) for E3/4 and 2% (n = 3) for E4/4. Participants were divided in two groups according to the presence of the E4 haplotype: E4 carriers (n = 30) and E4 non-carriers (n = 117). RESULTS: Spinal BMD was similar in the two groups, after adjusting for age, weight, height and years since menopause (mean +/- S.D., 0.835 +/- 0.16 g/cm2 in E4 non-carriers versus 0.831 +/- 0.16 g/cm2 in E4 carriers, P = 0.99). Serum osteocalcin levels did not differ significantly in the two groups (median (interquartile range, IQR), 0.55 (0.58) nmol/l in E4 non-carriers versus 0.51 (0.43) nmol/l in E4 carriers), whereas urinary hydroxyproline/creatinine ratio was significantly higher in the E4 non-carriers group (median (IQR), 5.18 (6.04) micromol/mmol in E4 non-carriers versus 1.73 (3.45) micromol/mmol in E4 carriers, P < 0.01). Urinary pyridinoline/creatinine and deoxypyridinoline/creatinine ratios, measured in a subgroup of 51 women, were similar between ApoE carriers and non-carriers, respectively (median (IQR), 25.1 (9.3) nmol/mmol in E4 non-carriers versus 21.8 (7) nmol/mmol in E4 carriers and 6.7 (3.1) nmol/mmol in E4 non-carriers versus 7 (2.2) nmol/mmol in E4 carriers). CONCLUSION: In conclusion, in a Greek female postmenopausal population, ApoE gene does not seem to play an important role in determining BMD and neither does it affect the majority of metabolic bone markers. |
| 17357883 | The association between type 2 diabetes mellitus and A1/A2 polymorphism of glycoprotein IIIa gene.Glycoprotein IIIa (GpIIIa) is a membrane receptor, found in various tissues, that has two alleles: A1 and A2. Signalling cascade of GpIIIa is modulated by enzymes called calpains, proteases that may also influence glucose metabolism. There is one small study that shows a high association of A1/A2 polymorphism with type 2 diabetes mellitus. In our research we planned to evaluate the association of A1/A2 polymorphism with type 2 diabetes in a population of patients with ST elevation acute myocardial infarction (STEMI). The study comprised 352 individuals. From the cohort of patients hospitalised for STEMI we chose 113 patients with diagnosed diabetes (diabetic group) and 118 patients with STEMI and normal glucose metabolism (non-diabetic group). The population group consisted of 121 persons. Genotyping was performed by the restriction fragments length polymorphism (RFLP) method. The frequency of alleles in all groups was in Hardy-Weinberg equilibrium. The percentage of A2 allele carriers was comparable among all groups : 20.4% (diabetic patients), 23.7% (nondiabetic) and 21.5% (control group) (p>0.05). There was no significant difference in frequency of A2 allele among the groups. We have not observed any association between GpIIIa polymorphism with either type 2 diabetes or STEMI. |
| 18497882 | Overindulgence and metabolic syndrome: is FoxO1 a missing link?Excessive production of triglyceride-rich VLDL, which can result from dietary overindulgence, underlies metabolic syndrome--a combination of disorders including high blood pressure, obesity, high triglyceride, and insulin resistance--and places individuals at increased risk of developing cardiovascular disease and type 2 diabetes. However, the link between VLDL overproduction and insulin resistance has remained unclear. VLDL assembly in the liver is catalyzed by microsomal triglyceride transfer protein (MTP). In this issue of the JCI, Kamagate et al. investigate the events controlling hepatic MTP expression and VLDL production and secretion (see the related article beginning on page 2347). They demonstrate that MTP is a target of the transcription factor FoxO1 and that excessive VLDL production associated with insulin resistance is caused by the inability of insulin to regulate FoxO1 transcriptional activation of MTP. |
| 19696412 | Urotensin II receptor knockout mice on an ApoE knockout background fed a high-fat diet exhibit an enhanced hyperlipidemic and atherosclerotic phenotype.RATIONALE: Expression of the vasoactive peptide Urotensin II (UII) is elevated in a number of cardiovascular diseases. OBJECTIVE: Here, we sought to determine the effect of UII receptor (UT) gene deletion in a mouse model of atherosclerosis. METHODS AND RESULTS: UT knockout (KO) mice were crossed with ApoE KO mice to generate UT/ApoE double knockout (DKO) mice. Mice were placed on a high-fat Western-type diet for 12 weeks. We evaluated the degree of atherosclerosis and hepatic steatosis by histology. In addition, serum glucose, insulin, and lipids were determined. DKO mice exhibited significantly increased atherosclerosis compared to ApoE KO mice (P<0.05). This was associated with a significant increase in serum insulin and lipids (P<0.001) but a decrease in hepatic steatosis (P<0.001). UT gene deletion led to a significant increase in systolic pressure and pulse pressure. RT-PCR and immunoblot analyses showed significant reductions in hepatic scavenger receptors, nuclear receptors, and acyl-CoA:cholesterol acyltransferase (ACAT1) expression in DKO mice. UII induced a significant increase in intracellular cholesteryl ester formation in primary mouse hepatocytes, which was blocked by the MEK inhibitor, PD98059. Hepatocytes of UTKO mice showed a significant reduction in lipoprotein uptake compared to wild-type mice. CONCLUSIONS: We propose that UT gene deletion in an ApoE-deficient background promotes downregulation of ACAT1, which in turn attenuates hepatic lipoprotein receptor-mediated uptake and lipid transporter expression. As the liver is the main organ for uptake of lipoprotein-derived lipids, DKO leads to an increase in hyperlipidemia, with a concomitant decrease in hepatic steatosis, and consequently increased atherosclerotic lesion formation. Furthermore, the hypertension associated with UT gene deletion is likely to contribute to the increased atherosclerotic burden. |
| 19837407 | Impact of android overweight or obesity and insulin resistance on basal and postprandial SR-BI and ABCA1-mediated serum cholesterol efflux capacities.Since android overweight/obesity and insulin resistance are independent risk factors for cardiovascular disease, we investigated their impact on basal and postprandial scavenger receptor BI (SR-BI) and ATP binding cassette transporter A1 (ABCA1)-mediated serum cholesterol efflux. Twelve android overweight to obese and 9 normal weight controls women underwent body composition analysis by dual energy X-ray absorptiometry, a euglycemic hyperinsulinemic clamp, and an oral fat load with blood sampling at initial time (T0), 4h (T4) and 10h (T10) after the fat load. Serum lipids and HDL-parameters, capacities of serum to promote cholesterol efflux from SR-BI expressing Fu5AH hepatoma cells or from ABCA1-expressing J774 macrophages and to abilities of serum to induce a net removal of cholesterol from macrophage foam cells were measured at T0, T4 and T10. Sera from overweight/obese exhibited moderately decreased SR-BI-mediated cholesterol efflux capacities, in accordance with reduced HDL concentrations, but importantly increased ABCA1-mediated cholesterol efflux and increased cholesterol extraction capacities over the postprandial period, partly related to higher prebeta-HDL concentrations. In multiple regression analyses, android obesity-related parameters and HDL-PL or prebeta-HDL levels remained the only independent correlates for SR-BI or ABCA1-dependent fractional cholesterol efflux while only prebeta-HDL levels remained correlated to cholesterol extraction capacities. Our results suggest that android overweight/obesity may not result in an impaired cholesterol efflux capacity. |
| 23056479 | Lactobacillus reuteri prevents diet-induced obesity, but not atherosclerosis, in a strain dependent fashion in Apoe-/- mice.OBJECTIVE: To investigate whether the specific strains of Lactobacillus reuteri modulates the metabolic syndrome in Apoe-/- mice. METHODS: 8 week-old Apoe-/- mice were subdivided into four groups who received either L. reuteri ATCC PTA 4659 (ATCC), DSM 17938 (DSM), L6798, or no bacterial supplement in the drinking water for 12 weeks. The mice were fed a high-fat Western diet with 0.2% cholesterol and body weights were monitored weekly. At the end of the study, oral glucose and insulin tolerance tests were conducted. In addition, adipose and liver weights were recorded along with analyses of mRNA expression of ileal Angiopoietin-like protein 4 (Angptl4), the macrophage marker F4/80 encoded by the gene Emr1 and liver Acetyl-CoA carboxylase 1 (Acc1), Fatty acid synthase (Fas) and Carnitine palmitoyltransferase 1a (Cpt1a). Atherosclerosis was assessed in the aortic root region of the heart. RESULTS AND CONCLUSIONS: Mice receiving L. reuteri ATCC gained significantly less body weight than the control mice, whereas the L6798 mice gained significantly more. Adipose and liver weights were also reduced in the ATCC group. Serum insulin levels were lower in the ATCC group, but no significant effects were observed in the glucose or insulin tolerance tests. Lipogenic genes in the liver were not altered by any of the bacterial treatments, however, increased expression of Cpt1a was found in the ATCC group, indicating increased beta-oxidation. Correspondingly, the liver trended towards having lower fat content. There were no effects on inflammatory markers, blood cholesterol or atherosclerosis. In conclusion, the probiotic L. reuteri strain ATCC PTA 4659 partly prevented diet-induced obesity, possibly via a previously unknown mechanism of inducing liver expression of Cpt1a. |
| 22623957 | Overexpression of Akt1 enhances adipogenesis and leads to lipoma formation in zebrafish.BACKGROUND: Obesity is a complex, multifactorial disorder influenced by the interaction of genetic, epigenetic, and environmental factors. Obesity increases the risk of contracting many chronic diseases or metabolic syndrome. Researchers have established several mammalian models of obesity to study its underlying mechanism. However, a lower vertebrate model for conveniently performing drug screening against obesity remains elusive. The specific aim of this study was to create a zebrafish obesity model by over expressing the insulin signaling hub of the Akt1 gene. METHODOLOGY/PRINCIPAL FINDINGS: Skin oncogenic transformation screening shows that a stable zebrafish transgenic of Tg(krt4Hsa.myrAkt1)(cy18) displays severely obese phenotypes at the adult stage. In Tg(krt4:Hsa.myrAkt1)(cy18), the expression of exogenous human constitutively active Akt1 (myrAkt1) can activate endogenous downstream targets of mTOR, GSK-3alpha/beta, and 70S6K. During the embryonic to larval transitory phase, the specific over expression of myrAkt1 in skin can promote hypertrophic and hyperplastic growth. From 21 hour post-fertilization (hpf) onwards, myrAkt1 transgene was ectopically expressed in several mesenchymal derived tissues. This may be the result of the integration position effect. Tg(krt4:Hsa.myrAkt1)(cy18) caused a rapid increase of body weight, hyperplastic growth of adipocytes, abnormal accumulation of fat tissues, and blood glucose intolerance at the adult stage. Real-time RT-PCR analysis showed the majority of key genes on regulating adipogenesis, adipocytokine, and inflammation are highly upregulated in Tg(krt4:Hsa.myrAkt1)(cy18). In contrast, the myogenesis- and skeletogenesis-related gene transcripts are significantly downregulated in Tg(krt4:Hsa.myrAkt1)(cy18), suggesting that excess adipocyte differentiation occurs at the expense of other mesenchymal derived tissues. CONCLUSION/SIGNIFICANCE: Collectively, the findings of this study provide direct evidence that Akt1 signaling plays an important role in balancing normal levels of fat tissue in vivo. The obese zebrafish examined in this study could be a new powerful model to screen novel drugs for the treatment of human obesity. |
| 15060087 | The lipoprotein lipase S447X polymorphism and plasma lipids: interactions with APOE polymorphisms, smoking, and alcohol consumption.We studied 4,058 subjects from a representative sample of the Singapore population 1) to determine the association between the S447X polymorphism at the LPL locus and serum lipid concentration in Chinese, Malays, and Asian Indians living in Singapore and 2) to explore any interactions with apolipoprotein E (APOE) genotype, exercise, obesity, cigarette smoking, and alcohol intake. Information on obesity, lifestyle factors (including smoking, alcohol consumption, and exercise frequency), glucose tolerance, and fasting lipids was obtained. Male and female carriers of the X447 allele had lower serum triglyceride concentrations and higher HDL cholesterol (HDL-C) concentrations. The association between the X447 allele and serum HDL-C concentration was modulated by APOE genotype in males and cigarette smoking and alcohol intake in females. The effect of the X447 allele was greatest in men who carried the E4 allele and women who smoked or consumed alcohol. The X447 allele at the LPL locus is common and associated with a less atherogenic lipid profile in Asian populations. Interactions with APOE genotype, cigarette smoking, and alcohol intake reinforce the importance of examining genetic associations, such as this one, in the context of the population of interest. |
| 18477980 | [The role of angiotensin-converting enzyme and apolipoprotein E in the development of intracranial aneurysms.]The distribution of allele and genotype frequencies of the Alu-insertion polymorphism of the angiotensin-converting enzyme (ACE) gene and missence mutations leading to the substitution of arginine to cysteine in positions 112 and 158 of apolipoprotein E (APOE) has been studied in 166 patients with brain intracranial aneurysms and in 192 controls of Russian origin from Ural region. Brain vascular aneurysms with hypertension were associated with the D\*D\* ACE genotype in men and with the e2 allele and the e2/e3 APOE genotype in women. The association was also observed between the e2 allele and the e2/e3 APOE genotype and family history of stroke, hemorrhages and aneurysms in patients. Men with the I\*D\* ACE genotype and the e4 APOE allele were at lower risk. |
| 18805911 | Acylation stimulating protein but not complement C3 associates with metabolic syndrome components in Chinese children and adolescents.OBJECTIVE: Childhood obesity is increasing worldwide and is increasingly associated with metabolic syndrome (MetS). Our aim was to examine acylation stimulating protein (ASP) and its precursor complement C3, in normal, overweight, and obese Chinese children and adolescents, and the relationships with body size, blood parameters, pubertal development, family environment, and MetS. METHODS: Children and adolescents (n=1603) from 6 to 18 years, boys (n=873) and girls (n=730), including normal weight (n=603), overweight (n=291) and obese (n=709) were assessed for body size parameters, pubertal development, blood lipids, glucose, insulin, ASP, and C3. RESULTS: ASP levels were increased in overweight and obese versus normal weight (P<0.001), while C3 showed little variation. This effect of overweight/obesity remained throughout early stages when boys and girls were separated by pubertal development or age, although age and pubertal status itself had no effect. Separation based on ASP quintiles demonstrated significant associations with blood cholesterol, triglyceride, low-density lipoprotein cholesterol (LDL-Chol), glucose, insulin, and homeostatic model assessment of insulin resistance in boys, and LDL-Chol, high-density lipoprotein cholesterol, and glucose in girls. A positive correlation with mother's body mass index in boys and girls (P=0.002 and P=0.014 respectively) as well as birth weight (P<0.001) was noted. MetS was strongly associated with increased ASP, the presence of a single MetS factor (especially hypertension, central obesity, or hyperglycemia) was associated with increased ASP. CONCLUSION: Changes in the plasma adipokine ASP in early obesity are associated with blood lipid and glucose modifications, family environment, and distinct MetS risk factors. |
| 20705925 | DNA damage links mitochondrial dysfunction to atherosclerosis and the metabolic syndrome.RATIONALE: DNA damage is present in both genomic and mitochondrial DNA in atherosclerosis. However, whether DNA damage itself promotes atherosclerosis, or is simply a byproduct of the risk factors that promote atherosclerosis, is unknown. OBJECTIVE: To examine the effect of DNA damage on atherosclerosis, we studied apolipoprotein (Apo)E(-/-) mice that were haploinsufficient for the protein kinase ATM (ataxia telangiectasia mutated), which coordinates DNA repair. METHODS AND RESULTS: ATM(+/-)/ApoE(-/-) mice developed accelerated atherosclerosis and multiple features of the metabolic syndrome, including hypertension, hypercholesterolemia, obesity, steatohepatitis, and glucose intolerance. Transplantation with ATM(+/+) bone marrow attenuated atherosclerosis but not the metabolic syndrome. ATM(+/-) smooth muscle cells and macrophages showed increased nuclear DNA damage and defective DNA repair signaling, growth arrest, and apoptosis. Metabolomic screening of ATM(+/-)/ApoE(-/-) mouse tissues identified metabolic changes compatible with mitochondrial defects, with increased beta-hydroxybutyrate but reduced lactate, reduced glucose, and alterations in multiple lipid species. ATM(+/-)/ApoE(-/-) mouse tissues showed an increased frequency of a mouse mitochondrial "common" deletion equivalent and reduced mitochondrial oxidative phosphorylation. CONCLUSIONS: We propose that failure of DNA repair generates defects in cell proliferation, apoptosis, and mitochondrial dysfunction. This in turn leads to ketosis, hyperlipidemia, and increased fat storage, promoting atherosclerosis and the metabolic syndrome. Prevention of mitochondrial dysfunction may represent a novel target in cardiovascular disease. |
| 21033077 | [Correlation of metabolic syndrome clinical signs and genetic determinants at children with obese].The aim of the work was to study the clinical and genetic factors at children with obese that predispose to the development of MS, and the development of algorithm for generating risk of MS. MATERIALS AND METHODS: Two comparable age and sex groups of children--148 children with obesity and 46--with normal body weight. We assessed anthropometric indices, blood pressure (BP), lipid profile, carbohydrate metabolism, the level of uric acid. 83 children with obesity were genotyped for polymorphisms: I/D gene ACE, G-75A ApoA1, S19W ApoA5, Sstl ApoC3, E2/E3/E4 ApoE and W/R ADRB3. RESULTS: 98,0% of children had abdominal obesity. In 35,8% was identified high blood pressure. In 47,4% was diagnosed hypo-alpha cholesterolemia and/or hypertriglyceridemia (HTG). In 21,0% of children was identified hyperglycemia. 25,7%were suffered from hyperuricemia. Among the genotyped children 57,0% of homo-and heterozygous carriers of D allele ACE gene had high blood pressure. More than half of the holders of 19W-allele ApoA5 (68,5%),--75A-allele of ApoA1 (56,0%), 52-allele of the gene ApoC3 (53,0%), E4-ApoE gene (85,7%), in the heterozygous state had metabolic TG and/or HDL. In 60,3% of the carriers W/W genotype of ADRB3 gene revealed a combination of hyperglycemia with hyperinsulinemia and/or TG. CONCLUSION: As a result of, aiming aimed at early detection of the major manifestations of MS clinical and genetic study was revealed stable combination of constitutional, metabolic and molecular-genetic factors. Based on these data was developed algorithm for forming groups at risk of MS and individual tactics to prevent and/or therapy. |
| 22961083 | Apolipoprotein E2 accentuates postprandial inflammation and diet-induced obesity to promote hyperinsulinemia in mice.Genetic studies have revealed the association between the epsilon2 allele of the apolipoprotein E (apoE) gene and greater risk of metabolic diseases. This study compared C57BL/6 mice in which the endogenous mouse gene has been replaced by the human APOE2 or APOE3 gene (APOE2 and APOE3 mice) to identify the mechanism underlying the relationship between epsilon2 and obesity and diabetes. In comparison with APOE3 mice, the APOE2 mice had elevated fasting plasma lipid and insulin levels and displayed prolonged postprandial hyperlipidemia accompanied by increased granulocyte number and inflammation 2 h after being fed a lipid-rich meal. In comparison with APOE3 mice, the APOE2 mice also showed increased adiposity when maintained on a Western-type, high-fat, high-cholesterol diet. Adipose tissue dysfunction with increased macrophage infiltration, abundant crown-like structures, and inflammation were also observed in adipose tissues of APOE2 mice. The severe adipocyte dysfunction and tissue inflammation corresponded with the robust hyperinsulinemia observed in APOE2 mice after being fed the Western-type diet. Taken together, these data showed that impaired plasma clearance of apoE2-containing, triglyceride-rich lipoproteins promotes lipid redistribution to neutrophils and adipocytes to accentuate inflammation and adiposity, thereby accelerating the development of hyperinsulinemia that will ultimately lead to advanced metabolic diseases. |
| 23680665 | Neuronal nitric oxide synthase is phosphorylated in response to insulin stimulation in skeletal muscle.Type 2 Diabetes (T2DM) is the seventh leading cause of death in the United States, and is quickly becoming a global pandemic. T2DM results from reduced insulin sensitivity coupled with a relative failure of insulin secretion. Reduced insulin sensitivity has been associated with reduced nitric oxide synthase (NOS) activity and impaired glucose uptake in T2DM skeletal muscle. Upon insulin stimulation, NO synthesis increases in normal adult skeletal muscle, whereas no such increase is observed in T2DM adults. Endothelial NOS is activated by phosphorylation in the C-terminal tail in response to insulin. Neuronal NOS (nNOS), the primary NOS isoform in skeletal muscle, contains a homologous phosphorylation site, raising the possibility that nNOS, too, may undergo an activating phosphorylation event upon insulin treatment. Yet it remains unknown if or how nNOS is regulated by insulin in skeletal muscle. Data shown herein indicate that nNOS is phosphorylated in response to insulin in skeletal muscle and that this phosphorylation event occurs rapidly in C2C12 myotubes, resulting in increased NO production. In vivo phosphorylation of nNOS was also observed in response to insulin in mouse skeletal muscle. These results indicate, for the first time, that nNOS is phosphorylated in skeletal muscle in response to insulin and in association with increased NO production. |
| 11156623 | Genetic analysis of case/control data using estimated haplotype frequencies: application to APOE locus variation and Alzheimer's disease.There is growing debate over the utility of multiple locus association analyses in the identification of genomic regions harboring sequence variants that influence common complex traits such as hypertension and diabetes. Much of this debate concerns the manner in which one can use the genotypic information from individuals gathered in simple sampling frameworks, such as the case/control designs, to actually assess the association between alleles in a particular genomic region and a trait. In this paper we describe methods for testing associations between estimated haplotype frequencies derived from multilocus genotype data and disease endpoints assuming a simple case/control sampling design. These proposed methods overcome the lack of phase information usually associated with samples of unrelated individuals and provide a comprehensive way of assessing the relationship between sequence or multiple-site variation and traits and diseases within populations. We applied the proposed methods in a study of the relationship between polymorphisms within the APOE gene region and Alzheimer's disease. Cases and controls for this study were collected from the United States and France. Our results confirm the known association between the APOE locus and Alzheimer's disease, even when the epsilon 4 polymorphism is not contained in the tested haplotypes. This suggests that, in certain situations, haplotype information and linkage disequilibrium-induced associations between polymorphic loci that neighbor loci harboring functional sequence variants can be exploited to identify disease-predisposing alleles in large, freely mixing populations via estimated haplotype frequency methods. |
| 17109061 | Links between cardiovascular disease and osteoporosis in postmenopausal women: serum lipids or atherosclerosis per se?INTRODUCTION AND HYPOTHESIS: Epidemiological observations suggest links between osteoporosis and risk of acute cardiovascular events and vice versa. Whether the two clinical conditions are linked by common pathogenic factors or atherosclerosis per se remains incompletely understood. We investigated whether serum lipids and polymorphism in the ApoE gene modifying serum lipids could be a biological linkage. METHODS: This was an observational study including 1176 elderly women 60-85 years old. Women were genotyped for epsilon (epsilon) allelic variants of the ApoE gene, and data concerning serum lipids (total cholesterol, triglycerides, HDL-C, LDL-C, apoA1, ApoB, Lp(a)), hip and spine BMD, aorta calcification (AC), radiographic vertebral fracture and self-reported wrist and hip fractures, cardiovascular events together with a wide array of demographic and lifestyle characteristics were collected. RESULTS: Presence of the ApoE epsilon 4 allele had a significant impact on serum lipid profile, yet no association with spine/hip BMD or AC could be established. In multiple regression models, apoA1 was a significant independent contributor to the variation in AC. However, none of the lipid components were independent contributors to the variation in spine or hip BMD. When comparing the women with or without vertebral fractures, serum triglycerides showed significant differences. This finding was however not applicable to hip or wrist fractures. After adjustment for age, severe AC score (>or=6) and/or manifest cardiovascular disease increased the risk of hip but not vertebral or wrist fractures. CONCLUSION: The contribution of serum lipids to the modulators of BMD does not seem to be direct but rather indirect via promotion of atherosclerosis, which in turn can affect bone metabolism locally, especially when skeletal sites supplied by end-arteries are concerned. Further studies are needed to explore the genetic or environmental risk factors underlying the association of low triglyceride levels to vertebral fractures. |
| 18651156 | Role of nitric oxide synthase gene intron 4 and exon 7 polymorphisms in obstructive sleep apnea syndrome.The objective of our study was to assess the association of eNOS4 and eNOS296 polymorphisms of endothelial nitric oxide synthase (eNOS) gene with obstructive sleep apnea syndrome (OSAS). Forty-eight patients with OSAS and 181 healthy volunteers were included in the study. Genotype analyses were performed for eNOS intron 4 VNTR and exon 7, Glu298Asp (G --> T) polymorphisms. There was no significant difference between the patients and controls regarding eNOS4 polymorphism (P > 0.05). There was a significant difference between the patients and controls regarding eNOS296 polymorphism. Glu/Asp variant was more frequent whereas Glu/Glu variant was less frequent in the patients compared to controls (P < 0.001). There was no relationship between eNOS4 and eNOS296 polymorphisms and polysomnography parameters, apnea-hypopnea index, age, gender, body weight and height, body mass index, hypertension, coronary artery disease, arrhythmia, diabetes mellitus, hypercholesterolemia and smoking (P > 0.05). The eNOS4 polymorphism of NOS gene is not associated with OSAS. However, eNOS296 polymorphism of NOS gene is associated with occurrence of OSAS, but not with severity of OSAS. |
| 20373846 | The apolipoprotein E gene and Taq1A polymorphisms in childhood obesity.Obesity is a multifactorial disease that is influenced by genetic and environmental factors. The apolipoprotein E (Apo E) polymorphism has been reported to influence some lipid profile abnormalities associated with obesity in childhood. In this study, the relationship between the Apo E gene and Taq1A polymorphisms with childhood obesity has been studied. Regarding the Apo E genotypes, e3/4 was the most frequent in both the patient and control groups. Further, there was a significance between the Apo E genotypes with low density lipoprotein and total cholesterol levels. However, no relationship was found between the Taq1A polymorphism and obesity. In conclusion, polygenic inheritance should be kept in mind when dealing with childhood obesity. |
| 20817608 | APOE and cholesterol homeostasis in Alzheimer's disease.Converging evidence from clinical and pathological studies indicate the presence of important relationships between the ongoing deterioration of brain lipid homeostasis, vascular changes and the pathophysiology of sporadic Alzheimer's disease (AD). These associations include the recognition of cholesterol transporters apolipoprotein E (APOE), APOC1 and APOJ as major genetic risk factors for common AD and observations associating risk factors for cardiovascular disease such as high midlife plasma cholesterol, diabetes, stroke, obesity and hypertension to dementia. Moreover, recent clinical findings lend support to the notion that progressive deterioration of cholesterol homeostasis in AD is a central player in the disease pathophysiology and is, therefore, a potential therapeutic target for disease prevention. |
| 21088116 | A CD36-dependent pathway enhances macrophage and adipose tissue inflammation and impairs insulin signalling.AIMS: Obesity and hyperlipidaemia are associated with insulin resistance (IR); however, the mechanisms responsible remain incompletely understood. Pro-atherogenic hyperlipidaemic states are characterized by inflammation, oxidant stress, and pathophysiologic oxidized lipids, including ligands for the scavenger receptor CD36. Here we tested the hypothesis that the absence of CD36 protects mice from IR associated with diet-induced obesity and hyperlipidaemia. METHODS AND RESULTS: Adipose tissue from CD36(-/-) mice demonstrated a less inflammatory phenotype and improved insulin signalling in vivo and at the level of the adipocyte and macrophage. The pathophysiologic ligand oxidized low-density lipoprotein (oxLDL) activated c-Jun N-terminal kinase (JNK) and disrupted insulin signalling in both adipocytes and macrophages in a CD36-dependent manner. Macrophages isolated from CD36(-/-) mice after high-fat diet feeding elicited less JNK activation and inhibition of insulin signalling in adipocytes after co-culture compared with wild-type macrophages. CONCLUSION: These data suggest that a CD36-dependent inflammatory paracrine loop between adipocytes and macrophages facilitates chronic inflammation and contributes to IR common in obesity and dyslipidaemia. |
| 22739111 | Downregulation of fetuin-B and zinc-alpha2-glycoprotein is linked to impaired fatty acid metabolism in liver cells.BACKGROUND: Our recent proteomic study has shown that plasma protein levels of fetuin-B (Ft-B) and zinc-alpha2-glycoprotein (ZAG) are significantly elevated in obesity-resistant (OR) rats exposed to a high fat diet. Time profiling of the plasma concentrations of Ft-B and ZAG in OR rats has shown stable regulation of these proteins throughout the entire period of rat breeding. METHODS: To firmly establish roles for these proteins in lipogenesis, we efficiently knocked down (KD) the genes FETUB and AZGP1 encoding Ft-B and ZAG, respectively, using siRNA in Chang liver cells. Results: Reduced expression of FETUB and AZGP1 led to a significant increase in the expression of lipogenic genes, thereby resulting in higher lipid levels in both KD cells. Collectively with our previous findings, we confirmed that Ft-B was similarly regulated with Ft-A, in that their plasma protein levels were commonly reduced in diet-induced obese rats. CONCLUSION: Our results provide a possible relationship between reduced plasma protein levels of Ft-B and ZAG and higher risk of diet-induced obesity through impaired fatty acid metabolism in hepatocytes. |
| 22921891 | Is there any association of apolipoprotein E gene polymorphism with obesity status and lipid profiles? Tehran Lipid and Glucose Study (TLGS).AIMS: Considering the key role played by the apolipoprotein E (Apo E) gene in the regulation of lipid metabolism and obesity, the current study has evaluate the association between abdominal obesity and Apo E gene polymorphism in a population of Tehran. MATERIALS AND METHODS: A cross-sectional study was performed on 345 men and 498 women, aged 19-86 years, selected from among participants of the Tehran Lipid and Glucose Study. The RFLP-PCR technique was employed to investigate polymorphism in the gene fragments. Based on the national survey of risk factors for non-communicable diseases of Iran, waist circumference (WC) cut off was set at 89 cm for men and 91 cm for women. The risk effect of obesity related variables and lipid profiles in two groups of WC were examined by logistic regression. For body mass index (BMI), waist to hip ratio (WHR), high-density lipoprotein-cholesterol (HDL-C), triglyceride (TG), fasting blood sugar (FBS), total cholesterol (TC), low-density lipoprotein-cholesterol (LDL-C), and blood pressure (BP), the standard risk cut-offs were applied. RESULTS: Frequencies of E2, E3, and E4 alleles were 9.7, 73, and 14.6%, respectively. The presence of the E3 allele was significantly associated with higher TG level in subjects with high WC, while, the presence of E4 allele decreased the plasma HDL-C (E2:52.1+/-13.1 vs., E3:48.9+/-11.2 vs., E4:44.6+/-10.6 mg/dl, p<0.05), HDL-C2 (E2:20.4+/-9.2 vs., E3:19.1+/-8.8 vs., E4:16.3+/-7.9 mg/dl, p<0.05), and HDL-C3 (E2:32.1+/-7.4 vs., E3:30.3+/-6.2 vs., E4:28.3+/-6.1 mg/dl, p<0.05) in normal WC subjects. The presence of the E3 carrier increased the risk of having higher plasma TG, compared with the E2 carrier (95% CI OR=1.91, 1.02-3.57; p=0.04). CONCLUSION: According to the results of this study, the E3 carrier, caused an approximately 90% increase in the levels of TG in the group with abdominal obesity. |
| 8813307 | Systemic and fetal-maternal nitric oxide synthesis in normal pregnancy and pre-eclampsia.OBJECTIVE: To investigate systemic and fetal-placental nitric oxide synthesis by biochemical and molecular biology means in normal human pregnancy and pre-eclampsia. DESIGN AND PARTICIPANTS: Three groups of women were studied: healthy pregnant women (n = 8), pregnant women with pre-eclampsia (n = 8), and age-matched nonpregnant controls (n = 8). Pre-eclamptic patients were treated with nifedipine (30-60 mg/day) for severe hypertension. Systemic nitric oxide synthesis was assessed in normal pregnant women at weeks 18-21, 29-32 and 38-39 and in pre-eclamptic women on admission to the hospital (29-32 weeks, 30 on average), before the morning nifedipine administration. Nonpregnant women were studied twice at four-week intervals as controls. The pattern of nitric oxide biosynthesis in fetal-placental circulation was studied in normal and pre-eclamptic women at the delivery. SETTING: Mario Negri Institute for Pharmacological Research, Bergamo, and the Division of Obstetrics and Gynaecology of the University of Brescia. MAIN OUTCOME MEASURES: Plasma cGMP levels and platelet nitric oxide synthesis, assessed by measuring the conversion of [3H]L-arginine to [3H]L-citrulline as well as intracellular cGMP, were evaluated. Constitutive nitric oxide synthase (EC-NOS) gene expression by Northern blot analysis and nitric oxide release by the conversion of [3H]L-arginine to [3H]L-citrulline were assessed in umbilical vein endothelial cells (HUVEC) and in placenta. Inducible nitric oxide synthase activity was also evaluated in HUVEC exposed to tumour necrosis factor alpha (TNF alpha) and in placenta homogenates incubated in calcium free medium. RESULTS: Plasma cGMP was higher in both normal pregnant and pre-eclamptic women than in nonpregnant controls. In normal pregnancy cGMP rose as early as 18-21 weeks and remained elevated throughout pregnancy. [3H]L-citrulline production and intracellular cGMP were comparable in platelets from all women. EC-NOS gene expression and nitric oxide synthesis were identical in HUVEC and placenta from normal pregnant and pre-eclamptic women. CONCLUSIONS: Systemic levels of CGMP, the nitric oxide second messenger, are increased in normal pregnancy. Excessive nitric oxide production does not derive from platelets. Pre-eclampsia is not associated with changes in fetal-placental nitric oxide synthesis. |
| 11955026 | High risk for hyperlipidemia and the metabolic syndrome after an episode of hypertriglyceridemia during 13-cis retinoic acid therapy for acne: a pharmacogenetic study.BACKGROUND: Administration of 13-cis retinoic acid (isotretinoin) for acne is occasionally accompanied by hyperlipidemia. It is not known why some persons develop this side effect. OBJECTIVE: To determine whether isotretinoin triggers a familial susceptibility to hyperlipidemia and the metabolic syndrome. DESIGN: Cross-sectional comparison. SETTING: University hospital in Lausanne, Switzerland. PARTICIPANTS: 102 persons in whom triglyceride levels increased at least 1.0 mmol/L (> or =89 mg/dL) (hyperresponders) and 100 persons in whom triglyceride levels changed 0.1 mmol/L (< or =9 mg/dL) or less (nonresponders) during isotretinoin therapy for acne. Parents of 71 hyperresponders and 60 nonresponders were also evaluated. MEASUREMENTS: Waist-to-hip ratio; fasting glucose, insulin, and lipid levels; and apoE genotype. RESULTS: Hyperresponders and nonresponders had similar pretreatment body weight and plasma lipid levels. When reevaluated approximately 4 years after completion of isotretinoin therapy, hyperresponders were more likely to have hypertriglyceridemia (triglyceride level > 2.0 mmol/L [>177 mg/dL]; odds ratio [OR], 4.8 [95% CI, 1.6 to 13.8]), hypercholesterolemia (cholesterol level > 6.5 mmol/L [>252 mg/dL]; OR, 9.1 [CI, 1.9 to 43]), truncal obesity (waist-to-hip ratio > 0.90 [OR, 11.0 (CI, 2.0 to 59]), and hyperinsulinemia (insulin-glucose ratio > 7.2; OR, 3.0 [CI, 1.6 to 5.7]). In addition, more hyperresponders had at least one parent with hypertriglyceridemia (OR, 2.6 [CI, 1.2 to 5.7]) or a ratio of total to high-density lipoprotein cholesterol that exceeded 4.0 (OR, 3.5 [CI, 1.5 to 8.0]). Lipid response to isotretinoin was closely associated with the apoE gene. CONCLUSION: Persons who develop hypertriglyceridemia during isotretinoin therapy for acne, as well as their parents, are at increased risk for future hyperlipidemia and the metabolic syndrome. |
| 12185856 | Analysis of the association between apolipoprotein E polymorphism and cardiovascular risk factors in an elderly population with longevity.OBJECTIVE: To establish the allelic and genotypic frequencies related to apolipoprotein E (ApoE) polymorphism and association of the genotypes with risk factors and cardiovascular morbidity in an elderly population with longevity. METHODS: We analyzed 70 elderly patients aged 80 years or more who were part of the Projeto Veranopolis. We used the gene amplification technique through the polymerase chain reaction-restriction fragment length polymorphism (PCR-RFLP) and cleavage with the restriction enzyme Hha I to identify the ApoE genotypes. The most frequent genotypes were compared considering biological variables and cardiovascular risks and morbidity. RESULTS: The frequencies of the E2, E3, and E4 alleles were 0.05, 0.84, and 0.11, respectively, and of the genotypes were as follows: E3E3 (0.70), E3E4 (0.22), E2E3 (0.06), and E2E2 (0.02). Individuals with the E3E4 had a mean age greater than those with the E3E3. No association was observed between the genotypes and the variables analyzed, except for obesity, which was associated with the E3E3 genotype. Individuals with the E3E4 genotype had high levels of LDL-cholesterol and fibrinogen as compared with those with the E3E3 genotype. CONCLUSION: The results suggest that the E4E4 genotype may be associated with early mortality. A balance between the protective or neutral factors and the cardiovascular risk factors may occur among the individuals with different genotypes, attenuating the negative effects of the E4 allele. |
| 15913838 | Expression of nitric oxide synthase isoforms in hypothalamo-pituitary-adrenal axis during the development of spontaneous hypertension in rats.This study was performed to investigate the expression of the major isoforms of nitric oxide synthase mRNA and protein in the hypothalamo-pituitary-adrenal axis (HPA axis) of spontaneously hypertensive rats (SHR) at two different postnatal ages corresponding to the development of genetic hypertension. Using RT-PCR and Western blot techniques, the mRNA and protein levels of neuronal (nNOS), endothelial (eNOS) and inducible (iNOS) isoforms were measured in 3- to 4-week-old (prehypertensive phase) and 12- to 13-week-old (established hypertension phase) SHR and age-matched normotensive Wistar-Kyoto (WKY) rats. nNOS but not eNOS mRNA levels were increased at prehypertensive and hypertensive phases in SHR HPA axis. Compared to age-matched WKY rats, significantly higher levels of nNOS protein were found in the hypothalamus, lower levels in the adrenal glands and no changes were observed in the pituitary gland. At both ages tested, there was no significant change in eNOS protein expression in SHR HPA axis. The expression of iNOS mRNA and protein was under detection limit. In the HPA axis, the expression of nNOS isoform appears to be differentially controlled at the transcriptional and translational levels in SHR. Increased mRNA levels and differential nNOS protein expression from birth in SHR HPA axis may contribute in the pathogenesis of genetic hypertension. |
| 19368146 | Platelet GP IIIA polymorphism HPA-1 (PLA1/2) is associated with hypertension as the primary cause for end-stage renal disease in hemodialysis patients from Greece.Human platelets carry membrane glycoproteins that control platelet aggregation and activation. A number of clinical studies have suggested that certain polymorphisms of genes encoding these proteins increase the risk for cardiovascular disease. The frequency of gene polymorphisms for the four most common platelet glycoproteins (HPA 1, 2, 3 and 5) was examined and correlated with the primary cause of end-stage renal disease (ESRD) in Greek patients on HD. Fifty-five (55) patients on chronic maintenance haemodialysis (HD) (22 female, 33 male), aged from 23- to 87-years-old, (mean age 66 years), being on dialysis for 53 +/- 34 months, were included in the study. HPA-1, -2, -3, and -5 genotyping was performed using polymerase chain reaction (PCR) amplification with sequence-specific primers (PCR-SSP). Calculated relative frequencies of the alleles were as follows: HPA-1a/b 0.81/0.19, HPA-2a/b 0.92/0.08, HPA-3a/b 0.62/0.38 and HPA-5a/b 0.93/0.07. There was a statistically significant association between the HPA-1b allele and hypertension as the primary cause of ESRD (65% of patients with hypertension vs 23% of all other patients carried the HPA-1b allele, p=0.02, Fisher's exact test). The results suggest that Greek carriers of the HPA-1b allele with hypertension may be at increased risk for developing end-stage renal disease. |
| 20397260 | Role of HSP-90 for increased nNOS-mediated vasodilation in mesenteric arteries in portal hypertension.AIM: To explore the role of heat shock protein-90 (HSP-90) for nitrergic vasorelaxation in the splanchnic circulation in rats with and without portal hypertension. METHODS: Neuronal nitric oxide synthase (nNOS) and HSP-90 were analyzed by immunofluorescence, western blotting and co-immunoprecipitation in the mesenteric vasculature and isolated nerves of portal-vein-ligated (PVL) rats and sham operated rats. In vitro perfused de-endothelialized mesenteric arterial vasculature was preconstricted with norepinephrine (EC(80)) and tested for nNOS-mediated vasorelaxation by periarterial nerve stimulation (PNS, 2-12 Hz, 45V) before and after incubation with geldanamycin (specific inhibitor of HSP-90 signalling, 3 microg/mL) or L-NAME (non-specific NOS-blocker, 10(-4) mol/L). RESULTS: nNOS and HSP-90 expression was significantly increased in mesenteric nerves from PVL as compared to sham rats. Moreover, nNOS and HSP-90 were visualized in mesenteric nerves by immunofluorescence and immunoprecipitation of nNOS co-immunoprecipitated HSP-90 in sham and PVL rats. PNS induced a frequency-dependent vasorelaxation which was more pronounced in PVL as compared to sham rats. L-NAME and geldanamycin markedly reduced nNOS-mediated vasorelaxation abrogating differences between the study groups. The effect of L-NAME and geldanamycin on nNOS-mediated vasorelaxation was significantly greater in PVL than in sham animals. However, no difference in magnitude of effect between L-NAME and geldanamycin was noted. CONCLUSION: HSP-90 acts as a signalling mediator of nNOS-dependent nerve mediated vascular responses in mesenteric arteries, and the increased nitrergic vasorelaxation observed in portal hypertension is mediated largely by HSP-90. |
| 22035022 | Effect Of G2706A and G1051A polymorphisms of the ABCA1 gene on the lipid, oxidative stress and homocystein levels in Turkish patients with polycystic ovary syndrome.BACKGROUND: Obesity, insulin resistance and hyperandrogenism, crucial parameters of Polycystic ovary syndrome (PCOS) play significant pathophysiological roles in lipidemic aberrations associated within the syndrome. Parts of the metabolic syndrome (low HDL and insulin resistance) appeared to facilitate the association between PCOS and coronary artery disease, independently of obesity. ABCA1 gene polymorphism may be altered this components in PCOS patients.In this study, we studied 98 PCOS patients and 93 healthy controls. All subjects underwent venous blood drawing for complete hormonal assays, lipid profile, glucose, insulin, malondialdehyde, nitric oxide, disulfide levels and ABCA genetic study. RESULTS: In PCOS group fasting glucose, DHEAS, 17-OHP, free testosterone, total-cholesterol, triglyceride, LDL-cholesterol and fibrinogen were significantly different compare to controls. The genotype ABCA G2706A distribution differed between the control group (GG 60.7%, GA 32.1%, AA 7.1%) and the PCOS patients (GG 8.7%, GA 8.7%, AA 76.8%). The frequency of the A allele (ABCAG2706A) was higher in PCOS patients than control group with 13,0% and 23,2%, respectively. In this study, the homocystein and insulin levels were significantly higher in PCOS patients with ABCA G1051A mutant genotype than those with heterozygote and wild genotypes. CONCLUSIONS: We found higher percentage of AA genotype and A allele of ABCA G2706A in PCOS patients compare to controls. The fasting insulin and homocystein levels were significantly higher in PCOS patients with ABCA G1051A mutant genotype than those with heterozygote and wild genotypes. |
| 23125252 | IL-13 receptor alpha2-arginase 2 pathway mediates IL-13-induced pulmonary hypertension.Although previous literature suggests that interleukin (IL)-13, a T-helper type 2 cell effector cytokine, might be involved in the pathogenesis of pulmonary hypertension (PH), direct proof is lacking. Furthermore, a potential mechanism underlying IL-13-induced PH has never been explored. This study's goal was to investigate the role and mechanism of IL-13 in the pathogenesis of PH. Lung-specific IL-13-overexpressing transgenic (Tg) mice were examined for hemodynamic changes and pulmonary vascular remodeling. IL-13 Tg mice spontaneously developed PH phenotype by the age of 2 mo with increased expression and activity of arginase 2 (Arg2). The role of Arg2 in the development of IL-13-stimulated PH was further investigated using Arg2 and IL-13 receptor alpha2 (Ralpha2) null mutant mice and the small-interfering RNA (siRNA)-silencing approach in vivo and in vitro, respectively. IL-13-stimulated medial thickening of pulmonary arteries and right ventricle systolic pressure were significantly decreased in the IL-13 Tg mice with Arg2 null mutation. On the other hand, the production of nitric oxide was further increased in the lungs of these mice. In our in vitro evaluations, the recombinant IL-13 treatment significantly enhanced the proliferation of human pulmonary artery smooth muscle cells in an Arg2-dependent manner. The IL-13-stimulated cellular proliferation and the expression of Arg2 in hpaSMC were markedly decreased with IL-13Ralpha2 siRNA silencing. Our studies demonstrate that IL-13 contributes to the development of PH via an IL-13Ralpha2-Arg2-dependent pathway. The intervention of this pathway could be a potential therapeutic target in pulmonary arterial hypertension. |
| 11398147 | Apolipoprotein E polymorphism modulates the association between obesity and dyslipidemias during young adulthood: The Bogalusa Heart Study.To elucidate to what extent apolipoprotein (apo) E polymorphism modulates obesity-induced dyslipidemias during young adulthood, longitudinal data on 759 individuals (72% white/28% black; initial and follow-up mean age, 25.9 and 32.7 years) were examined. Among both races and the total sample, the apo E2 group (with E2/2 or E2/3 phenotype) had significantly lower and the apo E4 (with E4/4 or E3/4 phenotype) group higher low-density lipoprotein (LDL) cholesterol than the apo E3 (with E3/3 phenotype) group at both examinations. In addition, the apo E2 group displayed higher high-density lipoprotein (HDL) cholesterol in the total sample. No allele-specific effect was noted for the longitudinal changes (Delta). An increase in Delta adiposity, measured as Delta body mass index (BMI), was accompanied by higher increase in Delta LDL cholesterol in the e4 carriers than the e2 carriers among the whites (P <.05) and the total sample (P <.01); an increase in Delta triglycerides and decrease in Delta HDL cholesterol in the e2 carriers than the e4 carriers among all the groups (P <.05 to.001). Among the apo E phenotype groups, the incidence of high (>75th percentile specific for race and sex) LDL cholesterol at follow-up was in the order E4 > E3 > E2 both in the obese (BMI > 30; P for trend =.033) and the nonobese (BMI < 25; P for trend =.035) groups. Although the increase of low (<25th percentile specific for race and sex) HDL cholesterol or high triglycerides showed no apo E phenotype-specific trend, the incidence of high triglycerides without high LDL cholesterol was in the order E2 > E3 > E4 only in the obese group (P for trend =.025). The prevalence trend for dyslipidemias at follow-up among the persistently obese and nonobese groups also gave similar results. Thus, apo E gene locus influences not only the levels of certain lipoprotein variables during young adulthood, but also modulates the association between obesity and dyslipidemias. |
| 12086099 | Apolipoprotein E genotype, lipid levels and coronary heart disease in a Polish population group.Genotype of apolipoprotein E has been identified in a group of randomly selected Polish subjects participating in a cross-sectional study performed within the POL-MONICA Program, the part of international study WHO-MONICA Project. The investigated group consisted of 170 persons, 92 males and 78 females aged 41-69 years (mean age 62.0+/-5.11). The observed frequency of apolipoprotein E alleles was: epsilon2 - 7.6%, epsilon3 - 81.8% and epsilon4 - 10.6%, which was similar to frequencies in the neighbouring European countries. Statistically significant lower means of total cholesterol (TC) and of low density lipoprotein cholesterol (LDL-C) levels in epsilon2 carriers and higher means of TC, of LDL-C and of triglycerides in epsilon4 carriers were observed as compared with noncarriers of respective alleles. Some nonlipid cardiovascular risk factors (hypertension (HT) and obesity) and coronary heart disease (CHD) showed a tendency to lower prevalence in the epsilon2 allele carriers as compared to noncarriers. In the epsilon4 allele carriers a tendency to higher prevalence of HT, but not of CHD was observed as compared to noncarriers of this allele. |
| 15990946 | Functional characterization of brain mitochondrial nitric oxide synthase during hypertension and aging.Nitric oxide (NO\*) plays an important role in various physiological processes. The aim of the present study was to investigate if brain mitochondrial nitric oxide synthase (mtNOS) is active and functional during hypertension. L-citrulline production, an indicator of nitric oxide synthesis, was concentration-dependent on L-arginine in all strains and all ages tested, and was inhibited by 7-Nitroindazole (7-NI). Brain mitochondria of 1 month-old (prehypertensive) spontaneously hypertensive rats (SHR) exhibited a significantly (p < 0.05) low basal L-citrulline content as compared to age-matched Wistar (W) and Wistar-Kyoto (WKY) rats. L-citrulline synthesis in SHR rats showed a significant (p < 0.01) low response to L-arginine in 3 and 7 months-old rats. Respiratory rates in states 3 and 4 increased with low L-arginine concentration in all strains and all ages. The results suggest that in rat brain mitochondria, L-citrulline synthesis is constant once age-related hypertension is installed and NO\* does not regulate oxidative phosphorylation. |
| 16606673 | Antioxidants protect from atherosclerosis by a heme oxygenase-1 pathway that is independent of free radical scavenging.Oxidative stress is implicated in atherogenesis, yet most clinical trials with antioxidants, particularly vitamin E, have failed to protect against atherosclerotic diseases. A striking exception is probucol, which retards atherosclerosis in carotid arteries and restenosis of coronary arteries after angioplasty. Because probucol has in vitro cellular-protective effects independent of inhibiting lipid oxidation, we investigated the mode of action of probucol in vivo. We used three models of vascular disease: apolipoprotein E-deficient mice, a model of atherosclerosis; rabbit aortic balloon injury, a model of restenosis; and carotid injury in obese Zucker rats, a model of type 2 diabetes. Unexpectedly, we observed that the phenol moieties of probucol were insufficient, whereas its sulphur atoms were required for protection. Probucol and its sulphur-containing metabolite, but not a sulphur-free phenolic analogue, protected via cell-specific effects on inhibiting macrophage accumulation, stimulating reendothelialization, and inhibiting vascular smooth muscle cell proliferation. These processes were mediated via induction of heme oxygenase-1 (HO-1), an activity not shared by vitamin E. Our findings identify HO-1 as the molecular target of probucol. They indicate 2-electron rather than radical (1-electron) oxidants as important contributors to atherogenesis, and point to novel lead compounds for therapeutic intervention against atherosclerotic diseases. |
| 17170237 | Glucose, insulin, and leptin signaling pathways modulate nitric oxide synthesis in glucose-inhibited neurons in the ventromedial hypothalamus.Glucose-sensing neurons in the ventromedial hypothalamus (VMH) are involved in the regulation of glucose homeostasis. Glucose-sensing neurons alter their action potential frequency in response to physiological changes in extracellular glucose, insulin, and leptin. Glucose-excited neurons decrease, whereas glucose-inhibited (GI) neurons increase, their action potential frequency when extracellular glucose is reduced. Central nitric oxide (NO) synthesis is regulated by changes in local fuel availability, as well as insulin and leptin. NO is involved in the regulation of food intake and is altered in obesity and diabetes. Thus this study tests the hypothesis that NO synthesis is a site of convergence for glucose, leptin, and insulin signaling in VMH glucose-sensing neurons. With the use of the NO-sensitive dye 4-amino-5-methylamino-2',7'-difluorofluorescein in conjunction with the membrane potential-sensitive dye fluorometric imaging plate reader, we found that glucose and leptin suppress, whereas insulin stimulates neuronal nitric oxide synthase (nNOS)-dependent NO production in cultured VMH GI neurons. The effects of glucose and leptin were mediated by suppression of AMP-activated protein kinase (AMPK). The AMPK activator 5-aminoimidazole-4-carboxamide-1-beta-4-ribofuranoside (AICAR) increased both NO production and neuronal activity in GI neurons. In contrast, the effects of insulin on NO production were blocked by the phosphoinositide 3-kinase inhibitors wortmannin and LY-294002. Furthermore, decreased glucose, insulin, and AICAR increase the phosphorylation of VMH nNOS, whereas leptin decreases it. Finally, VMH neurons express soluble guanylyl cyclase, a downstream mediator of NO signaling. Thus NO may mediate, in part, glucose, leptin, and insulin signaling in VMH glucose-sensing neurons. |
| 17611635 | Detection of proteolytic cleavages of diabetes-associated protein IA-2 beta in the pancreas and the brain using novel anti-IA-2 beta monoclonal antibodies.Insulinoma-associated protein (IA)-2 beta, an inactive member of the protein-tyrosine phosphatase (PTP) family, is a major autoantigen in type-1 diabetes mellitus. IA-2 beta exists mainly in a 60-kDa form, and is frequently located in the dense-core secretory vesicles of pancreatic beta cells. As IA-2 beta gene-deficient mice exhibit impaired insulin secretions, IA-2 beta is probably involved in insulin secretions. In the present study, we characterized the major forms of IA-2 beta in the brain and pancreas of normal and non-obese diabetic (NOD) mice. Novel monoclonal antibodies (mAbs) against IA-2 beta revealed that this brain protein was of multiple compositions incorporating the 60-, 64-, 67- and 71-kDa forms, which were designated as IA-2 beta 60, IA-2 beta 64, IA-2 beta 67 and IA-2 beta 71, respectively. On the contrary, only the 60-kDa isoform of IA-2 beta was expressed in the mouse pancreas and in the mouse pancreatic beta cell line, MIN6. Sequence analyses revealed that IA-2 beta 60, IA-2 beta 64 and IA-2 beta 71 (brain-derived immunoprecipitated IA-2 beta isoforms) contained alternative NH2- termini starting from Glu489, Ala464, and Ser414, respectively, while IA-2 beta 60 (an MIN6-derived immunoprecipitated IA-2 beta isoform) contained those from Glu489. Consistent with the lack of an NH2-terminal region of IA-2 beta, the isoforms were recognized by their respective mAbs characterized with different epitope regions. Furthermore, Western blotting and immunohistochemistry demonstrated that NOD mice expressed similar isoforms present in the brains and pancreatic islets of C57BL/6J, BALC/CA and ICR mice, accordingly. Taken together, these results suggest that IA-2 beta undergoes at least three distinct proteolytic cleavages. |
| 18057060 | Fibrinogen Aalpha Thr312Ala polymorphism is associated with chronic thromboembolic pulmonary hypertension.Although chronic thromboembolic pulmonary hypertension (CTEPH) is characterised by the persistence of organised thrombus, few pro-thrombotic risk factors have been identified in subjects with the disease. The aim of the present study was to compare the prevalence of eight functionally relevant haemostatic polymorphisms between CTEPH subjects and healthy controls. Genomic DNA was isolated from 214 CTEPH subjects and 200 healthy controls, and analysed for Factor V Leiden, prothrombin guanine (G) to adenine (A) substitution at nucleotide 20210 (20210G>A), plasminogen activator inhibitor-1 4G/5G, tissue plasminogen activator 7351 cytosine (C)>thymidine (T), Factor XIII 100G>T, fibrinogen Aalpha substitution of threonine with alanine at position 312 (Thr312Ala), fibrinogen Bbeta substitution of arginine with lysine at position 448 (Arg448Lys) and fibrinogen Bbeta 455G>A polymorphisms. A significant difference was demonstrated in fibrinogen Aalpha Thr312Ala genotype and allele frequencies between CTEPH subjects and controls. The presence of the alanine allele significantly increased the risk of CTEPH. The fibrinogen Aalpha alanine 312 allele alters fibrinogen alpha-alpha chain cross-linkage and has previously been associated with both increased risk of embolisation and increased resistance to thrombolysis. An association between this polymorphism and chronic thromboembolic pulmonary hypertension, therefore, supports an embolic aetiology for this disease, and may provide a mechanism by which thrombus persists following an acute event. |
| 18298402 | Inhibition of the interaction between protein phosphatase 1 glycogen-targeting subunit and glycogen phosphorylase increases glycogen synthesis in primary rat hepatocytes.In Type 2 diabetes, increased glycogenolysis contributes to the hyperglycaemic state, therefore the inhibition of GP (glycogen phosphorylase), a key glycogenolytic enzyme, is one of the possibilities to lower plasma glucose levels. Following this strategy, a number of GPis (GP inhibitors) have been described. However, certain critical issues are associated with their mode of action, e.g. an impairment of muscle function. The interaction between GP and the liver glycogen targeting subunit (termed G(L)) of PP1 (protein phosphatase 1) has emerged as a new potential anti-diabetic target, as the disruption of this interaction should increase glycogen synthesis, potentially providing an alternative approach to counteract the enhanced glycogenolysis without inhibiting GP activity. We identified an inhibitor of the G(L)-GP interaction (termed G(L)-GPi) and characterized its mechanism of action in comparison with direct GPis. In primary rat hepatocytes, at elevated glucose levels, the G(L)-GPi increased glycogen synthesis similarly to direct GPis. Direct GPis significantly reduced the cellular GP activity, caused a dephosphorylation of the enzyme and decreased the amounts of GP in the glycogen-enriched fraction; the G(L)-GPi did not influence any of these parameters. Both mechanisms increased glycogen accumulation at elevated glucose levels. However, at low glucose levels, only direct GPis led to increased glycogen amounts, whereas the G(L)-GPi allowed the mobilization of glycogen because it did not block the activity of GP. Due to this characteristic, G(L)-GPi in comparison with GPis could offer an advantageous risk/benefit profile circumventing the potential downsides of a complete prevention of glycogen breakdown while retaining glucose-lowering efficacy, suggesting that inhibition of the G(L)-GP interaction may provide an attractive novel approach for rebalancing the disturbed glycogen metabolism in diabetic patients. |
| 18349115 | A moderate increase in carnitine palmitoyltransferase 1a activity is sufficient to substantially reduce hepatic triglyceride levels.Nonalcoholic fatty liver disease (NAFLD), hypertriglyceridemia, and elevated free fatty acids are present in the majority of patients with metabolic syndrome and type 2 diabetes mellitus and are strongly associated with hepatic insulin resistance. In the current study, we tested the hypothesis that an increased rate of fatty acid oxidation in liver would prevent the potentially harmful effects of fatty acid elevation, including hepatic triglyceride (TG) accumulation and elevated TG secretion. Primary rat hepatocytes were transduced with adenovirus encoding carnitine palmitoyltransferase 1a (Adv-CPT-1a) or control adenoviruses encoding either beta-galactosidase (Adv-beta-gal) or carnitine palmitoyltransferase 2 (Adv-CPT-2). Overexpression of CPT-1a increased the rate of beta-oxidation and ketogenesis by approximately 70%, whereas esterification of exogenous fatty acids and de novo lipogenesis were unchanged. Importantly, CPT-1a overexpression was accompanied by a 35% reduction in TG accumulation and a 60% decrease in TG secretion by hepatocytes. There were no changes in secretion of apolipoprotein B (apoB), suggesting the synthesis of smaller, less atherogenic VLDL particles. To evaluate the effect of increasing hepatic CPT-1a activity in vivo, we injected lean or obese male rats with Adv-CPT-1a, Adv-beta-gal, or Adv-CPT-2. Hepatic CPT-1a activity was increased by approximately 46%, and the rate of fatty acid oxidation was increased by approximately 44% in lean and approximately 36% in obese CPT-1a-overexpressing animals compared with Adv-CPT-2- or Adv-beta-gal-treated rats. Similar to observations in vitro, liver TG content was reduced by approximately 37% (lean) and approximately 69% (obese) by this in vivo intervention. We conclude that a moderate stimulation of fatty acid oxidation achieved by an increase in CPT-1a activity is sufficient to substantially reduce hepatic TG accumulation both in vitro and in vivo. Therefore, interventions that increase CPT-1a activity could have potential benefits in the treatment of NAFLD. |
| 20019774 | [High-risk factors and clinical pathological analysis in 21 cases of fatal pulmonary embolism].OBJECTIVE: To explore the clinical and pathological characteristics of fatal pulmonary thromboembolism, especially the contribution of acquired and genetic risk factors of fatal pulmonary thromboembolism, so as to provide some useful information for its clinical prevention and treatment. METHODS: The medical dispute autopsy cases performed at the Department of Pathology, School of Basic Medical Sciences, Peking University from January 1, 2002 to September 30, 2008 were retrospectively reviewed. FV ( factor V ) Leiden mutation and FII ( factor II) G20210A mutation were analyzed by using methods of PCR and restriction fragment length polymorphism (RFLP) in fatal pulmonary embolism cases and random selected control cases of adult autopsy. RESULTS: There were 21 patients who died from fatal pulmonary thromboembolism, accounting for 12.96% (21/162) of adult autopsy cases. In these cases, there was at least one of the known risk factors in each case and more than two risk factors could be found in most cases. The acquired risk factors, such as surgery, trauma and fractures, cancer, pregnancy and puerperium, the elderly, hypertension, diabetes, infections, could be found in these patients. FV Leiden mutation and FII G20210A mutation were not detected in any of the 21 cases of fatal pulmonary thromboembolism cases and six control cases. Of all the 21 cases, quantitative D-dimer measurement was performed only in six patients and D-dime level in these six samples was all significantly increased. According to the records, there was only 1 patient treated by preventive anticoagulants in the 21 patients. CONCLUSION: Fatal pulmonary thromboembolism is an important reason for medical dispute. Fractures and trauma, surgery and other acquired factors are the main reasons for fatal pulmonary embolism in Chinese. Genetic factors (FV Leiden and FII G20210A mutations) are not the reason for Chinese to suffer from fatal pulmonary thromboembolism. Assessment of risk factors (especially traumatic fractures and surgical intervention) of pulmonary thromboembolism may play an important role in taking appropriate preventive procedures, early diagnosis and reasonable treatment to reduce its mortality. |
| 20619835 | Haemostatic alterations in overweight children: associations between metabolic syndrome, thrombin generation, and fibrinogen levels.BACKGROUND: The metabolic syndrome (MetS) is associated with central obesity and leads to increased morbidity and mortality due to cardiovascular disease (CVD). Since obesity is associated with a hypercoagulable state, it has been speculated that hypercoagulation is linking MetS to CVD. METHODS: We prospectively examined 81 overweight children and 32 normal-weight children aged 10-16 years. We analyzed blood pressure, fasting lipids, glucose, insulin, fibrinogen, and thrombin generating test determining time to peak (TTPeak), peak, time preceding the thrombin burst (lag-time), and 'endogenous' thrombin potential (ETP). RESULTS: Overweight children demonstrated significantly higher fibrinogen levels (p<0.001), shorter lag-time (p<0.001), and TTPeak (p=0.038) compared to normal-weight children. Furthermore, ETP (p<0.001) and peak (p<0.001) were significantly higher in overweight than in normal-weight children. Fibrinogen and all parameters of the clotting test correlated significantly (p always <0.05) to body mass index (BMI) but not significantly to insulin resistance index HOMA-IR or occurrence of MetS in multiple linear backward regression analyses adjusted for age and gender. CONCLUSIONS: The increased fibrinogen levels and the changes in the thrombin generation test points towards a haemostatic alteration in overweight children. The parameters of the clotting test were related to the degree of overweight but not to insulin resistance or occurrence of MetS questioning a direct association between MetS and the coagulation system. Longitudinal studies are needed to confirm these findings. |
| 21276794 | Cyclophilin D deficiency prevents diet-induced obesity in mice.Mitochondrial coupling efficiency is pivotal in thermogenesis and energy homeostasis. Here we show that deletion of cyclophilin D (CypD), a key modulator of the mitochondrial permeability transition pore, demonstrated resistance to diet-induced obesity (DIO) in both male and female mice, due to increased basal metabolic rate, heat production, total energy expenditure and expenditure of fat energy, despite increased food consumption. Absorption of fatty acids is not altered between CypD(-/-) and wild-type mice. Adult CypD(-/-) developed hyperglycemia, insulin resistance and glucose intolerance albeit resistant to DIO. These data demonstrate that inhibition of CypD function could protect from HFD-IO by increasing energy expenditure in both male and female mice. Inhibition of CypD may offer a novel target to modulate metabolism. |
| 11383910 | An assessment of genetic markers as predictors of bone turnover in healthy adults.In 1992 a significant relationship between bone turnover and the vitamin D receptor (VDR) genotype was reported in Australian subjects of UK-Irish decent. Since then, several groups have investigated the relationship between VDR and other bone-related genotypes, bone mass and bone turnover in several populations. However, the results of these studies are conflicting. Therefore, our aim was to determine bone-related genotypes in a population of healthy Irish adults and relate these genotypes to the rate of bone turnover. One hundred and eighteen healthy Irish adults (aged 19-67 yr) were recruited and fasting blood and first void urines were collected from each subject. Bone-related genotype frequencies in healthy Irish adults were similar to those reported in other Caucasian populations and were in Hardy-Weinberg equilibrium. Estrogen receptor (Pvu II or Xba I), apolipoprotein E and collagen IA1 genotypes were not related to bone turnover. The tt VDR genotype was associated with significantly higher serum osteocalcin (29% and 40%) compared with the Tt and TT genotypes, respectively. The ff VDR genotype was associated with significantly higher urinary pyridinoline (by approximately 44% and approximately 29%) and deoxypyridinoline (by approximately 76% and approximately 58%) levels and higher serum osteocalcin (by approximately 25% and approximately 53%) compared with the Ff or FF genotypes, respectively. These findings suggest that healthy Irish adults with either the tt or ff VDR genotype have higher rates of bone turnover than those with Tt or TT, or Ff or FF genotypes, respectively, and therefore may have a higher risk of low bone mineral density and osteoporosis in later life. |
| 15795809 | The aerobic fitness (VO2 peak) and alpha-fibrinogen genetic polymorphism in obese and non-obese Chinese boys.The purpose of the study was to compare the aerobic fitness (VO (2) peak) between obese and non-obese boys at pre-puberty and examine the effect of body composition on VO (2) peak in this cohort with reference to TaqI polymorphism at alpha-fibrinogen gene locus. Seventy-seven Chinese boys with similar lifestyle participated in the study. Among them, 47 were diagnosed as obese. VO (2) peak was measured by a treadmill test and body composition was assessed via a combined anthropometrical and bioelectrical impedance analysis method. The alpha-fibrinogen genetic polymorphism was detected through PCR-based digestion with TaqI restriction enzyme. The results indicated that VO (2) peak was significantly lower in obese boys compared with normal weight counterparts when the data were expressed either in conventional ratio unit (ml (-1) . min (-1) . lean body weight [LBW] (-1)) or in allometric unit (ml (-1) . min (-1) . body weight [BW] (-2/3)). LBW, fat mass (FM), and body fat content (BF %) all were correlated with VO (2) peak, while LBW was the strongest predictor. The relationship between body composition and VO (2) peak seemed quite comparable across different alpha-fibrinogen genotypes. Significant difference was observed between obese and non-obese boys in terms of the proportion of genotypes and frequency of alleles. T1T1 homozygotes had higher risk for obesity. We came to the conclusion that prepubertal obese boys exhibited impaired aerobic fitness compared with their normal weight peers. VO (2) peak is closely related to LBW and independent of FM. This relationship remains constant irrespective of the TaqI alpha-fibrinogen genotypes that may be associated with fatness in boys. |
| 15886801 | Lipoprotein (a) and other prothrombotic risk factors in Caucasian women with unexplained recurrent miscarriage. Results of a multicentre case-control study.From 1998 to 2003, 133 Caucasian women aged 17-40 years (median 29 years) suffering from unexplained recurrent miscarriage (uRM) were consecutively enrolled. In patients and 133 age-matched healthy controls prothrombotic risk factors (factor V (FV) G1691A, factor II (FII) G20210A, MTHFR T677T, 4G/5G plasminogen activator inhibitor (PAI)-1, lipoprotein (Lp) (a), protein C (PC), protein S (PS), antithrombin (AT), antiphospholipid/anticardiolipin (APA/ACA) antibodies) as well as associated environmental conditions (smoking and obesity) were investigated. 70 (52.6%) of the patients had at least one prothrombotic risk factor compared with 26 control women (19.5%; p<0.0001). Body mass index (BMI; p=0.78) and smoking habits (p=0.44) did not differ significantly between the groups investigated. Upon univariate analysis the heterozygous FV mutation, Lp(a) > 30 mg/dL, increased APA/ACA and BMI > 25 kg/m(2) in combination with a prothrombotic risk factor were found to be significantly associated with uRM. In multivariate analysis, increased Lp(a) (odds ratio (OR): 4.7/95% confidence interval (CI): 2.0-10.7), the FV mutation (OR:3.8/CI:1.4-10.7), and increased APA/ACA (OR: 4.5/CI: 1.1-17.7) had independent associations with uRM. |
| 18356838 | Cord blood ASP is predicted by maternal lipids and correlates with fetal birth weight.BACKGROUND: The acylation stimulating protein (ASP) is a potent lipogenic adipokine that correlates with postprandial triglyceride (TG) clearance and is linked to the pathophysiology of obesity and related disorders. OBJECTIVE: To investigate ASP levels in cord blood and its relation to maternal and cord blood lipid parameters and fetal birth weight. METHODS AND PROCEDURES: Thirty nondiabetic pregnant women, their newborns, and thirty-three nonpregnant controls were included in this study. Fasting maternal and cord blood ASP, TGs, nonesterified fatty acids (NEFAs), cholesterol, glucose levels, in addition to maternal BMI and fetal birth weight were measured. RESULTS: No significant difference was found between cord blood ASP (16.3 +/- 0.96 nmol/l) and ASP levels in the adult controls (15.7 +/- 1.0 nmol/l). Cord blood ASP, however, was lower than maternal plasma ASP levels (25.4 +/- 1.6 nmol/l, P < 0.001). Yet, lipid levels in cord blood, particularly TGs were markedly decreased compared to control and maternal TG levels (threefold and 7.4-fold, P < 0.001 respectively). Maternal TGs significantly correlated with fetal birth weight (r = 0.54, P = 0.002). Multiple regression analysis showed that maternal TGs (beta = 0.57, P = 0.01) and NEFAs (beta = 0.43, P = 0.024) predicted 45% variation in cord blood ASP levels, independent of all measured maternal and cord blood parameters. Cord blood ASP showed a positive correlation with fetal birth weight (r = 0.524, P = 0.037) in neonates above average fetal birth weight of the studied population. DISCUSSION: This is the first study investigating ASP in cord blood. We suggest that maternal hypertriglyceridemia is associated with increased fetal ASP production, thus enhancing fetal fat storage independent of maternal glucose variations in nondiabetic women. |
| 18412778 | Thrombophilic mutations in pre-eclampsia and pregnancy-induced hypertension.AIM: The aim of the present study was to determine the existence or prevalence of thrombophilic markers such as Factor V Leiden, prothrombin G20210A, protein S, protein C, activated protein C and anti-thrombin in pre-eclampsia and pregnancy-induced hypertensive patients. METHODS: Blood samples were collected from a total number of 124 women at the maternity unit, University of Malaya Medical Center. These included 49 patients with pre-eclampsia, 63 patients with pregnancy-induced hypertension and 12 normal pregnant women. DNA was extracted from the blood samples. Factor V Leiden (Taq I) and prothrombin G20210A (Hind III) genotyping was done on polymerase chain reaction-restriction fragment length polymorphism. Anti-thrombin activity and the concentrations of protein C, protein S and activated protein C were measured using the IL Coagulation System (Hemosil). RESULTS: Of the 124 subjects, one pre-eclampsia patient was homozygous for Factor V Leiden mutation but prothrombin G20210A mutation was not present in any of the subjects. The subject with Factor V Leiden mutation also had a low activated protein C resistance and a low protein S concentration. CONCLUSIONS: Factor V Leiden mutation is present in the Asian population and may very well serve as one of the genetic factors responsible for pre-eclampsia and other adverse pregnancy outcomes. |
| 19420351 | High prevalence of dysfibrinogenemia among patients with chronic thromboembolic pulmonary hypertension.The mechanism by which chronic thromboembolic pulmonary hypertension (CTEPH) develops after acute pulmonary thromboembolism is unknown. We previously reported that fibrin from CTEPH patients is relatively resistant to fibrinolysis in vitro. In the present study, we performed proteomic, genomic, and functional studies on fibrin(ogen) to investigate whether abnormal fibrin(ogen) might contribute to the pathogenesis of CTEPH. Reduced and denatured fibrinogen from 33 CTEPH patients was subjected to liquid chromatography-mass spectrometry analysis. Fibrinogen from 21 healthy controls was used to distinguish atypical from commonly occurring mass peaks. Atypical peaks were further investigated by targeted genomic DNA sequencing. Five fibrinogen variants with corresponding heterozygous gene mutations (dysfibrinogenemias) were observed in 5 of 33 CTEPH patients: Bbeta P235L/gamma R375W, Bbeta P235L/gamma Y114H, Bbeta P235L, Aalpha L69H, and Aalpha R554H (fibrinogens(San Diego I-V)). Bbeta P235L was found in 3 unrelated CTEPH patients. Functional analysis disclosed abnormalities in fibrin polymer structure and/or lysis with all CTEPH-associated mutations. These results suggest that, in some patients, differences in the molecular structure of fibrin may be implicated in the development of CTEPH after acute thromboembolism. |
| 20592457 | Analysis of polymorphisms in genes (AGT, MTHFR, GPIIIa, and GSTP1) associated with hypertension, thrombophilia and oxidative stress in Mestizo and Amerindian populations of Mexico.Several polymorphisms related to hypertension, thrombophilia, and oxidative stress has been associated with the development of cardiovascular disease. We analyzed the frequency of M235T angiotensinogen (AGT), A222V 5,10 methylenete-trahydrofolate reductase (MTHFR), L33P glycoprotein IIIa (GPIIIa), and I105V glutathione S-transferase P1 (GSTP1)} polymorphisms in 285 individuals belonging to Mexican-Mestizo and five Amerindian population from Mexico, by real time PCR allelic discrimination. Allele and genotype frequencies were compared using chi square tests. All populations followed the Hardy Weinberg equilibrium for assay markers with the exception of the Triki, whose were in Hardy Weinberg dysequilibrium for the glutathione S-transferase P1 polymorphism. Interestingly, according to all the analyzed single nucleotide polymorphisms (SNPs), the Triki population was the most differentiated and homogeneous group of the six populations analyzed. A comparison of our data with those previously published for some Caucasian, Asian and Black populations showed quite significant differences. These differences were remarkable with all the Mexican populations having a lower frequency of the 105V allele of the glutathione S-transferase P1 and reduced occurrence of the 222A allele of the 5,10 methylenetetrahydrofolate reductase. Our results show the genetic diversity among different Mexican populations and with other racial groups. |
| 20847307 | Body composition as determinant of thrombin generation in plasma: the Hoorn study.OBJECTIVE: The association between obesity and cardiovascular disease and venous thromboembolism might, at least partially, be explained by a hypercoagulable state. The extent to which body fat mass and its distribution contribute to a hypercoagulable state is unknown. In this study, we investigated the association between body composition and thrombin generation and evaluated the potential mediating role of low-grade inflammation. METHODS AND RESULTS: We studied 586 individuals from the Hoorn Study (mean age, 69.7 +/- 6.5 years, 298 women) in whom body composition was assessed by whole body dual-energy absorptiometry. Thrombin generation was measured using the calibrated automated thrombogram. Multiple regression analyses showed a positive association between total body fat and thrombin generation in women but not in men. In addition, detailed analyses of regional body composition showed that central but not peripheral fat mass was associated with greater thrombin generation and that there was a trend toward an inverse association with peripheral lean mass. The reported positive associations were partially attenuated by low-grade inflammation, however. CONCLUSIONS: Body fat mass, in particular a central pattern of fat distribution, is associated with higher levels of thrombin generation in elderly women but not in men. This association may partially be explained by adiposity-related low-grade inflammation, but this hypothesis needs to be further investigated in mechanistic/prospective studies. |
| 2732157 | Prevention of myocardial disease in JCR:LA-corpulent rats by running.The JCR:LA-corpulent rat is a congenic strain that, if homozygous for the cp gene, is obese with a very low-density lipoprotein hyperlipidemia and is insulin resistant. The male corpulent rats develop atherosclerotic lesions of the major arteries and myocardial lesions. Corpulent and lean male rats were induced through mild food restriction to run intensively (approximately 6,000 m/day) from 6 wk to 6 mo of age. Food restriction, especially when coupled with running, lowered all classes of lipids in the whole serum of corpulent rats. The principal changes in lipid concentrations were in the very low-density lipoprotein fraction. Food restriction caused a significant drop in fasting insulin levels of corpulent rats and decreased beta-cell hyperplasia. Both effects were more marked in the running animals. There was a significant decrease in myocardial lesion frequency in the food-restricted corpulent rats and an absence of lesions in the running rats. The results indicate that intensive physical activity can largely correct the lipid abnormalities and insulin resistance of this atherosclerosis-prone strain, and these changes are associated with inhibition of the disease process. However, moderate food restriction has similar effects, and the greater effects seen with intensive running may simply reflect an effectively more severe metabolic restriction in the presence of the exercise. |
| 17686833 | Effect of weight loss on LDL and HDL kinetics in the metabolic syndrome: associations with changes in plasma retinol-binding protein-4 and adiponectin levels.OBJECTIVE: The purpose of this study was to examine the effect of weight loss on LDL and HDL kinetics and plasma retinol-binding protein-4 (RBP-4) and adiponectin levels in men with the metabolic syndrome. RESEARCH DESIGN AND METHODS: LDL apolipoprotein (apo)B-100 and HDL apoA-I kinetics were studied in 35 obese men with the metabolic syndrome at the start and end of a 16-week intervention trial of a hypocaloric, low-fat diet (n = 20) versus a weight maintenance diet (n = 15) using a stable isotope technique and multicompartmental modeling. RESULTS: Consumption of the low-fat diet produced significant reductions (P < 0.01) in BMI, abdominal fat compartments, and homeostasis model assessment score compared with weight maintenance. These were associated with a significant increase in adiponectin and a fall in plasma RBP-4, triglycerides, LDL cholesterol, and LDL apoB-100 concentration (P < 0.05). Weight loss significantly increased the catabolism of LDL apoB-100 (+27%, P < 0.05) but did not affect production; it also decreased both the catabolic (-13%) and production (-13%) rates of HDL apoA-I (P < 0.05), thereby not altering plasma HDL apoA-I or HDL cholesterol concentrations. VLDL apoB-100 production fell significantly with weight loss (P < 0.05). The increase in LDL catabolism was inversely correlated with the fall in RBP-4 (r = -0.54, P < 0.05) and the decrease in HDL catabolism with the rise in adiponectin (r = -0.56, P < 0.01). CONCLUSIONS: In obese men with metabolic syndrome, weight loss with a low-fat diet decreases the plasma LDL apoB-100 concentration by increasing the catabolism of LDL apoB-100; weight loss also delays the catabolism of HDL apoA-I with a concomitant reduction in the secretion of HDL apoA-I. These effects of weight loss could partly involve changes in RBP-4 and adiponectin levels. |
| 19941836 | Endostatin expression in neurons during the early stage of cerebral ischemia is associated with neuronal apoptotic cell death in adult hypertensive rat model of stroke.Endostatin (ES) has been recognized as a potent anti-angiogenic factor. We here investigated the expression of ES in ischemic brain and the consequence of cells expressing ES after stroke in adult stroke-prone renovascular hypertensive rats. A single dose of Ca-074ME, a membrane-permeable cathepsin B (CB) specific inhibitor, or vehicle was given by intraperitoneal injection immediately after distal middle cerebral artery occlusion (dMCAO), ES expression was evaluated using fluorescent immunohistochemistry staining, and CB enzyme activity was tested by measuring the free 7-amino-4-methylcoumarin (AMC) released by CB from its' specific substrate, the Z-Arg-Arg-7-amido-4-methylcoumarin. ES immunoreactivity (IR) was significantly up-regulated as early as 6 h and returned to baseline level at 3 days in peri-infarct area following dMCAO. Double-staining experiment revealed that the majority of ischemia-induced ES positive cells were neurons. Furthermore, ES was co-labeled with CB and Cleaved Caspase-3(Asp175) whereas treatment with Ca-074ME reduced up-regulation of ES expression and attenuated apoptosis in peri-infarct neurons. Collectively, our data suggest that peri-infarct neurons express ES during the early stage of cerebral ischemia and treatment with Ca-074ME attenuates ES expression and apoptosis in peri-infarct neurons. |
| 21378161 | Metabolic regulation by C1q/TNF-related protein-13 (CTRP13): activation OF AMP-activated protein kinase and suppression of fatty acid-induced JNK signaling.Members of the C1q/TNF family play important and diverse roles in the immune, endocrine, skeletal, vascular, and sensory systems. Here, we identify and characterize CTRP13, a new and extremely conserved member of the C1q/TNF family. CTRP13 is preferentially expressed by adipose tissue and the brain in mice and predominantly by adipose tissue in humans. Within mouse adipose tissue, CTRP13 is largely expressed by cells of the stromal vascular compartment. Due to sexually dimorphic expression patterns, female mice have higher transcript and circulating CTRP13 levels than males. CTRP13 transcript and circulating levels are elevated in obese male mice, suggesting a potential role in energy metabolism. The insulin-sensitizing drug rosiglitazone also increases the expression of CTRP13 in adipocytes, which correlates with the insulin-sensitizing action of CTRP13. In a heterologous expression system, CTRP13 is secreted as a disulfide-linked oligomeric protein. When co-expressed, CTRP13 forms heteromeric complexes with a closely related family member, CTRP10. This heteromeric association does not involve conserved N-terminal Cys residues. Functional studies using purified recombinant protein demonstrated that CTRP13 is an adipokine that promotes glucose uptake in adipocytes, myotubes, and hepatocytes via activation of the AMPK signaling pathway. CTRP13 also ameliorates lipid-induced insulin resistance in hepatocytes through suppression of the SAPK/JNK stress signaling that impairs the insulin signaling pathway. Further, CTRP13 reduces glucose output in hepatocytes by inhibiting the mRNA expression of gluconeogenic enzymes, glucose-6-phosphatase and the cytosolic form of phosphoenolpyruvate carboxykinase. These results provide the first functional characterization of CTRP13 and establish its importance in glucose homeostasis. |

  
  
----- Star papers (those papers include more than 100 genes) -----  

|  |  |
| --- | --- |
| 20490451 | Type 2 diabetes risk alleles near ADCY5, CDKAL1 and HHEX-IDE are associated with reduced birthweight.AIMS/HYPOTHESIS: The fetal insulin hypothesis suggests that variation in the fetal genotype influencing insulin secretion or action may predispose to low birthweight and type 2 diabetes. We examined associations between 25 confirmed type 2 diabetes risk variants and birthweight in individuals from the Danish Inter99 population and in meta-analyses including Inter99 data and reported studies. METHODS: Midwife records from the Danish State Archives provided information on mother's age and parity, as well as birthweight, length at birth and prematurity of the newborn in 4,744 individuals of the population-based Inter99 study. We genotyped 25 risk alleles showing genome-wide associations with type 2 diabetes. RESULTS: Birthweight was inversely associated with the type 2 diabetes risk alleles of ADCY5 rs11708067 (beta = -33 g [95% CI -55, -10], p = 0.004) and CDKAL1 rs7756992 (beta = -22 g [95% CI -43, -1], p = 0.04). The association for the latter locus was confirmed in a meta-analysis (n = 24,885) (beta = -20 g [95% CI -29, -11], p = 5 x 10(-6)). The HHEX-IDE rs1111875 variant showed no significant association among Danes (p = 0.09); however, in a meta-analysis (n = 25,164) this type 2 diabetes risk allele was associated with lower birthweight (beta = -16 g [95% CI -24, -8], p = 8 x 10(-5)). On average, individuals with high genetic risk (>or=25 type 2 diabetes risk alleles) weighed marginally less at birth than those with low genetic risk (<25 type 2 diabetes risk alleles) (beta = -35 g [95% CI -69, -2], p = 0.037). CONCLUSIONS/INTERPRETATION: We report a novel association between the fetal ADCY5 type 2 diabetes risk allele and decreased birthweight, and confirm in meta-analyses associations between decreased birthweight and the type 2 diabetes risk alleles of HHEX-IDE and CDKAL1. No strong general effect on birthweight can be ascribed to the 25 common type 2 diabetes risk alleles. |
| 20682687 | Common variants in 40 genes assessed for diabetes incidence and response to metformin and lifestyle intervention in the diabetes prevention program.OBJECTIVE: Genome-wide association studies have begun to elucidate the genetic architecture of type 2 diabetes. We examined whether single nucleotide polymorphisms (SNPs) identified through targeted complementary approaches affect diabetes incidence in the at-risk population of the Diabetes Prevention Program (DPP) and whether they influence a response to preventive interventions. RESEARCH DESIGN AND METHODS: We selected SNPs identified by prior genome-wide association studies for type 2 diabetes and related traits, or capturing common variation in 40 candidate genes previously associated with type 2 diabetes, implicated in monogenic diabetes, encoding type 2 diabetes drug targets or drug-metabolizing/transporting enzymes, or involved in relevant physiological processes. We analyzed 1,590 SNPs for association with incident diabetes and their interaction with response to metformin or lifestyle interventions in 2,994 DPP participants. We controlled for multiple hypothesis testing by assessing false discovery rates. RESULTS: We replicated the association of variants in the metformin transporter gene SLC47A1 with metformin response and detected nominal interactions in the AMP kinase (AMPK) gene STK11, the AMPK subunit genes PRKAA1 and PRKAA2, and a missense SNP in SLC22A1, which encodes another metformin transporter. The most significant association with diabetes incidence occurred in the AMPK subunit gene PRKAG2 (hazard ratio 1.24, 95% CI 1.09-1.40, P = 7 x 10(-4)). Overall, there were nominal associations with diabetes incidence at 85 SNPs and nominal interactions with the metformin and lifestyle interventions at 91 and 69 mostly nonoverlapping SNPs, respectively. The lowest P values were consistent with experiment-wide 33% false discovery rates. CONCLUSIONS: We have identified potential genetic determinants of metformin response. These results merit confirmation in independent samples. |
| 19020323 | Genotype score in addition to common risk factors for prediction of type 2 diabetes.BACKGROUND: Multiple genetic loci have been convincingly associated with the risk of type 2 diabetes mellitus. We tested the hypothesis that knowledge of these loci allows better prediction of risk than knowledge of common phenotypic risk factors alone. METHODS: We genotyped single-nucleotide polymorphisms (SNPs) at 18 loci associated with diabetes in 2377 participants of the Framingham Offspring Study. We created a genotype score from the number of risk alleles and used logistic regression to generate C statistics indicating the extent to which the genotype score can discriminate the risk of diabetes when used alone and in addition to clinical risk factors. RESULTS: There were 255 new cases of diabetes during 28 years of follow-up. The mean (+/-SD) genotype score was 17.7+/-2.7 among subjects in whom diabetes developed and 17.1+/-2.6 among those in whom diabetes did not develop (P<0.001). The sex-adjusted odds ratio for diabetes was 1.12 per risk allele (95% confidence interval, 1.07 to 1.17). The C statistic was 0.534 without the genotype score and 0.581 with the score (P=0.01). In a model adjusted for sex and self-reported family history of diabetes, the C statistic was 0.595 without the genotype score and 0.615 with the score (P=0.11). In a model adjusted for age, sex, family history, body-mass index, fasting glucose level, systolic blood pressure, high-density lipoprotein cholesterol level, and triglyceride level, the C statistic was 0.900 without the genotype score and 0.901 with the score (P=0.49). The genotype score resulted in the appropriate risk reclassification of, at most, 4% of the subjects. CONCLUSIONS: A genotype score based on 18 risk alleles predicted new cases of diabetes in the community but provided only a slightly better prediction of risk than knowledge of common risk factors alone. |
| 19587357 | A systematic meta-analysis of genetic association studies for diabetic retinopathy.OBJECTIVE: Diabetic retinopathy is a sight-threatening microvascular complication of diabetes with a complex multifactorial pathogenesis. A systematic meta-analysis was undertaken to collectively assess genetic studies and determine which previously investigated polymorphisms are associated with diabetic retinopathy. RESEARCH DESIGN AND METHODS: All studies investigating the association of genetic variants with the development of diabetic retinopathy were identified in PubMed and ISI Web of Knowledge. Crude odds ratios (ORs) and 95% CIs were calculated for single nucleotide polymorphisms and microsatellite markers previously investigated in at least two published studies. RESULTS: Twenty genes and 34 variants have previously been studied in multiple cohorts. The aldose reductase (AKR1B1) gene was found to have the largest number of polymorphisms significantly associated with diabetic retinopathy. The z-2 microsatellite was found to confer risk (OR 2.33 [95% CI 1.49-3.64], P = 2 x 10(-4)) in type 1 and type 2 diabetes and z+2 to confer protection (0.58 [0.36-0.93], P = 0.02) against diabetic retinopathy in type 2 diabetes regardless of ethnicity. The T allele of the AKR1B1 promoter rs759853 variant is also significantly protective against diabetic retinopathy in type 1 diabetes (0.5 [0.35-0.71], P = 1.00 x 10(-4)), regardless of ethnicity. These associations were also found in the white population alone (P < 0.05). Polymorphisms in NOS3, VEGF, ITGA2, and ICAM1 are also associated with diabetic retinopathy after meta-analysis. CONCLUSIONS: Variations within the AKR1B1 gene are highly significantly associated with diabetic retinopathy development irrespective of ethnicity. Identification of genetic risk factors in diabetic retinopathy will assist in further understanding of this complex and debilitating diabetes complication. |
| 20889853 | Genetic risk reclassification for type 2 diabetes by age below or above 50 years using 40 type 2 diabetes risk single nucleotide polymorphisms.OBJECTIVE: To test if knowledge of type 2 diabetes genetic variants improves disease prediction. RESEARCH DESIGN AND METHODS: We tested 40 single nucleotide polymorphisms (SNPs) associated with diabetes in 3,471 Framingham Offspring Study subjects followed over 34 years using pooled logistic regression models stratified by age (<50 years, diabetes cases = 144; or >/=50 years, diabetes cases = 302). Models included clinical risk factors and a 40-SNP weighted genetic risk score. RESULTS: In people <50 years of age, the clinical risk factors model C-statistic was 0.908; the 40-SNP score increased it to 0.911 (P = 0.3; net reclassification improvement (NRI): 10.2%, P = 0.001). In people >/=50 years of age, the C-statistics without and with the score were 0.883 and 0.884 (P = 0.2; NRI: 0.4%). The risk per risk allele was higher in people <50 than >/=50 years of age (24 vs. 11%; P value for age interaction = 0.02). CONCLUSIONS: Knowledge of common genetic variation appropriately reclassifies younger people for type 2 diabetes risk beyond clinical risk factors but not older people. |
| 15188402 | Proteins associated with type II bone morphogenetic protein receptor (BMPR-II) and identified by two-dimensional gel electrophoresis and mass spectrometry.Bone morphogenetic proteins (BMP) are polypeptide growth factors that regulate cell differentiation and proliferation. BMPs bind to type I and type II serine/threonine kinase receptors to initiate intracellular signalling. BMPR-II is the type II receptor, its mutations lead to hereditary pulmonary hypertension, and knockout of Bmpr-II results in early embryonic lethality. To identify novel interacting proteins and explore signalling pathways that can be initiated by BMPR-II, we performed glutathione-S-transferase (GST) pull-down assays with BMPR-II protein constructs fused to GST and extracts of mouse myoblast C2C12 cells. We generated three constructs which contain different parts of the cytoplasmic region of BMPR-II: full-length cytoplasmic part of BMPR-II, only the kinase domain, or only the C-terminal tail of BMPR-II. Proteins which formed complexes with these BMPR-II constructs were analyzed by two-dimensional gel electrophoresis (2-D GE), and specifically interacting proteins were identified by matrix-assisted laser desorption/ionization-time of flight-mass spectrometry (MALDI-TOF-MS). We identified 33 interacting proteins; 11 proteins interacted with the C-terminal tail of BMPR-II, 4 with full-length BMPR-II, and 18 with a short form of the receptor with a deleted tail. Fourteen proteins have assigned functions in various signalling processes, suggesting links of BMP signalling to regulation of MAP kinase pathway, apoptosis, transcription, PKCss, and PKA. Five of the identified proteins are components of the cytoskeleton, and four are enzymes involved in metabolism, e.g., processing of estrogens or lipids. We confirmed interaction of PKC beta and CtBP with BMPR-II using immunodetection. We showed that the C-terminal tail of BMPR-II provides binding sites for a number of regulatory proteins that may initiate Smad-independent signalling. |
| 20581827 | Twelve type 2 diabetes susceptibility loci identified through large-scale association analysis.By combining genome-wide association data from 8,130 individuals with type 2 diabetes (T2D) and 38,987 controls of European descent and following up previously unidentified meta-analysis signals in a further 34,412 cases and 59,925 controls, we identified 12 new T2D association signals with combined P<5x10(-8). These include a second independent signal at the KCNQ1 locus; the first report, to our knowledge, of an X-chromosomal association (near DUSP9); and a further instance of overlap between loci implicated in monogenic and multifactorial forms of diabetes (at HNF1A). The identified loci affect both beta-cell function and insulin action, and, overall, T2D association signals show evidence of enrichment for genes involved in cell cycle regulation. We also show that a high proportion of T2D susceptibility loci harbor independent association signals influencing apparently unrelated complex traits. |
| 21719444 | A high-sugar diet produces obesity and insulin resistance in wild-type Drosophila.Insulin-resistant, 'type 2' diabetes (T2D) results from a complex interplay between genes and environment. In particular, both caloric excess and obesity are strongly associated with T2D across many genetic backgrounds. To gain insights into how dietary excess affects insulin resistance, we studied the simple model organism Drosophila melanogaster. Larvae reared on a high-sugar diet were hyperglycemic, insulin resistant and accumulated fat--hallmarks of T2D--compared with those reared on control diets. Excess dietary sugars, but not fats or proteins, elicited insulin-resistant phenotypes. Expression of genes involved in lipogenesis, gluconeogenesis and beta-oxidation was upregulated in high-sugar-fed larvae, as were FOXO targets, consistent with known mechanisms of insulin resistance in humans. These data establish a novel Drosophila model of diet-induced insulin resistance that bears strong similarity to the pathophysiology of T2D in humans. |

Copyright © CoCiter 2011-2013. >>
Designed by QIAO Nan & HUANG Yi >>
Hanlab
