# Supplementary material for: Partial depletion of yolk during zebrafish embryogenesis changes the dynamics of methionine cycle and metabolic genes
Source: BMC Genomics. 2015 Jun 4;16(1):427. doi: 10.1186/s12864-015-1654-6 (PMC4455928; doi:10.1186/s12864-015-1654-6)
Supplement: Additional file 11: — CoCiter output of differentially expressed genes at 48 hpf. Microarray outcome associated to the terms “hypertension”, “obesity”, “type 2 diabetes” and “osteoporosis”. [file 12864_2015_1654_MOESM11_ESM.html]

CoCiter v1.1


**CoCiter v1.1**

## Co-citation analysis on genes/terms

Home
Gene-Gene
Gene-Term
Term-Term
ViewJob
HanLab

## Your search results:

**Genes/Terms involved in mistake below are not calculated in this result.**
Genes BC114317, ENSDART00000061430, XM\_001335964, CO802608, DN894763, XM\_683311, ENSDART00000057913, TC430452, 100330330, XM\_001336450, CK703250, XM\_001344486, AF533659, XM\_681577, ENSDART00000109034, ENSDART00000099375, DV587557, TC371560, EH455384, XM\_695355, TC389911, TC365406, ENSDART00000079718, 793474, CN508883, XM\_001345402, XM\_001921953, ENSDART00000111720, CT710128 in the Gene Set are not included in the database of that species!

| Set | Genes/Terms | PubMed Count | CI | Significance |
| --- | --- | --- | --- | --- |
| GeneSet 131 genes | agt (322485), serpine1 (100136840), ucp2 (555812), mmp9 (406397), ptgs2b (559020), sirt1 (797132), fos (394198), cebpb (140814), LOC557301 (557301), gstp2 (553169), txnipa (368359), slc5a1 (393654), ctssb.1 (554157), plaua (100008445), hbegfa (797938), si:dkey-260c8.4 (100004261), cga (402987), zgc:112302 (574008), fkbp5 (368924), LOC568363 (568363), arrdc3a (566685), LOC100006782 (100006782), mat2ab (791612), zgc:92480 (445091), nrxn3a (563598), cd74a (58113), tcap (556258), egln3 (406602), hmha1 (555849), cldn7a (436612), gadd45ba (406304), pkp2 (568060), cilp2 (557778), LOC100007488 (100007488), fstl3 (557352), odc1 (114426), lzts2b (100005471), il1b (405770), lepa (100150233), abat (378968), gadd45bb (497646), LOC792623 (792623), LOC402880 (402880), glrbb (445193), fosl1a (564241), LOC571737 (571737), cpa2 (445052), slc16a9a (795588), zgc:136864 (677749), qars (394188), elovl7b (327274), slc25a38a (767662), gcshb (436852), LOC559914 (559914), slc25a15a (791156), tmem41ab (406356), poc1a (406322), ggact.1 (777609), sst2 (79186), mknk2b (373121), tnfrsf21 (564874), stk35 (568080), ggact.2 (447805), LOC100001286 (100001286), LOC569604 (569604), wu:fk57a03 (386815), itga8 (100141346), arrdc3b (447866), lgals2b (393486), si:ch211-132g1.3 (100004401), pfkfb4l (386663), zgc:194101 (798684), zgc:63759 (393460), slc38a5b (436921), cbx7a (550551), abcb9 (570148), smcr8a (558696), smtlb (553408), zgc:113142 (503524), gldc (321621), crygm2c (493628), stc1l (393511), mmp13a (387293), ap3s1 (449791), LOC570238 (570238), LOC100331324 (100331324), im:7146765 (497532), si:ch211-284e13.9 (566600), si:dkey-182g1.6 (100005456), si:dkey-31e10.6 (100334656), sb:cb25 (321045), zgc:172260 (796228), LOC100000332 (100000332), fep15 (100329562), LOC559843 (559843), lepb (564348), tnfb (554167), col28a1a (100294690), scpp8 (100270761), si:dkey-40h20.1 (100526805), si:dkey-7c18.24 (562950), LOC100148704 (100148704), lyrm5b (561760), si:ch211-107o23.1 (571470), cyp2x8 (431734), si:ch211-270n8.1 (792467), zgc:158343 (568780), zgc:110340 (541345), wu:fb01a11 (337181), hephl1 (561607), si:dkey-182g1.3 (553357), bcl11ba (100093707), si:ch211-217k17.10 (798704), bmf2 (751764), lgals1l1 (326706), LOC793794 (793794), pth1a (405886), si:ch211-105c13.3 (325758), wu:fc21d07 (324189), zgc:172290 (796074), crygm2b (553954), zgc:174689 (795883), pnp4b (402940), LOC100005016 (100005016), zgc:113442 (503592), sgk2b (559050), gnrh2 (353222), ipo8 (555520), atf5b (567253), h1fx (322508), sb:cb930 (399650) | 1574 | 10.6211 | **p-value** <0.001\*\*\* **permutation** 1000 x **adjusted CI** 3.7513 |
| TermSet 4 terms | hypertension, obesity, type 2 diabetes, osteoporosis |

- (p-value significance: \*\*\*: p < 0.001; \*\*: p < 0.005; \*: p < 0.01)
  

The one-to-all results.

| Gene v.s. all terms | Term v.s. all Genes |
| --- | --- |
| | Gene | Count | CI | | --- | --- | --- | |    agt (322485) | 642 | 9.3287 | |    serpine1 (100136840) | 210 | 7.7211 | |    ucp2 (555812) | 194 | 7.6073 | |    mmp9 (406397) | 113 | 6.8329 | |    ptgs2b (559020) | 100 | 6.6582 | |    sirt1 (797132) | 94 | 6.5699 | |    fos (394198) | 51 | 5.7004 | |    cebpb (140814) | 31 | 5.0 | |    LOC557301 (557301) | 31 | 5.0 | |    gstp2 (553169) | 28 | 4.858 | |    txnipa (368359) | 20 | 4.3923 | |    slc5a1 (393654) | 14 | 3.9069 | |    ctssb.1 (554157) | 13 | 3.8074 | |    plaua (100008445) | 7 | 3.0 | |    hbegfa (797938) | 7 | 3.0 | |    si:dkey-260c8.4 (100004261) | 6 | 2.8074 | |    cga (402987) | 6 | 2.8074 | |    zgc:112302 (574008) | 5 | 2.585 | |    fkbp5 (368924) | 5 | 2.585 | |    LOC568363 (568363) | 5 | 2.585 | |    arrdc3a (566685) | 4 | 2.3219 | |    LOC100006782 (100006782) | 4 | 2.3219 | |    mat2ab (791612) | 4 | 2.3219 | |    zgc:92480 (445091) | 3 | 2.0 | |    nrxn3a (563598) | 3 | 2.0 | |    cd74a (58113) | 3 | 2.0 | |    tcap (556258) | 3 | 2.0 | |    egln3 (406602) | 3 | 2.0 | |    hmha1 (555849) | 2 | 1.585 | |    cldn7a (436612) | 2 | 1.585 | |    gadd45ba (406304) | 2 | 1.585 | |    pkp2 (568060) | 2 | 1.585 | |    cilp2 (557778) | 2 | 1.585 | |    LOC100007488 (100007488) | 2 | 1.585 | |    fstl3 (557352) | 2 | 1.585 | |    odc1 (114426) | 2 | 1.585 | |    lzts2b (100005471) | 2 | 1.585 | |    il1b (405770) | 2 | 1.585 | |    lepa (100150233) | 2 | 1.585 | |    abat (378968) | 2 | 1.585 | |    gadd45bb (497646) | 2 | 1.585 | |    LOC792623 (792623) | 2 | 1.585 | |    LOC402880 (402880) | 2 | 1.585 | |    glrbb (445193) | 1 | 1.0 | |    fosl1a (564241) | 1 | 1.0 | |    LOC571737 (571737) | 1 | 1.0 | |    cpa2 (445052) | 1 | 1.0 | |    slc16a9a (795588) | 1 | 1.0 | |    zgc:136864 (677749) | 1 | 1.0 | |    qars (394188) | 1 | 1.0 | |    elovl7b (327274) | 1 | 1.0 | |    slc25a38a (767662) | 1 | 1.0 | |    gcshb (436852) | 1 | 1.0 | |    LOC559914 (559914) | 1 | 1.0 | |    slc25a15a (791156) | 1 | 1.0 | |    tmem41ab (406356) | 1 | 1.0 | |    poc1a (406322) | 1 | 1.0 | |    ggact.1 (777609) | 1 | 1.0 | |    sst2 (79186) | 1 | 1.0 | |    mknk2b (373121) | 1 | 1.0 | |    tnfrsf21 (564874) | 1 | 1.0 | |    stk35 (568080) | 1 | 1.0 | |    ggact.2 (447805) | 1 | 1.0 | |    LOC100001286 (100001286) | 0 | 0.0 | |    LOC569604 (569604) | 0 | 0.0 | |    wu:fk57a03 (386815) | 0 | 0.0 | |    itga8 (100141346) | 0 | 0.0 | |    arrdc3b (447866) | 0 | 0.0 | |    lgals2b (393486) | 0 | 0.0 | |    si:ch211-132g1.3 (100004401) | 0 | 0.0 | |    pfkfb4l (386663) | 0 | 0.0 | |    zgc:194101 (798684) | 0 | 0.0 | |    zgc:63759 (393460) | 0 | 0.0 | |    slc38a5b (436921) | 0 | 0.0 | |    cbx7a (550551) | 0 | 0.0 | |    abcb9 (570148) | 0 | 0.0 | |    smcr8a (558696) | 0 | 0.0 | |    smtlb (553408) | 0 | 0.0 | |    zgc:113142 (503524) | 0 | 0.0 | |    gldc (321621) | 0 | 0.0 | |    crygm2c (493628) | 0 | 0.0 | |    stc1l (393511) | 0 | 0.0 | |    mmp13a (387293) | 0 | 0.0 | |    ap3s1 (449791) | 0 | 0.0 | |    LOC570238 (570238) | 0 | 0.0 | |    LOC100331324 (100331324) | 0 | 0.0 | |    im:7146765 (497532) | 0 | 0.0 | |    si:ch211-284e13.9 (566600) | 0 | 0.0 | |    si:dkey-182g1.6 (100005456) | 0 | 0.0 | |    si:dkey-31e10.6 (100334656) | 0 | 0.0 | |    sb:cb25 (321045) | 0 | 0.0 | |    zgc:172260 (796228) | 0 | 0.0 | |    LOC100000332 (100000332) | 0 | 0.0 | |    fep15 (100329562) | 0 | 0.0 | |    LOC559843 (559843) | 0 | 0.0 | |    lepb (564348) | 0 | 0.0 | |    tnfb (554167) | 0 | 0.0 | |    col28a1a (100294690) | 0 | 0.0 | |    scpp8 (100270761) | 0 | 0.0 | |    si:dkey-40h20.1 (100526805) | 0 | 0.0 | |    si:dkey-7c18.24 (562950) | 0 | 0.0 | |    LOC100148704 (100148704) | 0 | 0.0 | |    lyrm5b (561760) | 0 | 0.0 | |    si:ch211-107o23.1 (571470) | 0 | 0.0 | |    cyp2x8 (431734) | 0 | 0.0 | |    si:ch211-270n8.1 (792467) | 0 | 0.0 | |    zgc:158343 (568780) | 0 | 0.0 | |    zgc:110340 (541345) | 0 | 0.0 | |    wu:fb01a11 (337181) | 0 | 0.0 | |    hephl1 (561607) | 0 | 0.0 | |    si:dkey-182g1.3 (553357) | 0 | 0.0 | |    bcl11ba (100093707) | 0 | 0.0 | |    si:ch211-217k17.10 (798704) | 0 | 0.0 | |    bmf2 (751764) | 0 | 0.0 | |    lgals1l1 (326706) | 0 | 0.0 | |    LOC793794 (793794) | 0 | 0.0 | |    pth1a (405886) | 0 | 0.0 | |    si:ch211-105c13.3 (325758) | 0 | 0.0 | |    wu:fc21d07 (324189) | 0 | 0.0 | |    zgc:172290 (796074) | 0 | 0.0 | |    crygm2b (553954) | 0 | 0.0 | |    zgc:174689 (795883) | 0 | 0.0 | |    pnp4b (402940) | 0 | 0.0 | |    LOC100005016 (100005016) | 0 | 0.0 | |    zgc:113442 (503592) | 0 | 0.0 | |    sgk2b (559050) | 0 | 0.0 | |    gnrh2 (353222) | 0 | 0.0 | |    ipo8 (555520) | 0 | 0.0 | |    atf5b (567253) | 0 | 0.0 | |    h1fx (322508) | 0 | 0.0 | |    sb:cb930 (399650) | 0 | 0.0 | | | Term | Count | CI | | --- | --- | --- | |    hypertension | 876 | 9.7764 | |    obesity | 513 | 9.0056 | |    type 2 diabetes | 293 | 8.1997 | |    osteoporosis | 40 | 5.3576 | |

**100** of the **1574** PubMed papers involved are listed below (sorted by relevance):

| PubMed ID | Title |
| --- | --- |
| 15106800 | A common polymorphism of uncoupling protein 2 gene is associated with hypertension.OBJECTIVES: The genes responsible for obesity are also candidate genes for obesity-related conditions, such as hypertension and type 2 diabetes. A functional polymorphism in the uncoupling protein 2 (UCP2) promoter has been reported to be associated with obesity in Caucasians. To clarify the contribution of this polymorphism to obesity and related conditions, we studied the association of the -866 G/A polymorphism of the UCP2 gene with obesity, hypertension and type 2 diabetes mellitus. METHODS: A total of 632 unrelated Japanese subjects were studied: 342 type 2 diabetic patients (among them, 158 patients complicated with hypertension), 156 hypertensive patients without diabetes mellitus and 134 control subjects. The -866 G/A polymorphism of UCP2 was determined by polymerase chain reaction (PCR)-restriction fragment length polymorphism (RFLP). RESULTS: The frequency of the minor A allele was significantly higher in Japanese than in Caucasians (48.9 versus 37.2%, P=0.01). In contrast to the significant association with obesity in Caucasians, the polymorphism was not associated with obesity in Japanese. The polymorphism, however, was significantly associated with hypertension in Japanese (frequency of A allele: 51.8% in hypertensives versus 46.6% in normotensives, P<0.05). No significant difference was observed in body mass index (BMI), fasting insulin level or HOMA-R between patients with different genotypes. CONCLUSION: These data indicate that the polymorphism of the UCP2 gene is associated with hypertension, and suggest the possibility of UCP2 as a target molecule for studies on the etiology and treatment of hypertension. |
| 22236479 | Thioredoxin interacting protein genetic variation is associated with diabetes and hypertension in the Brazilian general population.OBJECTIVE: To investigate the relationship between TXNIP polymorphisms, diabetes and hypertension phenotypes in the Brazilian general population. METHODS: Five hundred seventy-six individuals randomly selected from the general urban population according to the MONICA-WHO project guidelines were phenotyped for cardiovascular risk factors. A second, independent, sample composed of 487 family-trios from a different site was also selected. Nine TXNIP polymorphisms were studied. The potential association between TXNIP variability and glucose-phenotypes in children was also explored. TXNIP expression was quantified by real-time PCR in 53 samples from human smooth muscle cells primary culture. RESULTS: TXNIP rs7211 and rs7212 polymorphisms were significantly associated with glucose and blood pressure related phenotypes. In multivariate logistic regression models the studied markers remained associated with diabetes even after adjustment for covariates. TXNIP rs7211 T/rs7212 G haplotype (present in approximately 17% of individuals) was significantly associated to diabetes in both samples. In children, the TXNIP rs7211 T/rs7212 G haplotype was associated with fasting insulin concentrations. Finally, cells harboring TXNIP rs7212 G allele presented higher TXNIP expression levels compared with carriers of TXNIP rs7212 CC genotype (p=0.02). CONCLUSION: Carriers of TXNIP genetic variants presented higher TXNIP expression, early signs of glucose homeostasis derangement and increased susceptibility to chronic metabolic conditions such as diabetes and hypertension. Our data suggest that genetic variation in the TXNIP gene may act as a "common ground" modulator of both traits: diabetes and hypertension. |
| 22815502 | TXNIP in Agrp neurons regulates adiposity, energy expenditure, and central leptin sensitivity.Thioredoxin interacting protein (TXNIP) has recently been described as a key regulator of energy metabolism through pleiotropic actions that include nutrient sensing in the mediobasal hypothalamus (MBH). However, the role of TXNIP in neurochemically specific hypothalamic subpopulations and the circuits downstream from MBH TXNIP engaged to regulate energy homeostasis remain unexplored. To evaluate the metabolic role of TXNIP activity specifically within arcuate Agrp neurons, we generated Agrp-specific TXNIP gain-of-function and loss-of-function mouse models using Agrp-Ires-cre mice, TXNIP (flox/flox) mice, and a lentivector expressing the human TXNIP isoform conditionally in the presence of Cre recombinase. Overexpression of TXNIP in Agrp neurons predisposed to diet-induced obesity and adipose tissue storage by decreasing energy expenditure and spontaneous locomotion, without affecting food intake. Conversely, Agrp neuronal TXNIP deletion protected against diet-induced obesity and adipose tissue storage by increasing energy expenditure and spontaneous locomotion, also without affecting food intake. TXNIP overexpression in Agrp neurons did not primarily affect glycemic control, whereas deletion of TXNIP in Agrp neurons improved fasting glucose levels and glucose tolerance independently of its effects on body weight and adiposity. Bidirectional manipulation of TXNIP expression induced reciprocal changes in central leptin sensitivity and the neural regulation of lipolysis. Together, these results identify a critical role for TXNIP in Agrp neurons in mediating diet-induced obesity through the regulation of energy expenditure and adipose tissue metabolism, independently of food intake. They also reveal a previously unidentified role for Agrp neurons in the brain-adipose axis. |
| 12743644 | The role of uncoupling protein 2 in the development of type 2 diabetes.Uncoupling proteins (UCP) are carriers expressed in the mitochondrial inner membrane that uncouple oxygen consumption by the respiratory chain from ATP synthesis. UCP2 is a member of the multigenic UCP family that is expressed in a wide range of tissues and organs. Possible functions of UCP2 include control of ATP synthesis, regulation of fatty acid metabolism and control of reactive oxygen species production. UCP2 expression in tissues involved in lipid and energy metabolism and mapping of the gene to a region linked to obesity and hyperinsulinemia prompted studies on the involvement of UCP2 in metabolic disorders, and especially in type 2 diabetes. In human adipose tissue and skeletal muscle, UCP2 expression is increased during fasting. The carrier was shown to be under the control of fatty acids and thyroid hormones in vivo. An upregulation has been observed in the liver during high-fat feeding and obesity. However, data in UCP2 gene knockout mice do not support a role for UCP2 in steatohepatitis. The most compelling metabolic role of UCP2 comes from studies in pancreatic beta cells. Overexpression in isolated pancreatic islets results in decreased ATP content and blunted glucose-stimulated insulin secretion. UCP2-deficient mice show an increased ATP level and an enhanced insulin secretion. Lack of UCP2 dramatically improves insulin secretion and decreases hyperglycemia in leptin-deficient mice. The role of UCP2 in the control of insulin secretion constitutes, to date, the most pertinent path to investigate in a therapeutic perspective. |
| 19996381 | SIRT1 inhibits inflammatory pathways in macrophages and modulates insulin sensitivity.Chronic inflammation is an important etiology underlying obesity-related disorders such as insulin resistance and type 2 diabetes, and recent findings indicate that the macrophage can be the initiating cell type responsible for this chronic inflammatory state. The mammalian silent information regulator 2 homolog SIRT1 modulates several physiological processes important for life span, and a potential role of SIRT1 in the regulation of insulin sensitivity has been shown. However, with respect to inflammation, the role of SIRT1 in regulating the proinflammatory pathway within macrophages is poorly understood. Here, we show that knockdown of SIRT1 in the mouse macrophage RAW264.7 cell line and in intraperitoneal macrophages broadly activates the JNK and IKK inflammatory pathways and increases LPS-stimulated TNFalpha secretion. Moreover, gene expression profiles reveal that SIRT1 knockdown leads to an increase in inflammatory gene expression. We also demonstrate that SIRT1 activators inhibit LPS-stimulated inflammatory pathways, as well as secretion of TNFalpha, in a SIRT1-dependent manner in RAW264.7 cells and in primary intraperitoneal macrophages. Treatment of Zucker fatty rats with a SIRT1 activator leads to greatly improved glucose tolerance, reduced hyperinsulinemia, and enhanced systemic insulin sensitivity during glucose clamp studies. These in vivo insulin-sensitizing effects were accompanied by a reduction in tissue inflammation markers and a decrease in the adipose tissue macrophage proinflammatory state, fully consistent with the in vitro effects of SIRT1 in macrophages. In conclusion, these results define a novel role for SIRT1 as an important regulator of macrophage inflammatory responses in the context of insulin resistance and raise the possibility that targeting of SIRT1 might be a useful strategy for treating the inflammatory component of metabolic diseases. |
| 23263277 | Role of FK506 binding protein 5 (FKBP5) in osteoclast differentiation.OBJECTIVES: We previously disclosed the enhanced expression of FK506 binding protein 5 (FKBP5) messenger RNA (mRNA) in bone marrow (BM) CD34(+) cells in rheumatoid arthritis (RA), in which systemic osteoporosis takes place. Since BM CD34(+) cells are precursors of osteoclasts, it is possible that FKBP5 overexpression might lead to osteoporosis by affecting osteoclastogenesis. We therefore explore the influences of FKBP5 in osteoclast differentiation. METHODS: Stable transfectants of RAW264.7 overexpressing murine FKBP5 gene were established. Osteoclast differentiation was induced by receptor activator of nuclear factor kappa B (NF-kappaB) ligand and was evaluated by tartrate-resistant acid phosphatase (TRAP) staining and pit formation assay. RESULTS: FKBP5 transfectants of RAW264.7 generated higher numbers of TRAP-positive multinucleated cells with increased activity of pit formation on calcium phosphate-coated culture slides than mock transfectants. The enhancement of osteoclast differentiation of FKBP5 transfectants was only partially inhibited by N-acetyl L-cysteine. Finally, glucocorticoid enhanced FKBP5 mRNA expression as well as osteoclast differentiation of RAW264.7 cells in a dose-dependent manner. CONCLUSIONS: These results indicate that FKBP5 promotes osteoclast differentiation by a mechanism distinct from NF-kappaB activation. Moreover, the data suggest that FKBP5 might play a role in bone destruction and development of osteoporosis in RA as well as in glucocorticoid-induced osteoporosis. |
| 14655518 | Aberrant monocyte prostaglandin synthase 2 (PGS2) expression in type 1 diabetes before and after disease onset.METHODS: We examined monocyte prostaglandin synthase 2 (PGS2/COX2) expression in individuals at risk for or with type 1 diabetes including: (i) 58 established type 1 and 2 diabetic patients; (ii) 34 autoantibody positive (AA+) children and adults; (iii) 164 infants and young children with insulin-dependent diabetes mellitus (IDDM) susceptibility human leukocyte antigen (HLA) alleles; and (iv) 37 healthy control individuals, over a 5-yr period. RESULTS: Established type 1 diabetic patients (1 month to 30+ yr post-disease onset) had significantly higher PGS2 expression than healthy controls; by contrast, insulin-treated type 2 diabetic patients had significantly lower PGS2 expression than healthy controls. Longitudinal studies of AA+ subjects at risk for type 1 diabetes indicated that 73% (11/15) of individuals who developed this disease during the study period expressed high levels of PGS2 prior to or after onset. We also found high level PGS2 expression in genetically at-risk infants and young children that correlated with having a first-degree relative with type 1 diabetes, but not with age, gender, or HLA genotype. In this population, high level PGS2 expression coincided with or preceded autoantibody detection in 30% (3/10) of subjects. CONCLUSIONS: These findings suggest that high level monocyte PGS2 expression, although subject to fluctuation, is present in at-risk subjects at an early age and is maintained during progression to and after type 1 diabetes onset. |
| 19155787 | Association of UCP2 and UCP3 polymorphisms with heart rate variability in Japanese men.OBJECTIVES: The mitochondrial uncoupling proteins UCP2 and UCP3 are implicated in energy metabolism and regulation of reactive oxygen species, which are closely involved in autonomic nervous system function. Heart rate variability (HRV) reflects cardiac autonomic regulation and has been used to evaluate dysfunction of the autonomic nervous system in hypertension and cardiovascular diseases. We examined the association between polymorphisms in the UCP2 and UCP3 genes and HRV in healthy young Japanese men. METHODS: The 45 bp insertion/deletion polymorphism in exon8 of UCP2 and the -55C/T polymorphism in the UCP3 promoter region were genotyped (n = 255). Cardiac autonomic function was evaluated by power spectral analysis of HRV during supine rest and in a standing position. Low-frequency (<0.15 Hz) and high-frequency (>0.15 Hz) components of HRV were quantified by frequency domain analysis. RESULTS: The I/I genotype of the UCP2 45 bp insertion/deletion polymorphism was associated with relatively higher blood pressure and HRV sympathetic indices (low frequency percentage and low frequency:high frequency ratio) at supine rest. For the -55C/T polymorphism of UCP3, individuals carrying the -55T allele had significantly lower HRV sympathetic indices, but higher HRV parasympathetic indices (high frequency and high frequency percentage), than carriers of the C/C genotype at standing. Both UCP2 and UCP3 polymorphisms were significantly associated with a third-degree family history of hypertension, diabetes, and obesity. Additionally, carriers of the UCP2 45 bp I allele -UCP3 -55C/C combined genotype had the lowest HRV sympathetic and the highest HRV parasympathetic indices at standing among the combined genotypes. CONCLUSION: UCP2 and UCP3 polymorphisms were associated with HRV in young and healthy states, suggesting a significant relationship between autonomic cardiovascular regulation and UCP2/UCP3 polymorphisms. |
| 20687374 | [Role of the M235T (c.704c>T) polymorphism of angiotensynogen gene as well as A724A (c.2171G>A) polymorphism of SERCA2a gene in ethiopathogenesis of left ventricular hypertrophy in essential hypertension].Left ventricle hypertrophy (LVH) is main organ complication developing in the course of primary hypertension. Among 'candidates genes' related with development of hypertension as well as LVH; the promoting, but not crucial, influence of (c.704C>T) angiotensinogen (AGT) gene was found. The elevated calcium ions concentration in the cytosol of muscle cells, might be one of the element in the ethiopatomechanism of essential hypertension development. The ATP-related ions pomp--SERCA2A regulates the intracellular calcium concentration. Mutations in the ATP2A2 gene coding the SERCA2A protein, has been associated with elevated calcium level in cardiomyocytes. The aim of the study was to analyze frequency of the M235T (c.704C>T) AGT gene polymorphism. The new mutations in the ATP2A2 gene was searched for in hypertensive patients, independently to the LVH presence in compare to the control group. 157 people participated in the study. Based on the echo-cardiographic examination participants were divided into subgroups: patients with essential hypertension (NT) and patients with NT and LVH. 50 healthy volunteers served as the control group. The frequency of the CC homozygotes, of the M235T (c.704C>T) AGT gene polymorphism, was the highest in the patients with essential hypertension and LVH in compare with patients without LVH (p = 0.67) and control group (p = 0.64). The value of the LVMI was the highest in CC carriers in compare to homozygotes TT (p = 0.33) and CT group (p = 0.66). In homozygotes TT as well as in the carriers of allel T, the elevated blood pressures value was detected. In the exon 15 of ATPA2A gene the new polymorphism A724A (C.2171G>A) was found. only the presence of GG and heterozygotes GA was detected in analyzed group. The frequency of GA genotype was significantly higher in control group vs patients with essential hypertension with (p = 0.05)/or without LVH (p = 0.04). The GA carriers had lower blood pressures values measured in doctor office as well as using ABPM method. The LVM as well as LVMI values were lower in group with mutated genotype GA in compare to GG group (p = 0.107 for LVM; p = 0.154 for LVMI). Results suggest a protective role of the c.2171G>A polymorphism of the ATP2A2 gene against the hypertension as well as LVH development. It seems also that c.704C>T polymorphism of AGT gene does not play crucial role in the essential hypertension development. |
| 11230286 | T+31C polymorphism of angiotensinogen gene and essential hypertension.A common variant at codon 235 of the angiotensinogen gene with methionine to threonine amino acid substitution (AGT M235T) has been reported as a genetic risk for essential hypertension. However, the frequency of AGT T235 was heterogeneous among races, and a positive association between AGT M235T and hypertension was not settled. To examine the association in a general population of Japanese (n=4013), we introduced the TaqMan polymerase chain reaction method and examined the relation between hypertension and T+31C polymorphism, which was in absolute linkage disequilibrium with AGT M235T. The C+31 allele of AGT was significantly associated with the positive family history of hypertension (FH) but not with the presence of hypertension or blood pressure. The subjects with CC tended to have hypertensive relatives, especially a hypertensive father or siblings, and its statistical significance was stronger in men. Adjustment of confounding factor did not alter the results of simple association study, suggesting that this positive association with FH is independent and significant. Our findings revealed that the TaqMan polymerase chain reaction method is a powerful tool for genetic association study with a large number of subjects and that AGT T+31C is significantly associated with paternal FH. |
| 18005249 | Age-associated loss of Sirt1-mediated enhancement of glucose-stimulated insulin secretion in beta cell-specific Sirt1-overexpressing (BESTO) mice.The Sir2 (silent information regulator 2) family of NAD-dependent deacetylases regulates aging and longevity across a wide variety of organisms, including yeast, worms, and flies. In mammals, the Sir2 ortholog Sirt1 promotes fat mobilization, fatty acid oxidation, glucose production, and insulin secretion in response to nutrient availability. We previously reported that an increased dosage of Sirt1 in pancreatic beta cells enhances glucose-stimulated insulin secretion (GSIS) and improves glucose tolerance in beta cell-specific Sirt1-overexpressing (BESTO) transgenic mice at 3 and 8 months of age. Here, we report that as this same cohort of BESTO mice reaches 18-24 months of age, the GSIS regulated by Sirt1 through repression of Ucp2 is blunted. Increased body weight and hyperlipidemia alone, which are observed in aged males and also induced by a Western-style high-fat diet, are not enough to abolish the positive effects of Sirt1 on beta cell function. Interestingly, plasma levels of nicotinamide mononucleotide (NMN), an important metabolite for the maintenance of normal NAD biosynthesis and GSIS in beta cells, are significantly reduced in aged BESTO mice. Furthermore, NMN administration restores enhanced GSIS and improved glucose tolerance in the aged BESTO females, suggesting that Sirt1 activity decreases with advanced age due to a decline in systemic NAD biosynthesis. These findings provide insight into the age-dependent regulation of Sirt1 activity and suggest that enhancement of systemic NAD biosynthesis and Sirt1 activity in tissues such as beta cells may be an effective therapeutic intervention for age-associated metabolic disorders such as type 2 diabetes. |
| 17502873 | UCP2 A55V variant is associated with obesity and related phenotypes in an aboriginal community in Taiwan.OBJECTIVE: Human uncoupling proteins 2 and 3 (UCP2 and UCP3) are two mitochondrial proteins that are involved in the control of metabolism of fatty acid and possibly protect against oxidative damage. The aim of this study was to analyze genetic associations of four polymorphisms of the UCP2 and UCP3 genes with insulin, leptin concentration and obesity in Taiwan aborigines. RESEARCH METHODS: Four polymorphisms were compared in 324 obese (body mass index (BMI) > or =30 kg/m(2)) and overweight (30>BMI > or =25 kg/m(2)) subjects, and 114 normal weight subjects (BMI <25 kg/m(2)) in an aboriginal community of southern Taiwan. Anthropometric characteristics and fasting levels of insulin, leptin, triglycerides and cholesterol were measured. RESULTS: Before and after adjusting for age distribution, only the Val55 allele in exon 4 of the UCP2 gene increased the risk of overweight and obesity (adjusted odds ratio (OR)=2.02, P=0.004) in comparison with Ala55. UCP2 V55V is also associated with higher fasting insulin levels than A55V (P=0.01) and A55A (P=0.04) in the obese/overweight group. Using the COCAPHASE program of the UNPHASED software, haplotype analysis of three single nucleotide polymorphisms (A55V-G866A-C-55T) revealed that A-G-C (73% in obese subjects and 77% in controls) was the most common haplotype and that the haplotype V-A-T (13% in obese subjects and 5% in controls) was significantly increased in obese and overweight subjects (BMI > or =25 kg/m(2)) (OR=2.62, P<0.001). DISCUSSIONS: UCP2 A55V variant might predispose to obesity and Val55 allele to confer population-attributable risk for 9.5% of obese disorders and increase insulin concentrations. The V-A-T haplotype within UCP2-UCP3 gene cluster is also significantly associated with obesity in Paiwan aborigines. |
| 18292186 | Uncoupling protein-2 modulates the lipid metabolic response to fasting in mice.Uncoupling protein-2 (UCP2) regulates insulin secretion by controlling ATP levels in beta-cells. Although UCP2 deficiency improves glycemic control in mice, increased expression of UCP2 interferes with glucose-stimulated insulin secretion. These observations link UCP2 to beta-cell dysfunction in type 2 diabetes with a perplexing evolutionary role. We found higher residual serum insulin levels and blunted lipid metabolic responses in fasted ucp2(-/-) mice, supporting the concept that UCP2 evolved to suppress insulin effects and to accommodate the fuel switch to fatty acids during starvation. In the absence of UCP2, fasting initially promotes peripheral lipolysis and hepatic fat accumulation at less than expected rates but culminates in protracted steatosis, indicating diminished hepatic utilization and clearance of fatty acids. We conclude that UCP2-mediated control of insulin secretion is a physiologically relevant mechanism of the metabolic response to fasting. |
| 18587729 | Further evidence for the association of MMP9 with nephropathy in type 2 diabetes and application of DNA pooling technology to candidate gene screening.BACKGROUND: Diabetic nephropathy is characterised by extracellular matrix (ECM) expansion, a key modulator of which is TGF-b1. Glucose-stimulated transcriptional activation of the TGF-b1 gene is an important component of the pathogenesis of nephropathy, following which latent TGF-b1 protein is synthesised. Matrix metalloproteinase 9 (MMP9) remodels the ECM and has been implicated in TGF-b1 activation. The ECM glycosaminoglycan hyaluronan (HA) influences TGF-b1 generation and can modulate its signal transduction activity; renal HA is synthesised by HA synthases HAS2 and HAS3. METHODS: We report the first screening of the genes encoding HAS2 and HAS3 for sequence variants predisposing to nephropathy in UK type 2 diabetes patients, together with the MMP9 and TGF-b1 genes. Also for the first time, we used validated DNA pools to carry out association analyses of single nucleotide polymorphisms on nephropathic and non-nephropathic cohorts from a total of 199 type 2 diabetes patients, to increase the throughput and decrease the cost of genotype analysis. RESULTS: None of the 23 single nucleotide polymorphisms analysed in DNA pools were found to be associated with diabetic nephropathy. However, genotyping of alleles at the MMP9 promoter microsatellite locus D20S838 in individual genomic DNA samples supported previous evidence of association between this locus and diabetic nephropathy. CONCLUSIONS: The use of DNA pooling technology increased the throughput and decreased the cost of our association analysis of nephropathy in our type 2 diabetes sample, which demonstrated sufficient sensitivity to support previous positive findings of association with a microsatellite in the MMP9 promoter region. |
| 18842989 | The MIF receptor CD74 in diabetic podocyte injury.Although metabolic derangement plays a central role in diabetic nephropathy, a better understanding of secondary mediators of injury may lead to new therapeutic strategies. Expression of macrophage migration inhibitory factor (MIF) is increased in experimental diabetic nephropathy, and increased tubulointerstitial mRNA expression of its receptor, CD74, has been observed in human diabetic nephropathy. Whether CD74 transduces MIF signals in podocytes, however, is unknown. Here, we found glomerular and tubulointerstitial CD74 mRNA expression to be increased in Pima Indians with type 2 diabetes and diabetic nephropathy. Immunohistochemistry confirmed the increased glomerular and tubular expression of CD74 in clinical and experimental diabetic nephropathy and localized glomerular CD74 to podocytes. In cultured human podocytes, CD74 was expressed at the cell surface, was upregulated by high concentrations of glucose and TNF-alpha, and was activated by MIF, leading to phosphorylation of extracellular signal-regulated kinase 1/2 and p38. High glucose also induced CD74 expression in a human proximal tubule cell line (HK2). In addition, MIF induced the expression of the inflammatory mediators TRAIL and monocyte chemoattractant protein 1 in podocytes and HK2 cells in a p38-dependent manner. These data suggest that CD74 acts as a receptor for MIF in podocytes and may play a role in the pathogenesis of diabetic nephropathy. |
| 22588935 | [Hepatic SIRT1 and UCP2 expressions in rats with type 2 diabetes mellitus and nonalcoholic fatty liver].OBJECTIVE: To observe the expression of SIRT1 and mitochondrial uncoupling protein 2 (UCP2) in the liver of rats with type 2 diabetes mellitus (T2DM) and nonalcoholic fatty liver (NAFLD) and explore the possible pathogenesis of T2DM and NAFLD. METHODS: Twenty-four male SD rat were randomized equally into control group and T2DM and NAFLD group (MC group), fed with standard diet and high-fat and high-sugar diet, respectively. At 12 weeks, the rats in MC group received a single dose of STZ (30 mg/kg) injected into the abdominal cavity for pancreatic islet destruction, and those in the control group received an equivalent volume of citric acid buffer. At 14 weeks, the body weight, FBG, hepatic function, blood lipid levels, FFAs, FINs and HOMA-IR of the rats were measured, and the liver pathology was examined with HE staining. The expression of SIRT1 and UCP2 in the rat liver was detected by immunohistochemistry and real-time quantitative PCR. RESULTS: At 14 weeks, FBG, ALT, AST, TC, TG, LDL-C, VLDL, FFAs, FINs and HOMA-IR were significantly higher and HDL-C was significantly lower in MC group than in the control group (P<0.05). Pathological examination showed good structural integrity of the liver in the control group, and the liver cells were closely arranged with rich cytoplasm and round cell nuclei; in MC group, moderate to severe fatty liver was detected, and the liver cells showed severe ballooning degeneration and contained lipid vacuoles in the cytoplasm. The expression of SIRT1 was significantly lower and UCP2 significantly higher in MC group than in the control group (P<0.05). CONCLUSION: The expression of SIRT1 is significantly lowered and UCP2 increased in the liver of rats with T2DM and NAFLD. |
| 10760499 | Activation of renal afferent pathways following furosemide treatment. II. Effect Of angiotensin blockade.The goal here and in the accompanying paper was to evaluate the two pathways used by the kidney to provide information to the central nervous system (CNS); e.g., the indirect, hormonal route via activation of the renin-angiotensin system and the direct pathway via activation of sympathetic afferents in the caudal thoracic spinal cord. Here, three experiments were designed to evaluate the actions of angiotensin elicited by subcutaneous injection of furosemide on neural activation of the CNS. The number of neurons immunocytochemically staining for the protein product (Fos) of the c-fos gene was used as an index of neuronal activation. In the first experiment, furosemide injection was preceded by treatment with a dose of Captopril, CAP, (an angiotensin-converting enzyme (ACE) inhibitor) that blocks the peripheral but not the central formation of angiotensin II. In the second experiment, furosemide injection was preceded by treatment with a higher dose of CAP; this dosage blocks the peripheral and central formation of angiotensin II. In the third experiment, furosemide injection was preceded by treatment with Losartan, a competitive receptor antagonist of type I angiotensin II receptors at a dose that would block central and peripheral angiotensin receptors. Control animals in each experiment received injections of vehicle (sterile isotonic saline) instead of furosemide. In each experiment, rats were sacrificed 1.75 h following furosemide or saline injection by transcardial perfusion and tissues were immunocytochemically processed for demonstration of Fos antigen. Rats receiving furosemide plus the low CAP dose showed more Fos-positive cells than control rats in the subfornical organ (SFO), organum vasculosum lamina terminalis (OVLT), supraoptic nucleus (SON), magnocellular region of the paraventricular nucleus, nucleus of the solitary tract (NTS), and caudal thoracic/rostral lumbar spinal cord dorsal horn. Rats receiving furosemide plus Losartan or furosemide plus the higher CAP dose did not show increased Fos immunoreactivity in any of the abovementioned structures relative to their respective control animals. We conclude that the receptor-mediated action of angiotensin II is in some way involved in the activation of the pathway that occurs in the SFO, OVLT, SON, and magnocellular region of the paraventricular nucleus (PVN) in response to furosemide treatment. It is possible that the furosemide-induced activation in the SON and PVN is not due to direct actions of angiotensin II on angiotensin receptors in those structures, but instead occurs synaptically as a result of inputs from the SFO and OVLT, which have themselves been activated directly by angiotensin II. In the accompanying paper, furosemide-induced activation in the NTS and caudal thoracic spinal cord is abolished by prior bilateral renal denervation, meaning that these neurons are likely part of a renal afferent pathway. Here, these structures did not elaborate Fos in animals injected with furosemide plus the high CAP dose or furosemide plus Losartan. Thus, the present results also suggest that the central blockade of the formation of angiotensin II or blockade of the actions of angiotensin II prevents in some way the activation of the renal afferent pathway mediated by the renal nerves (the direct pathway) in response to the actions of furosemide. Therefore, these results suggest that central angiotensin II is somehow involved in "priming" or increasing the sensitivity of the direct renal afferent pathway. Taken together with the accompanying paper, our results indicate that interruption of the direct pathway via renal denervation did not interfere with the elaboration of Fos in the lamina terminalis; in contrast, modification of the humoral renal afferent pathway can affect the sensitivity of the direct pathway. These results may have important implications for pathophysiological changes associated with fluid balance disorders including renal hypertension. |
| 11095476 | Influence of the M235T polymorphism of human angiotensinogen (AGT) on plasma AGT and renin concentrations after ethinylestradiol administration.The T235 allele of the angiotensinogen (AGT) gene is associated with plasma AGT concentration and pregnancy-induced hypertension. The aim of this study was to compare changes in the circulating renin-angiotensin system after short-term (2 days) and repeated (7 days) administration of 50 microg ethinylestradiol (EE) in homozygous normotensive men (TT and MM). After repeated EE administration, renin stimulation was induced by a single oral dose of 40 mg furosemide, followed by 50 mg captopril, 12 h later. The short-term administration of EE did not induce a significant differential genotype-dependent increase in AGT concentration. In the 7-day study, TT subjects had higher peak plasma AGT concentrations than MM subjects. The more pronounced AGT increase in TT subjects resulted in similar plasma renin activity at a lower plasma active renin concentration, with a higher plasma renin activity/active renin ratio. The difference between genotypes in renin secretion resulted in readjustment of angiotensins production. In conclusion, the T235 allele of the AGT gene is associated with greater stimulation of AGT secretion in plasma after EE administration. In the short-term, complete readjustment of the circulating renin-angiotensin system occurs, through a decrease in renin release, which blunts the effects of the increase in AGT concentration. |
| 12511525 | Angiotensinogen 235T allele "dosage" is associated with blood pressure phenotypes.The genetic mechanisms underlying interindividual blood pressure variation among humans may reflect, at least in part, clustering of functional gene variants belonging to complex blood pressure control systems. In this study, we investigated the association of specific functional gene variants of the renin-angiotensin system, ACE (I/D) and angiotensinogen (M/T) genes, with blood pressure phenotypes (systolic, mean, diastolic, and pulse pressure), in an ethnically mixed urban population in Brazil. Individuals (n=1421) were randomly selected from the general population of the Vitoria City Metropolitan area. Neither gender, age, smoking status, total cholesterol, triglycerides, HDL-cholesterol, LDL-cholesterol, VLDL-cholesterol, or diabetes was associated with ACE or AGT polymorphism in univariate analysis. No association was found between ACE variants and blood pressure phenotypes. However, a statistically significant association was revealed between the AGT 235T variant and all blood pressure phenotypes, consistent with an additive/codominant mode of action even after adjustment for age and gender (P<0.01). Genotypic analysis contemplating both ACE and AGT variants in the same model did not show any significant interaction between both genetic polymorphisms. In addition, the AGT 235T allele was significantly associated with hypertension in a recessive model, which remained as an independent risk factor for hypertension even after adjustment for age, gender, and ethnicity (OR, 1.33; 95% CI, 1.04 to 1.70). Taken together, these data indicate a linear relation between AGT 235T allele number ("dosage") and blood pressure in an ethnically mixed urban population and confirmed its role as an independent risk factor for hypertension for men and women when in homozygosity. |
| 15927971 | Increased cardiovascular morbidity and mortality in type 2 diabetes is associated with the glutathione S transferase theta-null genotype: a Go-DARTS study.BACKGROUND: Glutathione S-transferases (GSTs) modulate oxidative stress, and variation in GST genes has been associated with cardiovascular disease risk. We prospectively determined smoking-related cardiovascular morbidity by GST genotype in a large cohort of individuals with type 2 diabetes using a population-based diabetes research database (DARTS). METHODS AND RESULTS: We performed a cohort study of 2015 individuals with type 2 diabetes. Individuals were genotyped for the Ile105Val variant of GSTP1 and the deleted variants of GSTT1 and GSTM1. Clinical characteristics, smoking status, and incidence of subsequent cardiovascular events were obtained by examining the DARTS databases. Variation in the GSTP1 and GSTM1 genes was not associated with smoking-related risk of death or cardiovascular events. There was an increase in the rate of cardiovascular events in smokers lacking the GSTT1 gene compared with smokers with the GSTT1 gene intact (hazard ratio [HR], 1.96; P=0.001). This excess of cardiovascular events was due to both strokes (HR, 2.7; P=0.008) and myocardial infarctions (HR, 1.9; P=0.006). The rate of death as a result of a cardiovascular event was even more markedly increased in the GSTT1-null smokers (HR, 2.7; P=0.001), with a 2-fold increase in myocardial infarction fatality ratio. These effects translated into an increase in overall death and a decrease in age at death. We also found that the GSTT1- genotype was associated with progression of both diabetic retinopathy and nephropathy (P=0.005 and P=0.01, respectively), although we found little evidence for an interaction with smoking. CONCLUSIONS: Genetic absence of the GSTT1 enzyme is an independent and powerful predictor of premature vascular morbidity and death in individuals with type 2 diabetes. |
| 17785417 | SIRT1 promotes endothelium-dependent vascular relaxation by activating endothelial nitric oxide synthase.Reduced caloric intake decreases arterial blood pressure in healthy individuals and improves endothelium-dependent vasodilation in obese and overweight individuals. The SIRT1 protein deacetylase mediates many of the effects of calorie restriction (CR) on organismal lifespan and metabolic pathways. However, the role of SIRT1 in regulating endothelium-dependent vasomotor tone is not known. Here we show that SIRT1 promotes endothelium-dependent vasodilation by targeting endothelial nitric oxide synthase (eNOS) for deacetylation. SIRT1 and eNOS colocalize and coprecipitate in endothelial cells, and SIRT1 deacetylates eNOS, stimulating eNOS activity and increasing endothelial nitric oxide (NO). SIRT1-induced increase in endothelial NO is mediated through lysines 496 and 506 in the calmodulin-binding domain of eNOS. Inhibition of SIRT1 in the endothelium of arteries inhibits endothelium-dependent vasodilation and decreases bioavailable NO. Finally, CR of mice leads to deacetylation of eNOS. Our results demonstrate that SIRT1 plays a fundamental role in regulating endothelial NO and endothelium-dependent vascular tone by deacetylating eNOS. Furthermore, our results provide a possible molecular mechanism connecting the effects of CR on the endothelium and vascular tone to SIRT1-mediated deacetylation of eNOS. |
| 21421655 | Renin-angiotensin-aldosterone system genotypes and haplotypes affect the susceptibility to nephropathy in type 2 diabetes patients.BACKGROUND: The association between renin C-4063T and angiotensinogen (AGT) T174M, M235T, and A-6G polymorphisms with diabetic nephropathy (DN) was investigated in Tunisian type 2 diabetes (T2DM) patients. METHODS: Study subjects comprised 917 T2DM patients (405 normoalbuminuric, 329 microalbuminuric and 185 macroalbuminuric). Genotyping was done by PCR-RFLP. RESULTS: Renin C-4063T allele and genotype frequencies were comparable between DN cases and normoalbuminuric controls. Although AGT 235T and -6G allele, and 235T/T and -6G/G genotype frequencies were higher in DN compared to normoalbuminuric patients, they were comparable between microalbuminuric or macroalbuminuric patients. Three-locus AGT haplotype analysis (A-6G/T174M/M235T) identified DN-protective (ATM, AMM, GTM) and DN-susceptible (GTM, ATT, GMT and AMT) haplotypes, and demonstrated enrichment of GTT haplotype in macroalbuminuric compared to microalbuminuric or normoalbuminuric patients. Regression analysis confirmed negative (AMM) and positive (GTM, ATT, GMT, AMT) association of AGT haplotypes with microalbuminuria, and negative (AMM) and positive (GTM and ATT) association of AGT haplotypes with macroalbuminuria. None of the AGT haplotypes was associated with DN severity. CONCLUSIONS: Genetic variation at the AGT gene influences the risk of nephropathy in T2DM patients but not extent of DN severity, and thus represents a potential DN genetic susceptibility locus worthy of replication. |
| 21500980 | Association of the angiotensinogen M235T and APO E gene polymorphisms in Turkish type 2 diabetic patients with and without nephropathy.BACKGROUND: Diabetic nephropathy (DN) is a leading cause of diabetes-related morbidity and mortality. The aim of this study was to evaluate the relationship of AGT M235T and apoprotein E (APO E) gene polymorphism with DN in Turkish patients of Type 2 diabetes, and to compare genotype and allele distributions among DN patients, non-DN patients, and healthy controls. METHODS: AGT M235T and APO E genotype and allele analysis were performed in 111 DN patients, 108 non-DN patients, 106 healthy control subjects for APO E genotype, and 100 for AGT M235T genotype polymorphism. APO E and AGT M235T genotype were determined by RFLP-PCR. Results: The frequencies of APO E epsilon2/3, epsilon 3/3, epsilon 3/4 genotypes were 22.7%, 60%, 60%, respectively, among DN patients and 6.6%, 80%, 10.4%, respectively (p < 0.001), in the non-DN patients. The frequencies of AGT M235T MM, MT, TT genotypes among the same groups were 17%, 46%, 37% and 21%, 63%, 16%, respectively (p < 0.02). Having the epsilon2/3 genotype and TT genotype increased the risk for DN nephropathy [4.8-fold (95% CI: 1.94-11.67), 2.9-fold (95% CI: 1.27-6.69), respectively]. CONCLUSION: Our study has shown that AGT M235T TT genotype and APO E epsilon 2/3 genotype may be linked to a risk for DN among Turkish population. |
| 23652801 | Uncoupling protein 2 deficiency mimics the effects of hypoxia and endoplasmic reticulum stress on mitochondria and triggers pseudohypoxic pulmonary vascular remodeling and pulmonary hypertension.RATIONALE: Mitochondrial signaling regulates both the acute and the chronic response of the pulmonary circulation to hypoxia, and suppressed mitochondrial glucose oxidation contributes to the apoptosis-resistance and proliferative diathesis in the vascular remodeling in pulmonary hypertension. Hypoxia directly inhibits glucose oxidation, whereas endoplasmic reticulum (ER)-stress can indirectly inhibit glucose oxidation by decreasing mitochondrial calcium (Ca(2)(+)m levels). Both hypoxia and ER stress promote proliferative pulmonary vascular remodeling. Uncoupling protein 2 (UCP2) has been shown to conduct calcium from the ER to mitochondria and suppress mitochondrial function. OBJECTIVE: We hypothesized that UCP2 deficiency reduces Ca(2)(+)m in pulmonary artery smooth muscle cells (PASMCs), mimicking the effects of hypoxia and ER stress on mitochondria in vitro and in vivo, promoting normoxic hypoxia inducible factor-1alpha activation and pulmonary hypertension. METHODS AND RESULTS: Ucp2 knockout (KO)-PASMCs had lower mitochondrial calcium than Ucp2 wildtype (WT)-PASMCs at baseline and during histamine-stimulated ER-Ca(2)(+) release. Normoxic Ucp2KO-PASMCs had mitochondrial hyperpolarization, lower Ca(2)(+)-sensitive mitochondrial enzyme activity, reduced levels of mitochondrial reactive oxygen species and Krebs' cycle intermediates, and increased resistance to apoptosis, mimicking the hypoxia-induced changes in Ucp2WT-PASMC. Ucp2KO mice spontaneously developed pulmonary vascular remodeling and pulmonary hypertension and exhibited a pseudohypoxic state with pulmonary vascular and systemic hypoxia inducible factor-1alpha activation (increased hematocrit), not exacerbated further by chronic hypoxia. CONCLUSIONS: This first description of the role of UCP2 in oxygen sensing and in pulmonary hypertension vascular remodeling may open a new window in biomarker and therapeutic strategies. |
| 10997636 | Gene expression of angiotensinogen in adipose tissue of obese patients.Recently, the genes of components of the renin-angiotensin system (RAS), namely angiotensinogen (AGT), angiotensin converting enzyme and angiotensin II receptor have been described in adipose tissue. In animal models the angiotensinogen in adipose tissue has been implicated in the pathogenesis of metabolic alterations and hypertension associated with obesity. The aim of our study was to evaluate the AGT gene expression both in visceral and subcutaneous adipose tissue in obese patients and lean subjects. AGT mRNA levels were measured by reverse transcriptase polymerase chain reaction (RT-PCR) using specific primers. AGT mRNA was expressed at variable levels in obese patients. It was significantly greater in visceral than in subcutaneous adipose tissue. Positive and significant correlation was found between the expression of AGT in visceral adipose tissue and BMI. These data suggest that angiotensinogen may be determinant of fat distribution and may be involved in the plurimetabolic syndrome of central obesity. |
| 18637188 | RAS gene polymorphisms, classical risk factors and the advent of coronary artery disease in the Portuguese population.BACKGROUND: Several polymorphisms within the renin-angiotensin system cluster of genes have been associated with the advent of coronary artery disease (CAD) or related pathologies. We investigated the distribution of 5 of these polymorphisms in order to find any association with CAD development and distinguish if any of the biochemical and behavioural factors interact with genetic polymorphisms in the advent of the disease. METHODS: ACE I/D (rs4340), ACE A11860G (rs4343), AT1R A1166C (rs5186), AGT T174M (rs4762) and AGT M235T (rs699) gene polymorphisms were PCR-RFLP analysed in 298 CAD patients and 510 controls from Portugal. Several biochemical and behavioural markers were obtained. RESULTS: ACE I/D DD and ACE11860 GG genotypes are risk factors for CAD in this population. The simultaneous presence of ACE I/D I and ACE11860 A alleles corresponds to a significant trend towards a decrease in CAD incidence. We found several synergistic effects between the studied polymorphisms and classical risk factors such as hypertension, obesity, diabetes and dyslipidaemia: the presence of the DD genotype of ACE I/D (and also ACE11860 GG) increases the odds of developing CAD when associated to each one of these classical risk factors, particularly when considering the male and early onset CAD subgroup analysis; AGT235 TT also increases the CAD risk in the presence of hypertension and dyslipidaemia, and AT1R1166 interacts positively with hypertension, smoking and obesity. CONCLUSION: ACE polymorphisms were shown to play a major role in individual susceptibility to develop CAD. There is also a clear interaction between RAS predisposing genes and some biochemical/environmental risk factors in CAD onset, demonstrating a significant enhancement of classical markers particularly by ACE I/D and ACE11860. |
| 16202290 | [Correlation between 4G and 5G genetypes distribution of plasminogen activator inhibitor-1 gene polymorphism in its promoter region with polycystic ovarian syndrome].OBJECTIVE: To investigate the correlation of 4G and 5G genetypes distribution of plasminogen activator inhibitor-1 (PAI-1) gene polymorphism in its promoter region with polycystic ovary syndrome (PCOS). METHODS: In 101 Chinese PCOS patients and 42 women as control, 4G and 5G polymorphisms of PAI-1 gene were detected with PCR-restriction fragment length polymorphism (RFLP). Pregnant history, body mass index (BMI), waist-hip ratio (WHR) were collected and Homastasis Model Assessment score for insulin resistant (Homa-IR) and insulin sensitive index (ISI) in PCOS groups were calculated. RESULTS: The distributions of PAI-1 gene polymorphisms 4G type (4G/4G genetype) and 5G type (5G/5G, 4G/5G genetype) were different between the PCOS group and the control. The PCOS group had higher 4G type 57% (58/101) distribution than that of the control group 38% (16/42); 5G type is vise verse (P < 0.05). PCOS patients were divided into obese and non-obese sub-groups according to BMI. There were significant lower Homa-IR and higher ISI in non-obese subgroup than that in obese group (P < 0.05; P < 0.01). 68% (36/53) 4G type distribution in the non-obese is higher than that [48% (23/48)] in obese sub-group (P < 0.05). There were higher 4G type distribution 79% (11/14) and lower 5G type distribution 21% (3/14) in spontaneous miscarriage group than that of in non-spontaneous miscarriage group 38% (5/13) and 62% (8/13) among patients who impregnated at least once (n = 27, P < 0.05). CONCLUSION: PAI-1 gene polymorphism 4G genetype may be correlated with PCOS in Chinese women, especially in PCOS patients with non-obese PCOS and spontaneous miscarriage. |
| 18409009 | The M235T polymorphism of the AGT gene modifies the risk of coronary artery disease associated with the presence of hypercholesterolemia.OBJECTIVE: Atherosclerosis is an inflammatory disease resulting from interactions between various genetic and non-genetic factors. Angiotensinogen gene (AGT) belongs to polymorphic candidate genes. Recent evidence show that many traditional risk factors of coronary artery disease (CAD) influence synthesis of AGT. This report focuses on the interactions between M235T polymorphism of AGT gene and traditional risk factors of CAD. MATERIAL AND METHODS: 255 subjects, including 158 patients with angiographically confirmed CAD and 97 blood donors without history of cardiovascular diseases were studied. M235T polymorphism of the AGT gene was genotyped using PCR-RFLP method. To determine the possible interactions of AGT genotypes and traditional risk factors of CAD the attributable proportion due to interaction (AP) and synergy models were used. RESULTS: The frequency of 235T allele carriers was significantly higher in patients than in controls (77.8 vs. 62.9, OR = 2.20, 95% CI; 1.10-4.40, P = 0.026, in multivariate logistic regression model). We found the existence of interaction between the 235T allele carrier-state and hypercholesterolemia (total cholesterol > or = 5 mmol/l) increasing the risk of CAD (SI = 3.39, 95% CI; 1.33-8.66, AP = 0.65, 95% CI; 0.39-0.91). The 235T allele also interacted with elevated LDL cholesterol levels (> or = 3 mmol/l) (AP = 0.49, 95% CI; 0.20-0.96), but not with the hypertension, overweight/ obesity and cigarette smoking. CONCLUSION: The 235T allele increases the risk of CAD associated with the presence of hypercholesterolemia. |
| 20361261 | Angiotensin converting enzyme I/D, angiotensinogen M235T and AT1-R A/C1166 gene polymorphisms in patients with acromegaly.Acromegaly is associated with increased morbidity and mortality related to cardiovascular disease. Hypertension is one of the most common cardiovascular risk factors in acromegalic patients. The aim of this study was to investigate association between the frequencies of angiotensin converting enzyme (ACE) I/D, angiotensinogen (AGT) M235T and the angiotensin II type 1 receptor (AT1-R) A/C1166 gene polymorphisms and some clinical parameters of acromegalic patients. Total of 33 acromegalic patients and 63 controls were enrolled to study. We determined the ACE I/D, AGT M235T and AT1-R A/C1166 gene polymorphisms. Serum insulin, glucose, triglyceride, HDL-cholesterol, LDL-cholesterol, growth hormone and Insulin-like growth factor I (IGF-I) levels of subjects were analyzed. The frequencies of ACE and M235T AGT genotype were not significantly different between control and patients. The distribution of AT1R A/C1166 genotypes was significantly different between patients and control subjects (P=0.016). None of the three ACE genotypes, DD, ID and II displayed significant difference in acromegalic patients. A significant difference in systolic blood pressure and the serum IGF-I levels among the three AGT genotype, MM, MT and TT genotypes was found in patient group. Individuals with MT genotypes had significantly higher serum IGF-I levels and systolic blood pressure than MM and TT genotype subjects, P<0.05. In addition, serum triglyceride and HDL levels differed significantly between MM and MT genotypes, P<0.05. However, systolic blood pressure of patients with CC genotypes was found to be significantly higher than AA genotypes individuals in acromegaly group, P<0.05. It can be said that the angiotensinogen MT and AT1R CC1166 genotype carriers may have more risk than other genotypes in the development of hypertension in acromegaly. |
| 21985785 | Sirt1 enhances skeletal muscle insulin sensitivity in mice during caloric restriction.Skeletal muscle insulin resistance is a key component of the etiology of type 2 diabetes. Caloric restriction (CR) enhances the sensitivity of skeletal muscle to insulin. However, the molecular signals within skeletal muscle linking CR to improved insulin action remain largely unknown. Recently, the mammalian ortholog of Sir2, sirtuin 1 (Sirt1), has been identified as a potential transducer of perturbations in cellular energy flux into subsequent metabolic adaptations, including modulation of skeletal muscle insulin action. Here, we have demonstrated that CR increases Sirt1 deacetylase activity in skeletal muscle in mice, in parallel with enhanced insulin-stimulated phosphoinositide 3-kinase (PI3K) signaling and glucose uptake. These adaptations in skeletal muscle insulin action were completely abrogated in mice lacking Sirt1 deacetylase activity. Mechanistically, Sirt1 was found to be required for the deacetylation and inactivation of the transcription factor Stat3 during CR, which resulted in decreased gene and protein expression of the p55alpha/p50alpha subunits of PI3K, thereby promoting more efficient PI3K signaling during insulin stimulation. Thus, these data demonstrate that Sirt1 is an integral signaling node in skeletal muscle linking CR to improved insulin action, primarily via modulation of PI3K signaling. |
| 21988197 | Angiotensinogen gene haplotype is associated with the prevalence of Japanese non-alcoholic steatohepatitis.Aim: Non-alcoholic steatohepatitis (NASH) patients frequently have hypertension, which is considered to be an important predictive factor for the subsequent development of hepatic fibrosis. The renin-angiotensin system is also known to contribute to the progression of NASH. Various types of functional single-nucleotide polymorphisms (SNPs) involved in the development of NASH have been proposed. Angiotensinogen (AGT) gene SNPs related to cardiovascular diseases have been reported. We aimed to evaluate the involvement of the AGT gene haplotype in Japanese NASH patients. Methods: Previously described genotypes of SNPs of the AGT gene, rs4762 C/T polymorphism (T207M), rs699 C/T polymorphism (T268M), and rs7079 C/A polymorphism (C11537A), were determined in 124 Japanese biopsy-proven NASH patients and 150 healthy volunteers (controls). Results: The allele and genotype frequencies in rs4762 and rs699 SNPs in NASH patients were similar to those in controls, while the frequency of the A allele and A/- genotype in rs7079 SNPs were much higher in NASH patients than in controls. In addition, the 3-SNP haplotype CTA was significantly over-represented in NASH patients compared with controls. Regarding clinical features of NASH patients, diastolic blood pressures in patients with the CTA/- genotype were much higher than in patients with other genotypes. Conclusions: We found a 3-SNP haplotype of the AGT gene that is involved in the development of NASH and influences hypertension in NASH patients. These results provide new insight into the therapy of NASH patients with the CTA haplotype using ACE inhibitors or angiotensin II type 1 receptor blockers. |
| 22974788 | Association of angiotensinogen (M235T) gene polymorphism with blood pressure lowering response to angiotensin converting enzyme inhibitor (Enalapril).PURPOSE: It has been suggested that genetic backgrounds, which have an association with essential hypertension, may also determine the responsiveness to ACE inhibitor. We determined the association of angiotensinogen (M235T) gene polymorphism with essential hypertension and the relationship between polymorphism in the angiotensinogen (M235T) gene and blood pressure response to ACE inhibitor (Enalapril) in patients with essential hypertension from northern Indian subjects. METHODS: 250 patients with essential hypertension and 250 normal healthy controls from Delhi and surrounding areas were recruited for the investigation. Blood pressure was recorded before and after 6 weeks of treatment with ACE inhibitors, Enalapril. Genotyping were carried out by polymerase chain reaction and Restriction fragment length polymorphism technique. RESULTS: Statistically significant association of T allele was observed with essential hypertension [x2 = 14.67, p = 0.00013, Odds ratio = 1.76 (1.3-2.32) at 95% CI], the relative risk at 95% CI being 1.28 (1.2-1.54). The decrease in systolic blood pressure and diastolic blood pressure after six weeks of treatment of the patients carrying TT genotype (SBP = 26 +/- 17.4 mmHg, DBP = 14.83 +/- 7.6 mmHg) were greater than the groups carrying MT (SBP = 3.0 +/- 7.8 mmHg, DBP = 6.2 +/- 3.0 mmHg) and MM genotypes (SBP = 1.2 +/- 0.8 mmHg, DBP = 0.10 +/- 12.1 mm Hg. CONCLUSIONS: The angiotensinogen (M235T) gene polymorphism is significantly associated with essential hypertension. Patients carrying TT genotype had higher blood pressure lowering response when treated with ACE inhibitor, Enalapril than those carrying MM and MT genotypes suggesting that the T allele may be a possible genetic marker for essential hypertension. |
| 11577832 | Angiotensin-related genes involved in essential hypertension: allelic distribution in an Italian population sample.BACKGROUND: Blood pressure is a quantitative multifactorial trait influenced by environmental and genetic determinants. Although several candidate genes have been associated with the development of essential hypertension, the mechanisms of individual susceptibility still remain unclear. Knowledge on the distribution of genetic polymorphisms in different populations is fundamental for the assessment of the predictive value of genetic variation. METHODS: We genotyped 300 healthy normotensive subjects from the Italian population for three polymorphisms, at the angiotensinogen (AGT, M and T), angiotensin II type 1 receptor (ATIR, A and C) and angiotensin-converting enzyme (ACE, D and I) genes. Polymorphisms were analyzed by polymerase chain reaction and restriction enzyme digestion. Statistical analysis was performed to verify the agreement with the Hardy-Weinberg equilibrium. RESULTS: The observed allelic distribution was in accordance with estimates reported for Caucasian populations. Variant allelic frequencies were 0.36 for the T and C alleles at the AGT andAT1R locus and 0.47 for the I allele of the ACE gene. AT1R and ACE genotype frequencies were in Hardy-Weinberg equilibrium, while there was a deviation of the AGT genotypes from those predicted by the equation. CONCLUSIONS: The studied polymorphisms are largely distributed in the Italian population sample, with a frequency of homozygous subjects for mutant alleles ranging from 9 to 22%. Epidemiology of mutations in the genes involved in blood pressure regulation provides tools to evaluate susceptibility to hypertension. |
| 12044476 | Down-regulation of basal Fos expression at nucleus tractus solitarii underlies restoration of baroreflex response after antihypertensive treatment in spontaneously hypertensive rats.Antihypertensive therapy not only normalizes the elevated blood pressure but also restores the reduced baroreceptor reflex response associated with hypertension, although the underlying mechanism is not fully understood. We assessed the hypothesis that a reversal of the enhanced basal Fos expression seen during hypertension in nucleus tractus solitarii, the terminal site of baroreceptor afferents, underlies the restoration of baroreceptor reflex sensitivity after antihypertensive treatment. Male adult spontaneously hypertensive or normotensive Wistar-Kyoto rats received for 3 weeks captopril (100 mg/kg/day) added to their drinking water. Evaluated subsequently under pentobarbital anesthesia, captopril-treated spontaneously hypertensive rats exhibited significantly lowered systolic blood pressure and restoration of the sensitivity in baroreceptor reflex control of heart rate to levels comparable with Wistar-Kyoto rats. Reverse transcription-polymerase chain reaction analysis and immunohistochemical evaluation revealed concomitant down-regulation of basal expression in nucleus tractus solitarii of c-fos gene at both mRNA and protein levels. Captopril treatment, on the other hand, elicited no discernible effect on systolic blood pressure, cardiac baroreceptor reflex sensitivity or basal expression of Fos protein at the nucleus tractus solitarii of normotensive Wistar-Kyoto rats. From these findings we suggest that a down-regulation of basal Fos expression in nucleus tractus solitarii may contribute to the restoration of baroreceptor reflex sensitivity in spontaneously hypertensive rats that received antihypertensive treatment such as captopril. |
| 14643574 | Angiotensinogen Met235Thr polymorphism, angiotensin-converting enzyme inhibitor therapy, and the risk of nonfatal stroke or myocardial infarction in hypertensive patients.The ThrThr genotype of the angiotensinogen (AGT) Met235Thr polymorphism has been associated with elevated AGT levels, hypertension, increased heart disease risk, and improved blood pressure (BP) response to angiotensin-converting enzyme (ACE) inhibitors. We hypothesized that risk of stroke or myocardial infarction (MI) associated with ACE inhibitor use varies by AGT genotype, with a larger protective effect of ACE inhibitors in individuals with the ThrThr genotype than individuals who are carriers of the Met allele. METHODS: We conducted a population-based case-control study. Participants were health maintenance organization members aged 30 to 79 years with treated hypertension. Those who survived incident stroke (n = 116) or MI (n = 208) during the study period were designated as cases. Control subjects (n = 717) were randomly sampled and frequency-matched to MI cases on age, sex, and calendar year. Health history, medication use, and AGT genotype were assessed. RESULTS: ThrThr genotype was present in 21% of stroke cases, 26% of MI cases, and 19% of control subjects. Compared with nonuse, ACE inhibitor use was associated with lower stroke risk among Thr homozygotes (odds ratio [OR] = 0.37, 95% CI = 0.14 to 0.99) than among Met carriers (OR = 1.4, 95% CI = 0.88 to 2.4; P for interaction =.02). Compared with nonuse, ACE inhibitor use was associated with similar MI risk among Thr homozygotes (OR = 0.90, 95% CI = 0.62 to 1.3) and among Met carriers (OR = 1.2, 95% CI = 0.60 to 2.5; P for interaction = 0.5). CONCLUSIONS: In this hypertensive population, the association of ACE inhibitor use with risk of nonfatal stroke varied by genotype. The protective association between ACE inhibitor use and nonfatal stroke risk among individuals with ThrThr genotype was not observed for nonfatal MI. |
| 16164224 | [Anti-AP-1 treatment].This review emphasizes our first discovery on the contribution of over-activation of c-fos gene to the pathogenesis of rheumatoid joint destruction. In particular, c-Fos signalling was required for increased activity of synovial mesenchymal cells which finally leads to rheumatoid joint destruction and peri-articular osteoporosis. Over-activation of c-fos via Wee1 kinase is responsible for tumor-like synovial over-growth. Our team designed anti-c-Fos drugs that specifically inhibit action of c-Fos at the AP-1 consensus sequence by using a computer-assisted drug design, which was the front-runner work executed in Japan. |
| 22387727 | Renin-angiotensin system genes polymorphism in Egyptians with premature coronary artery disease.Genetics polymorphism of the renin-angiotensin system (RAS) affects the pathogenesis of atherosclerosis and associated with coronary artery disease (CAD). We aimed to investigate the association between the RAS genes and premature CAD (PCAD) in Egyptians. 116 patients with PCAD, 114 patients with late onset CAD and 119 controls were included in the study. Angiotensin converting enzyme (ACE), angiotensin II receptor type 1 (ATR1) and angiotensinogen (AGT) genes polymorphisms were analyzed by polymerase chain reaction (PCR). We found that ACE DD, AGT TT and ATR1 CC increased the risk of PCAD by 2.7, 2.8 and 2.86 respectively). Smoking, hypertension, diabetes, total cholesterol, triglycerides and LDL cholesterol were independent risk factors for the development of PCAD. We conclude that the ACE DD, AGT TT and ATR1 CC genotypes may increase the susceptibility of an individual to have PCAD. The coexistence of CAD risk factors with these risky RAS genotypes may lead to the development of PCAD in Egyptian patients. |
| 22908267 | Oral advanced glycation endproducts (AGEs) promote insulin resistance and diabetes by depleting the antioxidant defenses AGE receptor-1 and sirtuin 1.The epidemics of insulin resistance (IR) and type 2 diabetes (T2D) affect the first world as well as less-developed countries, and now affect children as well. Persistently elevated oxidative stress and inflammation (OS/Infl) precede these polygenic conditions. A hallmark of contemporary lifestyle is a preference for thermally processed nutrients, replete with pro-OS/Infl advanced glycation endproducts (AGEs), which enhance appetite and cause overnutrition. We propose that chronic ingestion of oral AGEs promotes IR and T2D. The mechanism(s) involved in these findings were assessed in four generations of C57BL6 mice fed isocaloric diets with or without AGEs [synthetic methyl-glyoxal-derivatives (MG(+))]. F3/MG(+) mice manifested increased adiposity and premature IR, marked by severe deficiency of anti-AGE advanced glycation receptor 1 (AGER1) and of survival factor sirtuin 1 (SIRT1) in white adipose tissue (WAT), skeletal muscle, and liver. Impaired 2-deoxy-glucose uptake was associated with marked changes in insulin receptor (InsR), IRS-1, IRS-2, Akt activation, and a macrophage and adipocyte shift to a pro-OS/inflammatory (M1) phenotype. These features were absent in F3/MG(-) mice. MG stimulation of 3T3-L1 adipocytes led to suppressed AGER1 and SIRT1, and altered InsR, IRS-1, IRS-2 phosphorylation, and nuclear factor kappa-light chain enhancer of activated B cells (Nf-kappaB) p65 acetylation. Gene modulation revealed these effects to be coregulated by AGER1 and SIRT1. Thus, prolonged oral exposure to MG-AGEs can deplete host-defenses AGER1 and SIRT1, raise basal OS/Infl, and increase susceptibility to dysmetabolic IR. Because exposure to AGEs can be decreased, these insights provide an important framework for alleviating a major lifestyle-linked disease epidemic. |
| 22995745 | Genetic association of adipokine and UCP2 polymorphism with recurrent miscarriage among non-obese women.The adipokines produced from adipose tissues influence energy homeostasis, resulting in alterations of the adipokine concentrations. This process may be associated with fertility impairment, resulting in recurrent miscarriage. The present study investigated whether there was any association between the UCP2 45-bp indel polymorphism and the adipokine gene polymorphisms, namely leptin 2549 (C/A), adeponectin 276 (G/T) and 45 (T/G) and resistin 420 (C/G) in 200 non-obese recurrent miscarriage patients and 300 ethnically matched negative controls. These markers were studied using gene-specific PCR single specific primer and restriction fragment length polymorphism. For leptin 2549 and adeponectin 276, the A allele and G allele showed 3.42-fold (P=0.0001) and 1.36-fold (P=0.036) increased risk of recurrent miscarriage, respectively. Combined analysis of UCP2 45-bp indel and leptin 2549 showed U0-L0 and U1-L0 variants to be at 2- and 3-fold increased associative risk, respectively. Combined analysis of leptin 2549 and adeponectin 276 showed L0-D0 and L0-D1 variants to be at 2- and 4-fold increased associative risk, respectively. The combination U1-L0-D1-A1-R1 was 4.39-fold higher (P=0.0007) among recurrent miscarriage patients. In conclusion, the results highlight the role of the studied adipokine and UCP2 polymorphisms in recurrent miscarriage among the North Indian non-obese population. Pregnancy invokes a large shift in maternal metabolism. The normal concentrations of adipokines, which maintain the integrity of the hypothalamus-pituitary-gonadal axis, regular ovulatory processes and successful embryo implantation, are altered because of the influence of energy homeostasis, which in turn leads to fertility impairment and recurrent miscarriage of unknown aetiology. Recurrent miscarriage is reported in higher frequency among obese women. The UCP2 45-bp indel polymorphism and the adipokine gene polymorphisms namely leptin 2549 (C/A), adeponectin 276 (G/T), adeponectin 45 (T/G) and resistin 420 (C/G) have been shown to be associated with obesity. Most of the adipokine-related studies done previously have taken into consideration the metabolic function and obesity. However, there exist very few studies to evaluate the role of adipokines in non-obese recurrent miscarriage with no cause of repeated pregnancy losses. The present study focused at evaluating the independent effect of these single-nucleotide polymorphisms in non-obese women undergoing recurrent miscarriage. |
| 9403547 | Theodore Cooper Memorial Lecture. A mouse view of hypertension.Essential hypertension probably results from combinations of genetic variations, not necessarily the same in all afflicted persons, which individually may not cause sufficient deviation from normality to be significantly harmful. Genes contributing to hypertension are being sought by analytic experiments aimed at identifying candidate genes associated or segregating with the phenotype in humans and animals and by synthetic experiments in which changes are made in candidate genes in animals and their effects on blood pressure are determined. We have used gene targeting to vary the amounts of angiotensinogen and angiotensin-converting enzyme (ACE) synthesized from their genes (Agt and Ace). These "gene titration" experiments establish that changes in Agt gene expression cause changes in the blood pressures of mice. Surprisingly, quantitative changes in Ace gene expression over a threefold range do not affect blood pressures. Computer simulations with a simple version of the renin-angiotensin system predict that changes in Agt function alter the steady state levels of both angiotensin I (Ang I) and angiotensin II (Ang II). In contrast, modest changes in Ace function alter Ang I levels considerably but scarcely affect Ang II levels. Simulations over the ranges of ACE levels that can be achieved with ACE inhibitors predict that Ang II levels will decrease only when Ang I levels have plateaued. Comparisons of the computer simulations with our genetic experiments and with prior work of others using wide dose ranges of ACE inhibitor show a satisfactory agreement and help reconcile the apparent contradictions between the genetic and pharmacological experiments. |
| 10365380 | [Genetic polymorphisms of the renin-angiotensin system and essential hypertension].BACKGROUND: The renin-angiotensin system (RAS) plays an important role in blood pressure (BP) regulation. A number of RAS polymorphisms have been linked to essential hypertension (EH), but there is uncertainty about this association in other studies. We examined whether the insertion/deletion (I/D) polymorphism of the angiotensin converting enzyme (ACE) gene, and the M235T and T174M polymorphisms of the angiotensinogen (AGT) gene are associated with EH in a Spanish sample of hypertensive patients. MATERIAL AND METHODS: We studied 75 patients with EH (BP > 160/100 mmHg), aged 55 (8.5) years, 30 males, systolic BP (SBP) 182 +/- (22.1) mmHg, diastolic BP (DBP) 109 +/- (9.9) mmHg (mean [SD]) and a strong family history of the disease. As a control group, 75 healthy subjects with no family history of cardiovascular disease were studied. The polymorphisms were determined by PCR amplification of genomic DNA, followed by enzyme digestion for the AGT gene polymorphisms. RESULTS: The genotype distribution and the frequencies of the alleles of the three RAS polymorphisms were similar in hypertensive and control subjects. In addition, we did not find any compound effect of the I/D ACE gene and M235T AGT gene polymorphisms on BP levels in hypertensive and control subjects. CONCLUSIONS: In this sample, the contribution of the ACE I/D polymorphism and the AGT M235T and T174M polymorphisms in the development of EH seems to be less important than previously estimated. |
| 11816701 | Role of the 4G/5G polymorphism of PaI-1 gene promoter on PaI-1 levels in obese patients: influence of fat distribution and insulin-resistance.As PAI-1, a cardiovascular risk factor linked to insulin-resistance, may be influenced by a 4G/5G gene polymorphism in disease states, we studied both PAI-1 plasma concentration (PAI-1:Ag) and 4G/5G polymorphism, and their relationship with anthropometric and endocrinemetabolic parameters in 93 obese patients and 79 lean normal subjects. In obese patients PAI-1:Ag levels were significantly increased, namely in males and in those with central obesity, and tightly related to the insulin-resistance parameters. In obese patients the 4G/5G polymorphism was a determinant of PAI-1:Ag levels, which were highest in 4G/4G, intermediate in 4G/5G and lowest in 5G/5G genotype carriers. PAI-1:Ag levels were significantly associated with most of anthropometric and endocrine-metabolic parameters only in 4G allele obese carriers. Moreover, only in patients with central obesity was the relationship between genotype and PAI-1 concentration maintained, with the highest levels in the 4G/4G patients. In each genotype subset of patients with central, but not peripheral, obesity PAI-1:Ag levels were significantly increased compared to their lean counterparts. In conclusion, the 4G/5G polymorphism may influence PAI-1 expression in obesity, with a crucial role in central but not peripheral adiposity. Since subjects with central obesity are at high risk for cardiovascular disease, the effects of the 4G/5G polymorphism on PAI-1 concentration may further enhance this risk. |
| 12118911 | T+31C polymorphism of angiotensinogen gene and nocturnal blood pressure decline: the Ohasama study.BACKGROUND: We assessed the association between several polymorphisms of angiotensinogen gene (AGT) and essential hypertension using ambulatory blood pressure (BP). METHODS: We recruited 802 subjects in a rural Japanese community who were aged >40 years and who gave written informed consent for monitoring of their ambulatory BP and genetic analysis (the Ohasama Study). As a polymorphism of AGT, T+31C, which is in complete linkage disequilibrium with M235T, was determined using the TaqMan polymerase chain reaction method. RESULTS: The genotype distribution of AGT/T-+31C in the Ohasama Study was similar to that in another large Japanese population. Although there was no significant difference in 24-h and daytime ambulatory BP values, the nighttime BP was significantly lower in the subjects with TT, resulting in greater decline of nocturnal systolic (P = .090) and diastolic (P = .025) BP in subjects with TT. CONCLUSIONS: AGT/T+31C is associated with the circadian BP variation but not with BP level in the Japanese general population. |
| 20503258 | Candidate gene association study conditioning on individual ancestry in patients with type 2 diabetes and metabolic syndrome from Mexico City.BACKGROUND: Type 2 diabetes (T2D) is influenced by diverse environmental and genetic risk factors. Metabolic syndrome (MS) increases the risk of cardiovascular disease and diabetes. We analysed 14 cases of polymorphisms located in 10 candidate loci, in a sample of patients with T2D and controls from Mexico City. METHODS: We analysed the association of 14 polymorphisms located within 10 genes (TCF7L2, ENPP1, ADRB3, KCNJ11, LEPR, PPARgamma, FTO, CDKAL1, SIRT1 and HHEX) with T2D and MS. The analysis included 519 subjects with T2D defined according to the ADA criteria, 389 with MS defined according to the AHA/NHLBI criteria and 547 controls. Association was tested with the program ADMIXMAP including individual ancestry, age, sex, education and in some cases body mass index (BMI), in a logistic regression model. RESULTS: The two markers located within the TCF7L2 gene showed strong associations with T2D (rs7903146, T allele, odd ratio (OR) = 1.76, p = 0.001 and rs12255372, T allele, OR = 1.78, p = 0.002), but did not show significant association with MS. The non-synonymous rs4994 polymorphism of the ADRB3 gene was associated with T2D (Trp allele, OR = 0.62, p = 0.001) and MS (Trp allele, OR = 0.74, p = 0.018). Nominally significant associations were also observed between T2D and the SIRT1 rs3758391 SNP and MS and the HHEX rs5015480 polymorphism. CONCLUSIONS: Variants located within the gene TCF7L2 are strongly associated with T2D but not with MS, providing support to previous evidence indicating that polymorphisms at the TCF7L2 gene increase T2D risk. In contrast, the non-synonymous ADRB3 rs4994 polymorphism is associated with T2D and MS. |
| 22459021 | Relationship of metabolic syndrome and its components with 844 G/A and HindIII C/G PAI-1 gene polymorphisms in Mexican children.ABSTRACT: BACKGROUND: Several association studies have shown that 844 G/A and HindIII C/G PAI-1 polymorphisms are related with increase of PAI-1 levels, obesity, insulin resistance, glucose intolerance, hypertension and dyslipidemia, which are components of metabolic syndrome. The aim of this study was to analyze the allele and genotype frequencies of these polymorphisms in PAI-1 gene and its association with metabolic syndrome and its components in a sample of Mexican mestizo children. METHODS: This study included 100 children with an age range between 6-11 years divided in two groups: a) 48 children diagnosed with metabolic syndrome and b) 52 children metabolically healthy without any clinical and biochemical alteration. Metabolic syndrome was defined as the presence of three or more of the following criteria: fasting glucose levels [greater than or equal to] 100 mg/dL, triglycerides [greater than or equal to] 150 mg/dL, HDL-cholesterol < 40 mg/dL, obesity BMI [greater than or equal to] 95th percentile, systolic blood pressure (SBP) and diastolic blood pressure (DBP) [greater than or equal to] 95th percentile and insulin resistance HOMA-IR [greater than or equal to] 2.4. The 844 G/A and HindIII C/G PAI-1 polymorphisms were analyzed by PCR-RFLP. RESULTS: For the 844 G/A polymorphism, the G/A genotype (OR = 2.79; 95% CI, 1.11-7.08; p = 0.015) and the A allele (OR = 2.2; 95% CI, 1.10-4.43; p = 0.015) were associated with metabolic syndrome. The 844 G/A and A/A genotypes were associated with increase in plasma triglycerides levels (OR = 2.6; 95% CI, 1.16 to 6.04; p = 0.02), decrease in plasma HDL-cholesterol levels (OR = 2.4; 95% CI, 1.06 to 5.42; p = 0.03) and obesity (OR = 2.6; 95% CI, 1.17-5.92; p = 0.01). The C/G and G/G genotypes of the HindIII C/G polymorphism contributed to a significant increase in plasma total cholesterol levels (179 vs. 165 mg/dL; p = 0.02) in comparison with C/C genotype. CONCLUSIONS: The 844 G/A PAI-1 polymorphism is related with the risk of developing metabolic syndrome, obesity and atherogenic dyslipidemia, and the HindIII C/G PAI-1 polymorphism was associated with the increase of total cholesterol levels in Mexican children. |
| 24249967 | SERPINE1, PAI-1 protein coding gene, methylation levels and epigenetic relationships with adiposity changes in obese subjects with metabolic syndrome features under dietary restriction.Plasminogen activator inhibitor 1 (PAI-1) has been associated with metabolic disorders, through different mechanisms, which could involve changes in DNA methylation. This work aimed to assess the potential relationships of the cytosine methylation levels within SERPINE1 gene transcriptional regulatory region, which codes for PAI-1, in peripheral white blood cells with anthropometrical, metabolic and inflammatory features. Forty-six obese subjects with metabolic syndrome features followed Control or Metabolic Syndrome Reduction in Navarra (RESMENA) energy-restricted (-30%E) diets for 8 weeks. SERPINE1 transcriptional regulatory region methylation at baseline was analyzed by a microarray technical. Both dietary strategies reduced anthropometric and biochemical parameters. The Control group significantly reduced plasma PAI-1 concentrations but not the RESMENA group. Participants from both nutritional interventions with higher SERPINE1 methylation levels at baseline showed significantly major reductions in body weight, total fat mass, android fat mass, total cholesterol and triglycerides, as compared with those with lower initial SERPINE1 methylation levels. In conclusion, the DNA methylation levels of SERPINE1 transcriptional regulatory region were associated with some metabolic and anthropometric changes in obese subjects with metabolic syndrome under energy restriction, suggesting a complex epigenetic network in the regulation of this recognized pro-inflammatory marker. (www.clinicaltrials.gov; NCT01087086). |
| 11015607 | Bradykinin B2 null mice are prone to renal dysplasia: gene-environment interactions in kidney development.Congenital abnormalities of the kidney and urinary tract are a common cause of end-stage renal disease in children. Host and environment factors are implicated in the pathogenesis of aberrant renal development. However, direct evidence linking gene-environment interactions with congenital renal disease is lacking. We report an animal model of renal dysgenesis that is dependent on a defined genetic defect and specific embryonic stressor. Specifically, mice that are deficient in the bradykinin type 2 receptor gene (B(2)) and salt loaded during embryogenesis acquire an aberrant kidney phenotype and die shortly after birth. In contrast, B(2) mutant mice maintained on normal sodium intake or salt-loaded wild-type mice do not develop kidney abnormalities. The kidney abnormality is evident histologically on embryonic day 16, shortly after the onset of metanephric B(2) gene expression, and consists of distorted renal architecture, foci of tubular dysgenesis, and cyst formation. The dysplastic tubules are of distal nephron origin [Dolichos biflorus agglutinin (DBA)- and aquaporin-2 (AQP2) positive, and angiotensinogen negative]. Neonatal antihypertensive therapy fails to ameliorate the renal abnormalities, arguing against the possibility that the nephropathy is a consequence of early hypertension. Moreover, the nephropathy is intrinsic to the embryo, because B(2) homozygous offspring from heterozygous parents exhibit the same renal phenotype as offspring from homozygous null parents. Further characterization of the renal phenotype revealed an important genetic background effect since the penetrance of the congenital nephropathy is increased substantially upon backcrossing of 129/BL6 B(2) mutants to a uniform C57BL/6J. We conclude that the type 2 bradykinin receptor is required for the maintenance of metanephric structure and epithelial integrity in the presence of fetal stress. This study provides a "proof-of-principle" that defined gene-environment interactions are a cause of congenital renal disease. |
| 15959859 | Single nucleotide variants in the beta2-adrenergic and beta3-adrenergic receptor genes explained 18.3% of adolescent obesity variation.Associations of obesity with its candidate genes, beta-adrenergic receptor genes (ADRBs), peroxisome proliferator-activated receptor-gamma (PPARgamma), and uncoupling proteins (UCPs) were studied in Korean adolescents. We analyzed the obesity-related phenotypes body mass index (BMI), percentage of body fat, plasma leptin and insulin levels, fasting glucose concentration, and plasma lipid profile in 329 teenagers to investigate the effects of seven single nucleotide variants 252G/A, 523C/A and 1053G/C in ADRB2; Trp64Arg in ADRB3; 161C/T in PPARgamma; Ala55Val in UCP2; and 210C/T in UCP3. The 1053G/C polymorphism (P < 0.05) in the ADRB2 gene and the Trp64Arg polymorphism (P < 0.01) in the ADRB3 gene were associated with BMI after adjustment for dietary energy intake. Trp64Arg polymorphism also influenced percentage of body fat (P < 0.01) and plasma leptin level (P < 0.05). Furthermore, significant interaction effects between the 1053G/C and Trp64Arg polymorphisms were observed on BMI (P < 0.01). The polymorphisms of the ADRB2 and ADRB3 genes explained 4.3% and 10.1% of the variation on BMI, and the two loci effect, including their epistasis, explained 18.3%. We concluded that 1053G/C and Trp64Arg polymorphisms of the ADRB genes additively and interactively contributed to the variation of complex adolescent obesity. |
| 17307998 | Angiotensin II mediates postischemic leukocyte-endothelial interactions: role of calcitonin gene-related peptide.Vascular inflammation and enhanced production of angiotensin II (ANG II) are involved in the pathogenesis of hypertension and diabetes, disease states that predispose the afflicted individuals to ischemic disorders. In light of these observations, we postulated that ANG II may play a role in promoting leukocyte rolling (LR) and adhesion (LA) in postcapillary venules after exposure of the small intestine to ischemia-reperfusion (I/R). Using an intravital microscopic approach in C57BL/6J mice, we showed that ANG II type I (AT(1)) or type II (AT(2)) receptor antagonism (with valsartan or PD-123319, respectively), inhibition of angiotensin-converting enzyme (ACE) with captopril, or calcitonin gene-related peptide (CGRP) receptor blockade (CGRP8-37) prevented postischemic LR but did not influence I/R-induced LA. However, both postischemic LR and LA were largely abolished by concomitant AT(1) and AT(2) receptor blockade or chymase inhibition (with Y-40079). Additionally, exogenously administered ANG II increased LR and LA, effects that were attenuated by pretreatment with a CGRP receptor antagonist or an NADPH oxidase inhibitor (apocynin). Our work suggests that ANG II, formed by the enzymatic activity of ACE and chymase, plays an important role in inducing postischemic LR and LA, effects that involve the engagement of both AT(1) and AT(2) receptors and may be mediated by CGRP and NADPH oxidase. |
| 17894153 | Ala55Val polymorphism on UCP2 gene predicts greater weight loss in morbidly obese patients undergoing gastric banding.BACKGROUND: Variability in weight loss has been observed from morbidly obese patients receiving bariatric operations. Genetic effects may play a crucial role in this variability. METHODS: 304 morbidly obese patients (BMI > or =39) were recruited, 77 receiving laparoscopic adjustable gastric banding (LAGB) and 227 laparoscopic mini-gastric bypass (LMGB), and 304 matched non-obese controls (BMI < or =24). Initially, all subjects were genotyped for 4 SNPs (single nucleotide polymorphisms) on UCP2 gene in a case-control study. The SNPs significantly associated with morbid obesity (P < 0.05) were considered as candidate markers affecting weight change. Subsequently, effects on predicting weight loss of those candidate markers were explored in LAGB and LMGB, respectively. The peri-operative parameters were also compared between LAGB and LMGB. RESULTS: The rs660339 (Ala55Val), on exon 4, was associated with morbid obesity (P = 0.049). Morbidly obese patients with either TT or CT genotypes on rs660339 experienced greater weight loss compared to patients with CC after LAGB at 12 months (BMI loss 12.2 units vs 8.1 units) and 24 months (BMI loss 13.1 units vs 9.3 units). However, this phenomenon was not observed in patients after LMGB. Although greater weight loss was observed in patients receiving LMGB, this procedure had a higher operative complication rate than LAGB (7.5% vs. 2.8%; P < 0.05). CONCLUSION: Ala55Val may play a crucial role in obesity development and weight loss after LAGB. It may be considered as clinicians incorporate genetic susceptibility testing into weight loss prediction prior to bariatric operations. |
| 20027122 | Common genetic variations of the renin-angiotensin-aldosterone system and response to acute angiotensin I-converting enzyme inhibition in essential hypertension.OBJECTIVE: In order to get insight into possible genetic determinants of antihypertensive drug action, we analysed the relations between polymorphisms of the genes of the renin-angiotensin-aldosterone system and acute effects of ACE inhibition on blood pressure as well as circulating renin and aldosterone levels in hypertensive patients. METHODS: A total of 315 hypertensive patients referred for problems in drug treatment were given a single 50 mg dose of captopril. Plasma renin and aldosterone were measured before and 60 min after the drug administration. Four DNA variants, including angiotensin type I receptor (AGTR1) 1166 A/C, angiotensin-converting enzyme (ACE) I/D, angiotensinogen (AGT) M235T and AGT -217 G/A, were genotyped in the patients and normotensive men (n = 175). A replication study on the relation between AGTR1 1166 A/C and plasma renin and aldosterone levels was carried out in the 244 hypertensive men of the pharmacogenetic GENRES Study. RESULTS: Referred hypertensive patients with the AGTR1 CC genotype had higher aldosterone at baseline (P = 0.02) and after 60 min of captopril administration (P = 0.01) compared with the AA genotype. Replicate analysis in the GENRES patients showed a similar trend. When the two studies were combined (315 and 244 patients, respectively), plasma aldosterone level (P = 0.007) as well as aldosterone/renin ratio (P = 0.04) were significantly higher in the CC genotype (n = 13) than in the AA genotype (n = 370). Transfection studies in cultured HEK293 cells indicated that the 1166C allele was associated with higher mRNA levels than the 1166A allele. CONCLUSION: The AGTR1 1166C allele when present in homozygous form may be associated with a form of essential hypertension characterized by high plasma aldosterone and low plasma renin levels, possibly due to increased AGTR1 mRNA levels and augmented angiotensin II action. |
| 20577119 | Pharmacogenetic association of hypertension candidate genes with fasting glucose in the GenHAT Study.BACKGROUND: Several clinical studies report increased risk of diabetes mellitus with pharmacologic treatment for hypertension (HTN). HTN genes may modify glycemic response to antihypertensive treatment. METHOD: The current study examined the association of 24 single nucleotide polymorphisms (SNPs) in 11 HTN candidate genes with fasting glucose measured at 2, 4, and 6 years after treatment initiation. The study sample included participants free of diabetes at baseline in the Genetics of Hypertension Associated Treatment (GenHAT) study (N = 9309). GenHAT participants were randomized to receive treatment with a diuretic (chlorthalidone), calcium channel blocker (amlodipine), or angiotensin-converting enzyme (ACE) inhibitor (lisinopril). Mixed models for repeated measures were employed to test for gene and pharmacogenetic associations with fasting glucose during follow-up. RESULTS: Fasting glucose at year 2 increased on average 6.8, 4.8 and 3.0 mg/dl from baseline in the chlorthalidone, amlodipine and lisinopril groups, respectively. Carrying the I allele (rs1799752) of the ACE I/D polymorphism was associated with lower fasting glucose levels (P = 0.02). Additionally, an ACE promoter polymorphism (-262, rs4291) was associated with lower fasting glucose for the model AA/AT vs. TT, which remained significant after correction for multiple testing (P = 0.001). Finally, a SNP in the alpha-subunit of the amiloride-sensitive epithelial sodium channel (SCNN1A, rs2228576) modified the association of amlodipine vs. chlorthalidone treatment with fasting glucose (P < 0.001). CONCLUSION: Further examination of these genes and their relationships with cardiometabolic disease could foster development of pharmacogenetic guidelines aimed to prevent increases in fasting glucose during antihypertensive treatment. |
| 23304115 | Plasma plasminogen activator inhibitor-1 is associated with end-stage proliferative diabetic retinopathy in the Northern Chinese Han population.OBJECTIVE: To identify predictors of end-stage proliferative diabetic retinopathy (PDR) in a cohort of individuals with type 2 diabetes mellitus (T2DM) from the Northern Chinese Han population. METHODS: We investigated characteristics of 153 consecutive diabetic patients with end-stage PDR (62 males, 91 females), 123 consecutive PDR patients without end-stage PDR (48 males, 75 females), and 151 normal subjects (63 males, 88 females). Only one eye of each patient or healthy subject was included in this study. Univariate logistic regression models and multivariate logistic regression models were constructed to evaluate the predictors of end-stage PDR. RESULTS: In univariate analysis, systolic blood pressure, diastolic blood pressure, duration of diabetes, family history of T2DM, and plasminogen activator inhibitor-1 (PAI-1) were significently associated with end-stage PDR. After multivariate analysis, family history of T2DM, plasma PAI-1 levels, smoking, and duration of diabetes were four positive predictors associated with end-stage PDR. CONCLUSIONS: Higher plasma levels of PAI-1 were associated with end-stage PDR in the Northern Chinese Han population with T2DM. |
| 11531970 | Both alleles of the M235T polymorphism of the angiotensinogen gene can be a risk factor for myocardial infarction.We have studied the role of three polymorphic genes of the renin-angiotensin system (RAS) as independent risk factors for myocardial infarction (MI) and their correlation with three of the major coronary risk factors: serum cholesterol (CH), hypertension (HT) and smoking (SM). A population of 392 men was genotyped for the M235T polymorphism of the angiotensinogen (AGT) gene, the insertion/deletion of the angiotensin-converting enzyme (ACE) and the all66c of the angiotensin-II type 1 receptor (AT1R), by means of polymerase chain reaction (PCR) and restriction enzyme analysis. It was observed that the T allele frequency increased significantly in the MI with HT, CH, and SM subgroup (0.58 vs 0.31) (p<0.01). In contrast, the M allele frequency was higher in the MI without HT, CH, and SM (0.69 vs 0.42) (p<0.01). A strong association between the MM genotype and MI (p<0.001, odds ratio=4.29, confidence interval=1.95-9.42) was found when age-matched MM control subjects were compared to MI individuals with none of the other known major coronary risk factors. Futhermore, subjects with the MM genotype showed a significantly higher plasma renin activity (PRA) profile than those with the TT genotype (p<0.001). It can be concluded that the M allele is an independent risk factor for MI and the T allele modified the risk when other major risk factors are present. |
| 12187393 | Body weight changes and the A-6G polymorphism of the angiotensinogen gene.BACKGROUND: The objective of the study was to analyze the relationship of polymorphisms of the angiotensinogen gene with changes in body weight during 3 y of antihypertensive treatment, in a group of young adults with essential hypertension. METHODS: Essential hypertensives, less than 50 y old, never previously treated with antihypertensive drugs and in the absence of diabetes mellitus were included. After the initial evaluation, patients were treated using only non-pharmacological measures (n=29), beta-blockers (n=40) or angiotensin-converting enzyme inhibitors (n=66). Resting blood pressure, biochemical profile and body weight at the beginning and yearly were measured. The polymorphism A-6G of the angiotensinogen gene located in the promoter region was analyzed. RESULTS: One-hundred and thirty-five patients were included. Genotypes of the A-6G polymorphism of the AGT gene were in Hardy-Weinberg equilibrium (AA 34, AG 63, GG 38). No significant differences were observed among genotypes in terms of age, body mass index, body weight, systolic or diastolic blood pressure. No significant differences in the genotype distribution or in the allele frequencies were observed, although the A allele was most frequent among the obese subjects. During the 3 y of antihypertensive treatment, there was a trend to increase weight despite the dietary recommendations. The slopes of body weight over time, adjusted by age and baseline BMI, differed significantly among the homozygote genotypes (P=0.006). The highest were for those with the AA genotype and the lowest for the GG genotype (1.180+/-0.25 and -0.128+/-0.24 kg/y; P=0.0001). The influence of the genotype in the changes on body weight remained significant after considering its interaction with the kind of antihypertensive treatment, although among subjects carrying the AA genotype those treated with ACEi showed the least body weight change. Furthermore, A-6G genotypes had the largest influence on weight changes, accounting for 19% of the variance, when age, sex and initial body mass index were included in the model. CONCLUSIONS: In a group of young adult hypertensive subjects, there was a trend to increase weight despite dietary recommendations. Subjects with the AA genotype were those with the largest weight gain, but this effect was modified by the antihypertensive treatment. |
| 14592559 | Relationships between fibrinogen, plasminogen activator inhibitor-1, and their gene polymorphisms in current smokers with essential hypertension.BACKGROUND: To elucidate the role of some haemostatic gene polymorphisms and environmental factors, we studied fibrinogen (Fb), plasminogen activator inhibitor-1 (PAI-1), and tissue plasminogen activator (t-PA) levels with respect to Fb G455A and PAI-1 4G/5G gene polymorphisms in smokers and nonsmokers with essential hypertension. MATERIAL AND METHODS: The study was done in 90 patients (including 30 smokers) with essential hypertension (HT) and 40 controls (including 8 smokers). Fb and PAI-1 genotypes were PCR identified. The groups did not differ significantly as to genotype frequencies. RESULTS: When allele A455 carriers were compared, HT patients had significantly higher Fb (p=0.015) and t-PA levels (p=0.013). Comparison of 4G allele carriers (4G/4G homozygotes) revealed significantly higher Fb (p=0.045), PAI-1 (p=0.009), and t-PA levels (p=0.007) in HT patients than controls. Interactions of Fb and PAI-1 gene polymorphisms with smoking were disclosed in HT patients only. Allele A455-carrying HT smokers compared with nonsmokers had significantly higher t-PA (12.1 +/- 5.8 vs. 7.4 +/- 3.1 ng/ml; p=0.002) and tendency to higher Fb (3.36 +/- 0.74 vs. 2.95 +/- 0.70 g/l; p=0.075) levels. Higher Fb levels were disclosed in 4G/4G smokers than nonsmokers (3.31 +/- 0.81 vs. 2.84 +/- 0.85 g/l; p=0.064). Finally, in smokers, significantly higher levels of PAI-1 were found in 4G/4G (42.1 +/- 29.4 ng/ml) as compared with 4G/5G (18.6 +/- 13.7 ng/ml; p=0.025) and 5G/5G (14.4 +/- 10.8 ng/ml; p=0.044) genotypes. CONCLUSIONS: Smoking potentiates the prothrombotic effect of allele A455 and PAI-1 4G/4G genotype in untreated essential hypertension, reflected by increased levels of haemostatic risk factors and accelerated progression of cardiovascular diseases. |
| 14610081 | Cellular carbonyl stress enhances the expression of plasminogen activator inhibitor-1 in rat white adipocytes via reactive oxygen species-dependent pathway.Carbonyl stress is one of the important mechanisms of tissue damage in vascular complications of diabetes. In the present study, we observed that the plasminogen activator inhibitor-1 (PAI-1) levels in serum and its gene expression in adipose tissue were up-regulated in aged OLETF rats, model animals of obese type 2 diabetes. To study the mechanism of PAI-1 up-regulation, we examined the effect of advanced glycation end products (AGEs) and the product of lipid peroxidation (4-hydroxy-2-nonenal (HNE)), both of which are endogenously generated under carbonyl stress. Stimulation of primary white adipocytes by either AGE or HNE resulted in the elevation of PAI-1 in culture medium and at mRNA levels. The up-regulation of PAI-1 was also observed by incubating the cells in high glucose medium (30 mm, 48 h). The stimulatory effects by AGE or high glucose were inhibited by antioxidant, pyrrolidine dithiocarbamate, and reactive oxygen scavenger, probucol, suggesting a pivotal role of oxidative stress in white adipocytes. We also found that the effect by HNE was inhibited by antioxidant, N-acetylcysteine and that a specific inhibitor of glutathione biosynthesis, l-buthionine-S,R-sulfoximine, augmented the effect of subthreshold effect of HNE. Bioimaging of reactive oxygen species (ROS) by a fluorescent indicator, 6-carboxy-2',7'-dichlorodihydrofluorescein diacetate, revealed ROS production in white adipocytes treated with AGE or HNE. These results suggest that cellular carbonyl stress induced by AGEs or HNE may stimulate PAI-1 synthesis in and release from adipose tissues through ROS formation. |
| 15097234 | Hypertension-related gene polymorphisms in pre-eclampsia, eclampsia and gestational hypertension in Black South African women.OBJECTIVE: To examine whether polymorphisms in the renin-angiotensin system (RAS) are associated with pregnancy-related hypertensive disorders in a black South African population. DESIGN: The angiotensin-converting enzyme (ACE) insertion/deletion, angiotensinogen M235T and angiotensin II receptor type 1 1166A<--C polymorphisms were assessed in study groups comprising 204 women with pre-eclampsia, 120 with eclampsia, 67 with early onset pre-eclampsia and 78 with gestational hypertension. METHODS: Using chi analysis, results were compared with those obtained from 338 ethnically matched normotensive pregnant women following normal full term pregnancies. No significant differences in the distribution of any of these polymorphisms were found between patients with pre-eclampsia or eclampsia and the normal control subjects. Patients with gestational hypertension were less frequently homozygous for the ACE insertion polymorphism compared with controls (5 versus 13%, respectively; P = 0.049; odds ratio 0.36 [95% confidence interval (CI) 0.09-1.04]). CONCLUSION: The commonly occurring RAS polymorphisms are not predictive of pre-eclampsia or eclampsia in the Black South African population. |
| 15302839 | Endogenous angiotensin II induces atherosclerotic plaque vulnerability and elicits a Th1 response in ApoE-/- mice.Rupture of vulnerable plaques is the main cause of acute cardiovascular events. However, mechanisms responsible for transforming a stable into a vulnerable plaque remain elusive. Angiotensin II, a key regulator of blood pressure homeostasis, has a potential role in atherosclerosis. To study the contribution of angiotensin II in plaque vulnerability, we generated hypertensive hypercholesterolemic ApoE-/- mice with either normal or endogenously increased angiotensin II production (renovascular hypertension models). Hypertensive high angiotensin II ApoE-/- mice developed unstable plaques, whereas in hypertensive normal angiotensin II ApoE-/- mice plaques showed a stable phenotype. Vulnerable plaques from high angiotensin II ApoE-/- mice had thinner fibrous cap (P<0.01), larger lipid core (P<0.01), and increased macrophage content (P<0.01) than even more hypertensive but normal angiotensin II ApoE-/- mice. Moreover, in mice with high angiotensin II, a skewed T helper type 1-like phenotype was observed. Splenocytes from high angiotensin II ApoE-/- mice produced significantly higher amounts of interferon (IFN)-gamma than those from ApoE-/- mice with normal angiotensin II; secretion of IL4 and IL10 was not different. In addition, we provide evidence for a direct stimulating effect of angiotensin II on lymphocyte IFN-gamma production. These findings suggest a new mechanism in plaque vulnerability demonstrating that angiotensin II, within the context of hypertension and hypercholesterolemia, independently from its hemodynamic effect behaves as a local modulator promoting the induction of vulnerable plaques probably via a T helper switch. |
| 17496209 | Angiotensin-(1 7) stimulates the phosphorylation of JAK2, IRS-1 and Akt in rat heart in vivo: role of the AT1 and Mas receptors.Angiotensin (ANG) II exerts a negative modulation on insulin signal transduction that might be involved in the pathogenesis of hypertension and insulin resistance. ANG-(1-7), an endogenous heptapeptide hormone formed by cleavage of ANG I and ANG II, counteracts many actions of ANG II. In the current study, we have explored the role of ANG-(1-7) in the signaling crosstalk that exists between ANG II and insulin. We demonstrated that ANG-(1-7) stimulates the phosphorylation of Janus kinase 2 (JAK2) and insulin receptor substrate (IRS)-1 in rat heart in vivo. This stimulating effect was blocked by administration of the selective ANG type 1 (AT(1)) receptor blocker losartan. In contrast to ANG II, ANG-(1-7) stimulated cardiac Akt phosphorylation, and this stimulation was blunted in presence of the receptor Mas antagonist A-779 or the phosphatidylinositol 3-kinase (PI3K) inhibitor wortmannin. The specific JAK2 inhibitor AG-490 blocked ANG-(1-7)-induced JAK2 and IRS-1 phosphorylation but had no effect on ANG-(1-7)-induced phosphorylation of Akt, indicating that activation of cardiac Akt by ANG-(1-7) appears not to involve the recruitment of JAK2 but proceeds through the receptor Mas and involves PI3K. Acute in vivo insulin-induced cardiac Akt phosphorylation was inhibited by ANG II. Interestingly, coadministration of insulin with an equimolar mixture of ANG II and ANG-(1-7) reverted this inhibitory effect. On the basis of our present results, we postulate that ANG-(1-7) could be a positive physiological contributor to the actions of insulin in heart and that the balance between ANG II and ANG-(1-7) could be relevant for the association among insulin resistance, hypertension, and cardiovascular disease. |
| 17641732 | Pulmonary and cardiorenal cyclooxygenase-1 (COX-1), -2 (COX-2), and microsomal prostaglandin E synthase-1 (mPGES-1) and -2 (mPGES-2) expression in a hypertension model.Hypertensive mice that express the human renin and angiotensinogen genes are used as a model for human hypertension because they develop hypertension secondary to increased renin-angiotensin system activity. Our study investigated the cellular localization and distribution of COX-1, COX-2, mPGES-1, and mPGES-2 in organ tissues from a mouse model of human hypertension. Male (n = 15) and female (n = 15) double transgenic mice (h-Ang 204/1 h-Ren 9) were used in the study. Lung, kidney, and heart tissues were obtained from mice at necropsy and fixed in 10% neutral buffered formalin followed by embedding in paraffin wax. Cut sections were stained immunohistochemically with antibodies to COX-1, COX-2, mPGES-1, and mPGES-2 and analyzed by light microscopy. Renal expression of COX-1 was the highest in the distal convoluted tubules, cortical collecting ducts, and medullary collecting ducts; while proximal convoluted tubules lacked COX-1 expression. Bronchial and bronchiolar epithelial cells, alveolar macrophages, and cardiac vascular endothelial cells also had strong COX-1 expression, with other renal, pulmonary, or cardiac microanatomic locations having mild-to-moderate expression. mPGES-2 expression was strong in the bronchial and bronchiolar epithelial cells, mild to moderate in various renal microanatomic locations, and absent in cardiac tissues. COX-2 expression was strong in the proximal and distal convoluted tubules, alveolar macrophages, and bronchial and bronchiolar epithelial cells. Marked mPGES-1 was present only in bronchial and bronchiolar epithelial cells; while mild-to-moderate expression was present in other pulmonary, renal, or cardiac microanatomic locations. Expression of these molecules was similar between males and females. Our work suggests that in hypertensive mice, there are (a) significant microanatomic variations in the pulmonary, renal, and cardiac distribution and cellular localization of COX-1, COX-2, mPGES-1, and mPGES-2, and (b) no differences in expression between genders. |
| 21911891 | Plasminogen activator inhibitor type 1 serum levels and 4G/5G gene polymorphism in morbidly obese Hispanic patients with non-alcoholic fatty liver disease.BACKGROUND: The plasminogen activator inhibitor type-1 (PAI-1) has been implicated in the regulation of fibrinolysis and extracellular matrix components. The single base pair guanine insertion/deletion polymorphism (4G/5G) within the promoter region of the PAI-1 gene influences PAI-1 synthesis and may modulate hepatic fibrogenesis. AIM: To evaluate the influence of PAI-1 serum levels and 4G/5G polymorphism on the risk of liver fibrosis associated to non-alcoholic fatty liver disease (NAFLD) in morbidly obese patients. MATERIAL AND METHODS: Case-control study of 50 obese patients undergoing bariatric surgery and 71 non-obese subjects matched by age and sex. Anthropometric and biochemical measurements were performed, including PAI-1 serum levels. Genomic DNA was obtained to assess the presence of 4G/5G polymorphism. RESULTS: BMI, insulinemia, triglycerides, HOMA-IR, hypertension and diabetes were significantly higher in obese patients compared to control subjects. PAI-1 serum levels observed in obese patients were significantly lower (10.63 +/- 4.82) compared to controls (14.26 +/- 11.4; p < 0.05). No differences were observed in the PAI-1 4G/5G promoter genotypes frequencies (p = 0.12). No differences were observed in PAI-1 plasma levels among obese patients with liver fibrosis (10.64 +/- 4.35) compared to patients without liver fibrosis (10.61 +/- 5.2; p = 0.985). PAI-1 4G/5G promoter genotypes frequencies were similar in patients with or without liver fibrosis associated to NASH (p = 0.6). CONCLUSIONS: Morbidly obese patients had significantly lower PAI-1 serum levels with similar PAI-1 4G/5G genotypes frequencies compared to non-obese subjects. The frequency of 4G/5G genotypes in Chilean Hispanic healthy subjects was similar to that described in other populations. No association was found between PAI-1 serum levels or 4G/5G genotype with liver fibrosis in obese patients. |
| 22730330 | Cathepsin S cannibalism of cathepsin K as a mechanism to reduce type I collagen degradation.Cathepsins S and K are potent mammalian proteases secreted into the extracellular space and have been implicated in elastin and collagen degradation in diseases such as atherosclerosis and osteoporosis. Studies of individual cathepsins hydrolyzing elastin or collagen have provided insight into their binding and kinetics, but cooperative or synergistic activity between cathepsins K and S is less described. Using fluorogenic substrate assays, Western blotting, cathepsin zymography, and computational analyses, we uncovered cathepsin cannibalism, a novel mechanism by which cathepsins degrade each other as well as the substrate, with cathepsin S predominantly degrading cathepsin K. As a consequence of these proteolytic interactions, a reduction in total hydrolysis of elastin and type I collagen was measured compared with computationally predicted values derived from individual cathepsin assays. Furthermore, type I collagen was preserved from hydrolysis when a 10-fold ratio of cathepsin S cannibalized the highly collagenolytic cathepsin K, preventing its activity. Elastin was not preserved due to strong elastinolytic ability of both enzymes. Together, these results provide new insight into the combined proteolytic activities of cathepsins toward substrates and each other and present kinetic models to consider for more accurate predictions and descriptions of these systems. |
| 22733179 | A 45-bp insertion/deletion polymorphism in uncoupling protein 2 is not associated with obesity in a Chinese population.The association of a 45-bp insertion/deletion (UCP2-45 bp I/D) polymorphism in uncoupling protein 2 with body mass index (BMI) remains controversial. A case-control study was conducted to examine the association in a Chinese population. The 1,526 subjects recruited in downtown Beijing and genotyped included 616 obese subjects with BMI >28 and 910 age- and gender-matched controls with BMI <24. The association of the polymorphisms with obesity was estimated using multivariate logistic regression in three models of inheritance. The odds ratios were 1.08 (95 % CI 0.846-1.368; P = 0.551) in the dominant model, 0.931 (0.751-1.154; P = 0.513) in the additive model, and 1.18 (0.550-2.550; P = 0.666) in the recessive model. The overall comparison of the genotype distributions in obese and control subjects using the chi-square test yielded P = 0.801. Our study demonstrated no association between UCP2-45 bp I/D and BMI variation in the Chinese population. |
| 23559862 | Altered expression of transforming growth factor beta 1 and matrix metalloproteinase-9 results in elevated intraocular pressure in mice.PURPOSE: Extracellular matrix remodeling is thought to have profound effects on tissue architecture and associated function. We have shown previously that overexpression of transforming growth factor beta (TGFbeta), which stimulates matrix accumulation, results in altered morphology, cataract, and ocular hypertension in rodents. We have further shown that TGFbeta-induced cataracts can be mitigated through inhibition of the matrix metalloproteinases (MMP) MMP-2 and MMP-9. We therefore sought to determine whether loss of MMP expression also altered TGFbeta-induced changes in intraocular pressure (IOP). METHODS: To carry out this study, TGFbeta1 transgenic mice were bred onto a MMP-9 null background. IOP measurements were made at 1- to 2-, 2- to 3-, and 3- to 4-month time points using a TonoLab rebound tonometer. Histological and immunofluorescence findings were obtained at the same time points. RESULTS: Our results demonstrate that lens-specific expression of TGFbeta1 in mice results in altered morphology of the anterior segment and an accompanying significant increase in IOP. TGFbeta1 transgenic mice bred onto the MMP-9 null background exhibited a further increase in IOP. Interestingly, the MMP-9-deficient animals (without the TGFbeta transgene), which exhibited normal angle morphology, had increased IOP levels compared to their wild-type littermates. CONCLUSION: These results indicate that TGFbeta and MMP-9 likely act independently in regulating IOP. Additionally, MMP-9 plays an important role in maintaining IOP, and further investigation into the mechanisms of MMP-9 activity in the anterior angle may give clues to how extracellular matrix remodeling participates in ocular hypertension and glaucoma. |
| 7651415 | Repression of the interleukin-6 promoter by estrogen receptor is mediated by NF-kappa B and C/EBP beta.Bone metabolism is regulated by a balance between bone resorption caused by osteoclasts and bone formation caused by osteoblasts. This balance is disturbed in postmenopausal women as a result of lower serum estrogen levels. Estrogen, which is used in hormone replacement therapy to prevent postmenopausal osteoporosis, downregulates expression of the interleukin 6 (IL-6) gene in osteoblasts and bone marrow stromal cells. IL-6 is directly involved in bone resorption by activating immature osteoclasts. We show here that NF-kappa B and C/EBP beta are important regulators of IL-6 gene expression in human osteoblasts. Importantly, the IL-6 promoter is inhibited by estrogen in the absence of a functional estrogen receptor (ER) binding site. This inhibition is mediated by the transcription factors NF-kappa B and C/EBP beta. Evidence is presented for a direct interaction between these two factors and ER. We characterized the protein sequence requirements for this association in vitro and in vivo. The physical and functional interaction depends in part on the DNA binding domain and region D of ER and on the Rel homology domain of NF-kappa B and the bZIP region of C/EBP beta. The cross-coupling between ER, NF-kappa B, and C/EBP beta also results in reduced activity of promoters with ER binding sites. We further show that the mechanism of IL-6 gene repression by estrogen is clearly different from that of activation of promoters with ER binding sites. Therefore, drugs that separate the transactivation and transrepression functions of ER will be very helpful for treatment of osteoporosis without causing undesirable side effects. |
| 11463770 | Angiotensinogen polymorphism M235T, carotid atherosclerosis, and small-vessel disease-related cerebral abnormalities.The angiotensinogen M235T polymorphism has been linked to hypertension and cardiovascular disease. We studied the role of this polymorphism as a risk factor for carotid atherosclerosis and small-vessel disease-related brain abnormalities. A total of 431 randomly selected community-dwelling subjects without clinical evidence for strokes underwent angiotensinogen genotyping and carotid Duplex scanning; 1.5-T brain magnetic resonance imaging (MRI) was done in 396 individuals. At 3-year follow-up, we reexamined 343 and 267 study participants by ultrasound and brain MRI, respectively. Carotid atherosclerosis was graded on a 5-point scale. Small-vessel disease-related brain abnormalities were deep or subcortical white matter lesions or lacunes. Progression of carotid atherosclerosis and MRI findings was rated by direct imaging comparison by 3 independent raters. The M/M, M/T, and T/T genotypes were seen in 20.9%, 52.9%, and 18.1% of subjects, respectively. The M235T polymorphism was neither associated with baseline carotid findings nor with progression of carotid atherosclerosis. There was a trend toward more frequent small-vessel disease-related MRI abnormalities in the T/T than in the other genotypes at the baseline examination. Progression of brain lesions occurred significantly more commonly in T/T than in M/M and M/T carriers (P<0.001). Logistic regression analysis identified the T/T genotype (odds ratio, 3.19; P=0.002) and arterial hypertension (odds ratio, 3.06; P=0.03) as significant independent predictors of lesion progression. These data suggest that the angiotensinogen T/T genotype at position 235 is a genetic marker for brain lesions from and progression of small vessel disease but not for extracranial carotid atherosclerosis. |
| 12038776 | Prothrombotic genotypes are not associated with pre-eclampsia and gestational hypertension: results from a large population-based study and systematic review.DNA samples collected as part of a large population-based case-control study were genotyped to examine the associations of five prothrombotic gene polymorphisms with pre-eclampsia (PE) and gestational hypertension (GH). The polymorphisms studied were: G1691A in Factor V (Factor V Leiden; FVL), prothrombin G20210A, methylenetetrahydrofolate reductase (MTHFR) C677T, plasminogen activator inhibitor-1 4G/5G and the platelet collagen receptor alpha2beta1 C807T. A group of 404 women who developed PE were retrospectively compared with 303 women with GH and 164 control women. The frequency of genotypes did not differ significantly between cases of PE or GH and controls for any of the five polymorphisms studied. We conclude that these prothrombotic genotypes are not associated with the development of PE or GH in our population. The systematic review supports our conclusion, for all but cases of severe disease. which appear to be associated with FVL and, to a lesser extent, MTHFR C677T. There is little value in antenatal screening for prothrombotic polymorphisms to predict the development of pre-eclampsia or gestational hypertension. |
| 15638741 | Targeting the renin-angiotensin system: what's new?The renin-angiotensin system is a key target for drugs combating cardiovascular disease. Angiotensin-converting enzyme (ACE) inhibitors and angiotensin receptor type-1 (AT1 receptor) blockers are well known. However, angiotensin peptides can be generated through a number of pathways besides the classic system. This review outlines some of these pathways, their relation to the classic system and the likely effect of inhibiting them. Renin is still the key enzyme in angiotensin peptide generation and seems to be the only route to angiotensin I formation in vivo. Renin inhibitors may have some advantages in terms of specificity. Also, by blocking angiotensin I generation, the production of downstream bioactive angiotensin I metabolites should also be blocked. Chymase, a mast cell serine protease, cleaves angiotensin I to produce angiotensin II and may be important at sites of inflammation such as atherosclerotic plaque. Angiotensin-converting enzyme 2 (ACE2), a carboxypeptidase structurally related to ACE but resistant to ACE inhibitors, has a protective effect on cardiac function. Neutral endopeptidase 24.11 breaks down both atrial natriuretic peptide and angiotensin II. Inhibiting it potentiates the action of endogenous atrial peptide but only affects circulating angiotensin II when basal levels are above normal. Dual inhibitors of ACE and endopeptidase 24.11 may be of value where there is both sodium retention and increased angiotensin II. Targeting the renin-angiotensin system by gene therapy or antibody treatment may provide a longer-term treatment for hypertension. |
| 15673060 | Effects of perindopril treatment on hemostatic function in patients with essential hypertension in relation to angiotensin converting enzyme (ACE) and plasminogen activator inhibitor-1 (PAI-1) gene polymorphisms.BACKGROUND AND AIM: An imbalance in the hemostatic system is a frequent finding in untreated essential hypertension (HT), and it has been shown that treatment with angiotensin converting entyme (ACE) inhibitors improves hemostatic function. In order to elucidate the role of genetic factors, we studied hemostasis in patients with untreated and treated HT and correlated the results with ACE I/D and plasminogen activator enhibitor-1 (PAI-1) 4G/5G gene polymorphisms. METHODS AND RESULTS: Forty-three males with HT (mean age 31.7 +/- 6.8 years) were compared with 34 age and gender-matched controls. All of the patients were treated with perindopril (4 mg/day) and, after one and six months of therapy, their levels of plasma fibrinogen (Fb), t-PA antigen, PAI-1 antigen, von Willebrand factor (vWF), ACE activity and blood pressure were measured. ACE and PAI-1 genotypes were identified by means of the polymerase chain reaction on DNA isolated from peripheral blood lymphocytes. Untreated patients had significantly higher levels of Fb, PAI-1 (p < 0.01) and t-PA (p < 0.05) regardless of their ACE or PAI-1 genotypes. Perindopril reduced blood pressure regardless of ACE or PAI-1 genotype (p < 0.001). ACE II homozygotes showed the greatest decrease in ACE activity (p < 0.01) and a significant reduction in Fb levels (p < 0.05) after just one month of treatment. Analysis of the group as a whole revealed an increase in t-PA antigen levels after six months of treatment, regardless of ACE or PAI-1 genotype (p < 0.01). CONCLUSIONS: Our results show that essential hypertension predisposes to the procoagulant state characterized by hyperfibrinogenemia and hypofibrinolysis. Perindopril reduced fibrinogen levels in ACE II homozygotes due to its more potent inhibitory action on the renin-angiotensin system in such patients. It improved fibrinolysis by increasing t-PA levels regardless of ACE and PAI-1 genotype. |
| 17145981 | Angiotensinogen M235T and T174M gene polymorphisms in combination doubles the risk of mortality in heart failure.Angiotensinogen M235T and T174M polymorphisms have individually been associated with elevated levels of plasma angiotensinogen, hypertension, and left ventricular hypertrophy. In this study, heart failure patients (n=451) were genotyped for the angiotensinogen M235T and T174M polymorphisms to investigate association with survival (recorded over 4 years of follow-up) and prognostic hormone markers. Patients carrying the 235TT genotype (n=86) were 3 years younger at admission (P=0.011), and, in those with hypertension, diagnosis was made approximately 10 years earlier than other patients. Patients carrying >or=1 174M allele (n=94) were more likely to have a previous history of heart failure (P=0.044) and increased mortality during follow-up (risk ratio: 1.69, 95% CI: 1.03 to 2.79; P=0.038) compared with 174TT homozygotes (n=355), despite having a higher left ventricular ejection fraction (P=0.009). "High-risk" genotype combinations (defined a priori as 235TT and/or >or=1 174M allele; n=144; 32%) were independently predictive of mortality, conferring a 2-fold greater risk of dying during the follow-up period (odds ratio: 2.0; 95% CI: 1.3 to 3.0; P=0.001). This study suggested that angiotensinogen gene variants M235T and T174M may provide prognostic information for long-term survival in heart failure patients. |
| 17230448 | Regulation of PAI-1 gene expression during adipogenesis.Obesity is characterized by elevated levels of circulating plasminogen activator inhibitor-1 (PAI-1), which contribute towards the development of secondary disorders such as type 2 diabetes mellitus and cardiovascular complications. This increase in plasma PAI-1 levels is attributed to an increase in PAI-1 derived from adipose tissue. This study shows that adipose tissue evolved into a major PAI-1 producing organ by gaining capacity during adipocyte differentiation to respond to inducers of PAI-1 transcription. This is mediated by a decrease in E2F1 protein levels, an increase in pRB levels and a decrease in pRB phosphorylation, all leading to a decrease in levels of free E2F, a known transcriptional repressor of PAI-1. Depletion of E2F1-3 was sufficient for inducers such as insulin to potently induce PAI-1 gene expression in pre-adipocytes. Conversely, forced release of pRB-bound endogenous E2F using cell-penetrating peptides can suppress PAI-1 gene expression in adipocytes. This study describes the novel paradigm of cellular differentiation-associated increase in PAI-1 gene expression which is mediated by a decrease in repressor activity, and describes a way of desensitising terminally differentiated cells to PAI-1 inducing agents by restoring endogenous repressor activity. |
| 17914507 | Renin-angiotensin system polymorphisms and renal graft function in renal transplant recipients.OBJECTIVE: To analyze the role of 3 polymorphisms of the renin-angiotensin system (RAS) in renal transplant recipients (RTRs) and correlate them with graft function. METHODS: The present study was performed in the Drug Applied Research Center, Tabriz Medical University, Tabriz, Iran from September 2003 to December 2005 on 108 RTRs (66 males and 42 females, with a mean age of 37.34 +/- 4.97 years) with stable allograft function (creatinine < or =2.2 mg/dl). Following the DNA extraction from the blood leukocytes, the genotypes of the angiotensin converting enzyme (ACE I/D), angiotensinogen (ANG M235T), and angiotensin II type 1 receptor (ATR1 A1166C) were determined by polymerase chain reaction. The magnitude of clearance of creatinine (ClCr) in the setting of each of the above RAS polymorphisms was determined. The ClCr was measured by modification of diet in renal disease formula. Values were expressed as mean +/- SD; p< or =0.05 was considered to indicate statistical significance. RESULTS: There was no association of each genotype of the RAS alone with ClCr, serum urea, cyclosporine through level and the degree of urinary protein excretion rate. However, patients with the DD genotype of angiotensin converting enzyme + CC genotype of angiotensin II type I receptor polymorphisms had lower ClCr (p=0.05) and a higher urinary protein excretion rate (p=0.03). Other combination genotypes of RAS had no effect on allograft function. Interestingly, the percent of hypertensive patients in the C allele (70%) was more than the A allele (30%) of ATR1 polymorphism (p=0.04). CONCLUSION: Although none of the single gene polymorphisms of the RAS affected renal allograft function, combinations of these genotypes were associated with the outcome of allograft function. |
| 18093986 | Visfatin induces human endothelial VEGF and MMP-2/9 production via MAPK and PI3K/Akt signalling pathways: novel insights into visfatin-induced angiogenesis.AIMS: Visfatin is a novel adipokine whose plasma concentrations are altered in obesity and obesity-related disorders; these states are associated with an increased incidence of cardiovascular disease. We therefore investigated the effect of visfatin on vascular endothelial growth factor (VEGF) and matrix metalloproteinases (MMP-2, MMP-9) production and the potential signalling cascades. METHODS AND RESULTS: In human umbilical vein endothelial cells (HUVECs), visfatin significantly and dose-dependently up-regulated gene expression and protein production of VEGF and MMPs and down-regulated expression of tissue inhibitors of MMPs (TIMP-1 and TIMP-2). The gelatinolytic activity of MMPs (analysed by zymography) correlated with mRNA and western blot findings. Interestingly, visfatin significantly up-regulated VEGF receptor 2 expression. Inhibition of VEGFR2 and VEGF [by soluble FMS-like tyrosine kinase-1 (sFlt1)] down-regulated visfatin-induced MMP induction. Visfatin induced dose- and time-dependent proliferation and capillary-like tube formation. Importantly, visfatin was noted to have anti-apoptotic effects. In HUVECs, visfatin dose-dependently activated PI3K/Akt (phosphatidylinositol 3-kinase/Akt) and ERK(1/2) (extracellular signal-regulated kinase) pathways. The functional effects and MMP/VEGF induction were shown to be dependent on the MAPK/PI3K-Akt/VEGF signalling pathways. Inhibition of PI3K/Akt and ERK(1/2) pathways led to significant decrease of visfatin-induced MMP and VEGF production and activation, along with significant reduction in endothelial proliferation and capillary tube formation. CONCLUSION: Our data provide the first evidence of visfatin-induced endothelial VEGF and MMP production and activity. Further, we show for the first time the involvement of the MAPK and PI3K/Akt signalling pathways in mediating these actions, as well as endothelial cell proliferation. Collectively, our findings provide novel insights into visfatin-induced endothelial angiogenesis. |
| 19082699 | The rationale and design of the PERindopril GENEtic association study (PERGENE): a pharmacogenetic analysis of angiotensin-converting enzyme inhibitor therapy in patients with stable coronary artery disease.BACKGROUND: Angiotensin-converting enzyme (ACE) inhibitors reduce clinical symptoms and improve outcome in patients with hypertension, heart failure, and stable coronary artery disease (CAD) and are among the most frequently used drugs in these patient groups. For hypertension, treatment is guided by the level of blood pressure. In the secondary prevention setting, there are no means of guiding therapy. Prior attempts to target ACE-inhibitors to those patients that are most likely to benefit have not been successful, mainly due to the consistency in the treatment effect in clinical subgroups. Still, for prolonged prophylactic treatment with ACE-inhibitors it would be best to target treatment to only those patients most likely to benefit, which would considerably lower the number needed to treat and increase cost-effectiveness. A new approach for such "tailored-therapy" may be to integrate information on the genetic variation between patients. Until now, pharmacogenetic research of the efficacy of ACE-inhibitor therapy in CAD patients is still in a preliminary stage. METHODS: The PERindopril GENEtic association study (PERGENE) is a substudy of the EUROPA trial, a randomized double-blind placebo-controlled multicentre clinical trial which demonstrated a beneficial effect of the ACE-inhibitor perindopril in reducing cardiovascular morbidity and mortality in 12.218 patients with stable coronary artery disease (mean follow-up 4.2 years). Blood tubes were received from patients at the beginning of the EUROPA trial and buffy coats were stored at -40 degrees C at the central core laboratory. Candidate genes were selected in the renin-angiotensin-system and bradykinin pathways. Polymorphisms were selected based on haplotype tagging principles using the HapMap genome project, Seattle and other up-to-date genetic database platforms to comprehensively cover all common genetic variation within the genes. Selection also took into consideration the functionality of SNP's, location within the gene (promoter) and existing relevant literature. The main outcome measure of PERGENE is the effect of genetic factors on the treatment benefit with ACE-inhibitors. The size of this pharmacogenetic substudy allows detection with a statistical power of 98% to detect a difference in hazard ratios (treatment effect) of 20% between genotypes with minor allele frequency of 0.20 (two-sided alpha 0.05). CONCLUSION: The PERGENE study is a large cardiovascular pharmacogenetic study aimed to assess the feasibility of pharmacogenetic profiling of the treatment effect of ACE-inhibitor use with the perspective to individualize treatment in patients with stable coronary artery disease. |
| 19088256 | Tumor necrosis factor-alpha upregulates 11beta-hydroxysteroid dehydrogenase type 1 expression by CCAAT/enhancer binding protein-beta in HepG2 cells.The enzyme 11beta-hydroxysteroid dehydrogenase type 1 (11beta-HSD1) catalyzes the conversion of inactive to active glucocorticoids. 11beta-HSD1 plays a crucial role in the pathogenesis of obesity and controls glucocorticoid actions in inflammation. Several studies have demonstrated that TNF-alpha increases 11beta-HSD1 mRNA and activity in various cell models. Here, we demonstrate that mRNA and activity of 11beta-HSD1 is increased in liver tissue from transgenic mice overexpressing TNF-alpha, indicating that this effect also occurs in vivo. To dissect the molecular mechanism of this increase, we investigated basal and TNF-alpha-induced transcription of the 11beta-HSD1 gene (HSD11B1) in HepG2 cells. We found that TNF-alpha acts via p38 MAPK pathway. Transient transfections with variable lengths of human HSD11B1 promoter revealed highest activity with or without TNF-alpha in the proximal promoter region (-180 to +74). Cotransfection with human CCAAT/enhancer binding protein-alpha (C/EBPalpha) and C/EBPbeta-LAP expression vectors activated the HSD11B1 promoter with the strongest effect within the same region. Gel shift and RNA interference assays revealed the involvement of mainly C/EBPalpha, but also C/EBPbeta, in basal and only of C/EBPbeta in the TNF-alpha-induced HSD11B1 expression. Chromatin immunoprecipitation assay confirmed in vivo the increased abundance of C/EBPbeta on the proximal HSD11B1 promoter upon TNF-alpha treatment. In conclusion, C/EBPalpha and C/EBPbeta control basal transcription, and TNF-alpha upregulates 11beta-HSD1, most likely by p38 MAPK-mediated increased binding of C/EBPbeta to the human HSD11B1 promoter. To our knowledge, this is the first study showing involvement of p38 MAPK in the TNF-alpha-mediated 11beta-HSD1 regulation, and that TNF-alpha stimulates enzyme activity in vivo. |
| 19231582 | Diurnal expression of the rat intestinal sodium-glucose cotransporter 1 (SGLT1) is independent of local luminal factors.BACKGROUND: The intestinal sodium-glucose cotransporter 1 (SGLT1) is responsible for all secondary active transport of dietary glucose, and it presents a potential therapeutic target for obesity and diabetes. SGLT1 expression varies with a profound diurnal rhythm, matching expression to nutrient intake. The mechanisms entraining this rhythm remain unknown. We investigated the role of local nutrient signals in diurnal SGLT1 entrainment. METHODS: Male Sprague-Dawley rats, which were acclimatized to a 12:12 light:dark cycle, underwent laparotomy with formation of isolated proximal jejunal loops (Thiry-Vella loops). Animals were recovered for 10 days before harvesting at 4 6-h intervals (Zeitgeber times ZT3, ZT9, ZT15, and ZT21, where ZT0 is lights on; n = 6-8). SGLT1 expression was assessed in protein, and mRNA extracts of mucosa were harvested from both isolated loops (LOOP) and remnant jejunum (JEJ). RESULTS: Isolated loops were healthy but atrophic with minimal changes to villus architecture. A normal anticipatory rhythm was observed in Sglt1 transcription in both LOOP and JEJ, with the peak signal at ZT9 (2.7-fold, P < .001). Normal diurnal rhythms were also observed in the protein signal, with peak expression in both LOOP and JEJ at ZT9 to 15 (2.1-fold, P < .05). However, an additional more mobile polypeptide band was also observed in all LOOP samples but not in JEJ samples (61 kDa vs 69 kDa). Enzymatic deglycosylation suggested this to be deglycosylated SGLT1. CONCLUSION: The persistence of SGLT1 rhythmicity in isolated loops indicates that diurnal induction is independent of local luminal nutrient delivery, and it suggests a reliance on systemic entrainment pathways. However, local luminal signals may regulate glycosylation and, therefore, the posttranslational handling of SGLT1. |
| 19268562 | Influence of a type 2 diabetes associated prostaglandin E synthase 2 polymorphism on blood prostaglandin E2 levels.In this study we tested whether the type 2 diabetes mellitus associated prostaglandin E synthase 2 arginine to histidine polymorphism at position 298 (R298H) influences prostaglandin E2 levels in humans. Fasting prostaglandin E2 was determined in the blood of subjects carrying different genotypes of the R298H polymorphism. Subjects were matched by sex, age, and body mass index. No differences in prostaglandin E2 levels were found with respect to genotypes when considering the whole group. Male homozygous histidine carriers showed elevated prostaglandin E2 levels compared to heterozygous carriers and homozygous arginine carriers (188.2+/-42.4 vs. 80.4+/-26.5pg/ml, p=0.021; and vs. 92.9+/-15.3pg/ml, p=0.11). These differences were not evident in female subjects. In contrast, 6-keto-prostaglandin F1alpha levels as independent marker of arachidonic acid metabolism showed ambiguous results. Nevertheless, preliminary evidence of the prostaglandin E synthase 2 R298H polymorphism possibly influencing prostaglandin E2 blood levels in a gender-specific manner was obtained. |
| 20149706 | Exercise ameliorates serum MMP-9 and TIMP-2 levels in patients with type 2 diabetes.AIM: This study assessed the impact of regular exercise on inflammatory markers (high-sensitivity C-reactive protein [hsCRP], fibrinogen), and matrix metalloproteinases (MMPs) and their inhibitors (TIMPs), in patients with type 2 diabetes mellitus (T2DM). PATIENTS: Fifty overweight patients with T2DM were randomly assigned to two groups: (A) an exercise group (EXG, n=25), with self-controlled exercise for at least 150 min/week and one additional supervised exercise session/week; and (B) a control group (COG, n=25), with no exercise instructions. All participants were taking oral antidiabetic drugs, and none had diabetic complications. Clinical parameters, exercise capacity (VO(2 peak)), ventilatory threshold (VT), insulin resistance indices (fasting insulin, HOMA-IR, HOMA%S), hsCRP, fibrinogen, MMP-2, MMP-9, TIMP-1 and TIMP-2 were assessed at baseline and after 16 weeks. RESULTS: No significant changes were found in body mass index, waist/hip ratio, insulin-resistance indices, MMP-2 and TIMP-1 throughout the study in either group (P>0.05). Compared with controls, the EXG showed a significant decrease in systolic and mean blood pressure, total and LDL cholesterol, and HbA(1c) (P<0.05). Also, exercise significantly suppressed levels of fibrinogen (P=0.047), hsCRP (P=0.041) and MMP-9 (P=0.028), and the MMP-9-to-TIMP-1 ratio (P=0.038), whereas VO(2 peak) (P=0.011), VT (P=0.008) and plasma TIMP-2 levels (P=0.022) were considerably upregulated in the EXG vs. COG. Standard multiple-regression analyses revealed that MMP-9 changes were independently associated with fibrinogen and HbA(1c) changes, while fibrinogen changes independently predicted TIMP-2 alterations with exercise. CONCLUSION: Mostly self-controlled exercise of moderate intensity ameliorated serum levels of pro- and anti-atherogenic markers in patients with T2DM, with no effects on body weight. These data offer further insight into the cardioprotective mechanisms of exercise in patients with T2DM. |
| 21116608 | TNF-alpha acutely upregulates amylin expression in murine pancreatic beta cells.AIMS/HYPOTHESIS: Amylin, a secretory protein mainly produced by pancreatic beta cells, is elevated in the circulation of patients with diseases related to acute and chronic inflammation, including acute pancreatitis, pancreas graft rejection, obesity and insulin resistance. TNF-alpha is involved in these disorders. We investigated the effect of TNF-alpha on amylin levels and the underlying mechanisms, using murine pancreatic beta cell line MIN6 and pancreatic islets. METHODS: Amylin, proinsulin and prohormone convertase 1/3, 2 (Pc1/3, Pc2 [also known as Pcsk1/3 and Pcsk2, respectively]) mRNA levels, and amylin promoter and nuclear factor kappaB (NF-kappaB) activation were examined by real-time PCR and luciferase reporter assay, respectively. Amylin protein level and mitogen-activated protein kinase phosphorylation were detected by western blot. Activator protein 1 (AP1) activation was examined by electrophoretic mobility shift assay (EMSA). RESULTS: TNF-alpha acutely induced amylin expression at the transcriptional level and increased proamylin and the intermediate form of amylin in MIN6 cells and islets. However, it had no effect on proinsulin, Pc1/3 and Pc2 expression. Studies with (1) MIN6 cells treated with inhibitors of MEK1/2, c-Jun-N-terminal kinase (JNK) or protein kinase Czeta (PKC(zeta)), (2) MIN6 cells expressing a c-Jun-dominant negative construct and (3) islets from Fos knockout mice demonstrated that TNF-alpha induced amylin expression through the PKC(zeta)-extracellular signal-regulated kinase (ERK)/JNK pathways. EMSA showed that (PKC(zeta)), JNK and ERK1/2 were involved in TNF-alpha-induced AP1 activation, suggesting that TNF-alpha induces murine amylin expression through the (PKC(zeta)) - ERK1/2 - AP and PKC(zeta) - JNK - AP1 pathways. Further studies showed that TNF-alpha also induced murine amylin expression through the phosphatidylinositol 3 kinase-NF-kappaB signalling pathway and enhanced human amylin promoter activation through NF-kappaB and AP1. CONCLUSIONS/INTERPRETATION: TNF-alpha acutely induces amylin gene expression in beta cells through multiple signalling pathways, possibly contributing to amylin elevation in acute inflammation-related pancreatic disorders. |
| 21354214 | Feeble awake effects of plasminogen activator inhibitor type-1 in mice.Plasminogen activator inhibitor-type 1 (PAI-1) is involved in the fibrinolytic system and shows its increased levels in diseases, e.g., obesity and sleep apnea syndrome. The aim of the study is to investigate whether PAI-1 affects sleep-wake patterns in mice. When recombinant mouse PAI-1 was administered intraperitoneally, only rapid but short increases in time spent awake were observed after 20 or 100 mug/kg, although its plasma concentration was kept high for an hour. The results suggest that PAI-1 may serve its role rather as a marker than an initiator of disturbed sleep. |
| 21641389 | Angiotensin II-induced MMP-2 activity and MMP-14 and basigin protein expression are mediated via the angiotensin II receptor type 1-mitogen-activated protein kinase 1 pathway in retinal pigment epithelium: implications for age-related macular degeneration.Accumulation of various lipid-rich extracellular matrix (ECM) deposits under the retinal pigment epithelium (RPE) has been observed in eyes with age-related macular degeneration (AMD). RPE-derived matrix metalloproteinase (MMP)-2, MMP-14, and basigin (BSG) are major enzymes involved in the maintenance of ECM turnover. Hypertension (HTN) is a systemic risk factor for AMD. It has previously been reported that angiotensin II (Ang II), one of the most important hormones associated with HTN, increases MMP-2 activity and its key regulator, MMP-14, in RPE, inducing breakdown of the RPE basement membrane, which may lead to progression of sub-RPE deposits. Ang II exerts most of its actions by activating the mitogen-activated protein kinase (MAPK) signaling pathway. Herein is explored the MAPK signaling pathway as a potential key intracellular modulator of Ang II-induced increase in MMP-2 activity and MMP-14 and BSG protein expression. It was observed that Ang II stimulates phosphorylation of extracellular signal-regulated kinase (ERK) and p38 MAPK in RPE cells and ERK/p38 and Jun N-terminal kinase (JNK) in mice. These effects were mediated by Ang II type 1 receptors. Blockade of ERK or p38 MAPK abrogated the increase in MMP-2 activity and MMP-14 and BSG proteins in ARPE-19 cells. A better understanding of the molecular events by which Ang II induces ECM dysregulation is of critical importance to further define its contribution to the progression of sub-RPE deposits in AMD patients with HTN. |
| 22902781 | CCAAT/enhancer-binding protein beta (C/EBPbeta) expression regulates dietary-induced inflammation in macrophages and adipose tissue in mice.Strong evidence exists for a link between chronic low level inflammation and dietary-induced insulin resistance; however, little is known about the transcriptional networks involved. Here we show that high fat diet (HFD) or saturated fatty acid exposure directly activates CCAAT/enhancer-binding protein beta (C/EBPbeta) protein expression in liver, adipocytes, and macrophages. Global C/EBPbeta deletion prevented HFD-induced inflammation and surprisingly increased mitochondrial gene expression in white adipose tissue along with brown adipose tissue markers PRDM16, CIDEa, and UCP1, consistent with a resistance to HFD-induced obesity. In isolated peritoneal macrophages from C/EBPbeta(-/-) mice, the anti-inflammatory gene LXRalpha and its targets SCD1 and DGAT2 were strikingly up-regulated along with IL-10, while NLRP3, a gene important for activating the inflammasome, was suppressed in response to palmitate. Using RAW 264.7 macrophage cells or 3T3-L1 adipocytes, C/EBPbeta knockdown prevented palmitate-induced inflammation and p65-NFkappaB DNA binding activity, while C/EBPbeta overexpression induced NFkappaB binding, JNK activation, and pro-inflammatory cytokine gene expression directly. Finally, chimeric bone marrow mice transplanted with bone marrow lacking C/EBPbeta(-/-) demonstrated reduced systemic and adipose tissue inflammatory markers, macrophage content, and maintained insulin sensitivity on HFD. Taken together, these results demonstrate that HFD or palmitate exposure triggers C/EBPbeta expression that controls expression of distinct aspects of alternative macrophage activation. Reducing C/EBPbeta in macrophages confers protection from HFD-induced systemic inflammation and insulin resistance, suggesting it may be an attractive therapeutic target for ameliorating obesity-induced inflammatory responses. |
| 9814470 | Human adipose tissue expresses angiotensinogen and enzymes required for its conversion to angiotensin II.Angiotensin II regulates blood pressure and may affect adipogenesis and adipocyte metabolism. Angiotensin II is produced by cleavage of angiotensinogen by renin and angiotensin-converting enzyme in the circulation. In addition, angiotensin II may be produced in various tissues by enzymes of the renin-angiotensin system (RAS) or the nonrenin-angiotensin system (NRAS). We have analyzed the expression of angiotensinogen and enzymes required for its conversion to angiotensin II in human adipose tissue. Northern blot demonstrated angiotensinogen expression in adipose tissue from nine obese subjects. Western blot revealed a distinct band of expected size of the angiotensinogen protein (61 kDa) in isolated adipocytes. RT-PCR, followed by Southern blot, demonstrated renin expression in human adipose tissue. Angiotensin-converting enzyme messenger RNA was detected by RT-PCR, and the identity of the PCR products was verified by restriction enzyme cleavage. Transcripts for cathepsin D and cathepsin G, components of the NRAS, were detected by RT-PCR, verified by restriction enzyme cleavage. We conclude that human adipose tissue expresses angiotensinogen and enzymes of RAS and NRAS. This opens the possibility that angiotensinogen-derived peptides, produced in adipose tissue itself, may affect adipogenesis and play a role in the pathogenesis of obesity. |
| 12093889 | Opposite effects of cyclooxygenase-1 and -2 activity on the pressor response to angiotensin II.Therapeutic use of cyclooxygenase-inhibiting (COX-inhibiting) nonsteroidal antiinflammatory drugs (NSAIDs) is often complicated by renal side effects including hypertension and edema. The present studies were undertaken to elucidate the roles of COX1 and COX2 in regulating blood pressure and renal function. COX2 inhibitors or gene knockout dramatically augment the pressor effect of angiotensin II (Ang II). Unexpectedly, after a brief increase, the pressor effect of Ang II was abolished by COX1 deficiency (either inhibitor or knockout). Ang II infusion also reduced medullary blood flow in COX2-deficient but not in control or COX1-deficient animals, suggesting synthesis of COX2-dependent vasodilators in the renal medulla. Consistent with this, Ang II failed to stimulate renal medullary prostaglandin E(2) and prostaglandin I(2) production in COX2-deficient animals. Ang II infusion normally promotes natriuresis and diuresis, but COX2 deficiency blocked this effect. Thus, COX1 and COX2 exert opposite effects on systemic blood pressure and renal function. COX2 inhibitors reduce renal medullary blood flow, decrease urine flow, and enhance the pressor effect of Ang II. In contrast, the pressor effect of Ang II is blunted by COX1 inhibition. These results suggest that, rather than having similar cardiovascular effects, the activities of COX1 and COX2 are functionally antagonistic. |
| 15795519 | Reduced cyclooxygenase involvement in vascular endothelial function in rat renal transplantation.BACKGROUND: Cardiovascular disease is a major cause of death following renal transplantation. Mechanisms leading to vascular dysfunction outside the transplanted organ involve common risk factors such as hypertension, hypercholesterolemia, proteinuria, but immune-mediated factors may also be involved. We hypothesized that transplantation-associated risk factors are involved in the development of vascular dysfunction following renal transplantation. METHODS: Vascular function was studied in Fisher to Lewis allografts. Lewis to Lewis syngrafted rats served as controls. All rats received cyclosporin A for 10 days. Allografts were treated with ACE inhibition or AT1 receptor blockade or left untreated. After 34 weeks, aorta rings were studied for contractile and dilator responses in the presence or absence of L-NMMA and/or indomethacin. Tissue sections were immunostained for COX-1 and COX-2. RESULTS: In contrast to syngrafts and treated allografts, untreated allografts developed proteinuria and hypercholesterolemia. In aortic rings, NOS inhibition similarly increased contractile responses and decreased dilator responses in syngrafts and allografts, indicating comparable NO pathways. In contrast, indomethacin affected contractile and dilator responses in syngrafts, but not in treated and untreated allografts, indicating absence of COX-derived prostanoids in control over vascular tone in allografts. This was in line with immunohistologic analysis demonstrating reduced aortic COX-2 expression in allografts. COX-1 expression was unaltered. Interestingly, RAS blockade quantitatively increased endothelium-dependent dilation without qualitatively altering COX function and expression. CONCLUSION: Involvement of COX-derived prostaglandins in vascular endothelial function outside the transplanted organ is strongly diminished after allogeneic renal transplantation. RAS blockade improves common cardiovascular risk factors and endothelium-dependent dilation, but fails to restore prostaglandin function. |
| 15860667 | Transcriptional induction of cyclooxygenase-2 in osteoclast precursors is involved in RANKL-induced osteoclastogenesis.Regulation of osteoclast differentiation is key to understanding the pathogenesis and to developing treatments for bone diseases such as osteoporosis. To gain insight into the mechanism of the receptor activator of nuclear factor (NF)-kappaB ligand (RANKL)-specific induction of the osteoclast differentiation program, we took a suppression-subtractive hybridization screening approach to identify genes specifically induced via the RANKL-Rac1 signaling pathway. Among identified targets, we show that RANKL selectively induces cyclooxygenase (COX) 2 expression via Rac1 that results in turn in production of prostaglandin E2 (PGE2) in RAW 264.7 cells. By using transient transfection assays, we found that the -233/-206 region of the COX-2 promoter gene was critical for RANKL-induced promoter activity. This RANKL-responsive region contained an NF-kappaB site that, when mutated, completely abolished the induction of NF-kappaB DNA-binding activity by RANKL. Blockade of COX-2 by celecoxib inhibits differentiation of bone marrow-derived monocyte/macrophage precursor cells (BMMs) into tartrate-resistant acid phosphatase-positive (TRAP+) osteoclastic cells. This inhibition can be rescued by the addition of exogenous PGE2, suggesting that COX-2-dependent PGE2 induction by RANKL in osteoclast precursors is required for osteoclast differentiation. |
| 17942570 | Renal interstitial adenosine is increased in angiotensin II-induced hypertensive rats.Since marked renal vasoconstriction is observed in angiotensin II (ANG II)-mediated hypertensive rats, we studied the possible interaction between ANG II and adenosine in this model. ANG II was infused into male Wistar rats through osmotic minipumps (435 ng x kg(-1) x min(-1)) for 14 days. In sham and ANG II groups, renal tissue and interstitial adenosine were measured; both increased to a similar twofold extent in the ANG II-treated rats (31.40 +/- 4 vs. 62.0 +/- 8.4 nM, sham vs. ANG II, interstitial adenosine; P< 0.001). The latter decreased by 47% with the specific blockade of 5'-nucleotidase. Glomerular hemodynamics demonstrated marked renal vasoconstriction in the angiotensin-treated group, which was reverted by an adenosine A(1)-receptor antagonist (8-cyclopentyl-1,3-dipropylxanthine, 10 mug.kg(-1) x min(-1)). 5'-Nucleotidase and adenosine deaminase (ADA) activities were measured in the cytosolic and membrane fractions. Only the membrane ADA activity decreased from 1,202 +/- 80 to 900 +/- 50 mU/mg protein in the ANG II-treated rats (P< 0.05), as well as in their protein and mRNA expression. Despite the adenosine elevation, A(1) and A(2b) receptor protein did not change; in contrast, downregulation was observed in A(2a) receptor and upregulation in A(3) receptor. A similar pattern was found in the cortex and in the medulla; mRNA significantly decreased only in the A(3) receptor in both segments. These results suggest that the elevation of renal tissue and interstitial adenosine contributes to the renal vasoconstriction observed in the ANG II-induced hypertension and that it is mediated by a decrease in the activity and expression of ADA, increased production of adenosine, and an induced imbalance in adenosine receptors. |
| 18760347 | Distinct roles of Nox1 and Nox4 in basal and angiotensin II-stimulated superoxide and hydrogen peroxide production.NADPH oxidases are major sources of superoxide (O2\*-) and hydrogen peroxide (H2O2) in vascular cells. Production of these reactive oxygen species (ROS) is essential for cell proliferation and differentiation, while ROS overproduction has been implicated in hypertension and atherosclerosis. It is known that the heme-containing catalytic subunits Nox1 and Nox4 are responsible for oxygen reduction in vascular smooth muscle cells from large arteries. However, the exact mechanism of ROS production by NADPH oxidases is not completely understood. We hypothesized that Nox1 and Nox4 play distinct roles in basal and angiotensin II (AngII)-stimulated production of O2\*- and H2O2. Nox1 and Nox4 expression in rat aortic smooth muscle cells (RASMCs) was selectively reduced by treatment with siNox4 or antisense Nox1 adenovirus. Production of O2\*- and H2O2 in intact RASMCs was analyzed by dihydroethidium and Amplex Red assay. Activity of NADPH oxidases was measured by NADPH-dependent O2\*- and H2O2 production using electron spin resonance (ESR) and 1-hydroxy-3-carboxypyrrolidine (CPH) in the membrane fraction in the absence of cytosolic superoxide dismutase. It was found that production of O2\*- by quiescent RASMC NADPH oxidases was five times less than H2O2 production. Stimulation of cells with AngII led to a 2-fold increase of O2\*- production by NADPH oxidases, with a small 15 to 30% increase in H2O2 formation. Depletion of Nox4 in RASMCs led to diminished basal H2O2 production, but did not affect O2\*- or H2O2 production stimulated by AngII. In contrast, depletion of Nox1 in RASMCs inhibited production of O2\*- and AngII-stimulated H2O2 in the membrane fraction and intact cells. Our data suggest that Nox4 produces mainly H2O2, while Nox1 generates mostly O2\*- that is later converted to H2O2. Therefore, Nox4 is responsible for basal H2O2 production, while O2\*- production in nonstimulated and AngII-stimulated cells depends on Nox1. The difference in the products generated by Nox1 and Nox4 may help to explain the distinct roles of these NADPH oxidases in cell signaling. These findings also provide important insight into the origin of H2O2 in vascular cells, and may partially account for the limited pharmacological effect of antioxidant treatments with O2\*- scavengers that do not affect H2O2. |
| 20205596 | Toll-like receptor 4-deficient mice are resistant to chronic hypoxia-induced pulmonary hypertension.Current data suggest that Toll-like receptor 4 (TLR4), a key molecule in the innate immune response, may also be activated following tissue injury. Activation of this receptor is known to induce the production of several proinflammatory cytokines. Given that pulmonary inflammation has been shown to be a key contributor to chronic hypoxia-induced pulmonary vascular remodeling, the authors hypothesized that TLR4-deficient mice would be less susceptible to pulmonary hypertension (PH) as compared to mice with intact TLR4. TLR4-deficient and TLR4-intact strains of inbred mice were exposed to 4, 8, and 16 weeks of hypoxia (0.10 FiO(2)) or normoxia (0.21 FiO(2)) in a normobaric chamber. After chronic hypoxic exposure, TLR4-intact mice developed significant PH evidenced by increased right ventricular systolic pressure, right ventricular hypertrophy, and pulmonary artery medial thickening. In contrast, TLR4-deficient mice had no significant change in any of these parameters and this was associated with decreased pulmonary vascular inflammatory response as compared to the TLR4-intact mice. These results suggest that TLR4 deficiency may decrease the susceptibility to developing PH by attenuating the pulmonary vascular inflammatory response to chronic hypoxia. |
| 21186597 | [Effect of hypoxic hypercapnia on expression of COX-2 mRNA in pulmonary arterioles].AIM: To study the effect of chronic hypoxic hypercapnia on expression of COX-2 mRNA in pulmonary arterioles. METHODS: SD rats were randomly divided into two groups: control group and hypoxic hypercapnic group. COX-2 mRNA was observed in pulmonary arterioles by the technique of in situ hybridization. RESULTS: mPAP, weight ratio of right ventricle (RV) to left ventricle plus septum (LV + S) and COX-2 mRNA in pulmonary arterioles were much higher in rats of hypoxic hypercapnic group than those of control group. Light microscopy showed that vessel smooth muscle cell hypertrophy and vessel cavity straightness were found in hypoxic hypercapnic group. CONCLUSION: Changes of expressions of COX-2 mRNA may regulate hypoxic hypercapnic pulmonary hypertension. |
| 21640730 | The importance of cyclooxygenase 2-mediated oxidative stress in obesity-induced muscular insulin resistance in high-fat-fed rats.AIM: This study was undertaken to examine the effect of cyclooxygenase (COX) 2 inhibition on the development of muscular insulin resistance in high-fat-induced obese rats. MAIN METHODS: The rats were on a regular chow diet (C) or high-fat enriched diet (HFD) energy-restrictedly (HFr), or ad libitum (HFa) for 12weeks. The rats fed HFD ad libitum were further divided into 3 groups: oral gavage with vehicle (HFa), selective COX-2 inhibitors-celecoxib (HFa+C) or nimesulid (HFa+N), 30mg/kg/day, respectively. KEY FINDINGS: Increased fasting plasma insulin, triglyceride and 8-isoprostane levels in HFa were significantly suppressed in those of HFa+C and HFa+N. The whole body insulin resistance of HFa indicated by the increased fasting plasma insulin levels and the elevated area under curve of insulin obtained from the oral glucose tolerance test were significantly reversed in those combined with celecoxib and nimesulid administration compared with those in HFr. The gene expression of COX-2 was significantly increased in epididymal fat but not in soleus muscle in HFa and the enhanced adipose COX-2 expression in high-fat fed rats was suppressed by those with drug treatment. Both selective COX-2 inhibitors reversed the diminished insulin-stimulated glucose uptake and GLUT4 translocation in skeletal muscles of HFa. Obesity-induced oxidative stress indicated by the elevated plasma 8-isoprostane,the decreased ratio of GSH/GSSG and increased TBARS in soleus muscle were significantly reversed by COX-2 inhibition. SIGNIFICANCE: The results suggest that COX-2 inhibition might suppress the muscular insulin resistance indirectly through decreasing the COX-2-mediated systemic oxidative stress in this diet-induced obese model. |
| 21844877 | Matrix metalloproteinase-9 genetic variations affect MMP-9 levels in obese children.OBJECTIVE: Matrix metalloproteinase-9 (MMP-9) is involved in the atherosclerotic process and functional polymorphisms in the MMP-9 gene affect MMP-9 expression/activity, and are associated with cardiovascular diseases. However, no study has tested the hypothesis that functional MMP-9 polymorphisms could affect MMP-9 levels in obese children. We investigated whether three MMP-9 gene polymorphisms (C-1562T (rs3918242), 90(CA)((14-24)) (rs2234681) and Q279R (rs17576)), or haplotypes, affect MMP-9 levels in obese children. METHODS: We studied 175 healthy control children and 127 obese children. Plasma MMP-9, tissue inhibitor of MMPs (TIMP)-1 and adiponectin concentrations were measured using enzyme-linked immunosorbent assay. RESULTS: We found similar MMP-9 genotypes, allelic and haplotypes distributions in the two study groups (P>0.05). However, we found lower plasma MMP-9 concentrations in obese subjects carrying the CC or the QQ genotypes for the C-1562T and the Q279R polymorphisms, respectively, in obese children compared with children with the other genotypes, or with non-obese children with the same genotypes (all P<0.05). Moreover, we found lower MMP-9 levels and lower MMP-9/TIMP-1 ratios (which reflect net MMP-9 activity) in obese children carrying the H2 haplotype (which combines the C, H and Q alleles for the three polymorphisms, respectively) when compared with obese children carrying the other haplotypes, or with non-obese children carrying the same haplotype (P<0.05). CONCLUSIONS: Our findings show that MMP-9 genotypes and haplotypes affect MMP-9 levels in obese children and adolescents, and suggest that genetic factors may modify relevant pathogenetic mechanisms involved in the development of cardiovascular complications associated with obesity in childhood. |
| 21951301 | Protective effects of mineralocorticoid receptor blockade against neuropathy in experimental diabetic rats.AIMS: Mineralocorticoid receptor (MR) blockade is an effective treatment for hypertension and diabetic nephropathy. There are no data on the effects of MR blockade on diabetic peripheral neuropathy (DPN). The aim of this study was to determine whether MRs are present in the peripheral nerves and to investigate the effectiveness of MR blockade on DPN in streptozotocin (STZ)-induced diabetic rats. METHODS: Expression of MR protein and messenger RNA (mRNA) was examined in the peripheral nerves using Western blot analysis and RT-PCR. We next studied the effects of the selective MR antagonist eplerenone and the angiotensin II receptor blocker candesartan on motor and sensory nerve conduction velocity (NCV), morphometric changes and cyclooxygenase-2 (COX-2) gene and NF-kappaB protein expression in the peripheral nerves of STZ-induced diabetic rats. RESULTS: Expression of MR protein and mRNA in peripheral nerves was equal to that in the kidney. Motor NCV was significantly improved by 8 weeks of treatment with either eplerenone (39.1 +/- 1.2 m/s) or candesartan (46.4 +/- 6.8 m/s) compared with control diabetic rats (33.7 +/- 2.0 m/s) (p < 0.05). Sensory NCV was also improved by treatment with candesartan or eplerenone in diabetic rats. Eplerenone and candesartan caused significant improvement in mean myelin fibre area and mean myelin area compared with control diabetic rats (p < 0.05). COX-2 mRNA and NF-kappaB protein were significantly elevated in the peripheral nerves of diabetic rats compared with control rats, and treatment with eplerenone or candesartan reduced these changes in gene expression (p < 0.05). CONCLUSION: MR blockade may have neuroprotective effects on DPN. |
| 23201900 | Whole-organism screening for gluconeogenesis identifies activators of fasting metabolism.Improving the control of energy homeostasis can lower cardiovascular risk in metabolically compromised individuals. To identify new regulators of whole-body energy control, we conducted a high-throughput screen in transgenic reporter zebrafish for small molecules that modulate the expression of the fasting-inducible gluconeogenic gene pck1. We show that this in vivo strategy identified several drugs that affect gluconeogenesis in humans as well as metabolically uncharacterized compounds. Most notably, we find that the translocator protein ligands PK 11195 and Ro5-4864 are glucose-lowering agents despite a strong inductive effect on pck1 expression. We show that these drugs are activators of a fasting-like energy state and, notably, that they protect high-fat diet-induced obese mice from hepatosteatosis and glucose intolerance, two pathological manifestations of metabolic dysregulation. Thus, using a whole-organism screening strategy, this study has identified new small-molecule activators of fasting metabolism. |

  
  
----- Star papers (those papers include more than 100 genes) -----  

|  |  |
| --- | --- |
| 16369102 | Genetic and environmental factors associated with the development of hypertension in pregnancy.Hypertension in pregnancy (HP), one of the most common causes of perinatal deaths, is a multifactorial disease with genetic and environmental factors involved in its etiology. We have carried out molecular epidemiologic research with the purpose of (1) identifying gene variants associated with HP in Japanese women, and (2) analyzing the genetic and environmental factors involved in the pathophysiology of the disease. Self-administered questionnaires were returned by the subjects between 1 and 6 months after delivery. The candidate genetic variants were identified by use of a PCR-RFLP method. T235 of AGT, C1166 of AT1 and Asp298 of NOS3 were respectively associated with HP, although no significant associations were found between the common genetic variants and HP in ACE, FV, MTHFR, B3AR, TNF-A, PAI-1, GSTP1, mEH, and LPL. In analyses using genetic, environmental and lifestyle factors, 5 factors before pregnancy and 4 factors during pregnancy were significantly associated with HP in univariate analysis. Further multivariate analysis revealed 3 factors before pregnancy, i.e. "prepregnancy BMI > or = 24 kg/m(2)", "family history of hypertension" and "TT genotype of AGT", and 2 factors during pregnancy, i.e. "mentally stressful condition" and "salty dishes preferred". Dividing the subjects into 2 subgroups according to whether they possessed "TT genotype of AGT" or not, we identified acquired risk factors before and during pregnancy for HP in each groups. The multivariate analysis identified "mentally stressful condition" as a potent significant risk factor during pregnancy in the former subgroup. However, there were no significant risk factors concerning and "mental stress" in the latter subgroup. Through further exploration of the risk factors associated with HP, we hope to provide useful suggestions about the development of new and effective preventive measures for a range of multifactorial diseases. |
| 15616563 | Foxa2 regulates lipid metabolism and ketogenesis in the liver during fasting and in diabetes.The regulation of fat and glucose metabolism in the liver is controlled primarily by insulin and glucagon. Changes in the circulating concentrations of these hormones signal fed or starvation states and elicit counter-regulatory responses that maintain normoglycaemia. Here we show that in normal mice, plasma insulin inhibits the forkhead transcription factor Foxa2 by nuclear exclusion and that in the fasted (low insulin) state Foxa2 activates transcriptional programmes of lipid metabolism and ketogenesis. In insulin-resistant or hyperinsulinaemic mice, Foxa2 is inactive and permanently located in the cytoplasm of hepatocytes. In these mice, adenoviral expression of Foxa2T156A, a nuclear, constitutively active Foxa2 that cannot be inhibited by insulin, decreases hepatic triglyceride content, increases hepatic insulin sensitivity, reduces glucose production, normalizes plasma glucose and significantly lowers plasma insulin. These changes are associated with increased expression of genes encoding enzymes of fatty acid oxidation, ketogenesis and glycolysis. Chronic hyperinsulinaemia in insulin-resistant syndromes results in the cytoplasmic localization and inactivation of Foxa2, thereby promoting lipid accumulation and insulin resistance in the liver. Pharmacological intervention to inhibit phosphorylation of Foxa2 may be an effective treatment for type 2 diabetes. |
| 19876004 | Obesity-related polymorphisms and their associations with the ability to regulate fat oxidation in obese Europeans: the NUGENOB study.Both obesity and insulin resistance have been related to low fat oxidation rates, which may be genetically determined. The association between variation in fat oxidation rates among obese subjects and genotype was studied for 42 common single-nucleotide polymorphisms (SNPs) in 26 candidate genes for fat oxidation, insulin resistance, and obesity, including FTO. Energy expenditure (EE) and fat oxidation were measured with indirect calorimetry during fasting and 3 h after a high fat load containing 95 energy% of fat (60% saturated fat, energy content 50% of estimated resting EE) in 722 obese subjects (541 women, 181 men) from 8 European centers. After adjustment for center and gender, -178 A>C CD36 (rs2232169) (P = 0.02), -22510 C>G SLC6A14 (women, rs2011162) (P = 0.03), and T690S C>G PCSK1 (rs6235) (P = 0.02) were related to a reduced fat oxidation, whereas 17 C>G SREBF1 (17 C>G) (P = 0.01) was related to increased fat oxidation in the fasting state. The ability to increase fat oxidation after a high fat load was increased in subjects with -174 G>C IL6 (rs1800795) (P = 0.01). Effect sizes range from 1.1 to 3.1% differences in fat oxidation (expressed as % of EE). FTO rs9939609 was not related to fat oxidation. At the same time, the results are not adjusted for multiple testing, thus none of the associations can be considered statistically significant. The results should therefore only be considered as leads to new hypotheses about effects of specific genetic polymorphisms on fasting and postprandial fat oxidation. |
| 18927546 | Lp(a) and risk of recurrent cardiac events in obese postinfarction patients.Studies of recurrent coronary events in obese postinfarction patients show mixed results despite potential importance of obesity-related pathophysiologic processes and associated markers in establishing and predicting risk. The study aim was to determine specific markers of recurrent risk in obese postinfarction patients. Nondiabetic patients of the Thrombogenic Factors and Recurrent Coronary Events (THROMBO) postinfarction study were classified according to BMI as normal weight (<25 kg/m(2)), overweight (25.0-29.9 kg/m(2)), and obese (> or = 30 kg/m(2)). Cox multivariable regression with adjustment for significant clinical covariates was performed in each group monitoring outcome (cardiac death, myocardial infarction (MI), or unstable angina with 26 months follow-up) as a function of 17 thrombogenic, inflammatory, and metabolic blood markers and 17 cardiovascular disease-associated genetic polymorphisms. Results revealed no statistically significant genetic or blood marker variables in normal or overweight patients. For obese postinfarction patients, elevated lipoprotein(a) (Lp(a))was found to be a highly significant risk marker with hazard ratio and 95% confidence interval of 3.94 (2.11-7.35), P = 0.000017 (upper tertile vs. lower two tertiles). Additionally, elevated Lp(a) was found to interact with the -75G>A polymorphism of the apolipoprotein A-I gene and the -250G>A polymorphism of the hepatic lipase gene in establishing risk. We conclude that interactions of elevated Lp(a) with polymorphisms of the apolipoprotein A-I and hepatic lipase genes, primarily reflective of altered lipoprotein metabolism, play an important role in the establishment of recurrent coronary event risk in obese, nondiabetic postinfarction patients. These findings suggest close monitoring and consideration of weight reduction for obese postinfarction patients with elevated Lp(a) levels. |
| 20301725 | Familial Hypertrophic Cardiomyopathy OverviewHypertrophic cardiomyopathy (HCM), caused by mutation in one of the genes currently known to encode different components of the sarcomere, is characterized by left ventricular hypertrophy (LVH) in the absence of predisposing cardiac conditions (e.g., aortic stenosis) or cardiovascular conditions (e.g., long-standing hypertension). The clinical manifestations of HCM range from asymptomatic to progressive heart failure to sudden cardiac death and vary from individual to individual even within the same family. Common symptoms include shortness of breath (particularly with exertion), chest pain, palpitations, orthostasis, presyncope, and syncope. Most often the LVH of HCM becomes apparent during adolescence or young adulthood, although it may also develop late in life, in infancy, or in childhood. The diagnosis of HCM is most often established when two-dimensional echocardiography detects LVH in a nondilated ventricle; it can also be established by pathognomonic histopathologic findings in cardiac tissue. Familial HCM without multisystem involvement is diagnosed by family history and molecular genetic testing of any of the 14 genes currently known to encode different components of the sarcomere. Familial HCM caused by mutation in one of the genes currently known to encode different components of the sarcomere is inherited in an autosomal dominant manner. Formal genetic counseling can be used to identify those family members of a proband who are at increased risk for HCM. Treatment of manifestations: Medical management of diastolic dysfunction; medical or surgical management of ventricular outflow obstruction; restoration and maintenance of sinus rhythm in those with atrial fibrillation; implantable cardioverter-defibrillator (ICD) in survivors of cardiac arrest and those at high risk of cardiac arrest; medical treatment for heart failure and consideration for cardiac transplantation when necessary. Prevention of secondary complications: Consideration of anticoagulation in those with persistent or paroxysmal atrial fibrillation to reduce the risk of thromboembolism; consideration of antibiotic prophylaxis when necessary; during the pregnancy of a woman with HCM, care by an experienced cardiologist and obstetrician trained in high-risk OB. Surveillance: Reassessment of risk for SCD approximately once a year or more frequently based on clinical findings. Agents/circumstances to avoid: Competitive endurance training, burst activities (e.g., sprinting), intense isometric exercise (e.g., heavy weight lifting), dehydration, hypovolemia (i.e., use diuretics with caution), and medications that decrease afterload (e.g., ACE-inhibitors, angiotensin receptor blockers, and other direct vasodilators). Evaluation of relatives at risk: Guidelines have been proposed for periodic screening of asymptomatic at-risk family members. |

Copyright © CoCiter 2011-2013. >>
Designed by QIAO Nan & HUANG Yi >>
Hanlab
