# Supplementary material for: Partial depletion of yolk during zebrafish embryogenesis changes the dynamics of methionine cycle and metabolic genes
Source: BMC Genomics. 2015 Jun 4;16(1):427. doi: 10.1186/s12864-015-1654-6 (PMC4455928; doi:10.1186/s12864-015-1654-6)
Supplement: Additional file 12: — Overlap between outcomes of EdgeR and DESeq2 is statistically significant. For any given RNAseq datatset, the overlap between EdgeR and DESeq2 leads to a significant outcome, as denoted by the P-value of the hypergeometric test (Phyper). In the DESeq2 analysis, we compared YD vs SP triplicates at each time-point separately, using standard settings, using a multifactorial design (design = ~ treatment + batch). Phyper was calculated with the overlap between datasets, and the number of included genes (i.e. the sample pool) was adopted from de EdgeR dataset (numbers in Fig. 1F). EdgeR analysis was performed as described in the methods. YD: up-regulated in YD; SP: down-regulated in YD. [file 12864_2015_1654_MOESM12_ESM.pdf]

	EdgeR			DESeq2						
	YD	SP	SUM	YD	SP	SUM	overlap YD	overlap SP	overlap SUM	*Phyper* (X >= x)
8 hpf	271	32	303	178	26	204	161	14	175	<0.0001
24 hpf	51	62	113	20	48	68	18	33	51	<0.0001
32 hpf	35	168	203	71	111	182	16	77	93	<0.0001
