# Supplementary material for: Partial depletion of yolk during zebrafish embryogenesis changes the dynamics of methionine cycle and metabolic genes
Source: BMC Genomics. 2015 Jun 4;16(1):427. doi: 10.1186/s12864-015-1654-6 (PMC4455928; doi:10.1186/s12864-015-1654-6)
Supplement: Additional file 14: — Primers used for qRT PCR. Genome positions and orientation according to Zv9, Apr 2010 (www.ensembl.org). Splice site (Y) indicates that the primer overlaps with an exon-exon boundary, (N) if primer overlaps one exon only. Gonadotropin releasing hormone 2 was primed with a transcript-specific primer (gnrh2rt_tsp), all other transcripts were primed with the anchored (3′ V, V = A, C or G) oligo(dT) primer. [file 12864_2015_1654_MOESM14_ESM.pdf]

**gene symbol**	**primer name**	**sequence**	**chromosome**	**start**	**end**	**orientation**	**splice site**	**comments**
actb2	beta-act_F1	CATCCTTCTTGGGTATGGA	3	40521835	40521938	-	Y	
actb2	beta-act_F2	CAGACATCAGGGTGTCATG	3	40522885	40522993	-	Y	
actb2	beta-act_R1	AAGCATTTGCGGTGGACG	3	40521435	40521452	+	N	
actb2	beta-act_R2	CACCATCACCAGAGTCCA	3	40522254	40522271	+	N	
bhmt	bhm_F	CTGATCGCTGAGTACTTTG	21	288480	289573	-	Y	
bhmt	bhm_R	CAATGAAGCCCTGGCAGC	21	287927	287944	+	N	
egln3	egln3_F	ACTGGAATGCCAAGGAACA	17	9780206	9780889	-	Y	
egln3	egln3_R	CTGTGAGATGGCTGTGAG	17	9777726	9777826	+	Y	
fkbp5	fkbp5_F	TCAAGGCAGGACGATACAA	6	41041318	41042575	+	Y	
fkbp5	fkbp5_R	TGTTGCAGTTCTCCACTGT	6	41042727	41042745	-	N	
fosl1a	fosl1a_F	CGATGAATATCTCTCTCCTG	14	31612886	31615270	-	Y	
fosl1a	fosl1a_R	TCTTGGCGTAATGCTTAGTT	14	31611895	31611913	+	N	
gadd45ba	gadd45ba_F	AGGAGGATCTGGACGACA	11	6539366	6539383	+	N	
gadd45ba	gadd45ba_R	TTGAGAGTTAGTGACGAGG	11	6539535	6539650	-	Y	
gnmt	gnmt_F	CTGTAATCTGTCTTGGGAAC	17	49084702	49084721	-	N	
gnmt	gnmt_R	CTGTTTCAGGTCACTCTTAT	17	49079179	49082467	+	Y	
gnrh2	gnrh2_F	TGCTCAGTTGAGCAGCG	21	14028908	14028924	+	N	
gnrh2	gnrh2_R	GGGCATCCAGCAGTATTG	21	14030724	14030834	-	Y	
gnrh2	gnrh2rt_tsp	ATCAGCTTGGTGTCACTTTCTC	21	14030854	14030875	-	N	
hsp90aa1.2	h90q_F	CATCTCCAACTCCTCTGAT	20	54335017	54335035	-	N	
hsp90aa1.2	h90q_R	CCCACGCGTACTGCTCA	20	54333190	54333206	+	N	
klf10	klq_F	AGCTCGCATCTCAAAGCAC	16	56272075	56272093	+	N	
klf10	klq_R	TCAGTCTTGGGTGCGAGA	16	56272522	56272539	-	N	
lepa	lepa_F	CATTGACGGGCAAAATTTAC	18	10137942	10138054	+	Y	
lepa	lepa_R	CTGTTTAAAGTCATGTACTC	18	10138324	10138343	-	N	
mat2ab	mat2ab_F	GGCTTGCGAAACTGTTGC	5	72068970	72069080	-	Y	
mat2ab	mat2ab_R	ATCAAACCCTGATCACCAG	5	72067450	72067588	+	Y	
ndrg1b	ndr_F	GAGCTGGACATTACTGAGG	16	33816111	33817235	+	Y	
ndrg1b	ndr_R	TGAGGACCATTGGTAGGC	16	33818176	33818193	-	N	
	oligodT	TTTTTTTTTTTTTTTTTTV						
ponzr3	ponq_F	TGGTTCCGACCAGTGGTC	7	23842787	23842804	+	N	
ponzr3	ponq_R	GTCATTGCAGATAGAGTCC	7	23843101	23843810	-	Y	
sardh	sarc_F	TGGCACACAGCTGGACTT	10	10355118	10356790	+	Y	
sardh	sarc_R	GCACGCGGCGCCACTG	10	10359702	10359720	-	N	
slc16a9a	sl16a_ma_F	TGGCATTGTTGTAGGCATG	17	20490275	20490803	-	Y	used in RNAseq validation
slc16a9a	sl16a_ma_R	TGAGATCAGATCCAGACTC	17	20488089	20488107	+	N	
slc16a9a	slc16a_Rseq_F	ATTGTTGTAGGCATGGGCT	17	20490271	20490799	-	Y	used in microarray validation
slc16a9a	slc16a_Rseq_R	GCACATGCCATCAAGTTGA	17	20488220	20488238	+	N	
txnipb	txn_F	ACTCAAGAGCTCATGGCTC	16	45709230	45709340	-	Y	
txnipb	txn_R	ATATCACCCATGCCAGAGA	16	45708959	45708977	+	N	
