# Supplementary material for: Partial depletion of yolk during zebrafish embryogenesis changes the dynamics of methionine cycle and metabolic genes
Source: BMC Genomics. 2015 Jun 4;16(1):427. doi: 10.1186/s12864-015-1654-6 (PMC4455928; doi:10.1186/s12864-015-1654-6)
Supplement: Additional file 1: — Impression of YD and SP embryos at 4 time-points. A) Mean sphere volume of embryos after YD and SP treatment. In this particular batch, on average 31 % of yolk was removed from YD embryos. Error bars denote standard deviations. B) Images of the total batch (8 hpf), and samples within this batch at 23, 31 and 48 hpf. [file 12864_2015_1654_MOESM1_ESM.pdf]

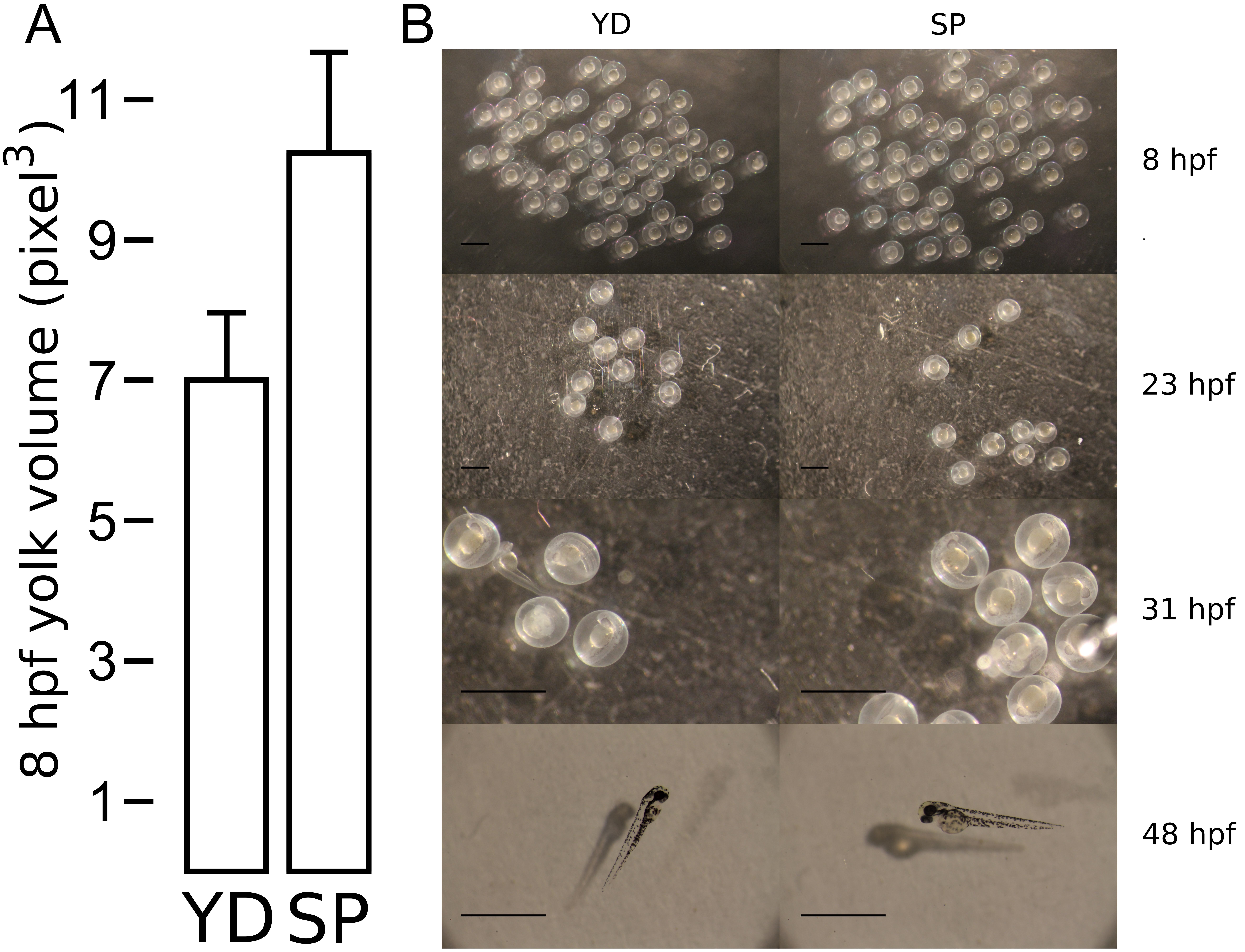
A
B
8 hpf yolk volume (pixel^3)
11
9
7
5
3
1
YD
SP
YD
SP
8 hpf
23 hpf
31 hpf
48 hpf
